# TENS alleviates CP/CPPS-related inflammation and pain by modulating Kir2.1-dependent macrophage polarization

**DOI:** 10.3389/fimmu.2025.1683500

**Published:** 2026-01-12

**Authors:** Ting Hong, Xinyu Liu, Zepai Chi, Guoyuan Liu, Yuanfeng Zhang, Xuanhao Lin, Peixiu Yao, Qingyun Gong, Yonghai Zhang, Xuwei Hong

**Affiliations:** 1 Clinical Medical Research Center, Shantou Central Hospital, Shantou, China; 2 Shantou Key Laboratory of Basic and Translational Research of Malignant Tumors, Shantou, China; 3 Department of Urology, Shantou Central Hospital, Shantou, China; 4 Department of Biobank, Shantou Central Hospital, Shantou, China; 5 Department of Pathology, Shantou Central Hospital, Shantou, China

**Keywords:** prostatitis, transcutaneous electric nerve stimulation, macrophages, potassium channels, chronic pelvic pain syndrome

## Abstract

**Introduction:**

Chronic prostatitis/chronic pelvic pain syndrome (CP/CPPS) remains a challenging urological condition. This study investigated the therapeutic potential of transcutaneous electrical nerve stimulation (TENS) in experimental autoimmune prostatitis (EAP) and explored its underlying mechanisms.

**Methods:**

Prostate tissue samples from 26 benign prostatic hyperplasia (BPH) patients were collected at Shantou Central Hospital and analyzed for M1/M2 macrophage infiltration using immunohistochemistry (IHC). In animal experiments, 24 male Sprague–Dawley rats were divided into control (*n* = 4), EAP (*n* = 7), and TENS treatment groups (*n* = 13; EAP + 1 Hz, *n* = 3; EAP + 2 Hz, *n* = 3; EAP + 4 Hz, *n* = 7). EAP was induced via prostate antigen injections, and TENS (1–4 Hz) was applied. In cell experiments, RAW264.7 macrophages were grouped into control, LPS-stimulated, electrical stimulation (ES)-treated (0.1–0.5 V/cm), and ES + pharmacological intervention (zacopride/probenecid) groups to study repolarization mechanisms.

**Results:**

In BPH tissues, the M1/M2 macrophage ratio positively correlated with inflammation severity. In EAP rats, 4 Hz TENS significantly alleviated pelvic pain, reduced proinflammatory cytokine expression, and reversed histopathological damage. TENS promoted macrophage repolarization toward the M2 phenotype. ES *in vitro* similarly induced M1-to-M2 repolarization, which was associated with decreased inwardly rectifying K+ channel Kir2.1 (Kir2.1) and Transient receptor potential vanilloid 2 (TRPV2) expression, membrane depolarization, reduced Ca^2+^ influx, and a shift from NF-κB/STAT1 to STAT6 signaling. Agonists of Kir2.1 TRPV2 reversed these effects.

**Conclusion:**

TENS alleviates CP/CPPS by promoting M1-to-M2 macrophage repolarization, representing a promising therapeutic strategy.

## 1 Introduction

Chronic prostatitis/chronic pelvic pain syndrome (CP/CPPS), classified as type III prostatitis by the National Institutes of Health (NIH), accounts for approximately 90%–95% of all prostatitis cases and predominantly affects men aged 35–45 years. The hallmark symptom of CP/CPPS is persistent pelvic or perineal pain lasting for at least 3 months (1), which significantly impairs quality of life due to its subtle and multifaceted clinical manifestations (2). Despite its high prevalence, the etiology and pathogenesis of CP/CPPS remain poorly understood, and current treatment options are limited and often ineffective. Transcutaneous electrical nerve stimulation (TENS) is a noninvasive therapeutic modality that delivers low-frequency electrical currents to the nerves or muscles. It has been reported to alleviate pain in various chronic pain disorders (3) and has shown preliminary clinical efficacy in the management of CP/CPPS (4, 5). Compared with other therapies, TENS is relatively convenient, safe, and free of significant adverse effects, providing an effective and noninvasive treatment option for patients with CP/CPPS (6). Nevertheless, the unclear mechanism of TENS in CP/CPPS treatment limits its broader clinical application.

Accumulating evidence underscores the critical involvement of immune dysregulation in the pathogenesis of CP/CPPS, particularly macrophage-mediated mechanisms (7–9). Macrophages are highly plastic immune cells that can polarize into classically activated M1 or alternatively activated M2 phenotypes in response to microenvironmental cues (10). M1 macrophages secrete proinflammatory cytokines, such as tumor necrosis factor α (TNF-α), interleukin (IL)-1β, and IL-6, contributing to tissue damage and pain, whereas M2 macrophages promote tissue repair by releasing anti-inflammatory cytokines, such as IL-10. Clinical observations revealed that the levels of macrophages and chemokines in prostatic fluid are positively correlated with chronic prostatitis symptom indices and pain scores (11). Notably, a chronic inflammatory microenvironment facilitates neural sensitization through sustained cytokine release, potentially driving the characteristic CP/CPPS symptomatology (9). Therapeutic interventions targeting macrophage polarization have shown promising results, such as blocking macrophage-mediated TNF-α to ameliorate prostate inflammation and epithelial hyperplasia (12), or enhancing macrophage-mediated IL-10 to attenuate inflammation (13). Therefore, regulating the dynamic equilibrium between M1 and M2 polarization states appears crucial in reversing CP/CPPS.

Macrophages possess electrical characteristics, and electric field-based alterations in cell membrane potential could modulate cell functions, including macrophage polarization, migration, differentiation, and phagocytosis (14). For instance, piezoelectric scaffolds or ultrasound-activated nanomaterials have been shown to promote anti-inflammatory macrophage polarization via pathways such as Mitogen-activated protein kinase (MAPK)/JNK inhibition and mitochondrial depolarization (15, 16). Additionally, electrical stimulation (ES) can modulate macrophage reprogramming by altering ionic distribution, affecting the cell membrane potential, and further regulating the activity of ion channels as well as the binding state or distribution of membrane receptor proteins (17). For example, ES influences the influx of Ca^2+^ ions through voltage-gated ion channels, establishing a Ca^2+^-CAMK2A-Nuclear factor kappa B (NF-κB) axis that drives macrophage polarization (18). Moreover, macrophages exhibit remarkable plasticity, adapting their phenotypes and transcriptional programs in response to microenvironmental signals (19). This plasticity has spurred interest in targeted therapies, with epigenetic modulators like histone deacetylase inhibitors (HDACi) and DNA methyltransferase inhibitors (DNMTi) emerging as promising treatments for inflammatory diseases by modulating macrophage function (20). These findings suggest that TENS may exert therapeutic effects in CP/CPPS by modulating macrophage polarization through similar electro-immunological mechanisms.

In this study, we investigated the role of macrophage reprogramming in the therapeutic effects of TENS in a rat model of experimental autoimmune prostatitis (EAP) and further explored the direct impact of electrical stimulation on macrophage phenotypic switching *in vitro*. Our findings provide novel mechanistic insights into the anti-inflammatory and analgesic effects of TENS and support its potential as a nonpharmacological intervention for CP/CPPS.

## 2 Materials and methods

### 2.1 Clinical tissue samples collection

Approval for the use of human tissues was obtained from the ethics committee of Shantou Central Hospital, with written informed consent obtained from all participants. Given the challenges in obtaining prostate tissue from CP/CPPS patients, prostate tissue specimens were collected from 26 patients diagnosed with benign BPH who underwent transurethral resection of the prostate (TURP) at Shantou Central Hospital between 2020 and 2024. These specimens were used as surrogate samples, as BPH often presents with inflammatory features. IHC was used to investigate M1 and M2 macrophage infiltration. The cohort included nine cases with mild, 11 with moderate, and six with severe inflammatory cell infiltration. Patients with preoperative urinary tract infection, catheterization, incidental prostate cancer, prostatic intraepithelial neoplasia, or prior pharmacotherapy were excluded. The diagnosis of BPH was confirmed independently by two pathologists using hematoxylin and eosin (H&E) staining, following the North American Chronic Prostatitis Collaborative Research Network and International Criteria (21). Detailed clinical information of the enrolled patients is summarized in **Table 1**.

**Table 1:** Baseline characteristics of BPH patients.

Parameter	Mild (*n* = 9)	Moderate (*n* = 11)	Severe (*n* = 6)
Age (years)	64.11 ± 1.98 b	74.55 ± 1.65 a	70.17 ± 2.69 a,b
NIH-CPSI			
Total scores	7.44 ± 1.20 c	22.27 ± 1.05 b	36.33 ± 1.31 a
Pain domain	2.89 ± 0.51 c	11.36 ± 0.66 b	17.83 ± 0.48 a
Urination domain	1.56 ± 0.24 c	4.73 ± 0.27 b	8.17 ± 0.31 a
QoL domain	3 ± 0.50 c	6.18 ± 0.40 b	10.33 ± 0.67 a
IPSS			
Total scores	5.22 ± 0.36 c	10.73 ± 0.92 b	23.1 ± 2.14 a
Obstruction symptom	2.9 ± 0.31 c	5.64 ± 0.53 b	13.33 ± 1.41 a
Irritation symptom	2.33 ± 0.17 c	5.09 ± 0.41 b	9.83 ± 0.83 a

Data are shown as the mean ± SEM. Statistical analysis was performed using one‐way ANOVA with Tukey’s test, and different letters (a–c) represent a significant difference (*p* < 0.05) in the comparison. *NIH-CPSI*, National Institutes of Health Chronic Prostatitis Symptom Index; *IPSS*, International Prostate Symptom Score; *QoL*, quality-of-life domain.

### 2.2 Prostate tissue inflammation evaluation

Assessment was done following the standards recommended by the North American Chronic Prostatitis Collaborative Research Network and the International Prostatitis Collaborative Network (21). Inflammation severity of BPH prostate tissue samples, based on H&E staining, was categorized as shown in **Table 2**.

**Table 2:** Criteria for determining the degree of prostate tissue inflammation.

Grading standard	Morphological description	Cell density
1. Mild	Scattered inflammatory cells	< 100 cells/mm^2^
2. Moderate	Merged inflammatory cell clusters	~ 100–500 cells/mm^2^
3. Severe	Inflammatory cell clusters with tissue destruction or formation of lymphoid nodules/follicles	> 500 cells/mm^2^

### 2.3 Animal experiments

#### 2.3.1 Animals and experimental design

Twenty-four male Sprague–Dawley (SD) rats (6–8 weeks old, 180–240 g) were purchased from the Guangdong Laidi Biomedical Research Institute Co. Ltd. (Guangdong, China). All animals were housed under specific pathogen-free (SPF) conditions (23 °C ± 3 °C, 60% ± 10% humidity) with *ad libitum* access to food and water and were allowed to adapt to the new environment before the experiment. Rats were randomly divided into the following groups: control (*n* = 4), EAP (*n* = 7), and TENS treatment groups (*n* = 13; including EAP + 1 Hz TENS, *n* = 3; EAP + 2 Hz TENS, *n* = 3; EAP + 4 Hz *TENS, n* = 7). This preliminary screening approach, which utilized smaller subgroup sizes, aligns with the 3R principles to efficiently identify the optimal stimulation parameter while minimizing animal use (22). Subsequent analyses of the primary outcomes were focused on the most efficacious frequency group identified through this process.

#### 2.3.2 EAP model establishment

The EAP model was induced as previously described. Briefly, rats in the EAP and TENS groups received daily subcutaneous injections of 0.1 mL prostate antigen (PAg) mixture near the base of the penis, alternating sides, for 7 consecutive days. Control rats received equal volumes of physiological saline via the same route and schedule.

#### 2.3.3 TENS treatment protocol

Starting from day 14 postmodeling, rats in the TENS groups received electrical stimulation treatment. Following anesthesia induction with 4% isoflurane and maintenance with 2% isoflurane, electrode pads were affixed bilaterally to the skin at the base of the penis and connected to the Madlab bioinformation medical signal acquisition and processing system (MADLAB-4C/5H, Zhongshi Technology, Beijing, China). Stimulation parameters were set at 20 min, 3 mA current intensity, 250 μs pulse duration, with frequencies of 1, 2, and 4 Hz applied to the respective subgroups. Each treatment session lasted 20 min and was administered every 3 days for a total of eight sessions.

#### 2.3.4 Tissue collection and processing

On day 36 postmodeling, rats were anesthetized via intraperitoneal injection of 2.5% tribromoethanol (12 mL/kg). Infrared imaging was performed to assess prostate inflammation. Subsequently, blood was collected from the abdominal aorta, and euthanasia was completed via exsanguination. Tissues, including prostate, bladder, seminal vesicle, testis, heart, liver, spleen, lungs, and kidneys, were harvested for further analysis. Blood samples were centrifuged to obtain serum and stored at − 80°C for subsequent analysis. Prostate tissue was divided into two portions after saline washing: one portion was fixed in 4% paraformaldehyde for paraffin embedding, whereas the other was processed immediately for flow cytometry analysis. All other collected tissues were fixed in 4% paraformaldehyde.

### 2.4 Chronic prostatitis pain assessment

Referred hyperalgesia was quantified using Von Frey filaments (Aesthesio, DanMic, San Jose, USA) on days 0, 7, 14, 21, 28, and 35 with forces of 1, 2, 4, 8, 15, and 26 g (23). Stimulation was performed when the rats remained quiet, with the abdomen and scrotum resting on the bottom of the cage. The filament was applied perpendicularly to the pubic region or scrotal base and was bent into a “C” or “S” shape. The procedure was repeated five times, with each simulation lasting 2 s and an interval of 5 s. Here, positive responses were defined by a series of behaviors, including sudden abdominal contraction, immediate licking or grasping the stimulation site, and jumping (24). The 50% withdrawal thresholds were determined using the “up–down” method as previously described (25) and calculated on a freely accessible website tool (https://bioapps.shinyapps.io/von_frey_app/) (26).

### 2.5 Infrared thermal imaging detection

After intraperitoneal injection of anesthesia with 2.5% tribromoethanol (12 mL/1 kg), the rats were positioned supine position on a constant-temperature heating device at 37°C (CX31 701013-1, Beijing Yi Zejia Technology Co. Ltd., Beijing, China). Following exposure of the prostate tissues, images were obtained, and tissue temperature was measured via infrared thermography (VarioCAM hr, InfraTec, Dresden, Germany).

### 2.6 Histology and immunohistochemistry staining

Collected tissues (prostate, bladder, seminal vesicle, testis, heart, liver, spleen, lungs, and kidneys) were fixed in 10% formalin for 24 h and processed for paraffin embedding. Tissues were sectioned at 5 µm thickness and stained with H&E for pathological examination under light microscopy.

For immunohistochemistry (IHC), prostate tissue sections were processed according to the kit’s instructions. Sections were incubated overnight at 4°C with primary antibodies against CD86 (bs-1035R, Bioss, Beijing, China), CD206 (18704-1-AP, Proteintech, Wuhan, China), TNF-α (GB115701, Servicebio, Wuhan, China), IL-1β (GB11113, Servicebio, Wuhan, China), IL-6 (GB11117, Servicebio, Wuhan, China), Cyclooxygenase-2 (COX-2) (Abcam, ab179800, Cambridge, UK), substance P (SP; Proteintech, Wuhan, China, 13839-1-AP), or Kir2.1 (19965-1-AP, Proteintech, Wuhan, China). Subsequently, sections were incubated with HRP-conjugated goat antirabbit antibody (GB23303, Servicebio) for 30 min at room temperature. After being washed three times with phosphate-buffered saline (PBS), the immune complexes were further stained with DAB (36302ES01, Yeason, Shanghai, China) and counterstained with hematoxylin (G1004, Servicebio, Wuhan, China). All histological and IHC analyses were performed by an investigator blinded to the experimental groups to minimize observer bias. Positive cells were quantified using QuPath (v0.2.0) software and counted per high-power field (HPF) at × 200 magnification.

### 2.7 Enzyme−linked immunosorbent assay

The concentrations of TNF-α, IL-1β, IL‐6, IL‐10, COX-2, SP, and Prostate-specific antigen (PSA) were measured using enzyme-linked immunosorbent assay (ELISA) kits. Blood samples and culture media were centrifuged at 2,000×*g* for 10 min, and the supernatants were collected and stored in a refrigerator at − 80°C for further use. The ELISA kits are listed in **Supplementary Table S1**.

### 2.8 Cell culture and processing

The macrophage cell line RAW264.7 (CL-0190, Pricella, Wuhan, China) was maintained in specialized medium (CM-0190, Pricella, Wuhan, China) at 37°C under 5% CO_2_. To investigate the impact of ES on macrophage repolarization, cells were first polarized to the M1 phenotype by 6-h stimulation with 50 ng/mL lipopolysaccharide (LPS; GC205009, Servicebio, Wuhan, China). Subsequently, cells were divided into five experimental groups: the control group remained untreated throughout; the LPS group received only the initial 6-h LPS stimulation; and the ES group underwent LPS stimulation followed by 1-h electrical stimulation (0.1, 0.25, 0.4, or 0.5 V/cm). For pharmacological interventions, the Za group received 100 nM zacopride (HY-103137, MCE, Monmouth Junction, USA), while the Pro group was treated with 2.5 μM probenecid (HY-B0545, MCE, Monmouth Junction, USA); both compounds were added 1 h post-ES and maintained for 16 h of incubation before membrane potential and calcium ion detection.

### 2.9 Cell Counting Kit‐8 assay

Cell viability was assessed 24 h after the 1-h electrical stimulation treatment using the Cell Counting Kit-8 (CCK-8) assay (Cat. No. G1613-1ML, Servicebio, Wuhan, China). Briefly, 10 μL of CCK-8 solution was added to each well of 96-well plates containing 100 μL of culture medium, 24 h post-ES treatment. The plates were incubated at 37°C for 2 h, and absorbance was measured at 450 nm using a microplate reader. Cell viability was calculated as a percentage relative to the control group.

### 2.10 Flow cytometry

Prostate tissues were digested with collagenase A and then subjected to differential centrifugation to obtain single-cell suspensions. Cells were stained with anti-CD86 (13395-1-AP, Proteintech) or anti-CD206 (18704-1-AP, Proteintech, Wuhan, China) antibodies for 1 h at 4°C, followed by staining with Fluorescein Isothiocyanate (FITC)-conjugated goat antirabbit antibody (A0562, Beyotime, Shanghai, China) for 0.5 h at 4°C. RAW264.7 cells were preincubated with Fc-block (101319, Biolegend, San Diego, USA) followed by FITC-conjugated anti-CD86 antibody (11-0862-81, Invitrogen, Carlsbad, USA) or APC-conjugated anti-CD206 antibody (17-2061-80, Invitrogen, Carlsbad, USA). All stained cells were captured via a flow cytometer (CytoFlex LX, Beckman Coulter, Brea, USA), and the data were analyzed using FlowJo (v10.0.7) software.

### 2.11 Western blot and immunoprecipitation

RAW264.7 cells were lysed on ice for 30 min in RIPA buffer (E121-01, Genstar, Beijing, China) supplemented with protease inhibitor (IPVH00005, Servicebio, Wuhan, China) and phosphatase inhibitor (G2007, Servicebio, Wuhan, China). The lysates were then centrifuged at 12,000×*g* at 4°C to collect the supernatant, combined with loading buffer (G2075, Servicebio, Wuhan, China), and boiled for 10 min before being subjected to sodium dodecyl sulfate–polyacrylamide (SDS-PAGE) gel electrophoresis. Subsequently, gels were transferred onto PVDF membranes (1620177, Millipore, Burlington, USA). The membranes were blocked with blocking buffer (P0252, Beyotime, Shanghai, China) and incubated with primary antibodies at 4°C overnight on a shaking platform. After washing with TBST, the membranes were incubated with HRP-conjugated goat antirabbit IgG (H + L) secondary antibodies (GB23303, Servicebio, Wuhan, China) (1:10,000) for 1 h and visualized with an iBright imager (ChemiDoc, Bio-Rad, Hercules, USA). The primary antibodies are listed in **Supplementary Table S2**.

### 2.12 Cell membrane potential measurement

After completing the full treatment course (6 h LPS induction, 1 h ES, and 16 h incubation with or without activators), RAW264.7 cells were collected for membrane potential measurement. Cells were suspended in PBS and incubated with working DiBAC4 (3) solution (5 μmol/L; Cat No. HY-101892, MCE) at 37°C for 45 min. Subsequently, the cell membrane potential was observed using a fluorescence microscope, and the mean fluorescence intensity was quantified via flow cytometry.

### 2.13 Intracellular Ca^2+^ measurement

Intracellular Ca^2+^ levels were assessed at the same experimental endpoint. Following the full treatment protocol, RAW264.7 cells were collected, and their intracellular Ca^2+^ concentration was measured using the Fluo-4 Calcium Assay Kit (Cat No. S1061M, Beyotime, Shanghai, China) according to the manufacturer’s instructions. Cells were also observed under a fluorescence microscope, and mean fluorescence intensity was measured via flow cytometry.

### 2.14 Quantitative proteomic analysis

Quantitative proteomic analysis was performed on RAW264.7 cells from the LPS and ES groups by Beijing Novogene Technology Co. Ltd. (Beijing, China). Briefly, proteins were extracted using DB lysis buffer and DTT solution. The samples were then analyzed on a Vanquish Neo nanoflow UHPLC system equipped with a C18 precolumn (174,500, 5 mm × 300 μm, 5 μm, Thermo, Waltham, USA) and a C18 analytical column (ES906, PepMap TM Neo UHPLC 150 μm × 15 cm, 2 μm, Thermo, Waltham, USA), followed by detection with a Thermo Orbitrap Astral mass spectrometer using an Easy-spray (ESI) ion source. Data processing was carried out with DIA-NN software, in which only peptide–spectrum matches (PSMs) exceeding 99% confidence were retained. Peptide and protein identifications were filtered at a false discovery rate (FDR) of < 1%. Protein quantification was performed with cross-run normalization. Differentially expressed proteins (DEPs) were identified using a two-sample *t*-test with *p* < 0.05. The complete list of DEPs is provided in **Supplementary Table S4**.

### 2.15 Gene Ontology classification of DEPs

Gene Ontology (GO) and Kyoto Encyclopedia of Genes and Genomes (KEGG) pathway enrichment analyses of the identified DEPs were performed using the clusterProfiler package (version 4.0) in R. The analysis included three categories: biological process (BP), cellular component (CC), and molecular function (MF). Statistical significance was determined via a hypergeometric test, with GO terms and KEGG pathways having *p* < 0.05 considered significantly enriched. To estimate the corresponding type I error rate, Benjamini–Hochberg FDR (padj) was further calculated. For visualization, GO enrichment results were plotted with –log_10_ (padj) to enhance the interpretability of significance. The GO categorization of the DEPs identified from these tissues is summarized in **Supplementary Table S4**.

### 2.16 Statistical analysis

All experiments in the present study were performed three times. Data are presented as mean ± SEM. Statistical analyses were performed using GraphPad software. Comparisons between two groups were analyzed using unpaired two-tailed Student’s *t*-tests. Multiple group comparisons were conducted using one-way ANOVA followed by Tukey’s *post-hoc* test. Longitudinal behavioral data were analyzed using a mixed-effects model to account for repeated measures. Behavioral pain assessments involving repeated measurements over time were analyzed using mixed-effects models to account for within-subject correlations. Correlations between the M1/M2 macrophage ratio and histopathological inflammation grade were assessed using Spearman’s rank correlation coefficient. Statistical significance was set at *p* < 0.05.

## 3 Result

### 3.1 Macrophage polarization is involved in the development of prostate tissue inflammation

**Figure 1:**
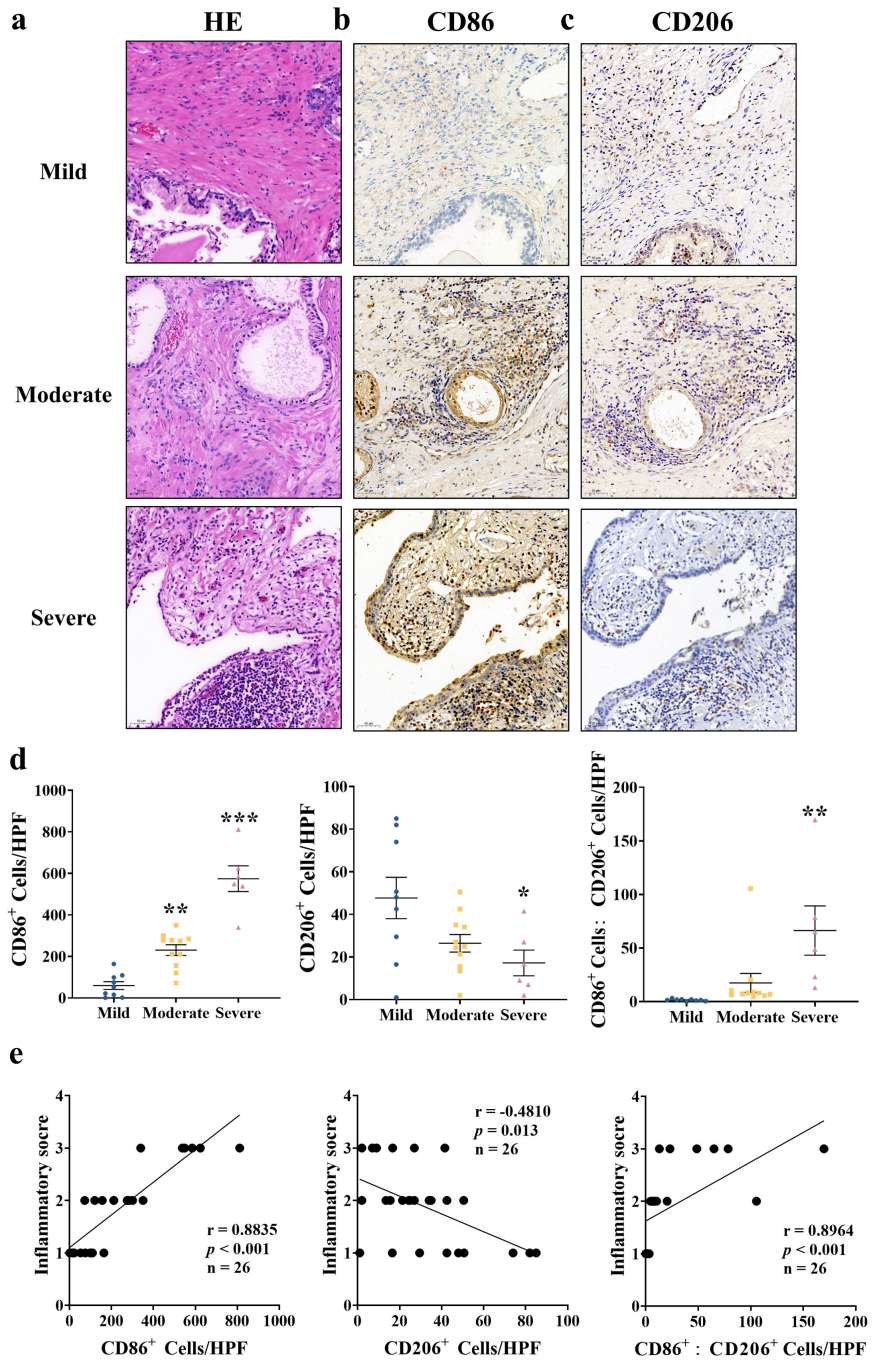
The ratio of CD86^+^ M1 macrophages is positively correlated with prostate tissue inflammation in patients. **(a)** Representative H&E images depicting the evaluation of inflammation grades in prostate tissue. **(b**, **c)** Representative IHC images of CD86^+^ M1 macrophages and CD206^+^ M2 macrophages in prostate tissue of patients with mild, moderate, and severe inflammation, respectively. Scale bars: 50 μm. **(d)** Quantitative analyses of CD86^+^ M1 macrophages, CD206^+^ M2 macrophages, and the M1/M2 ratio in prostate tissue. CD86^+^ M1 and CD206^+^ M2 cells were counted per high-power field (HPF) at × 200 magnification. **(e)** Spearman’s rank correlation analysis between inflammation scores and CD86^+^ cell counts, CD206^+^ cell counts, or M1/M2 ratios. Data are shown as the mean ± SEM (*n* = 9 mild, 11 moderate, 6 severe) and were analyzed using a two-tailed unpaired Student’s *t*-test. ^*^p < 0.05, ^**^p < 0.01, and ^***^p < 0.001 *vs*. the mild group.

To investigate the role of macrophage polarization in prostate tissue inflammation, we analyzed the presence of CD86^+^ M1 and CD206^+^ M2 macrophages in BPH tissue samples exhibiting varying degrees of inflammatory cell infiltration (mild, moderate, and severe). H&E staining revealed that tissue inflammation severity could be categorized into mild, moderate, and severe grades (**Figure 1a**). IHC analysis revealed distinct patterns of macrophage polarization across the different grades of prostate inflammation. Specifically, the number of CD86^+^ M1 macrophages was significantly elevated in both moderate and severe inflammation groups compared with mild cases (**Figures 1b, d**; *p* < 0.05). Conversely, CD206^+^ M2 macrophages were markedly decreased in the severe inflammation group relative to mild cases (**Figures 1c, d**; *p* < 0.05). Meanwhile, the M1/M2 macrophages ratio increased progressively with inflammation severity, exhibiting a positive correlation with inflammation severity in prostate tissue (**Figure 1d**; *p* < 0.05). Furthermore, Spearman’s rank correlation analysis demonstrated a strong positive correlation between the number of CD86^+^ cells and inflammation score (*r* = 0.8835, *p* < 0.001), a negative correlation between CD206^+^ cell count and inflammation score (*r* = − 0.4810, *p* = 0.013), and a robust positive correlation between the M1/M2 macrophage ratio and inflammation severity (*r* = 0.8964, *p* < 0.001; **Figure 1e**). These data suggest that macrophage polarization may be involved in the infiltration of inflammatory cells and the development of inflammation in prostate tissue.

### 3.2 TENS alleviates EAP-induced prostate inflammation and pain

**Figure 2:**
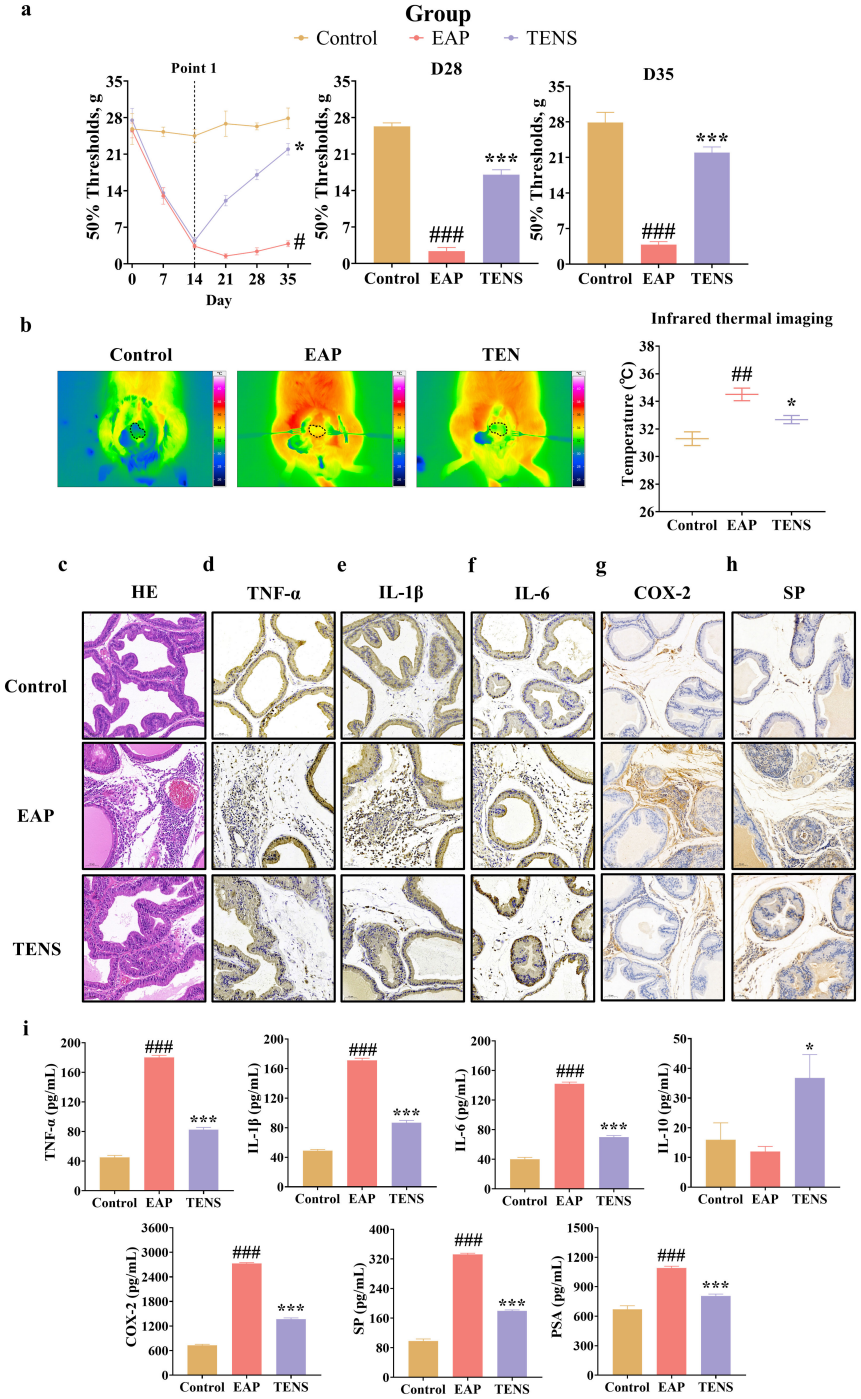
TENS ameliorated prostate inflammation in EAP rats. **(a)** Time-course analysis of 50% mechanical withdrawal thresholds in control, EAP, and TENS-treated EAP rats, with magnified comparisons at day 28 (D28) and day 35 (D35). Behavioral pain assessments involving repeated measurements over time were analyzed using mixed-effects models to account for within-subject correlations, whereas behavioral pain assessments of individual timepoints were analyzed using one-way ANOVA followed by Tukey’s *post-hoc* test. **(b)** Representative infrared thermal images showing the prostate area (outlined by dotted lines), along with corresponding quantitative analysis of thermal intensity. **(c)** Representative H&E images of prostate tissue. **(d**–**h)** Representative IHC images of TNF-α, IL-1β, IL-6, COX-2, and SP proteins in prostate tissue. Scale bars: 50 μm. **(i)** ELISA analyses of the blood TNF-α, IL-1β, IL-6, IL-10, COX-2, SP, and PSA proteins. Data are shown as the mean ± SEM and were analyzed using one-way ANOVA followed by Tukey’s *post-hoc* test. ^##^*p* < 0.01 and ^###^*p* < 0.001 *vs*. the control group. ^*^p < 0.05 and ^**^p < 0.001 *vs*. the EAP group (*n* = 3 per group).

To evaluate TENS effects on mechanical allodynia in EAP rats, 50% mechanical withdrawal thresholds were measured over 35 days. During the experiment, EAP rats exhibited a progressive decrease in thresholds, reaching the lowest at day 21 and remaining significantly lower than controls on days 28 and 35 (**Figure 2a**; **Supplementary Figure S1b**; *p* < 0.05). In contrast, TENS treatment (initiated at day 14) induced a gradual increase in thresholds, with a significant elevation by day 35 compared to untreated EAP rats (*p* < 0.05), approaching control levels, indicating that TENS alleviated mechanical allodynia. Notably, TENS treatment, particularly at 4 Hz, significantly decreased allodynic responses in EAP rats compared to the untreated EAP group (**Supplementary Figure S1b**; *p* < 0.05). Infrared imaging also showed attenuated prostate inflammation in the TENS-treated EAP rats (4Hz) compared to the EAP group (**Figure 2b**; *p* < 0.05). Histological analysis (H&E staining) of prostate tissue revealed a regular structure with no inflammatory changes in the control group. In contrast, the EAP group exhibited disordered gland cavities, angiogenesis, and extensive inflammatory cell infiltration. TENS treatment significantly alleviated these pathological features, leading to reduced inflammatory cell infiltration and restoration of tissue architecture (**Figure 2c**). Furthermore, IHC and ELISA analyses revealed that TENS treatment significantly reduced the elevated levels of proinflammatory cytokines TNF-α, IL-6, and IL-1β in the prostate tissue of EAP rats (**Figures 2d–f, i**; *p* < 0.05). Similarly, the expression of COX-2 and SP was markedly inhibited by TENS (**Figures 2g–i**; *p* < 0.05). Additionally, TENS treatment reduced serum PSA levels and increased serum IL-10 levels compared to the EAP group (**Figure 2i**; *p* < 0.05). Histological examination of other organs (bladder, seminal vesicle, testis, heart, liver, spleen, lung, kidney) showed no signs of inflammation or damage due to TENS treatment, confirming the safety of the intervention (**Supplementary Figures S1c, d**). Collectively, these results demonstrate that TENS effectively alleviates prostate inflammation and damage induced by autoimmunity in EAP rats.

### 3.3 TENS induces macrophage polarization of EAP rats

**Figure 3:**
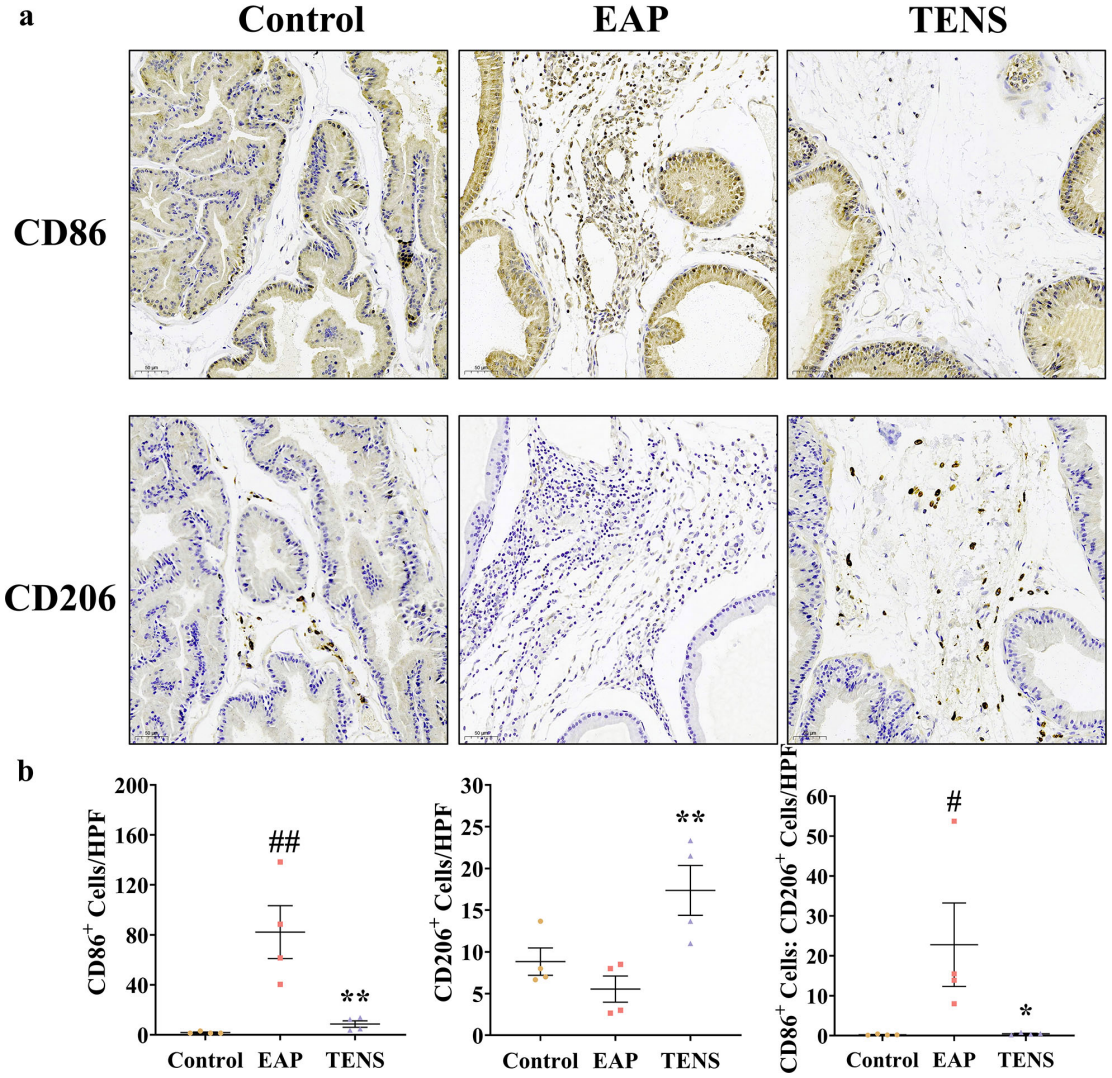
TENS induced M2 polarization of macrophages in prostate tissue. **(a)** Representative IHC images of CD86^+^ M1 macrophages and CD206^+^ M2 macrophages in rat prostate tissue. Scale bars: 50 μm. **(b)** Quantitative analyses of CD86^+^ M1 macrophages, CD206^+^ M2 macrophages, and the M1/M2 ratio in prostate tissue. CD86^+^ M1 and CD206^+^ M2 cells were counted per high-power field (HPF) at × 200 magnification. Data are shown as the mean ± SEM and were analyzed using one-way ANOVA followed by Tukey’s *post-hoc* test. ^#^*p* < 0.05 and ^##^*p* < 0.01 *vs*. the control group. ^*^p < 0.05 and ^**^p < 0.01 *vs*. the EAP group (*n* = 4 per group).

Given the crucial role of macrophage polarization in CP/CPPS pathogenesis, we investigated TENS effects on macrophage phenotype distribution in prostate tissue. Flow cytometry analysis revealed that TENS treatment increased the proportion of CD206^+^ M2 macrophages and decreased the proportion of CD86^+^ M1 macrophages in a frequency-dependent manner, relative to the EAP group (**Supplementary Figure S2**; *p* < 0.05). Consistent with these findings, IHC analysis demonstrated that TENS treatment (4 Hz) significantly reduced the number of CD86^+^ M1 macrophages while increasing the number of CD206^+^ M2 macrophages compared to the EAP group (**Figures 3a, b**; *p* < 0.05). These results suggest that TENS alleviates autoimmune prostatitis partly through promoting macrophage repolarization from proinflammatory M1 to the anti-inflammatory M2 phenotype.

### 3.4 Electrical stimulation promotes M1 to M2 macrophage repolarization *in vitro*

**Figure 4:**
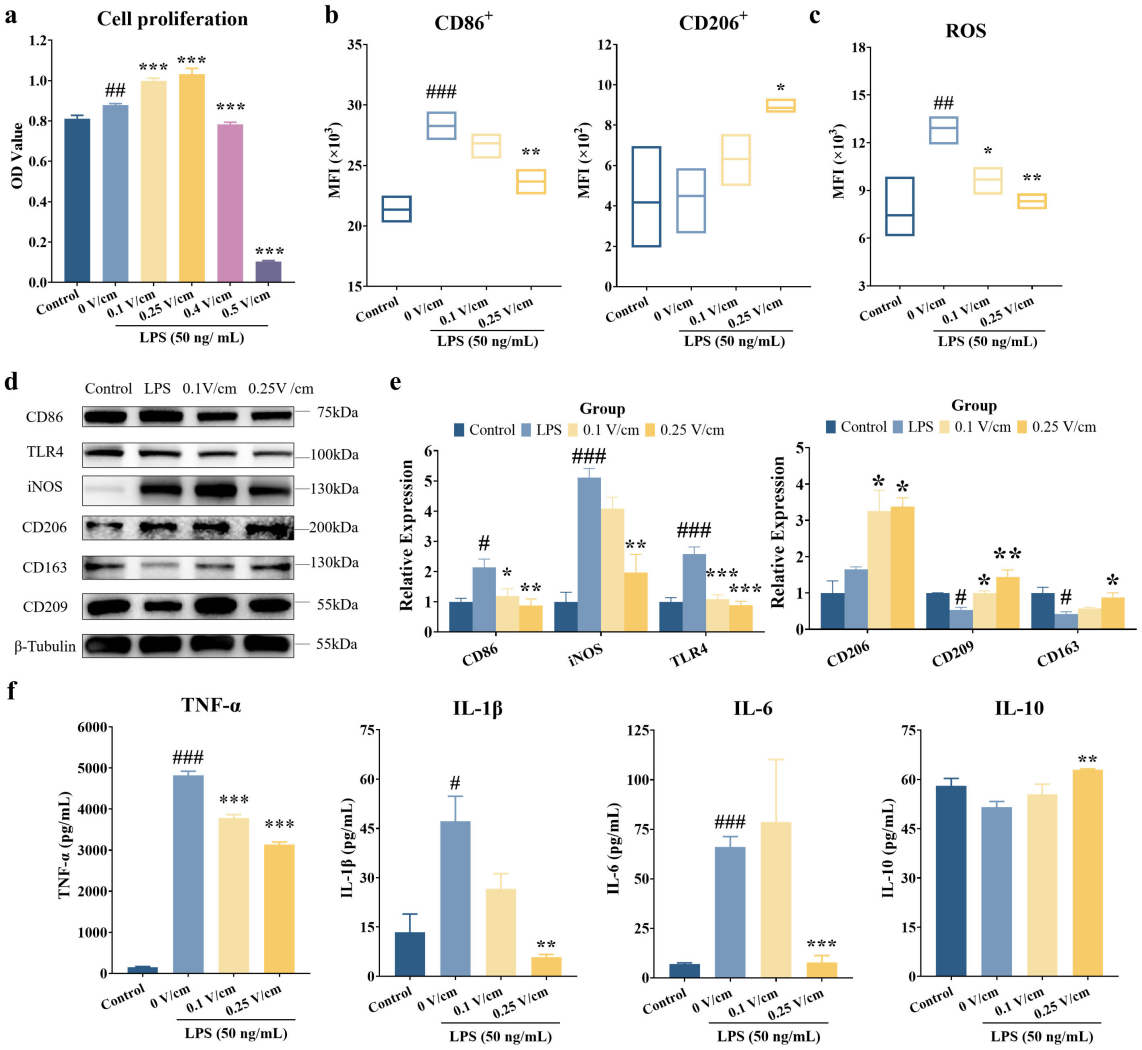
Electrical stimulation promoted the repolarization of M1 macrophages into M2 macrophages. **(a)** Cell viability after ES was assessed using the CCK8 assay. **(b)** Flow cytometry analysis of CD86^+^ M1 and CD206^+^ M2 macrophages in control, LPS-treated, and ES-treated (0.1 V/cm, 0.25 V/cm) RAW264.7 cells. MFI, mean fluorescence intensity. **(c)** Reactive oxygen species (ROS) detection in control, LPS-treated, and ES-treated (0.1 V/cm, 0.25 V/cm) RAW264.7 cells. **(d)** Western blot analysis of M1 markers (CD86, iNOS, TLR4) and M2 markers (CD206, CD163, CD209) in control, LPS-stimulated, and LPS + ES-treated (0.25 V/cm) RAW264.7 cells. **(e)** Quantitative analyses of macrophage phenotype marker expression. **(f)** ELISA analyses of TNF-α, IL-1β, IL-6, and IL-10 secretion in macrophage culture supernatants. Data are shown as the mean ± SEM and were analyzed using one-way ANOVA followed by Tukey’s *post-hoc test*. ^##^*p* < 0.01 and ^###^*p* < 0.001 *vs*. the control group. ^*^p < 0.05, ^**^p < 0.01, and ^***^p < 0.001 *vs*. the LPS group (*n* = 3 per group).

To elucidate the mechanism of TENS-mediated macrophage regulation, we investigated the effects of ES on RAW264.7 cells. Treatment with 50 ng/mL LPS significantly enhanced cell viability compared with the control group (**Figure 4a**), an effect consistent with previous reports that low-dose LPS can promote macrophage metabolic activity and survival (27). ES at intensities greater than 0.25 V/cm significantly reduced the viability of LPS-stimulated macrophages (**Figure 4a**; *p* < 0.05). Accordingly, 0.25 V/cm was selected as the optimal intensity for subsequent experiments focused on macrophage repolarization. Flow cytometry analysis revealed that ES at 0.25 V/cm reduced the mean fluorescence intensity (MFI) of CD86^+^ M1 macrophages and increased that of CD206^+^ M2 macrophages in LPS-stimulated cells (**Figure 4b**; *p* < 0.05). Additionally, ES at 0.25 V/cm significantly attenuated LPS-induced ROS production (**Figure 4c**; *p* < 0.05). Western blot analysis showed that ES at 0.25 V/cm led to a significant decrease in the expression of M1 phenotype markers (CD86, iNOS, TLR4) and a concomitant increase in the expression of M2 phenotype markers (CD206, CD163, CD209) in LPS-stimulated macrophages (**Figure 4d, e**; *p* < 0.05). Furthermore, ES suppressed the secretion of proinflammatory cytokines (TNF-α, IL-1β, IL-6) and promoted the secretion of the anti-inflammatory cytokine IL-10 (**Figure 4f**; *p* < 0.05). Taken together, these findings demonstrate that ES effectively promotes the repolarization of proinflammatory M1 macrophages toward an anti-inflammatory M2 phenotype.

### 3.5 Electrical stimulation mediates ion channels to reprogram macrophages via membrane potential modulation

To elucidate the mechanisms underlying ES-induced macrophage repolarization, we performed mass spectrometry (MS) analysis to identify DEPs between LPS-stimulated and ES-treated RAW264.7 cells. High reproducibility was observed across biological replicates (**Supplementary Figure S3a**). Of 8,576 identified proteins, 533 were differentially expressed (156 upregulated, 377 downregulated) with statistical significance (**Supplementary Table S4**; *p* < 0.05). KEGG pathway analysis revealed significant enrichment in complement and coagulation cascades, fat digestion and absorption, and MAPK signaling pathways (**Supplementary Table S4**; **Supplementary Figures S3b, c**; *p* < 0.05), all of which have previously been implicated in macrophage polarization (28–30).

**Figure 5:**
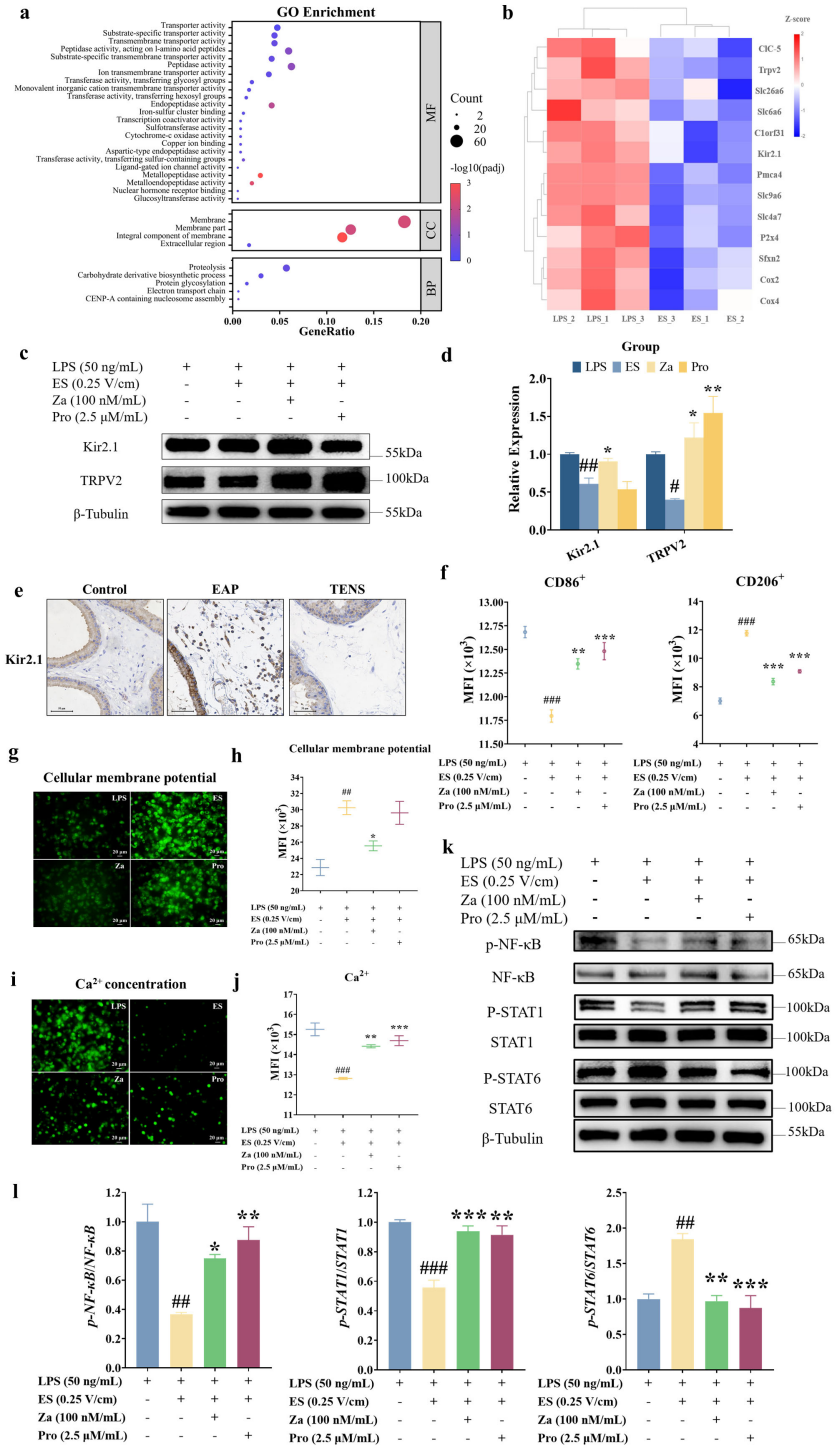
Repolarization mechanism of ES effect in macrophages. **(a)** GO classification of DEPs between the LPS and ES groups related to cellular component, molecular function, and biological process categories. **(b)** Heatmap of differentially clustered ion transmembrane transporter activity. The color scale represents the *Z*-score of normalized protein abundance; red indicates upregulation, and blue indicates downregulation. **(c)** Representative WB images of Kir2.1 and TRPV2 expression. **(d)** Quantitative analyses of Kir2.1 and TRPV2 marker expression. **(e)** Representative IHC images of Kir2.1 in rat prostate tissue. Scale bars: 50 μm. **(f)** Quantitative analyses of CD86^+^ and CD206^+^ expression in macrophages measured by flow cytometry. **(g)** Immunofluorescence staining images of cellular membrane potential in macrophages (scale bars: 20 μm). **(h)** Quantitative analysis of cellular membrane potential in macrophages measured by flow cytometry. **(i)** Immunofluorescence staining images of intracellular Ca^2+^ concentration in macrophages (scale bars: 20 μm). **(j)** Quantitative analysis of intracellular Ca^2+^ concentration in macrophages measured by flow cytometry. MFI, mean fluorescence intensity. **(k)** Representative WB images of NF-κB/STAT1/STAT6 signaling pathway expression. **(l)** Quantitative analyses of NF-κB/STAT1/STAT6 signaling pathway expression. Data are shown as the mean ± SEM and were analyzed using one-way ANOVA followed by Tukey’s *post-hoc* test. ^##^*p* < 0.01 and ^###^*p* < 0.001 *vs*. the LPS group. ^*^p < 0.05, ^**^p < 0.01, and ^***^p < 0.001 *vs*. the ES group (*n* = 3 per group).

GO enrichment analysis highlighted significant alterations in membrane-related cellular components (**Figure 5a**; **Supplementary Table S4**; *p* < 0.05). Furthermore, several GO terms associated with transporter activity were enriched, including transmembrane transporter activity, substrate-specific transporter activity, ion transmembrane transporter activity, and monovalent inorganic cation transmembrane transporter activity. Notably, previous studies have revealed that cellular membrane potential plays a critical role in macrophage differentiation (31), and that voltage-sensitive channels in macrophages mediate ion entry and influence polarization (18, 32); therefore, we focused on ion channel-related proteins. A heatmap depicting differential clustering of proteins involved in ion transmembrane transporter activity revealed downregulation of several key proteins in the ES group compared with the LPS group (**Figure 5b**), including the inwardly rectifying K^+^ channel family member Kir2.1, the Ca^2+^-permeable channel family member Trpv2, the calcium transporter C1orf31, and the mitochondrial components Cox2 and Cox4. As Kir2.1 and TRPV2 have established roles in macrophage inflammatory regulation (32, 33), we chose to further investigate their contribution to ES-induced macrophage reprogramming.

### 3.6 Downregulation of Kir2.1 is critical for ES-induced macrophage reprogramming via Ca^2+^/NF-κB/STAT signaling

To elucidate the mechanistic role of Kir2.1 and TRPV2 in ES-induced macrophage reprogramming, we employed pharmacological activation using the Kir2.1 agonist zacopride (Za) and TRPV2 agonist probenecid (Pro). ES at 0.25 V/cm downregulated Kir2.1 TRPV2 expression in LPS-stimulated RAW264.7 cells. Following LPS-induced M1 polarization and subsequent ES treatment, Za administration significantly reversed the ES-induced decrease in TRPV2 and Kir2.1 expression levels, while Pro enhanced TRPV2 expression (**Figures 5c, d**; *p* < 0.05). Notably, neither agonist affected cell viability (**Supplementary Figure S4**; *p* > 0.05), indicating that the observed effects were not due to cytotoxicity. Consistent with these *in vitro* findings, IHC revealed reduced Kir2.1 expression in macrophages from TENS-treated prostatic tissues compared to EAP controls (**Figure 5e**), suggesting ES-mediated Kir2.1 modulation *in vivo*. Flow cytometry demonstrated that both Za and Pro enhanced CD86 (M1 marker) while suppressing CD206 (M2 marker) compared with ES treatment alone (**Figure 5f**; *p* < 0.05), indicating that channel activation counteracted ES-induced anti-inflammatory reprogramming. Compared with untreated control cells, LPS stimulation alone did not significantly alter membrane potential or Kir2.1 protein levels in RAW264.7 macrophages (**Supplementary Figures S5a, c, d**), although it elevated intracellular Ca^2+^ concentrations and TRPV2 expression (**Supplementary Figures S5b–d**). Membrane potential assays further supported that ES elevated fluorescence intensity relative to LPS alone, an effect attenuated by Za (**Figures 5g, h**; *p* < 0.05), implicating Kir2.1-dependent depolarization in ES-mediated signaling. Mechanistically, ES significantly reduced intracellular Ca^2+^ levels compared with LPS stimulation, whereas Za and Pro restored Ca^2+^ concentrations beyond ES-treated levels (**Figures 5i, j**; *p* < 0.05). Western blot analysis revealed that ES suppressed LPS-induced NF-κB p65 and Signal transducer and activator of transcription 1 (STAT1) phosphorylation while enhancing Signal transducer and activator of transcription 6 (STAT6) activation (**Figures 5k, l**; **Supplementary Figures S5e, f**; *p* < 0.05). Notably, both agonists reversed these effects, with Za exerting stronger regulation. Collectively, these data establish a hierarchical signaling axis wherein ES-induced Kir2.1 activation modulates membrane potential to regulate TRPV2-mediated Ca^2+^ influx, ultimately fine-tuning NF-κB/STAT1/STAT6 dynamics to drive macrophage reprogramming.

## 4 Discussion

**Figure 6:**
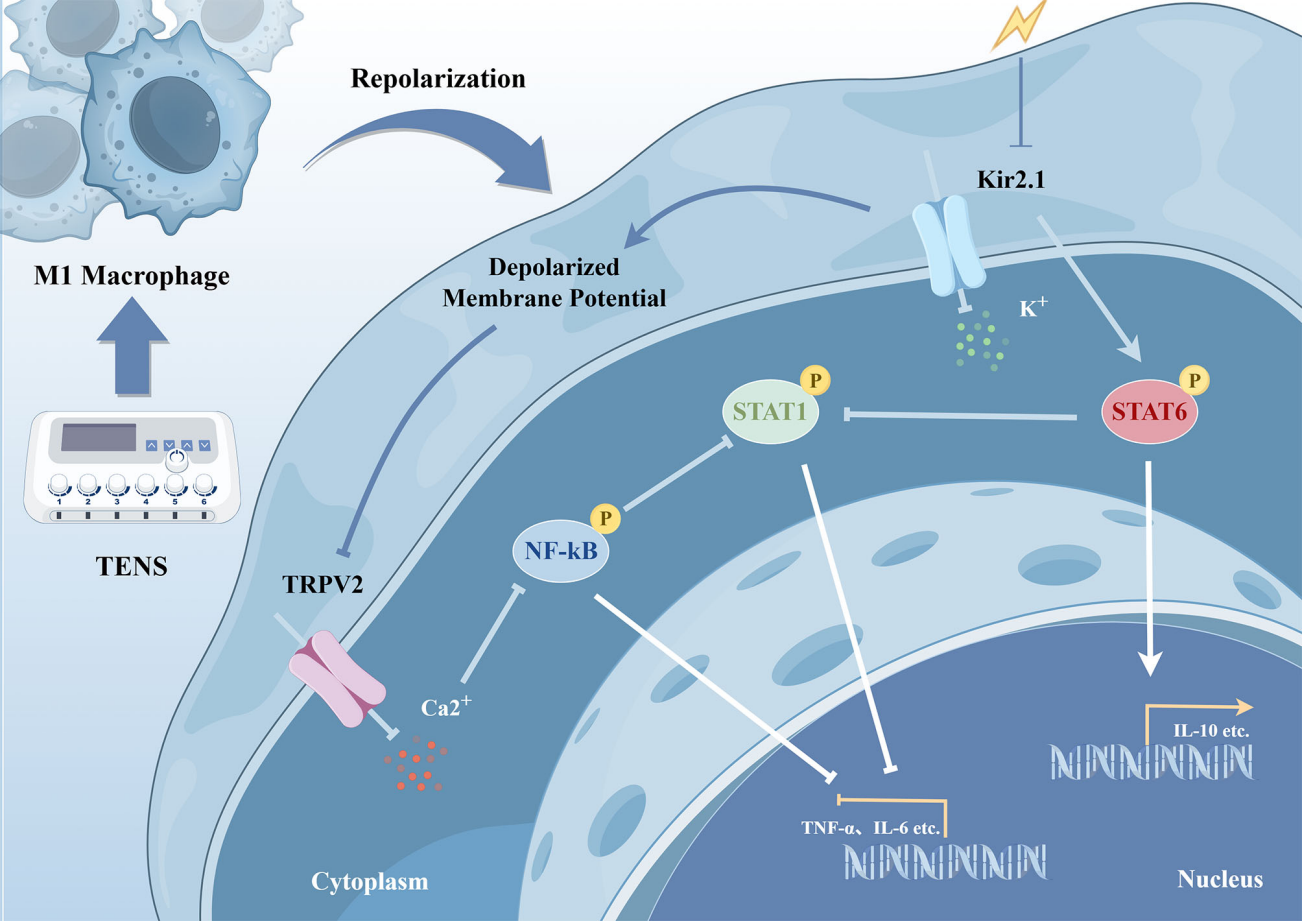
Schematic diagram depicting the underlying mechanism of macrophage repolarization in TENS treatment for CP/CPPS. The figure was created using FigDraw.

CP/CPPS, with its complex etiology and pathogenesis, still lacks clearly effective therapies to date (34). TENS, a nonpharmacological approach, has shown promise in managing CP/CPPS and is recognized as a potential treatment option (5). However, the precise mechanisms underlying TENS-mediated therapeutic effects in CP/CPPS remain incompletely elucidated, hindering the development of standardized clinical protocols and evidence-based optimization of treatment parameters, ultimately limiting broader clinical implementation. In the present study, we demonstrated the efficacy and safety of TENS in CP/CPPS using the EAP model. Macrophage reprogramming emerged as a pivotal mechanism in therapeutic interventions, regulated via cellular membrane potential through Kir2.1 mediation (**Figure 6**). These findings provide evidence that the Kir2.1 channel in macrophages may be a promising therapeutic target, contributing to the efficacy of TENS in CP/CPPS treatment. While our current data support an association between Kir2.1 modulation and macrophage reprogramming, the precise molecular interactions between Kir2.1 and TRPV2 in this context require further investigation.

As critical immune regulators, macrophages may play an immunoregulatory role in the pathogenesis of CP/CPPS, serving as key mediators of inflammatory responses (35, 36). Macrophages dynamically differentiate into functionally distinct polarization states, subsequently releasing phenotype-specific arrays of cytokines and bioactive mediators that orchestrate context-dependent immunomodulation within inflammatory microenvironments. Generally, macrophages polarize toward the M2 phenotype during the progression of inflammatory disorders, synthesizing anti-inflammatory mediators to mitigate inflammatory responses. Conversely, M1 macrophages predominantly secrete proinflammatory cytokines, thereby sustaining a proinflammatory microenvironment and aggravating tissue destruction (37). However, direct clinical evidence demonstrating that macrophage polarization contributes to the development of CP/CPPS is lacking. In the present study, using prostate tissue samples from BPH patients undergoing TURP, we verified that macrophage numbers increased with the severity of prostatic inflammation. Meanwhile, the M1/M2 macrophage ratio showed a positive correlation with inflammation severity in prostate tissue, indicating that macrophage polarization may contribute to prostatitis progression. Several studies suggest that ES may be an effective technique for modulating macrophage polarization (14). Therefore, we speculated that the regulation of macrophage reprogramming may be a crucial factor in the reversal of CP/CPPS with TENS.

Our study employed an EAP rat model, which recapitulates key histopathological features of human CP/CPPS (38), to assess the therapeutic potential of TENS. Eight TENS treatments were administered to the rats, and significant reductions in pain behavior were observed, as evidenced by improved pain threshold and mobility assessments. Proinflammatory cytokines such as TNF-α, IL-1β, and IL-6 are established mediators of inflammation in CP/CPPS (39, 40). In agreement with previous reports (41–43), we observed significantly elevated levels of these cytokines in the EAP rat model. Notably, TENS treatment markedly suppressed the secretion of TNF-α, IL-1β, and IL-6, while simultaneously enhancing the production of the anti-inflammatory cytokine IL-10, indicating a rebalancing of the local inflammatory microenvironment. Pain in CP/CPPS has been closely associated with neuroinflammatory mediators such as SP and COX-2 (23). In our study, TENS treatment significantly reduced the expression of both SP and COX-2 in prostate tissue, providing a plausible mechanistic basis for the observed improvements in pain-related behaviors in EAP rats. TENS treatment significantly reduced macrophage infiltration into the prostate, suggesting a potential role in modulating immune cell polarization. Given the established involvement of macrophage polarization in the pathogenesis of CP/CPPS (44, 45), we further investigate whether TENS exerts its effects via macrophage modulation using LPS-primed RAW264.7 cells to model M1 polarization. We found that ES effectively suppressed M1-associated markers while promoting M2 polarization. This phenotypic switch was accompanied by a decrease in proinflammatory cytokine secretion and an increase in anti-inflammatory mediators, suggesting a direct immunomodulatory effect of ES on macrophages. Collectively, our findings identify TENS as an effective nonpharmacological intervention for CP/CPPS, alleviating prostatic inflammation and pain *in vivo* and modulating macrophage polarization *in vitro*.

Emerging evidence suggests that ES modulates cell membrane potential (14), which may regulate macrophage function. Ion channels, expressed beyond excitable cells and sensitive to membrane potential (46), could mediate this process by translating ES into polarization signals (17, 47). Our proteomic data support this notion, showing downregulation of key ion transport proteins following ES. Furthermore, two key mediators emerged from this analysis: the potassium channel Kir2.1 and the calcium channel TRPV2. TRPV2 expression could be regulated by Kir2.1 activation. This observation aligns with the established biophysical principle that ES-induced ionic redistribution alters transmembrane potential gradients, subsequently modulating the activity of voltage-sensitive ion channels (48). Mechanistically, Kir2.1 channel maintains membrane potential via regulation of potassium influx (49), which might serve as a potential therapeutic target for inflammatory diseases (50). It plays a pivotal role in establishing membrane potential, supporting Ca^2+^ influx to promote M1 macrophage polarization through activation of the NF-κB signaling pathway (32). Conversely, inhibition of Kir2.1 promotes the phosphorylation of STAT6, inducing M2 macrophage polarization. Simultaneously, TRPV2 inhibition observed in our study reduces extracellular calcium influx, a critical driver of NF-κB signaling and cytokine production in macrophages (33). In the present study, we unveil a coordinated bioelectrical regulatory axis: ES regulates Kir2.1 channel to alter cell membrane potential in macrophages and suppresses TRPV2 to restrict Ca^2+^ influx, thereby facilitating macrophage reprogramming. Zacopride is classically described as an agonist that acutely activates the Kir2.1 channel, whereas probenecid serves as an agonist that gates the TRPV2 channel (51, 52). Several recent reports indicate that sustained agonist exposure can secondarily upregulate Kir2.1 and TRPV2 expression, respectively (53, 54). This phenomenon, observed in our experiments in which zacopride elevated Kir2.1 and probenecid elevated TRPV2 protein, might involve agonist-induced stabilization of the channel protein or activation of downstream signaling pathways that promote its transcription or translation. Overall, the Kir2.1 channel in macrophages might represent a promising therapeutic target, and Kir2.1-mediated macrophage reprogramming could contribute to the efficacy of TENS in the treatment of CP/CPPS.

Despite the promising findings, this study has several limitations. First, BPH tissues were used as substitutes for CP/CPPS samples due to limited clinical availability. Although BPH with chronic inflammation shares some pathological features with CP/CPPS, it may not fully represent its autoimmune and pain-related mechanisms. Future studies should include prostate biopsies or urinary immune profiling from diagnosed CP/CPPS patients to validate the clinical relevance of these findings. Second, during the preliminary phase for parameter screening, animal subgroup sizes were small (n = 3–7), which may limit the statistical power for those specific comparisons. However, these initial experiments were designed for optimization, and all main conclusions regarding TENS efficacy and the central role of macrophage Kir2.1 are supported by subsequent experiments with adequate sample sizes. Moreover, the selected treatment parameters were grounded in established clinical practices and prior preclinical evidence (22). Future studies with dedicated CP/CPPS clinical specimens and larger animal cohorts are warranted for definitive validation. Our EAP model experiments show TENS has anti-inflammatory and analgesic effects; however, future studies employing macrophage-specific Kir2.1 knockout mice or targeted blockers are required to determine whether Kir2.1 inhibition alone can reproduce TENS-mediated effects in CP/CPPS. Additionally, future investigations incorporating single-cell RNA sequencing or broader proteomic profiling would be valuable to delineate the full diversity of macrophage subpopulations involved in CP/CPPS pathogenesis and TENS-mediated resolution.

## 5 Conclusion

TENS administration effectively modulated macrophage reprogramming in the treatment of CP/CPPS *in vitro* and *in vivo*. Mechanistically, the Kir2.1-Ca^2+^-NF-κB signaling axis was identified as a key regulatory pathway. These findings highlight the Kir2.1 channel as a promising therapeutic target that could potentially enhance the clinical efficacy of TENS-based interventions for CP/CPPS management.

## Data Availability

The original contributions presented in the study are included in the article/**Supplementary Material**. Further inquiries can be directed to the corresponding authors.

## Supplementary material

Supplementary Figure 1 EAP rats with TENS treatment showed efficacy, specificity and safety *in vivo*. **(a)** Schematic illustration of *in vivo* experiment. **(b)** Assessment of abnormal pain sense of prostate. Point 1: The time point of the first TENS treatment. Curves of behavioral pain assessments were performed using mixed-effects models. **(c)** Representative HE images of bladder, spermatophore and testicles tissue. **(d)** Representative HE images of heart, liver, spleen, lung and kidney tissue. Scale bars: 50 μm. Data are shown as the mean ± SEM. Behavioral pain assessments of individual timepoints were performed utilizing one-way ANOVA followed by Tukey’s *post-hoc* test. ###*p* < 0.001 *vs* the Control group. ***p* < 0.01 and ****p* < 0.001 *vs* the EAP group. (n = 3 per group).

Supplementary Figure 2 Flow cytometry analysis revealed that TENS stimulation facilitated the polarization of prostate-infiltrating macrophages toward the M2 phenotype (CD86^−^CD206^+^) in rats with EAP. Data are shown as the mean ± SEM. One-way ANOVA followed by Tukey’s *post-hoc* test. **p* < 0.05, ***p* < 0.01 and ****p* < 0.001 *vs* the EAP group. (n = 3 per group).

Supplementary Figure 3 MS results. **(a)** Results of repetitive CV analysis. **(b)** KEGG pathway enrichment analysis of differentially expressed proteins between the LPS and ES groups (*p* < 0.05). **(c)** Heat map of differentially clustered complement and coagulation cascades, fat digestion and absorption, and MAPK signaling pathway. (n = 3 per group).

Supplementary Figure 4 Cell viability after ES and agonists tested by CCK8 assay. Data are shown as the mean ± SEM. One-way ANOVA followed by Tukey’s *post-hoc* test. ##*p* < 0.01 *vs* the LPS group. (n = 3 per group).

Supplementary Figure 5 Electrophysiological phenotype and polarization signaling pathway in macrophages with LPS and ES intervention. Quantitative analysis of **(a)** cellular membrane potential and intracellular Ca^2+^ concentration **(b)** in macrophages measured by flow cytometry. MFI: Mean Fluorescence Intensity. **(c)** Representative WB images of Kir2.1 and TRPV2 expression. **(d)** Quantitative analyses of Kir2.1 and TRPV2 markers expression. **(e)** Representative WB images of NF-κB/STAT1/STAT6 signaling pathway expression. **(f)** Quantitative analyses of NF-κB/STAT1/STAT6 signaling pathway expression. Data are shown as the mean ± SEM. One-way ANOVA followed by Tukey’s *post-hoc* test. ##*p* < 0.01 and ###*p* < 0.001 *vs* the LPS group. **p* < 0.05, ***p* < 0.01 and ****p* < 0.001 *vs* the ES group. (n = 3 per group).
