# Supplementary material for: TENS alleviates CP/CPPS-related inflammation and pain by modulating Kir2.1-dependent macrophage polarization
Source: Front Immunol. 2026 Jan 12;16:1683500. doi: 10.3389/fimmu.2025.1683500 (PMC12857300; doi:10.3389/fimmu.2025.1683500)
Supplement: Supplementary file 1 [file DataSheet1.pdf]

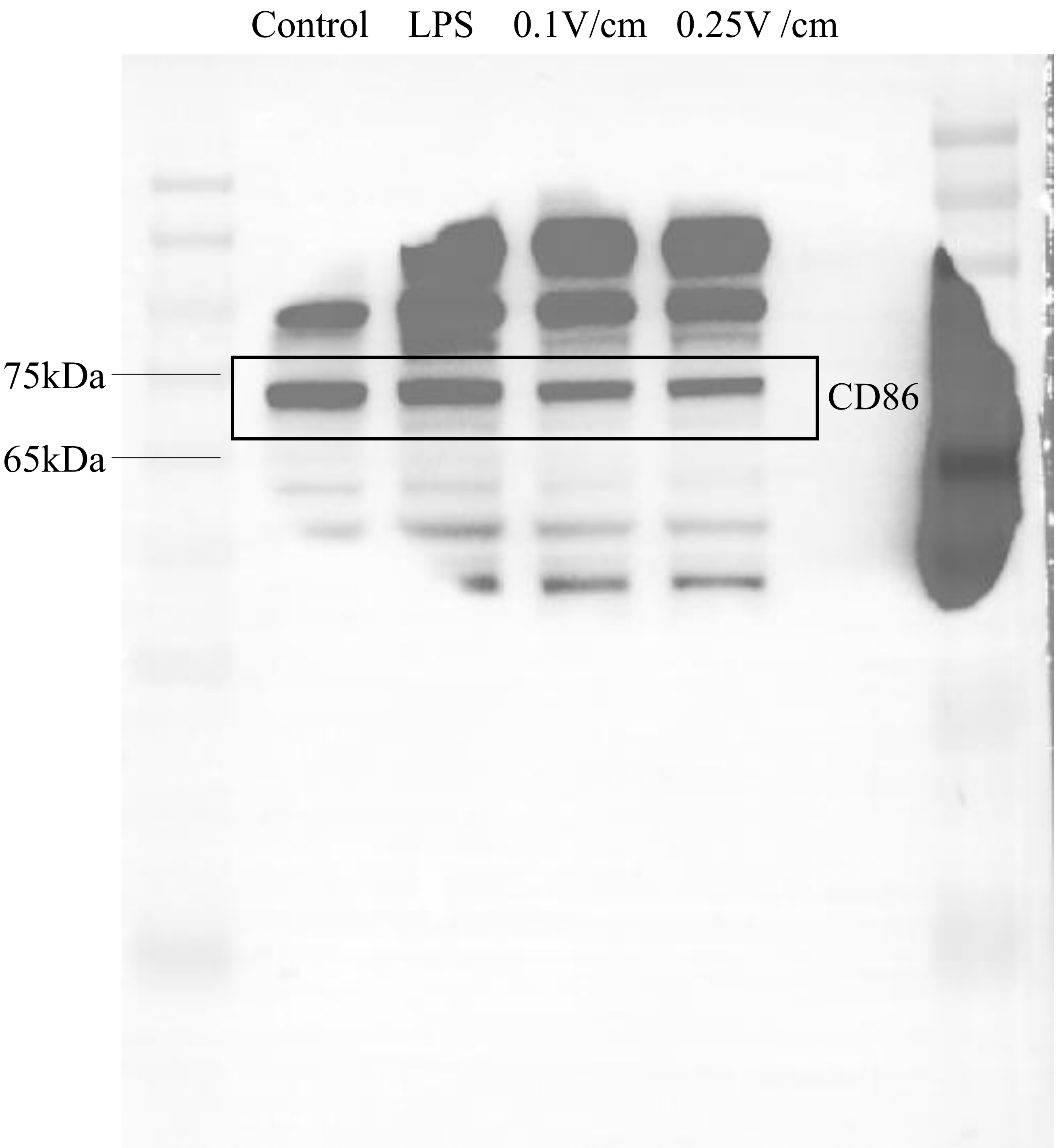
Control
LPS
0.1V/cm
0.25V /cm
75kDa
65kDa
CD86

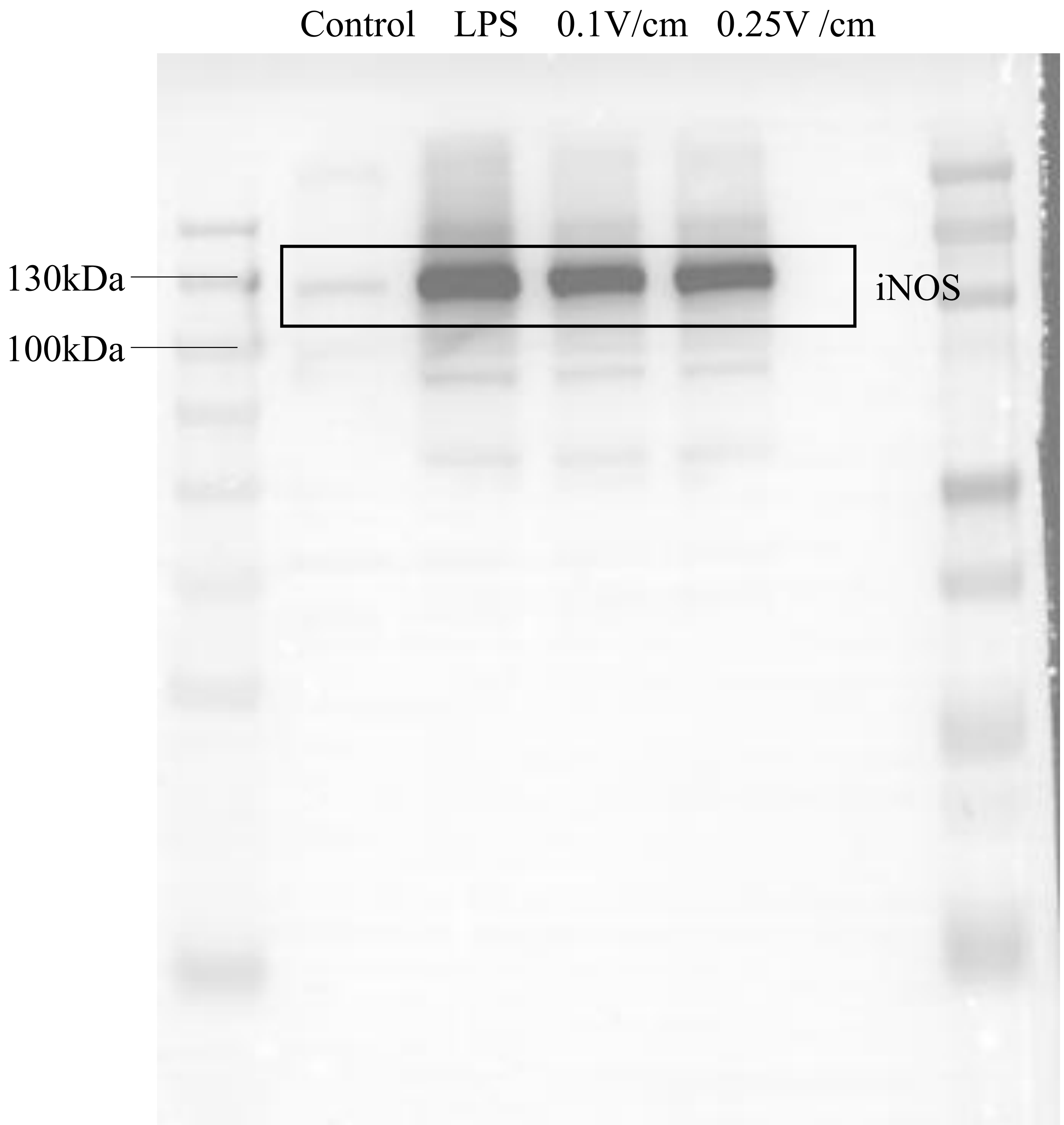
Control
LPS
0.1V/cm
0.25V /cm
130kDa
100kDa
iNOS

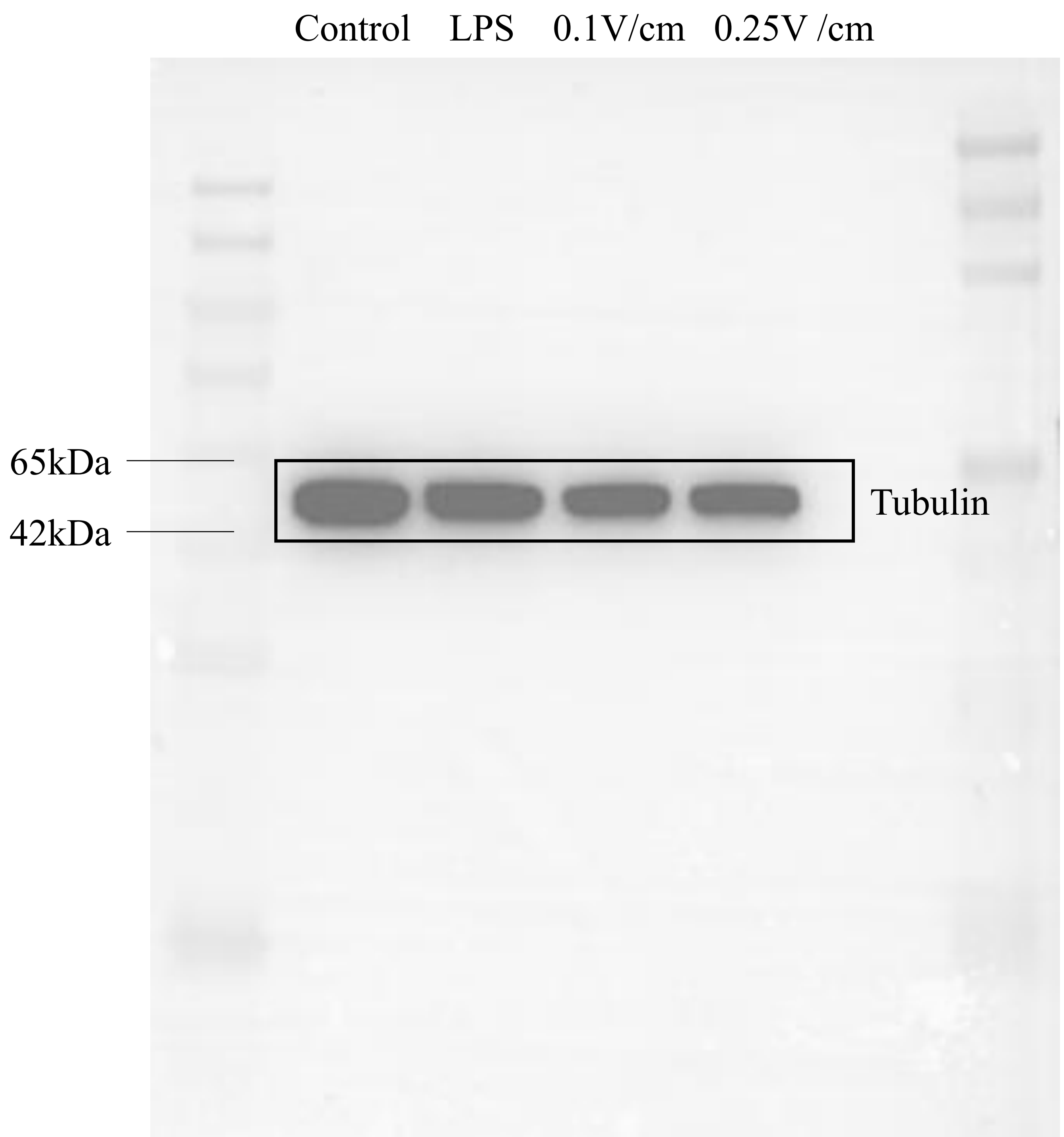
Control
LPS
0.1V/cm
0.25V /cm
65kDa
42kDa
Tubulin

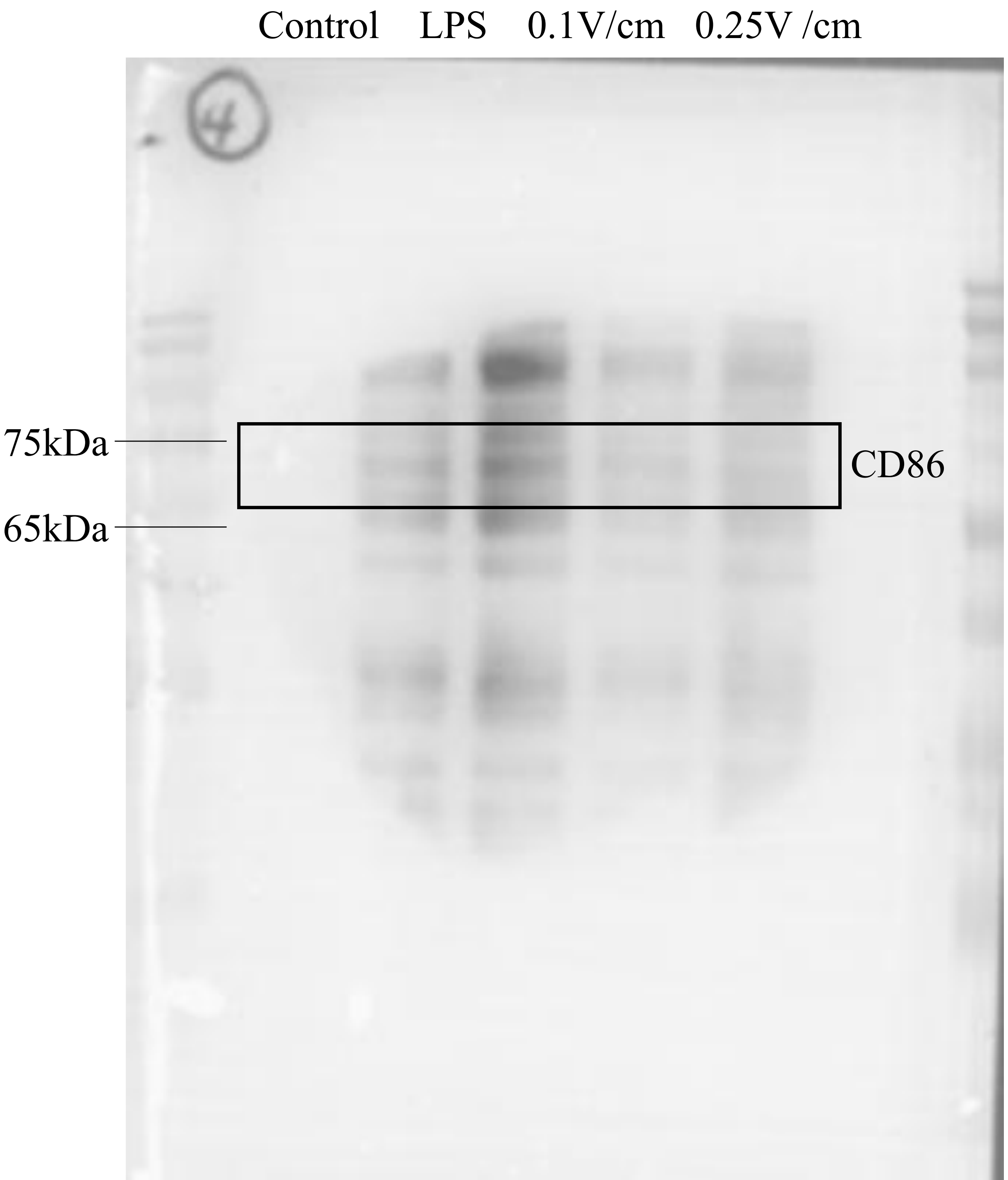
Control LPS 0.1V/cm 0.25V /cm
75kDa
65kDa
CD86

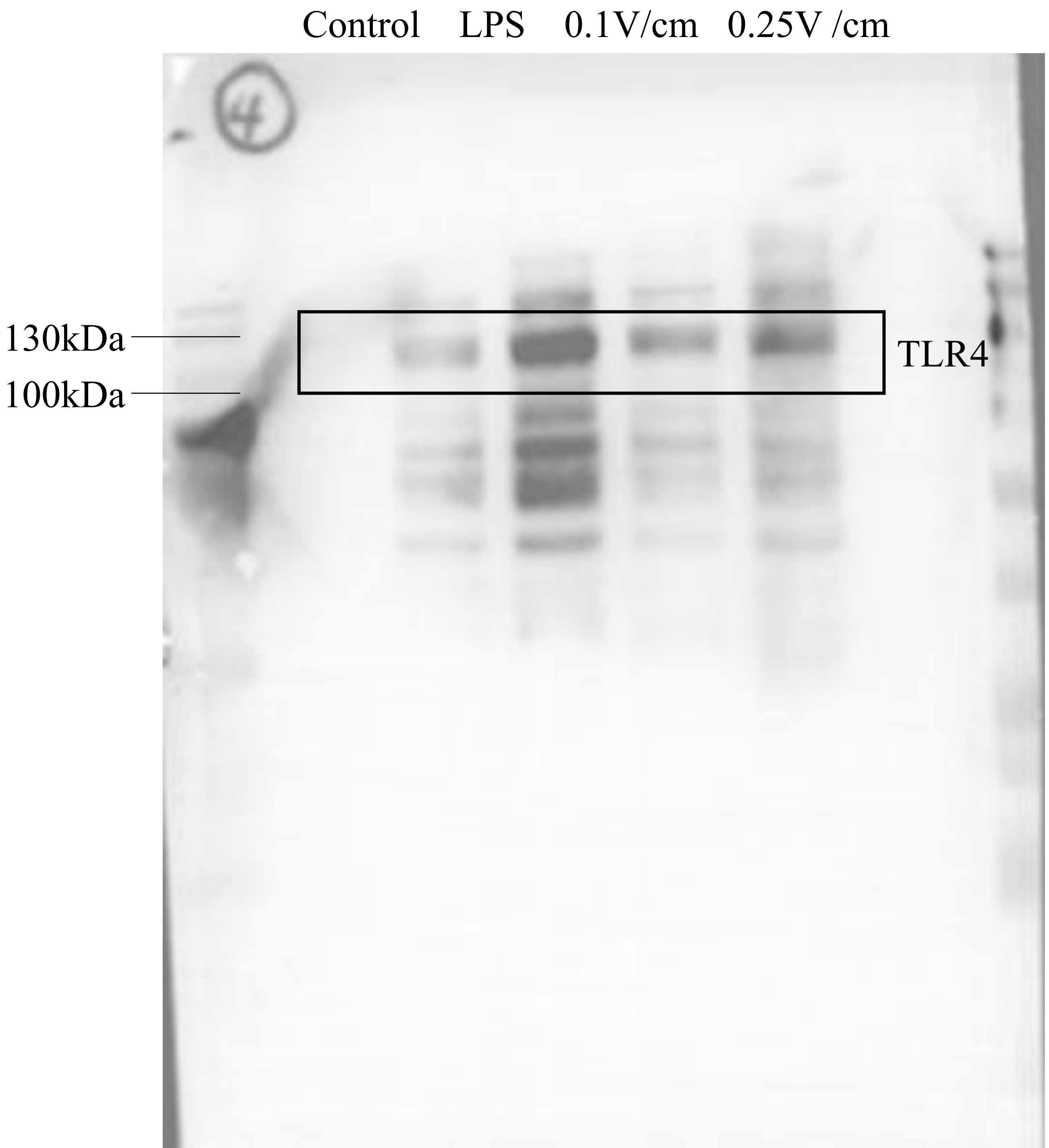
Control
LPS
0.1V/cm
0.25V /cm
130kDa
100kDa
TLR4

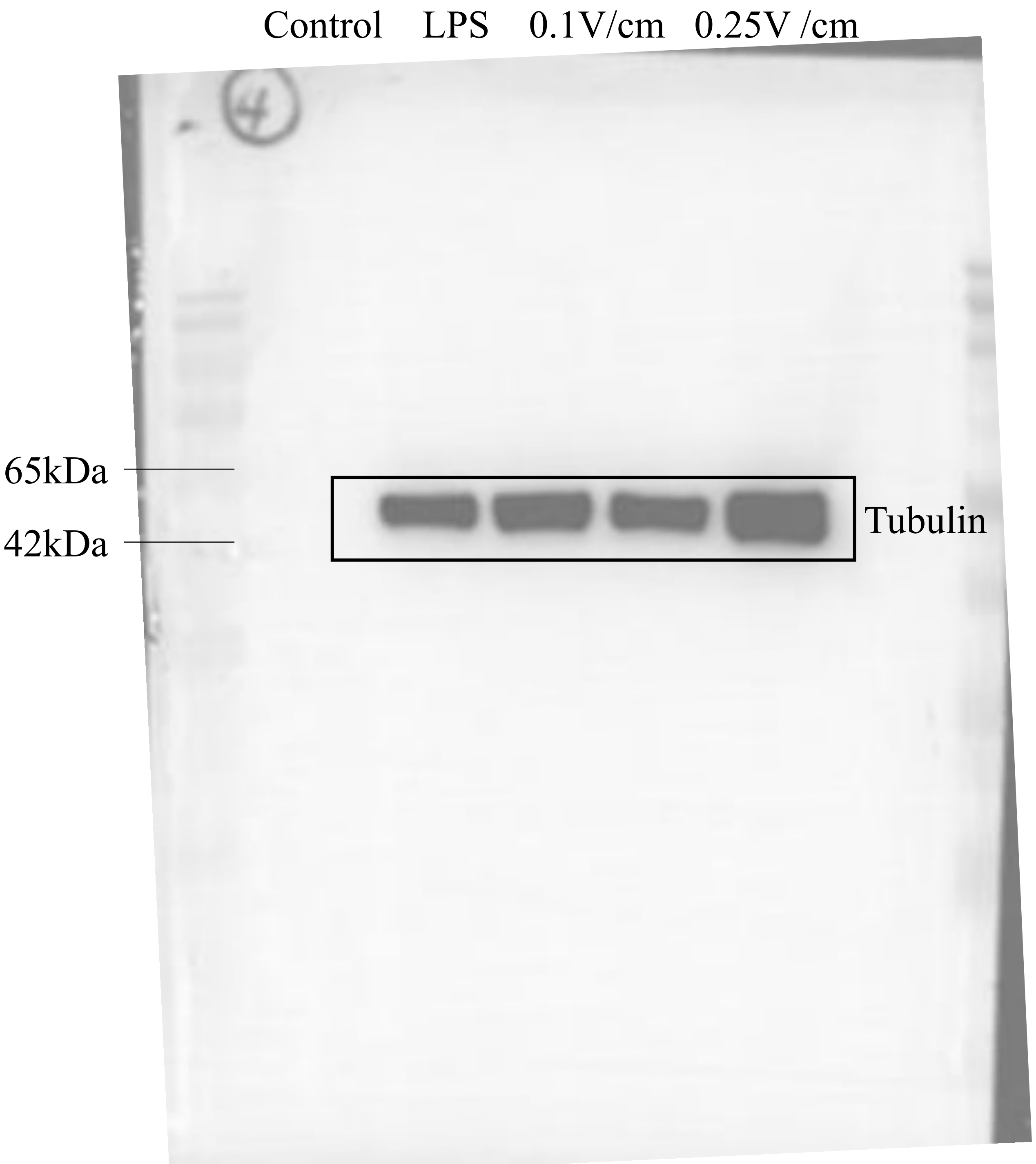
Control LPS 0.1V/cm 0.25V /cm
65kDa
42kDa
Tubulin

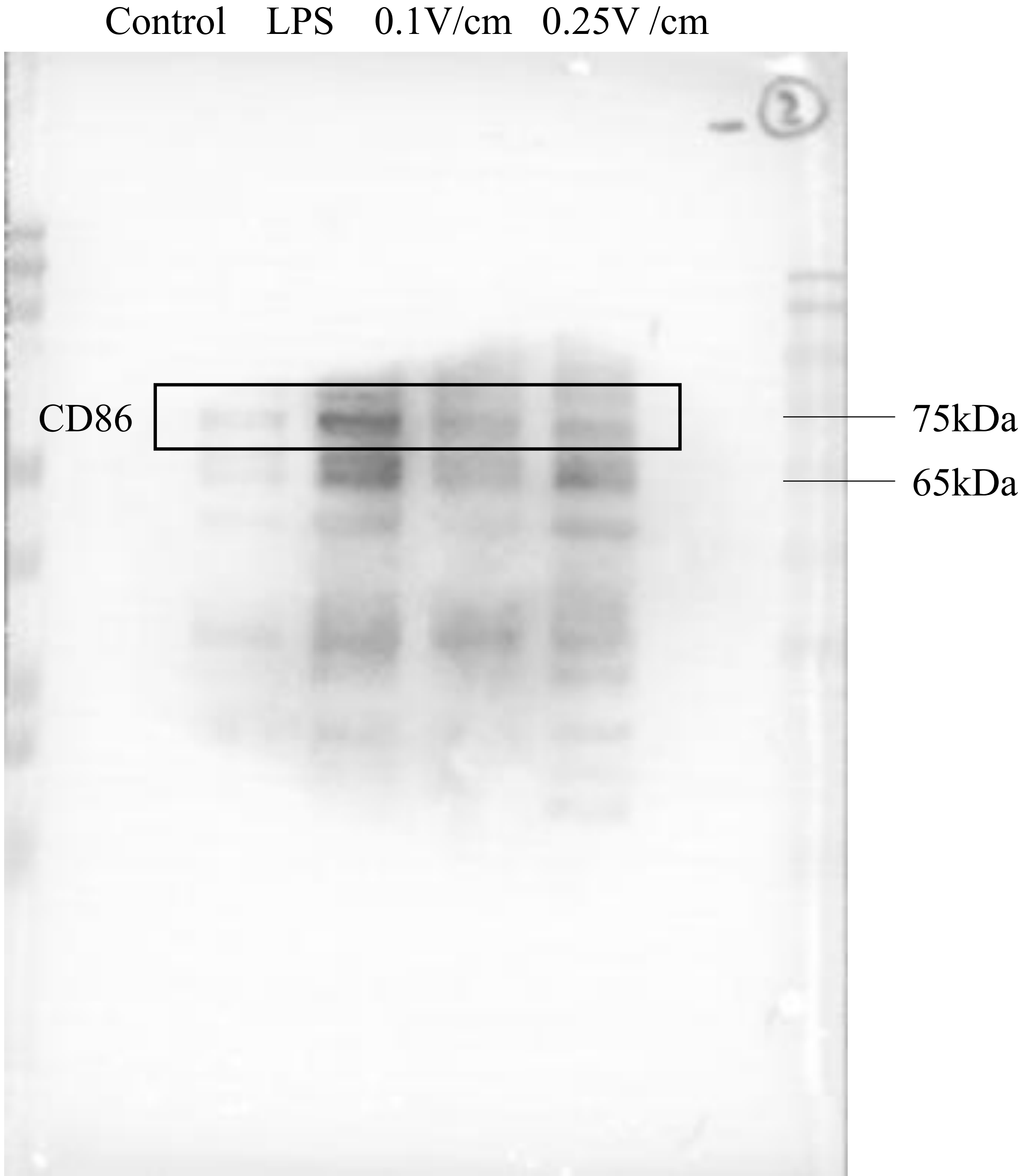
Control
LPS
0.1V/cm
0.25V /cm
CD86
75kDa
65kDa

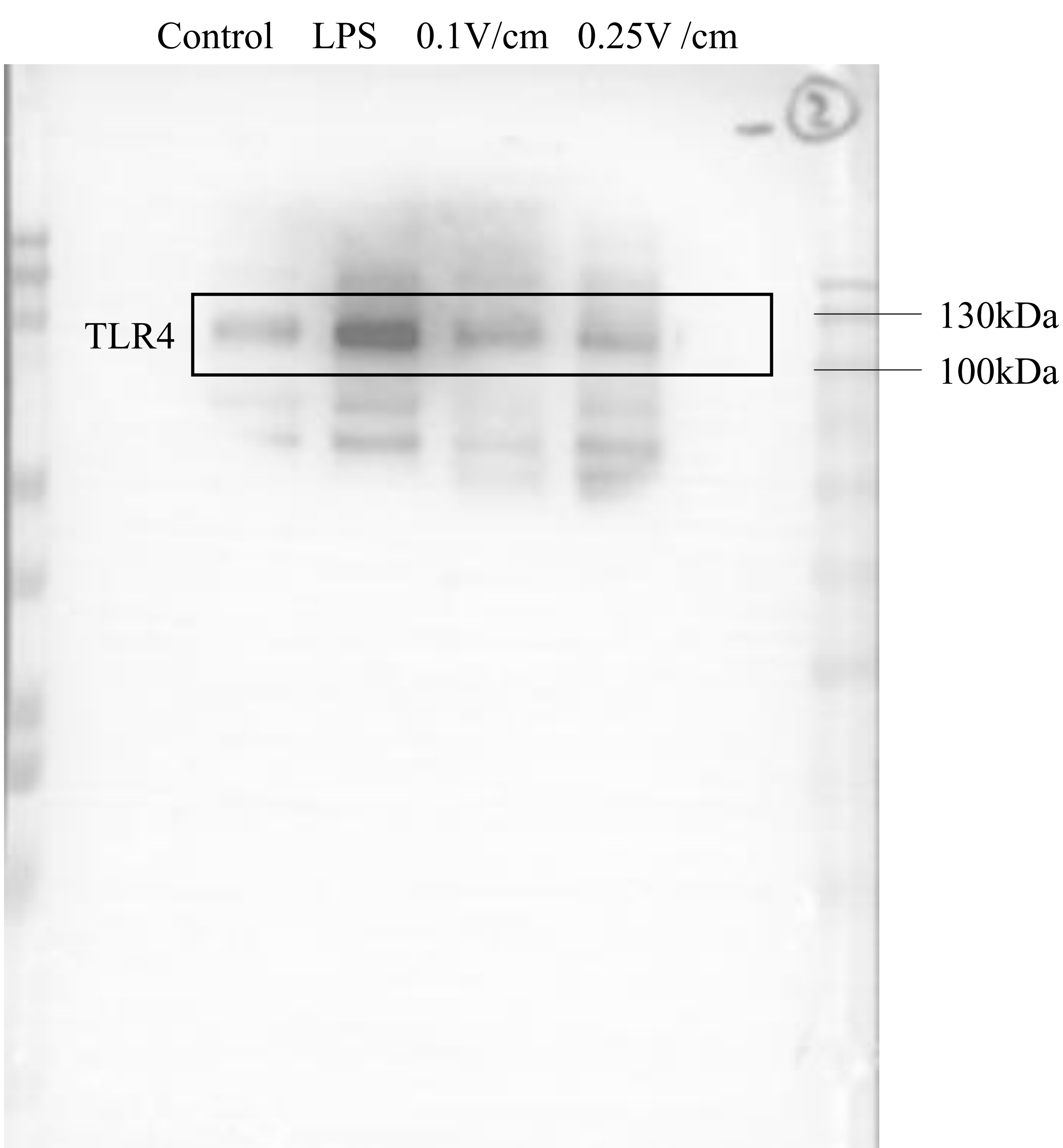
Control
LPS
0.1V/cm
0.25V /cm
TLR4
130kDa
100kDa

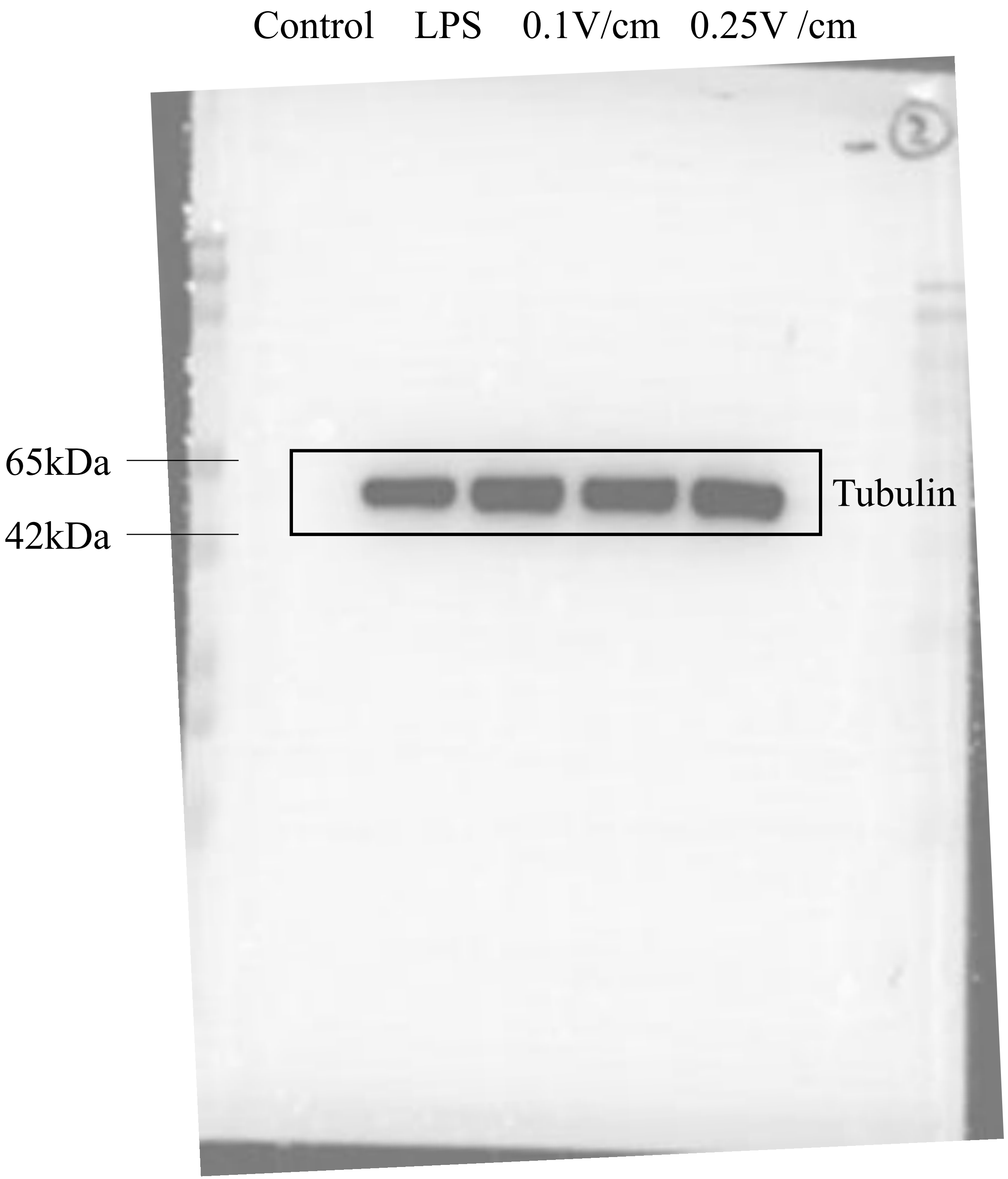
Control LPS 0.1V/cm 0.25V /cm
65kDa
42kDa
Tubulin

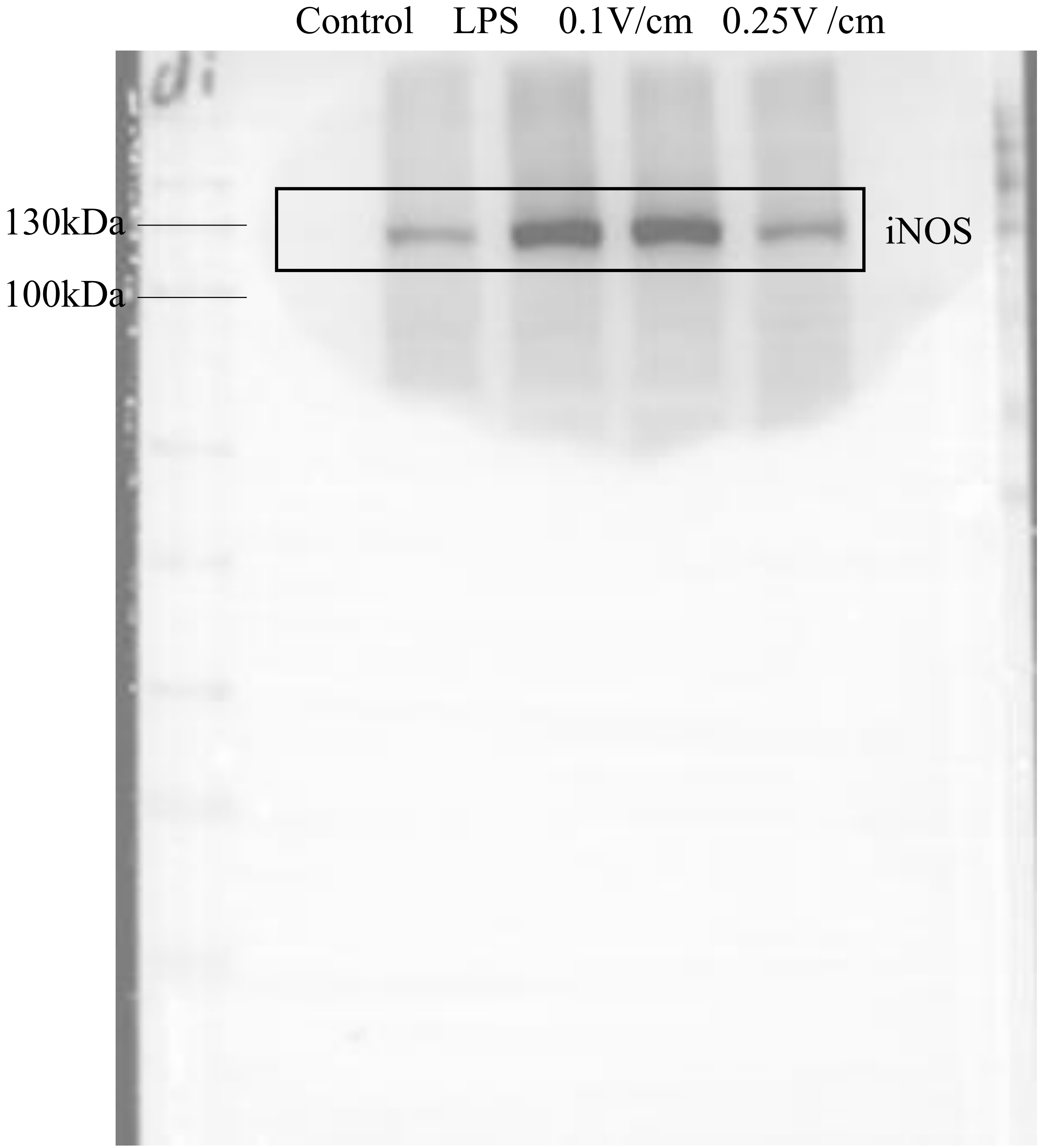
Control
LPS
0.1V/cm
0.25V /cm
130kDa
100kDa
iNOS

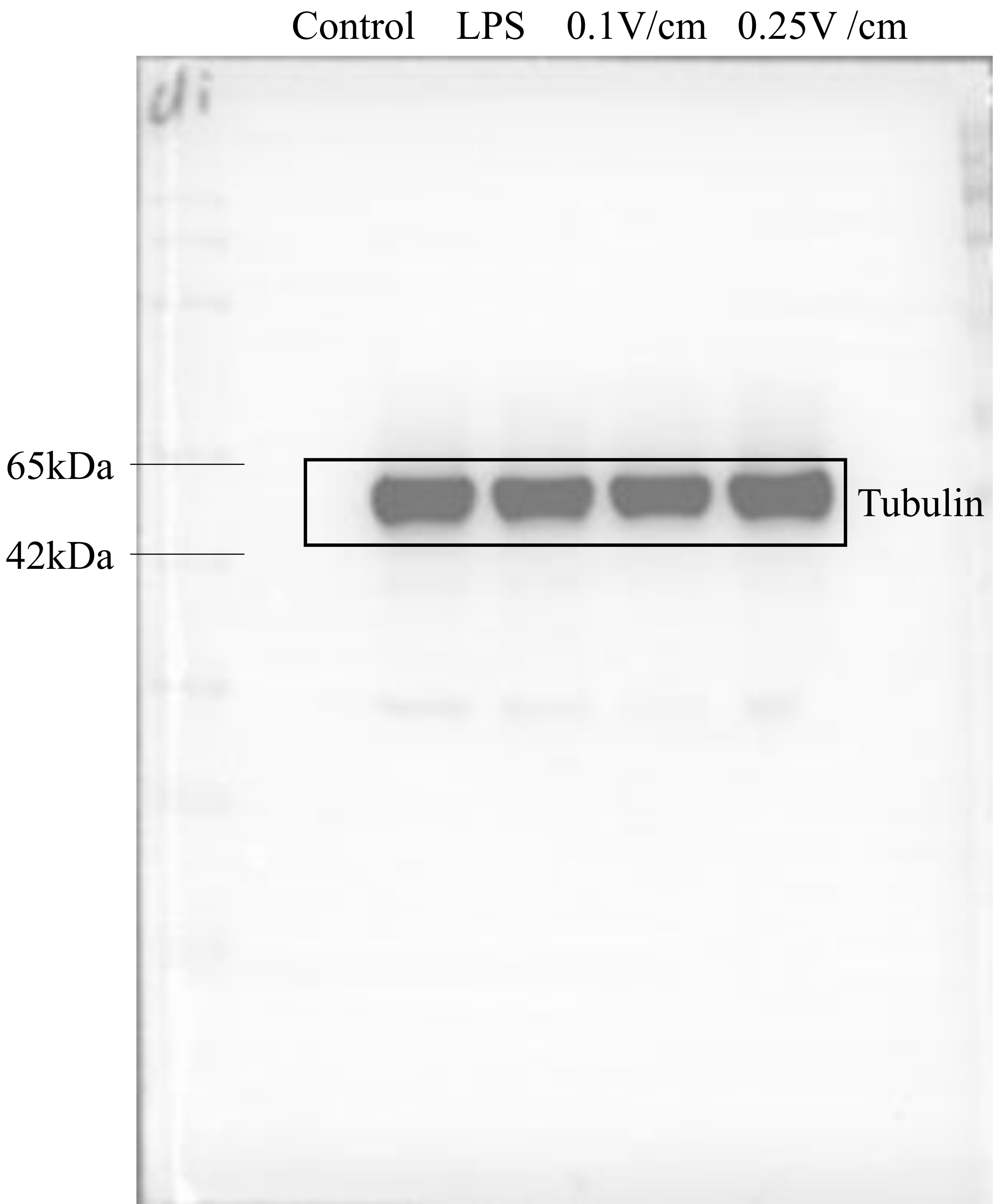
Control
LPS
0.1V/cm
0.25V /cm
65kDa
42kDa
Tubulin

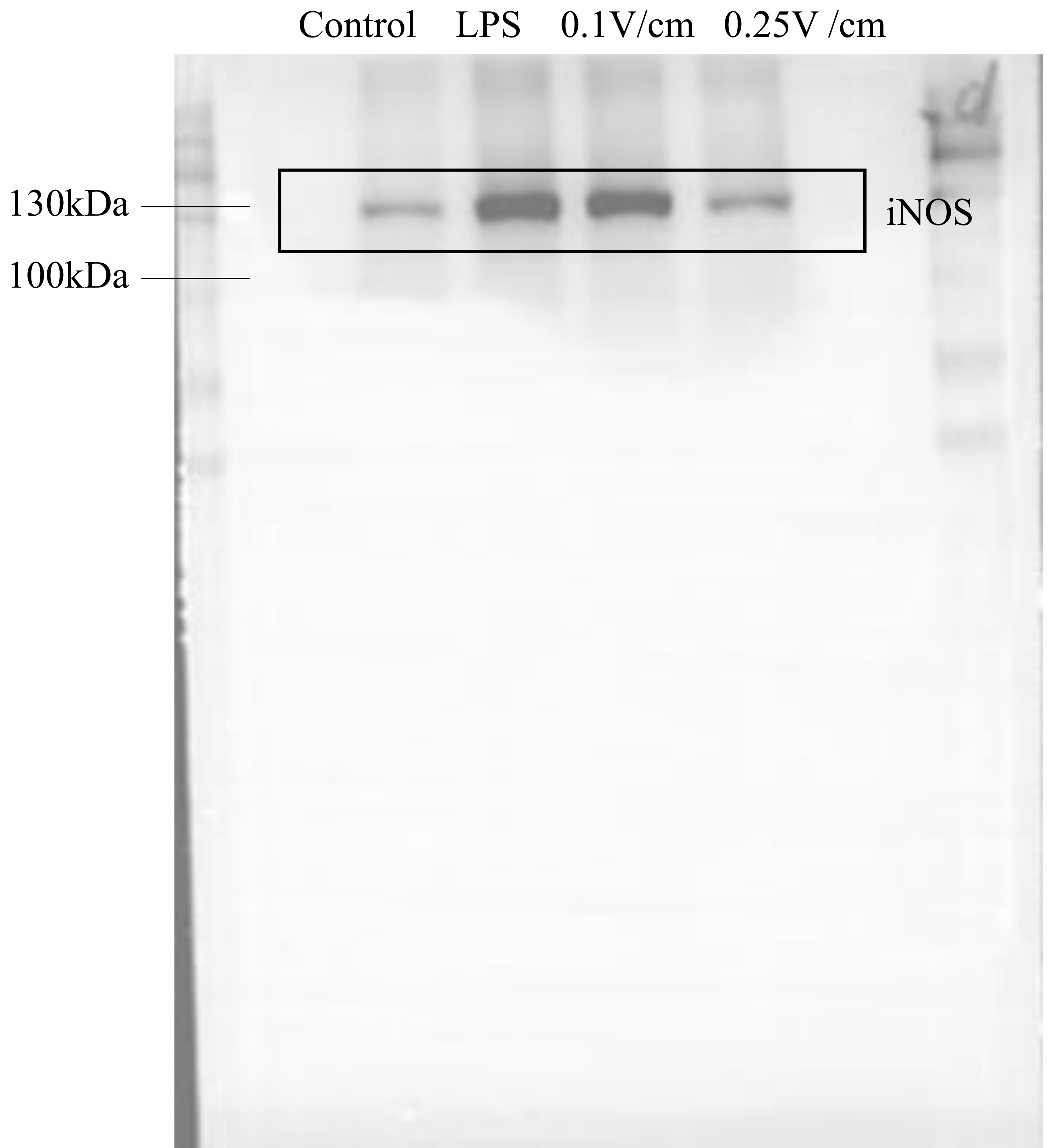

Control
LPS
0.1V/cm
0.25V /cm
130kDa
100kDa
iNOS

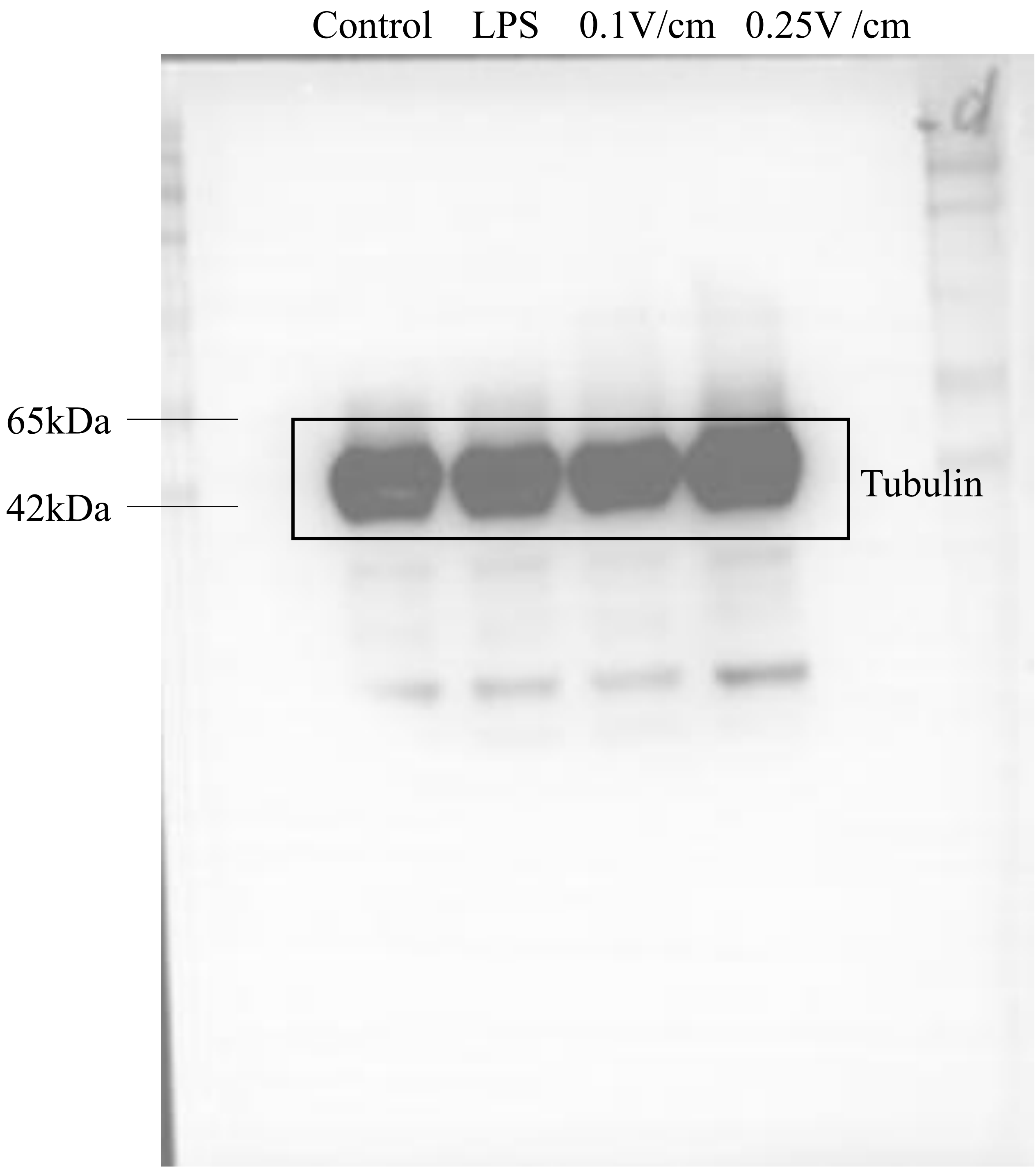
Control
LPS
0.1V/cm
0.25V /cm
65kDa
42kDa
Tubulin

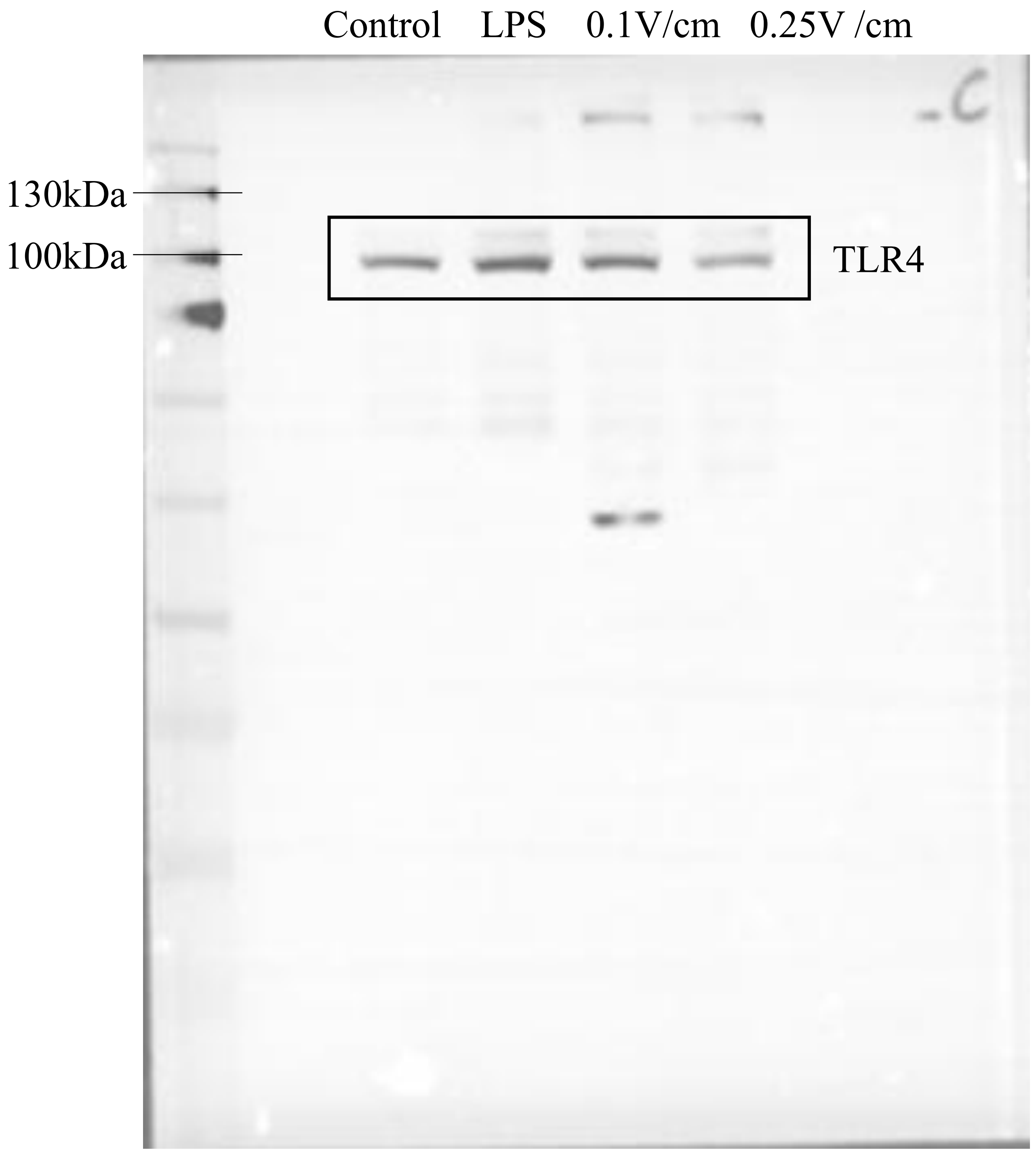

Control
LPS
0.1V/cm
0.25V /cm
130kDa
100kDa
TLR4

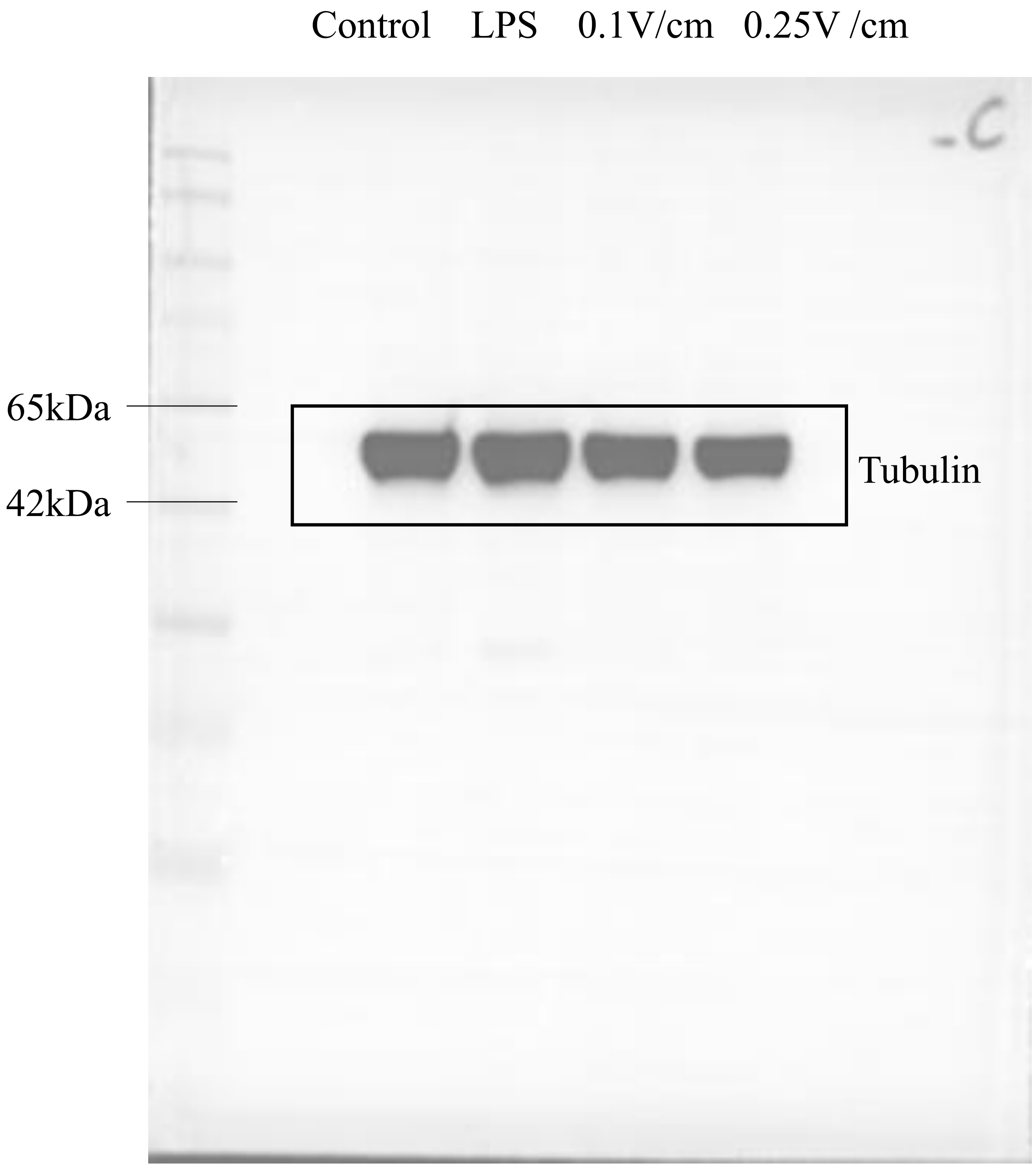
Control
LPS
0.1V/cm
0.25V /cm
65kDa
42kDa
Tubulin

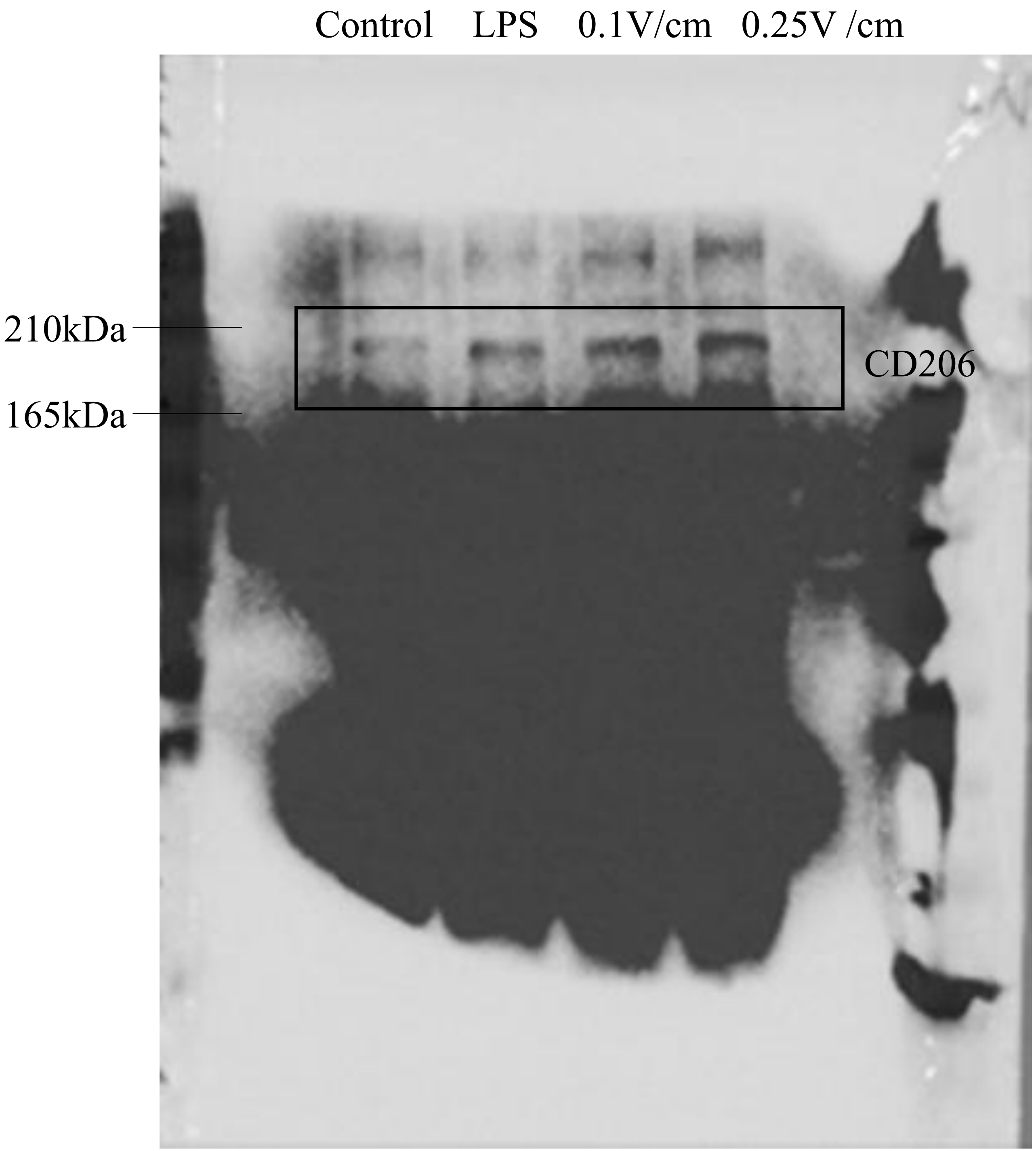
Control LPS 0.1V/cm 0.25V /cm
210kDa
165kDa
CD206

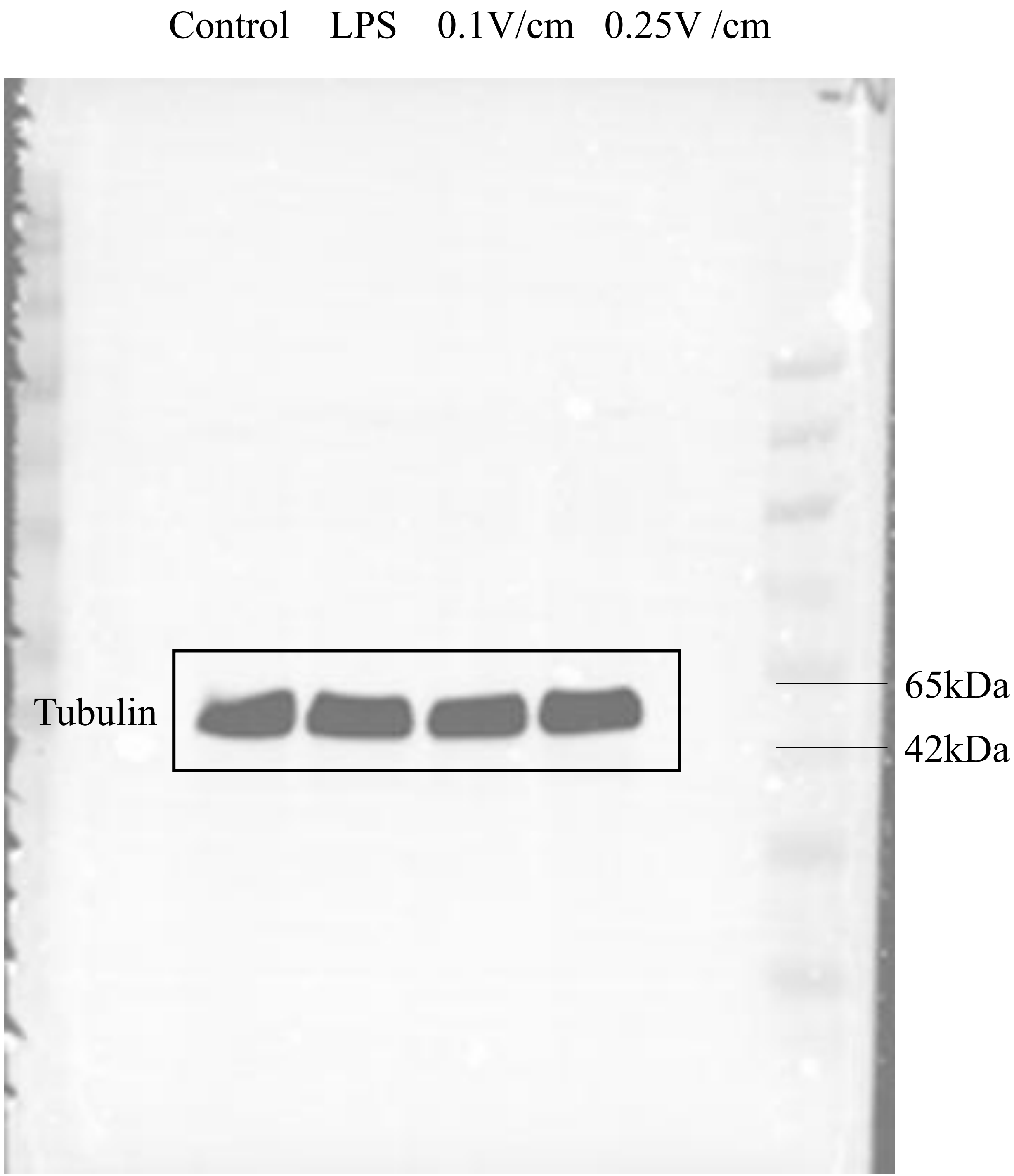

Control
LPS
0.1V/cm
0.25V /cm
Tubulin
65kDa
42kDa

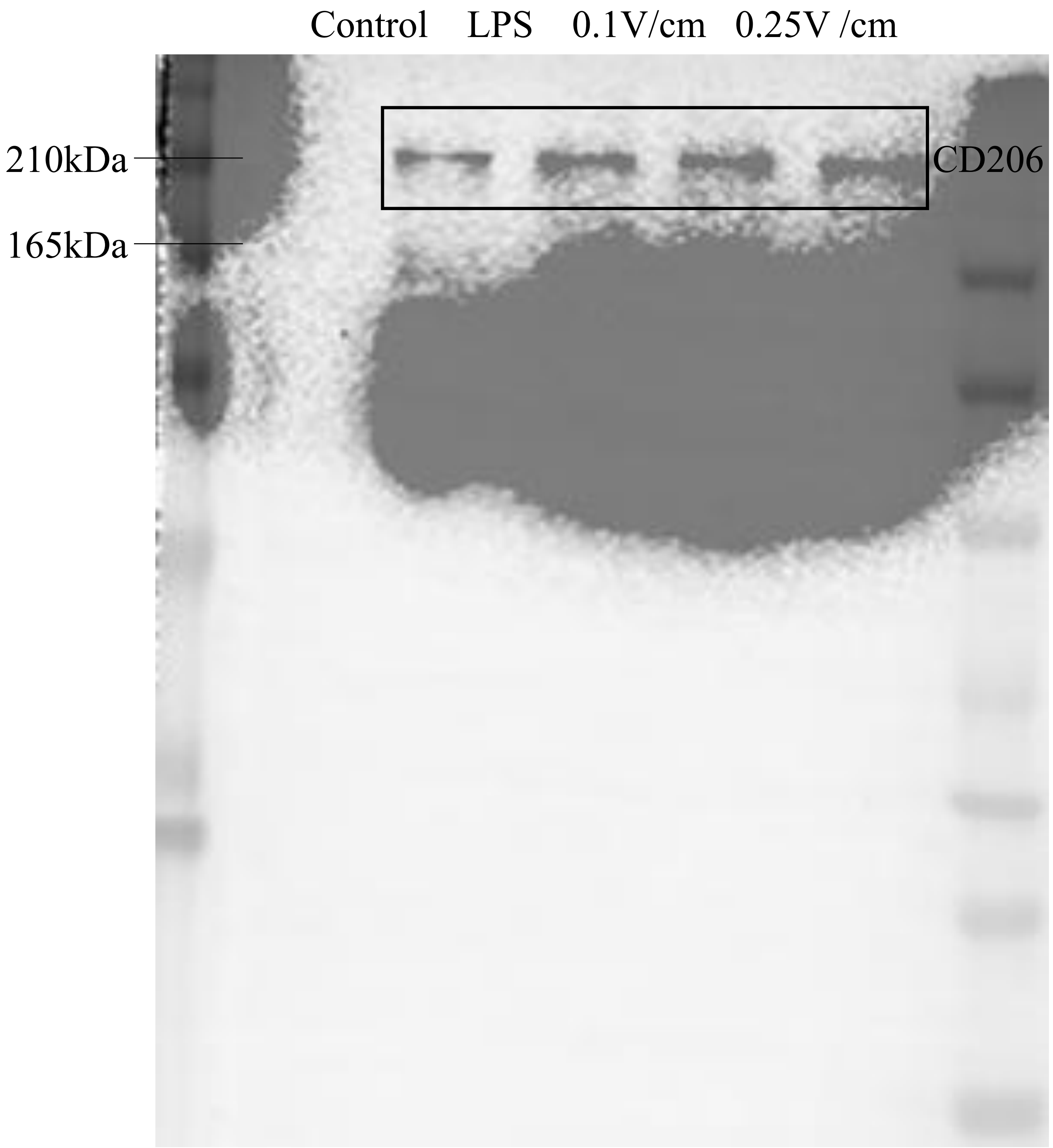
Control
LPS
0.1V/cm
0.25V /cm
210kDa
165kDa
CD206

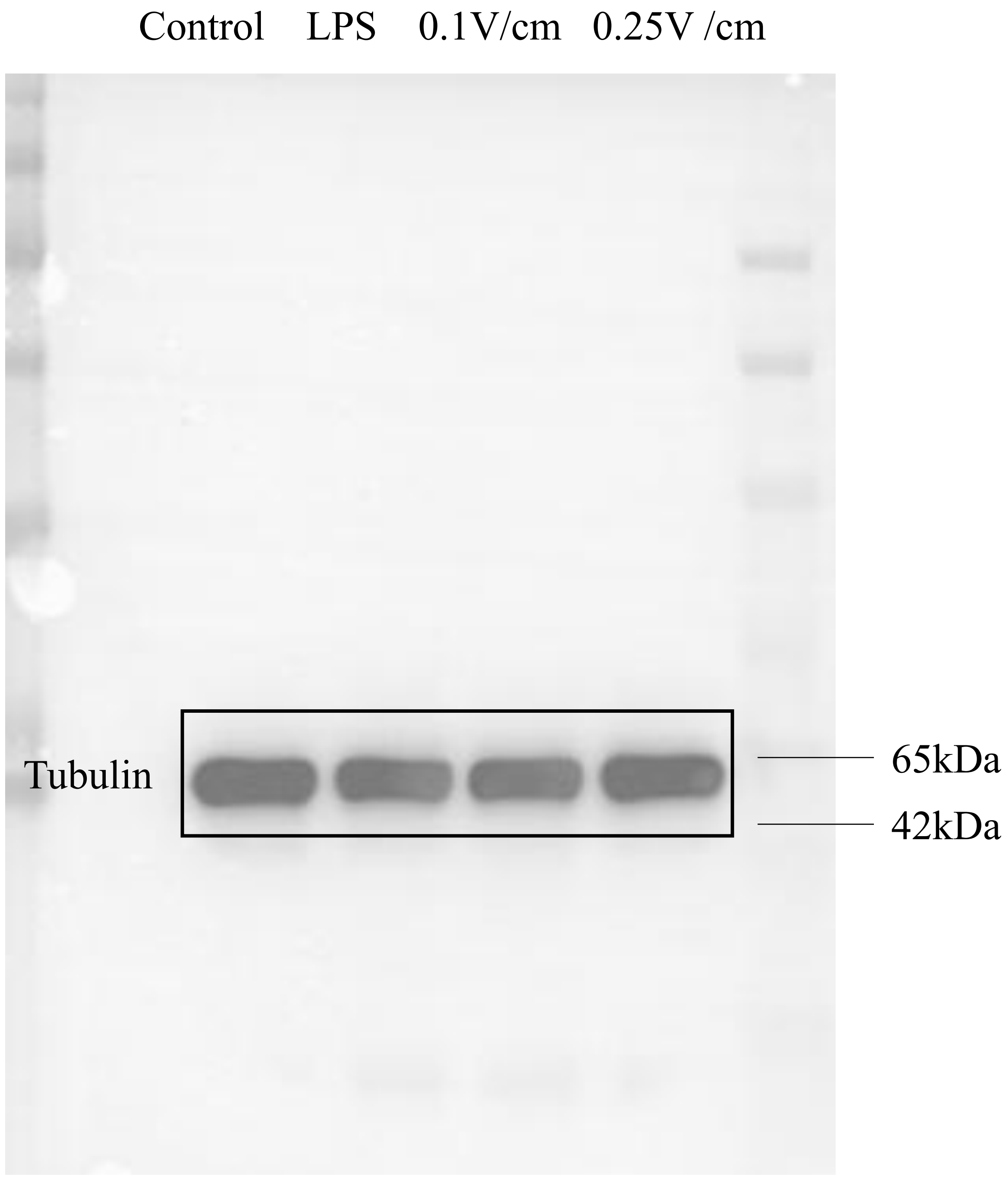
Control
LPS
0.1V/cm
0.25V /cm
Tubulin
65kDa
42kDa

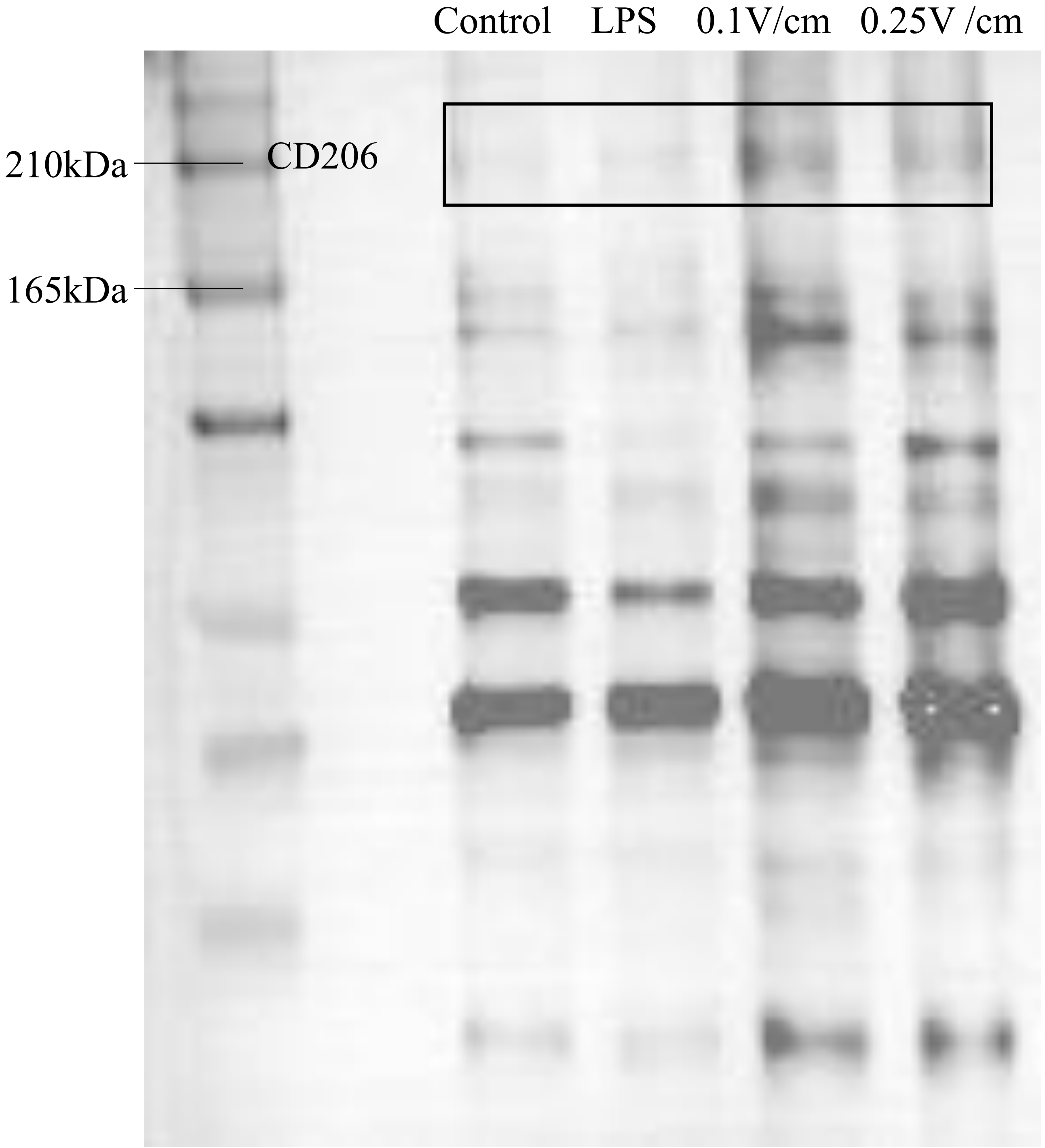
Control
LPS
0.1V/cm
0.25V /cm
210kDa
CD206
165kDa

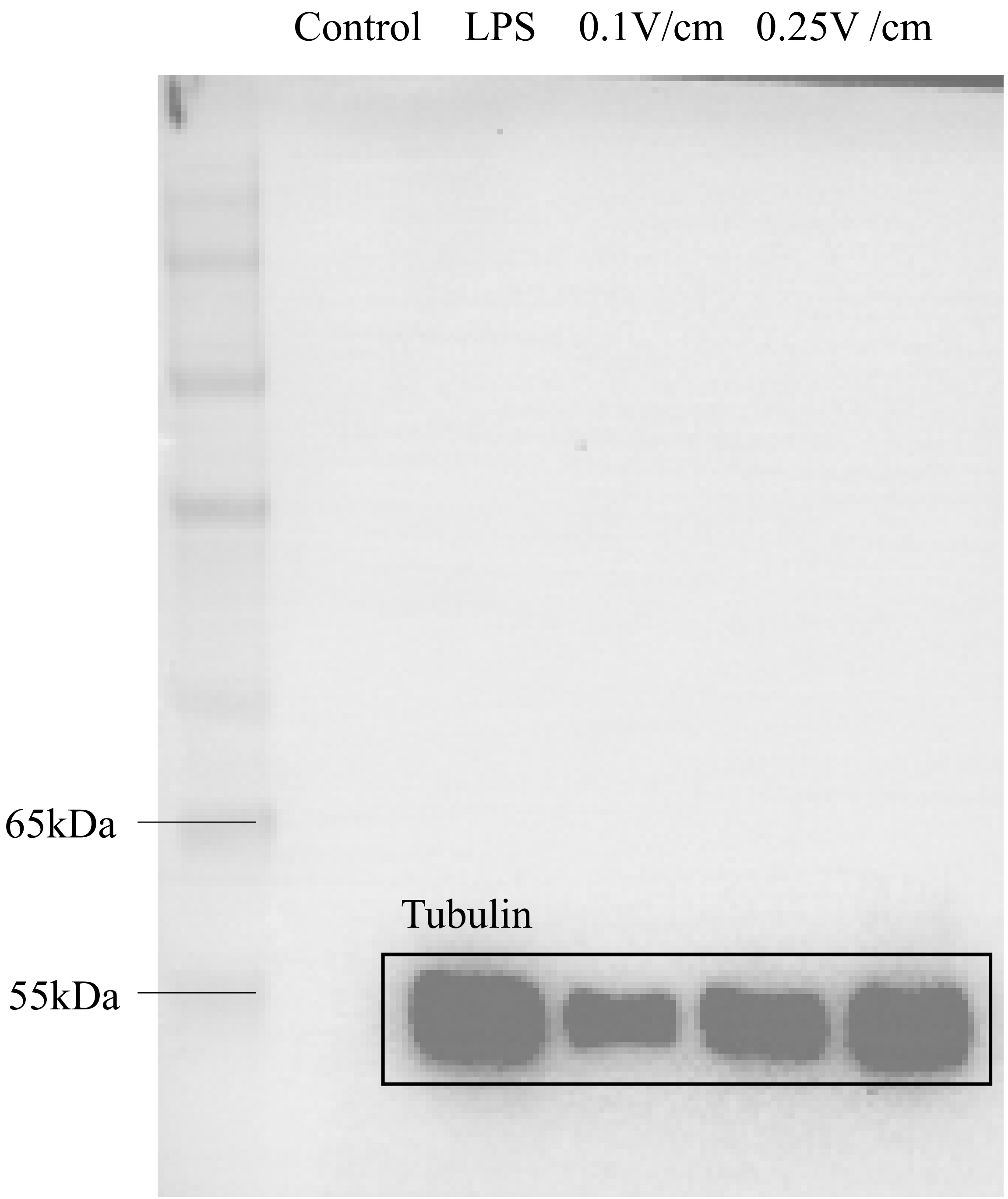
Control
LPS
0.1V/cm
0.25V /cm
65kDa
55kDa
Tubulin

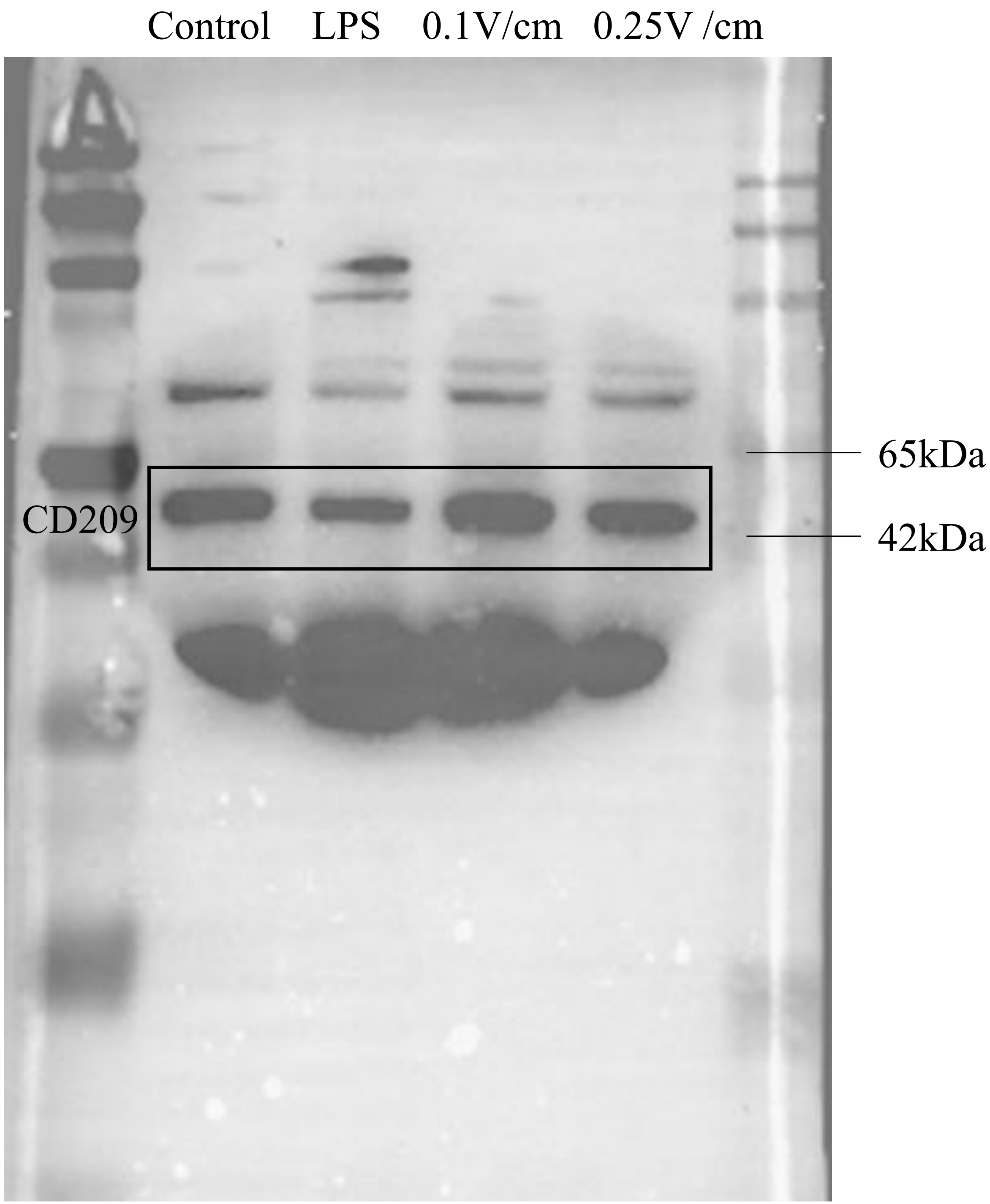
Control
LPS
0.1V/cm
0.25V /cm
CD209
65kDa
42kDa

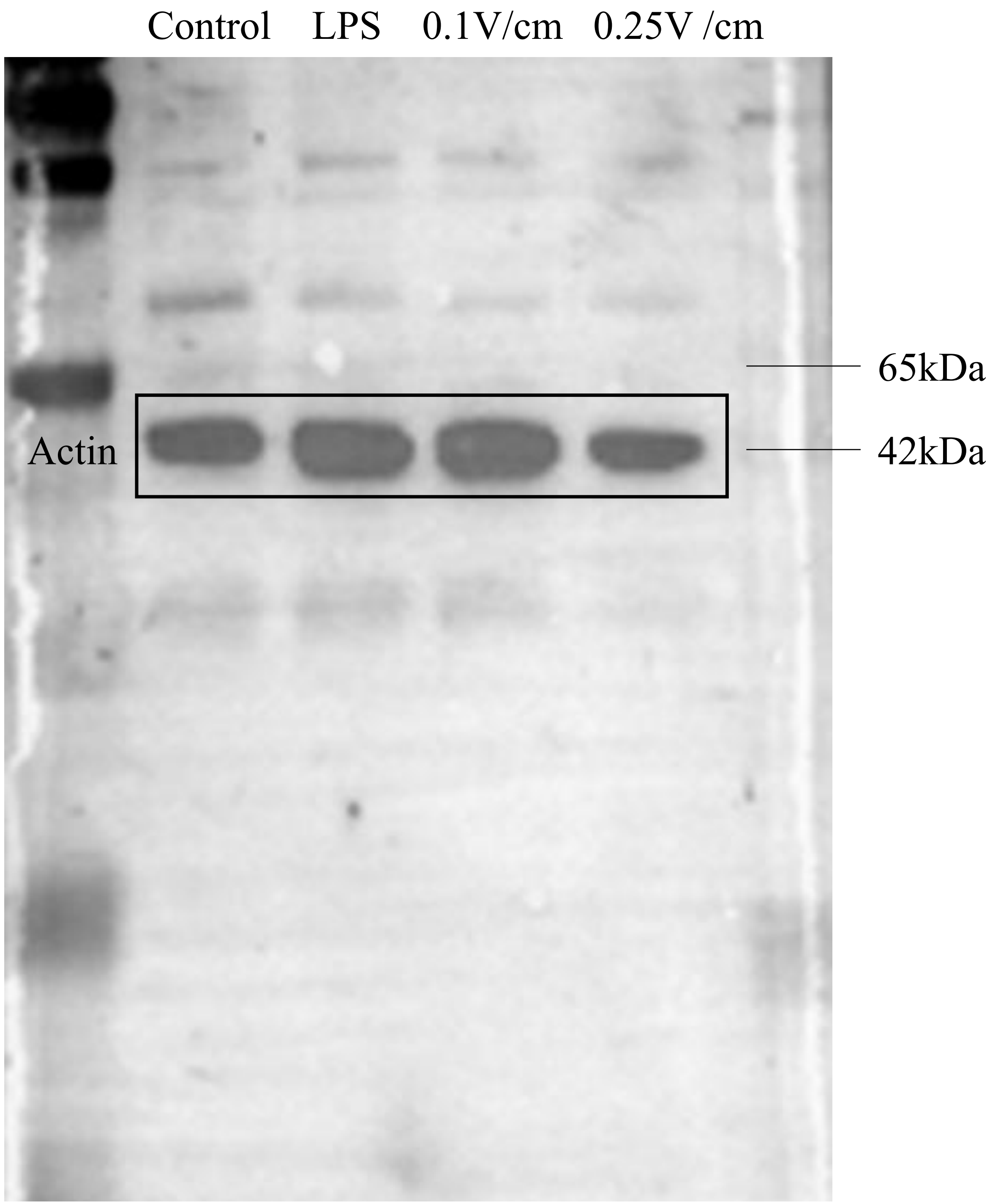
Control
LPS
0.1V/cm
0.25V /cm
Actin
65kDa
42kDa

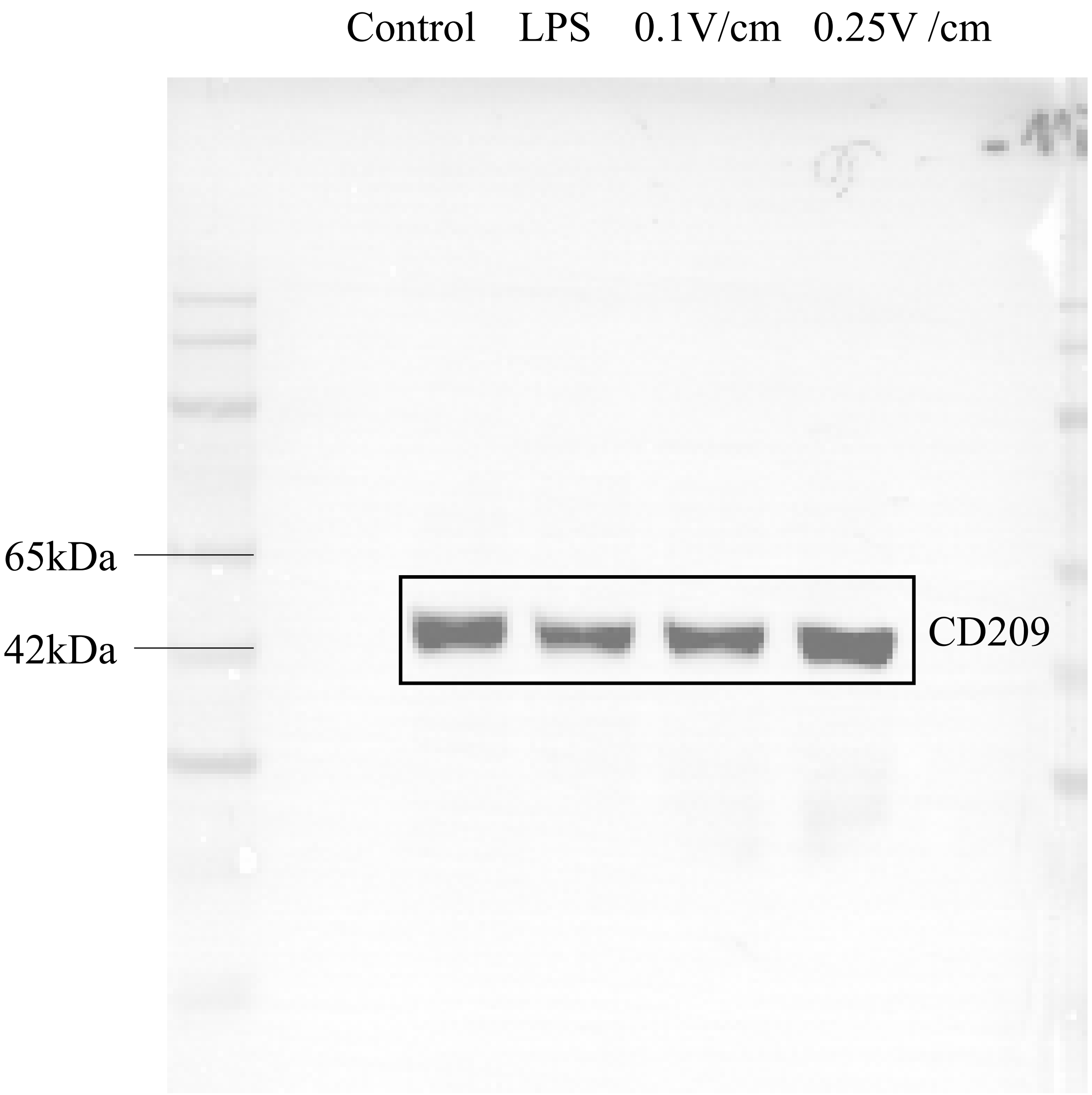
Control
LPS
0.1V/cm
0.25V /cm
65kDa
42kDa
CD209

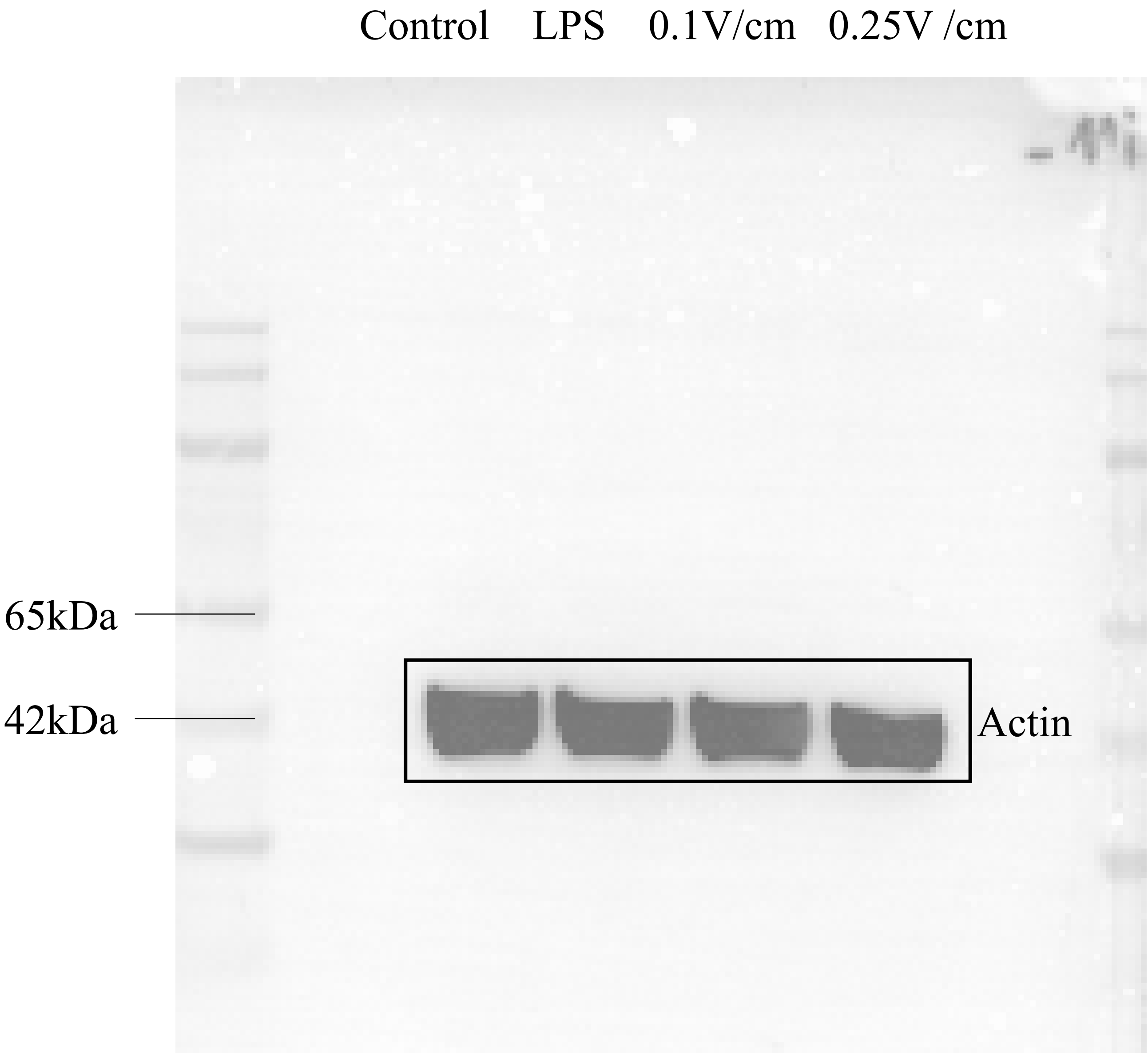
Control
LPS
0.1V/cm
0.25V /cm
65kDa
42kDa
Actin

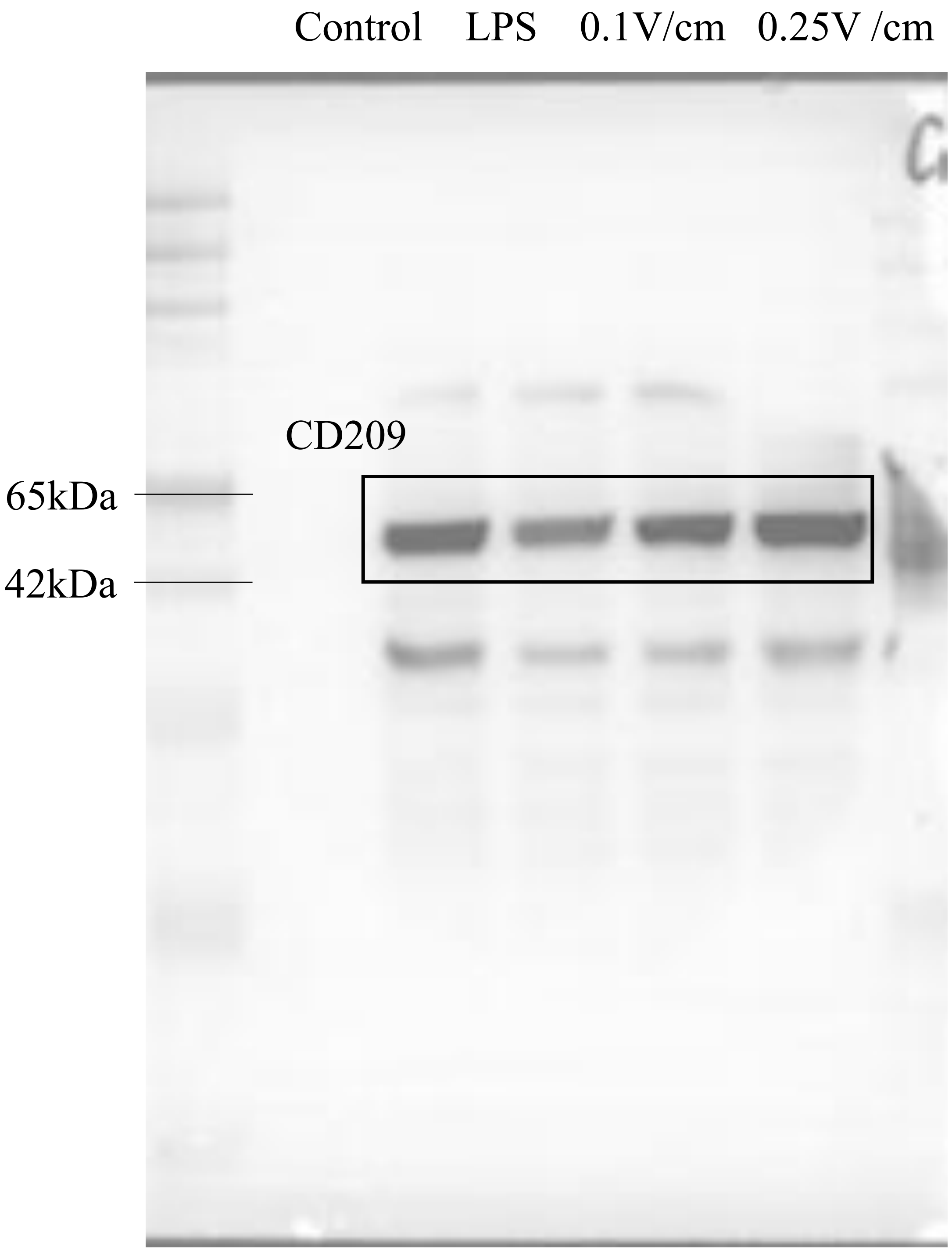
Control
LPS
0.1V/cm
0.25V /cm
CD209
65kDa
42kDa

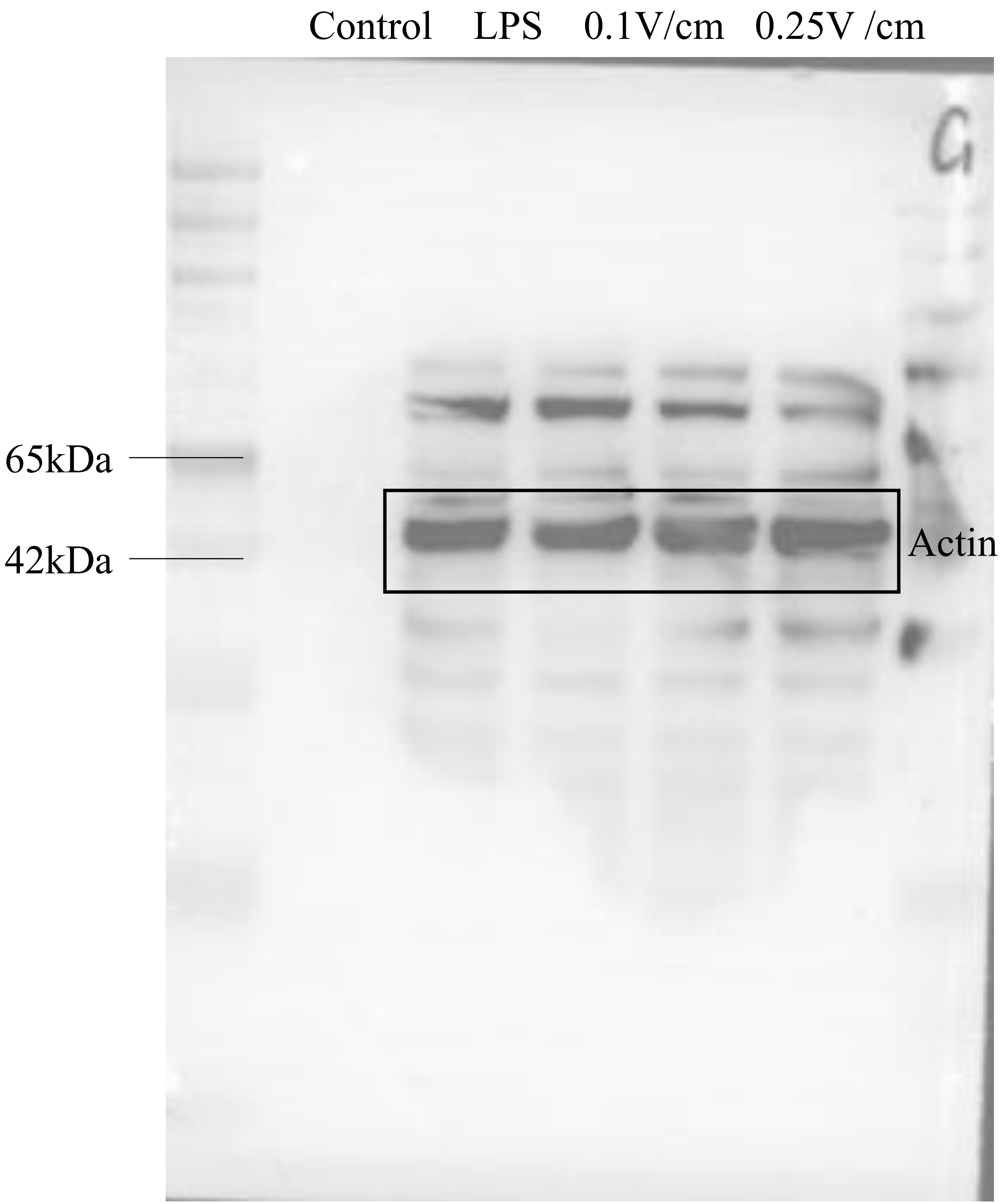
Control
LPS
0.1V/cm
0.25V /cm
65kDa
42kDa
Actin

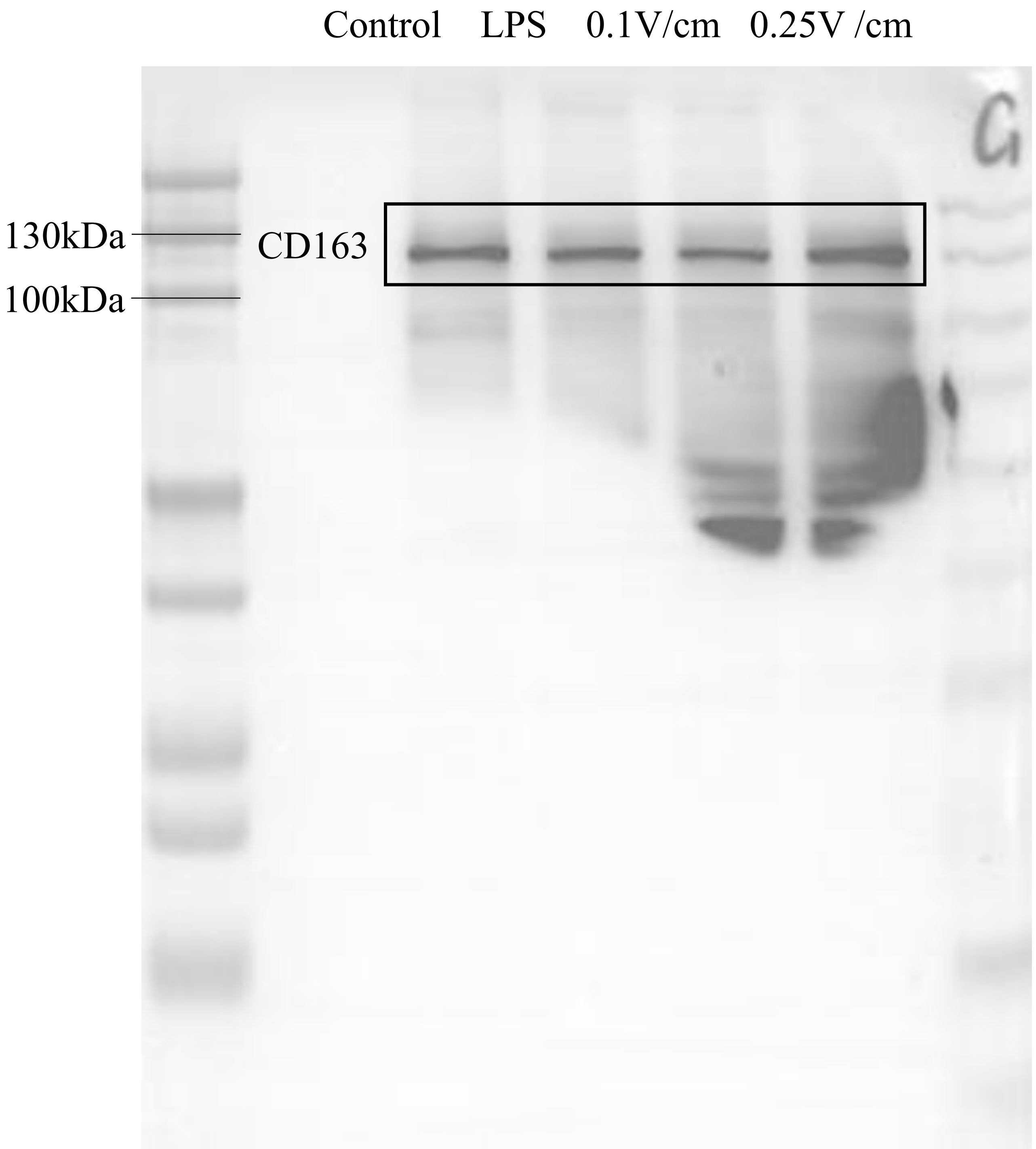
Control
LPS
0.1V/cm
0.25V /cm
130kDa
100kDa
CD163

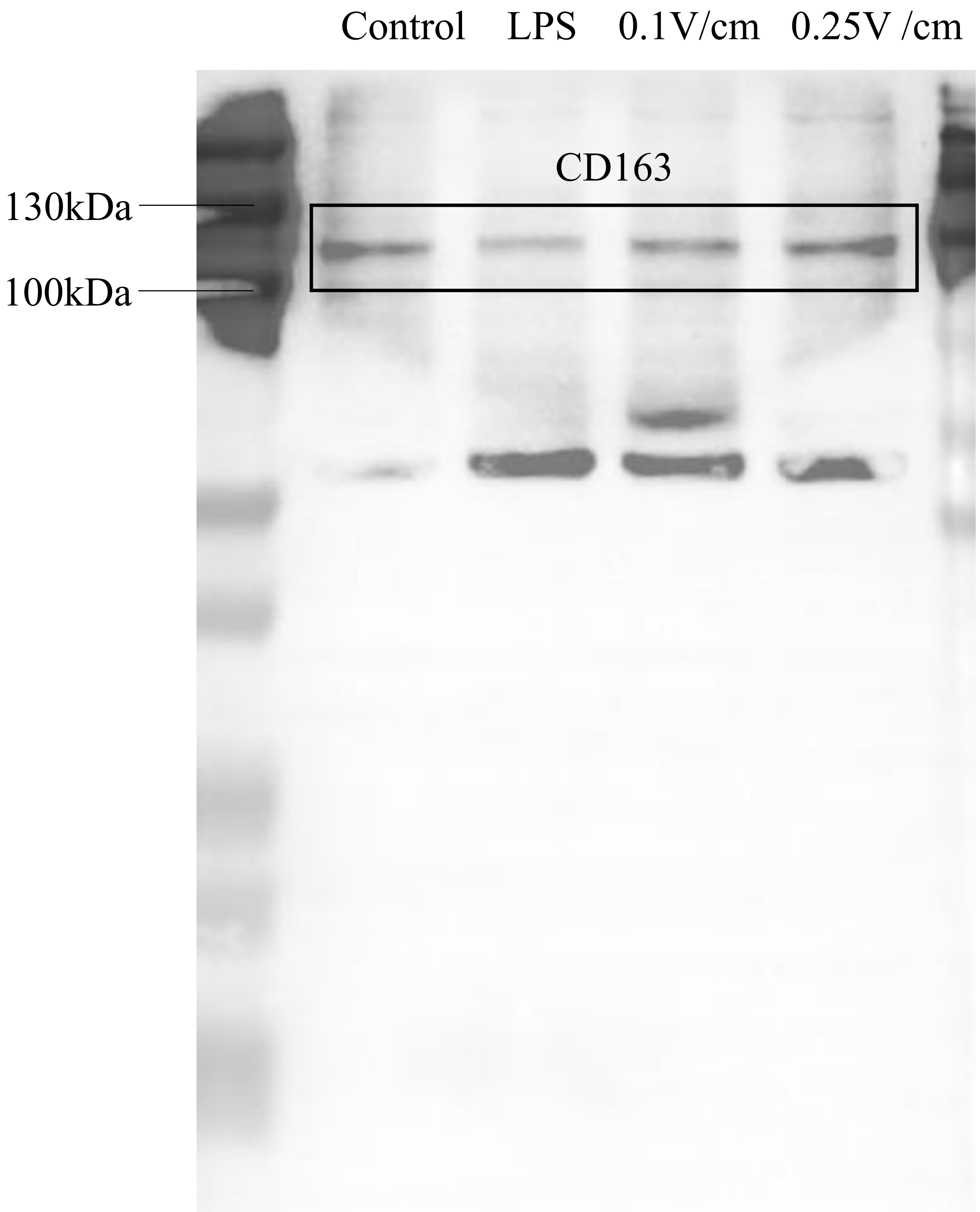
Control
LPS
0.1V/cm
0.25V /cm
CD163
130kDa
100kDa

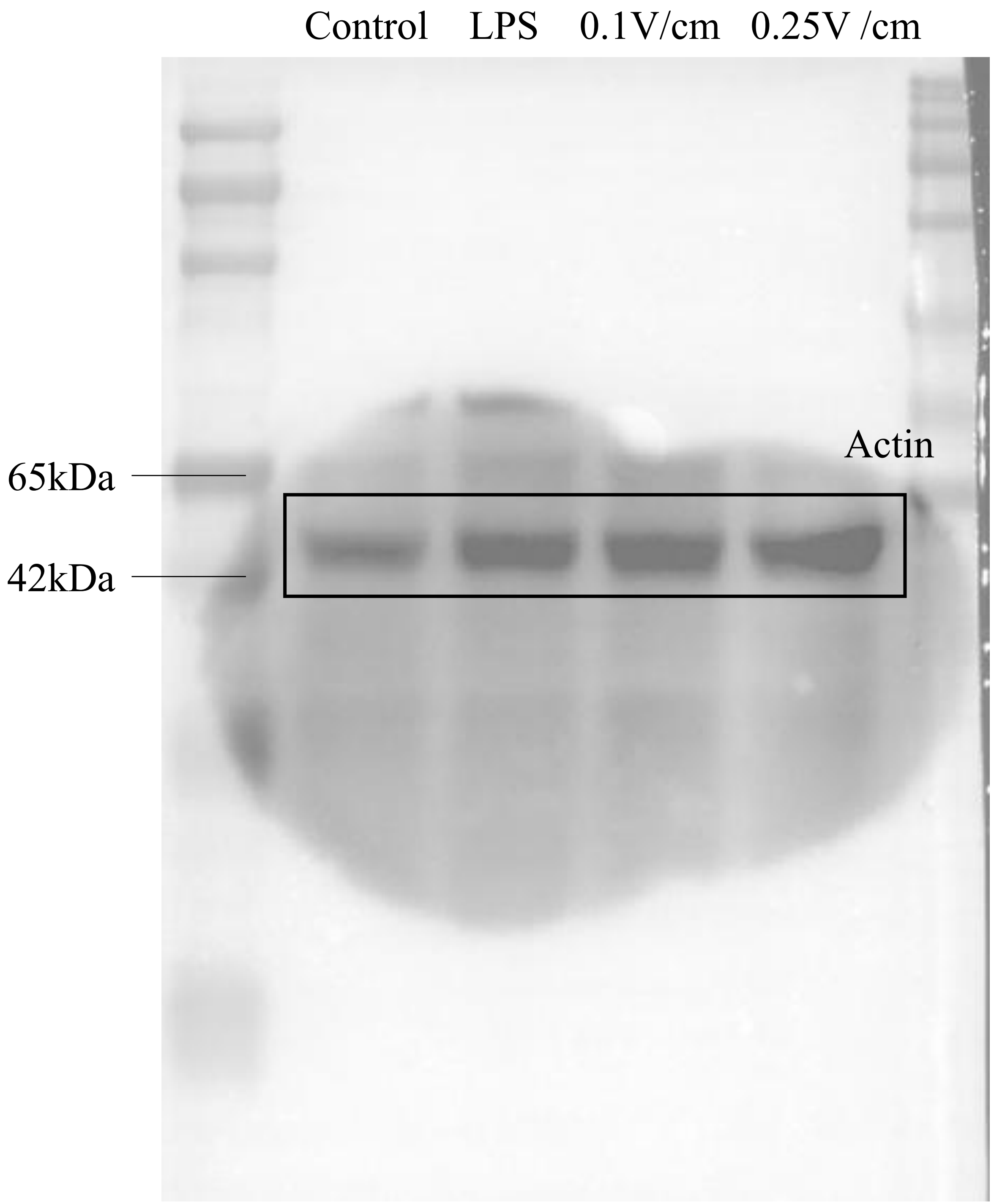
Control
LPS
0.1V/cm
0.25V /cm
Actin
65kDa
42kDa

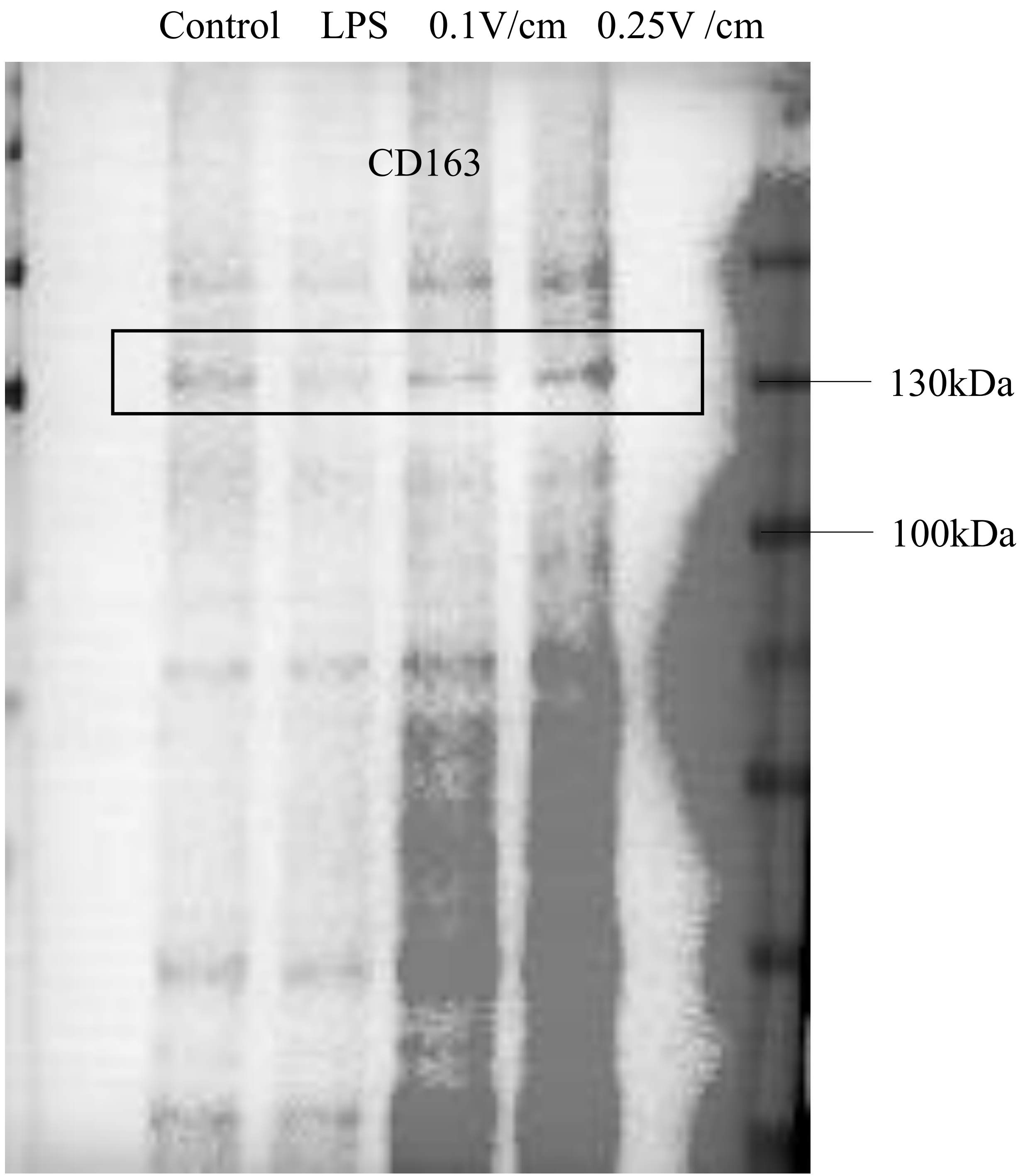
Control
LPS
0.1V/cm
0.25V /cm
CD163
130kDa
100kDa

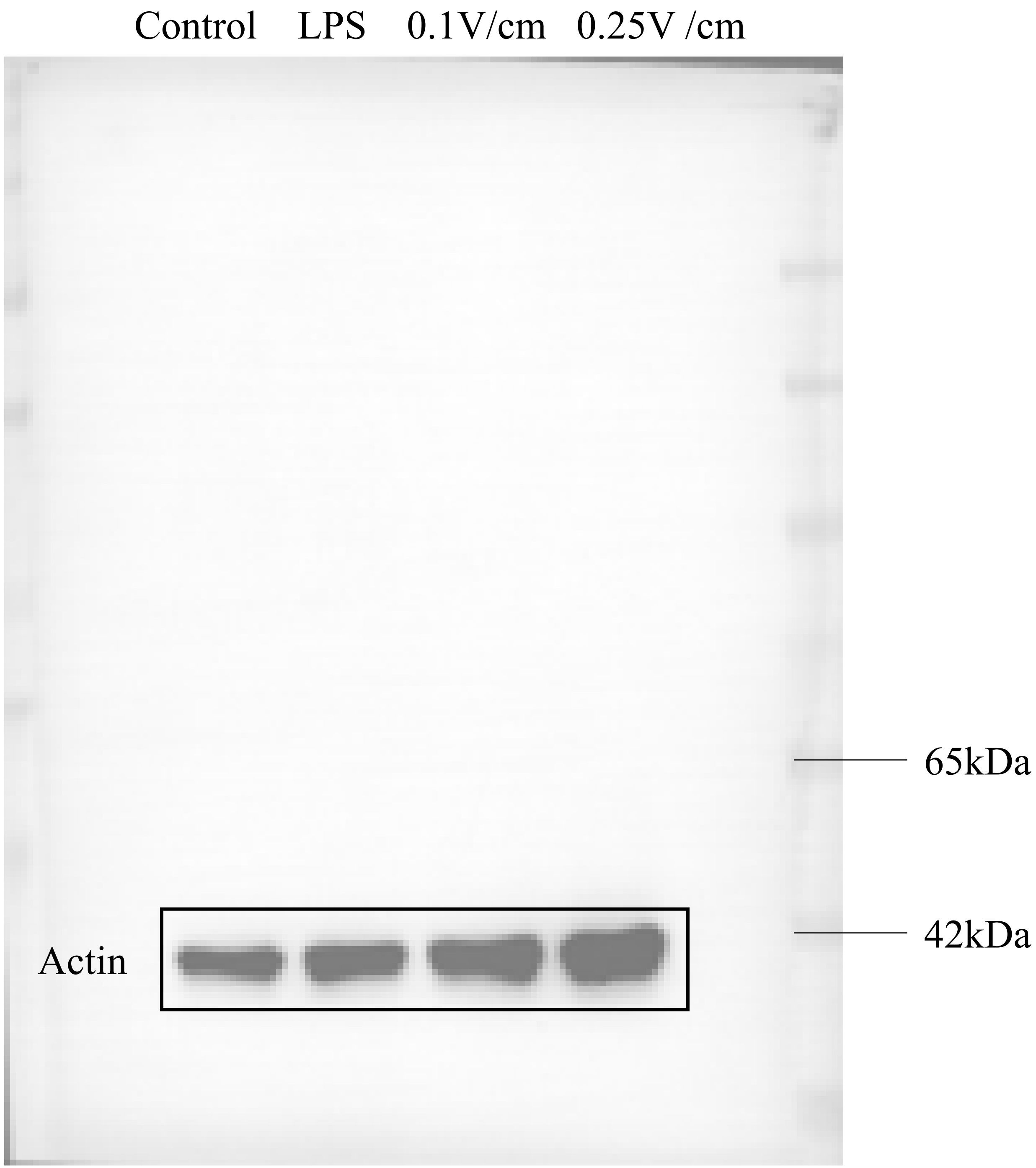
Control
LPS
0.1V/cm
0.25V /cm
Actin
65kDa
42kDa

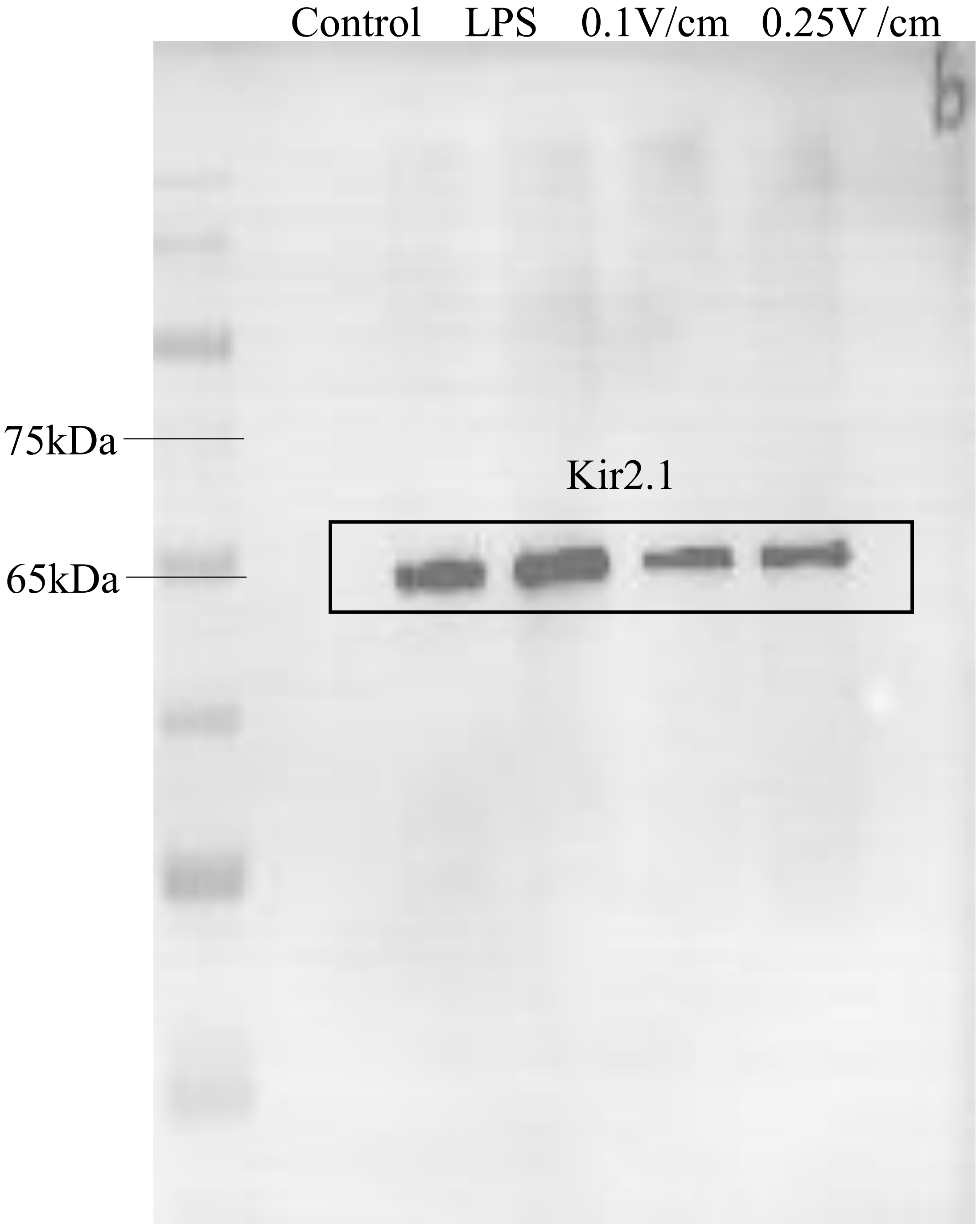

Control LPS 0.1V/cm 0.25V /cm
75kDa
Kir2.1
65kDa

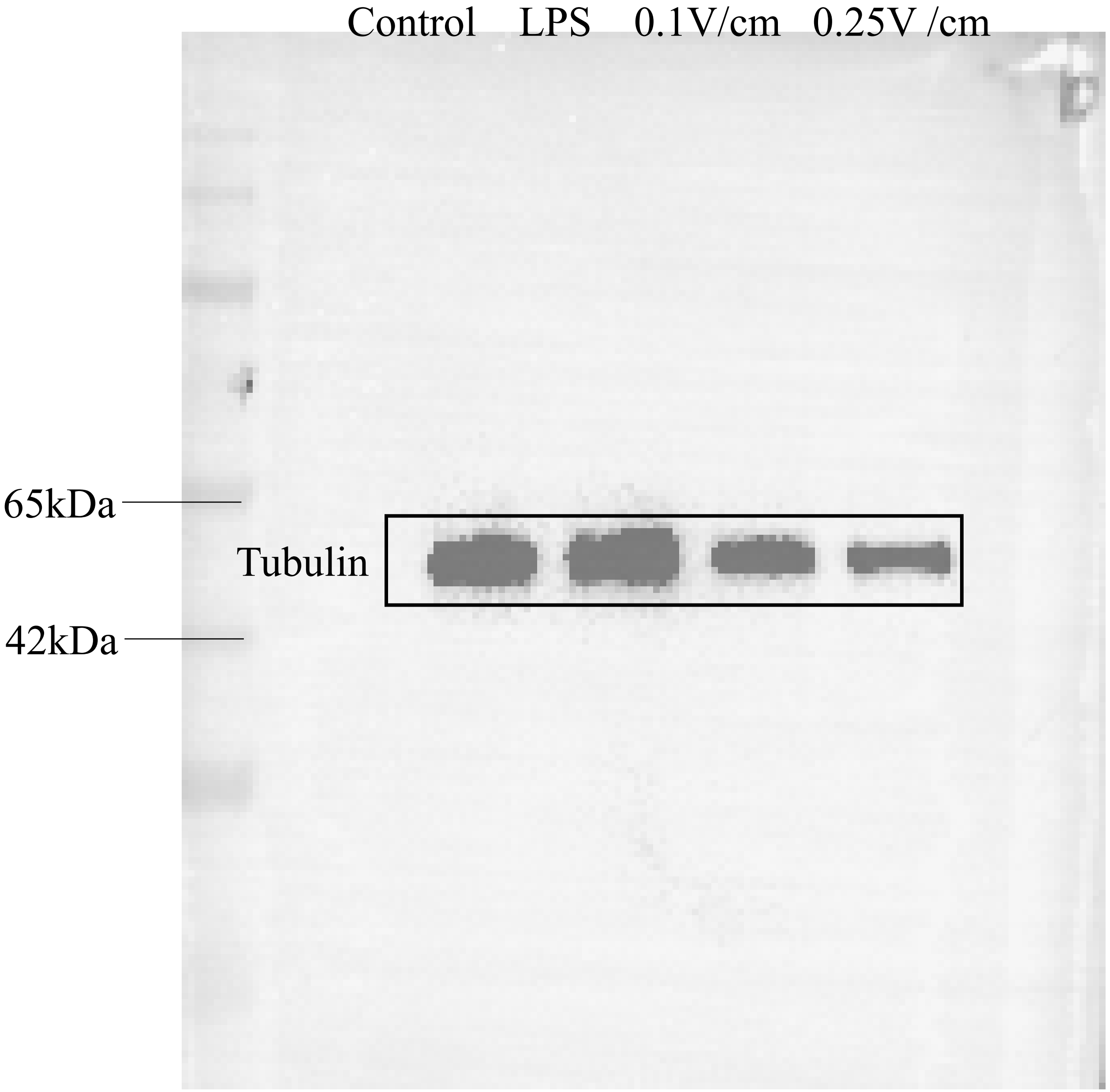
Control
LPS
0.1V/cm
0.25V /cm
65kDa
Tubulin
42kDa

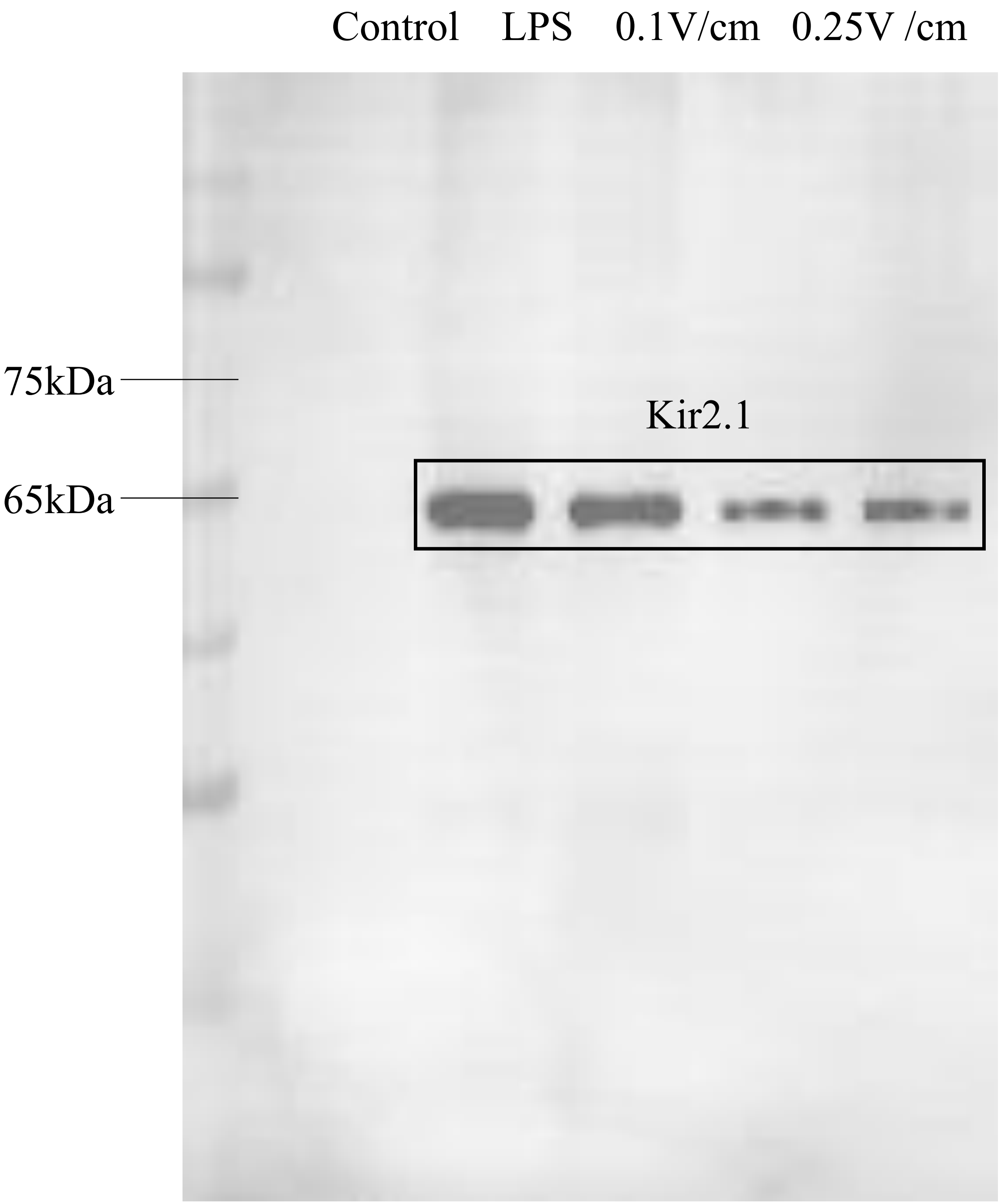
Control
LPS
0.1V/cm
0.25V /cm
75kDa
65kDa
Kir2.1

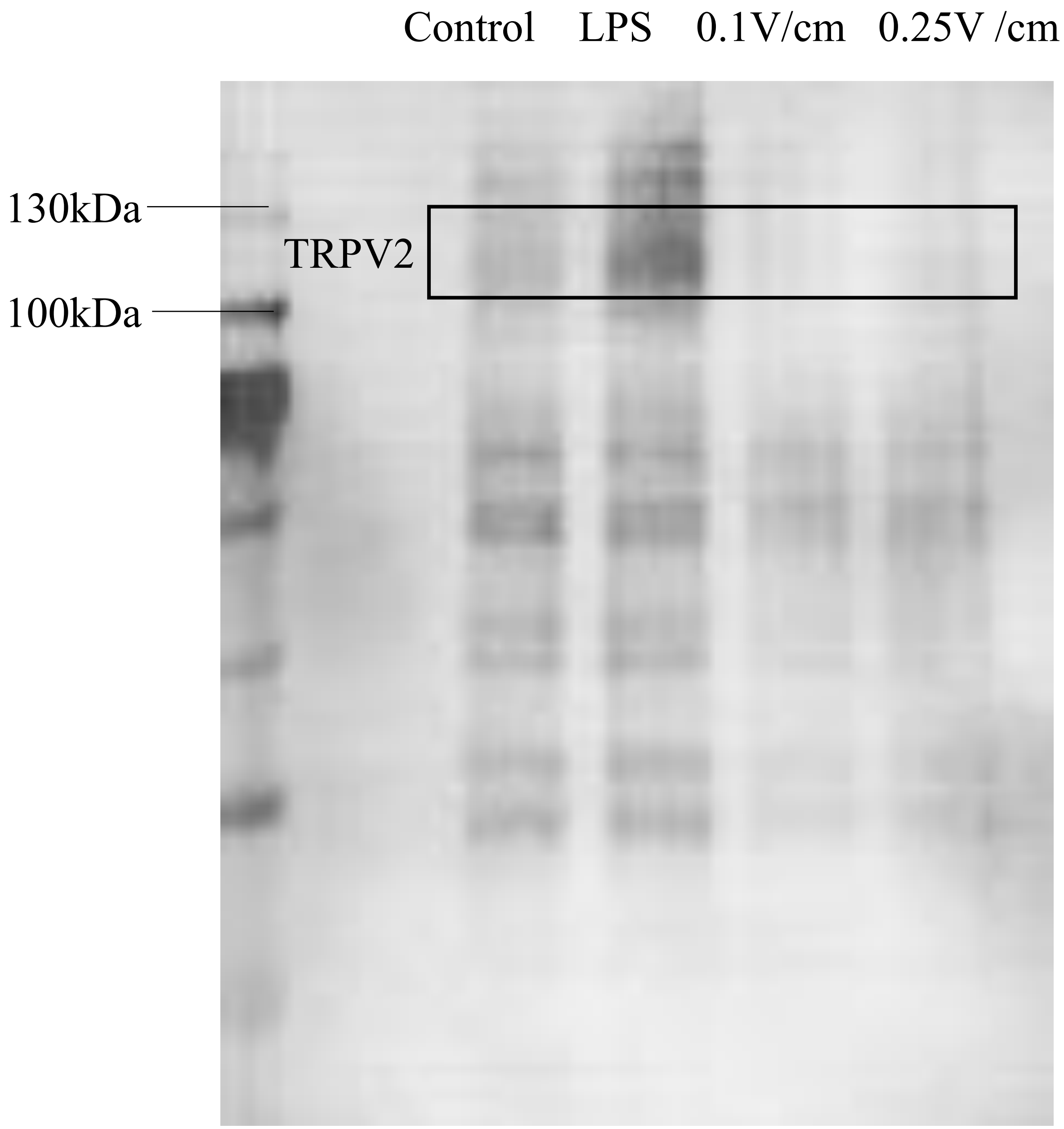
Control
LPS
0.1V/cm
0.25V /cm
130kDa
100kDa
TRPV2

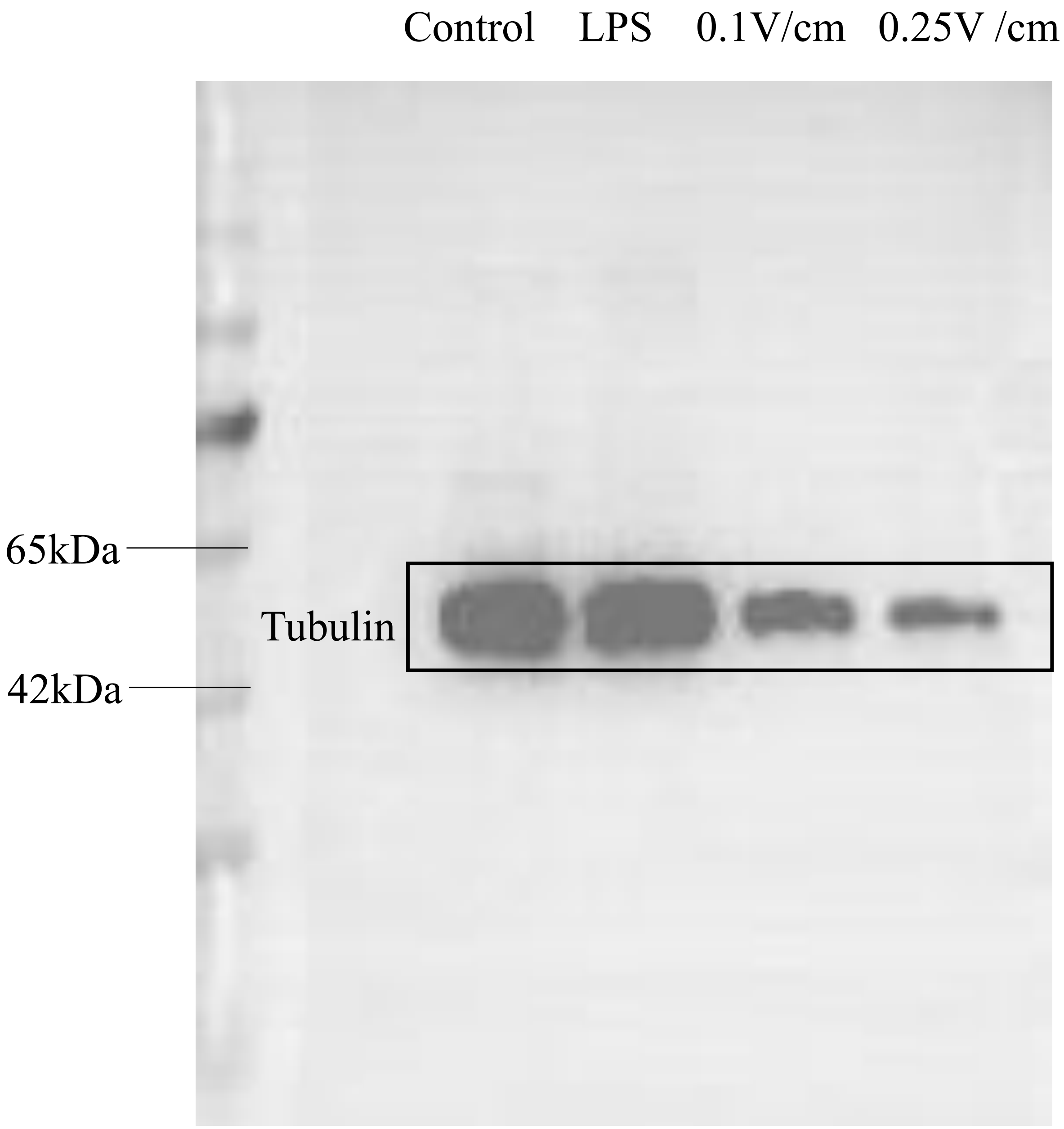
Control
LPS
0.1V/cm
0.25V /cm
65kDa
Tubulin
42kDa

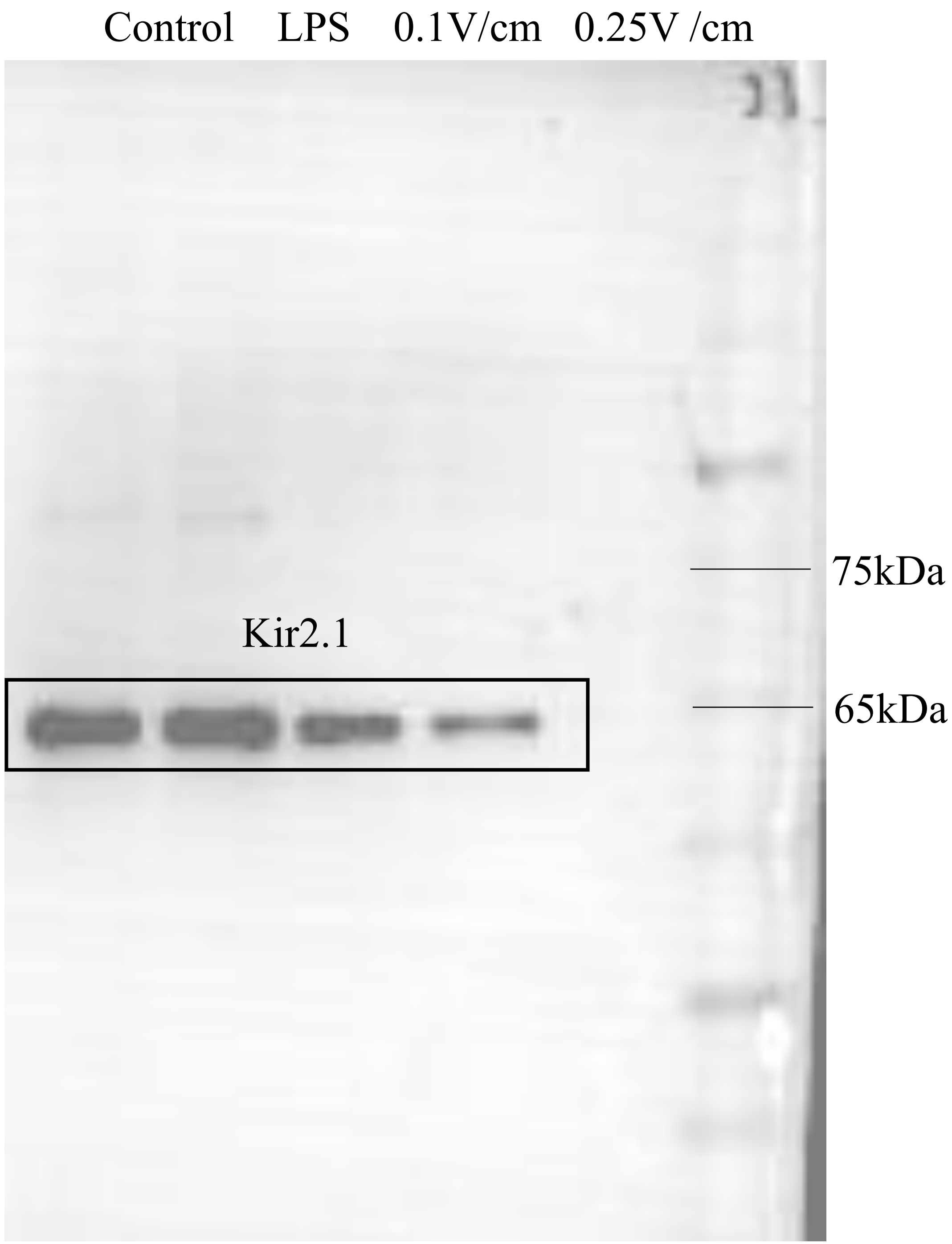
Control
LPS
0.1V/cm
0.25V /cm
Kir2.1
75kDa
65kDa

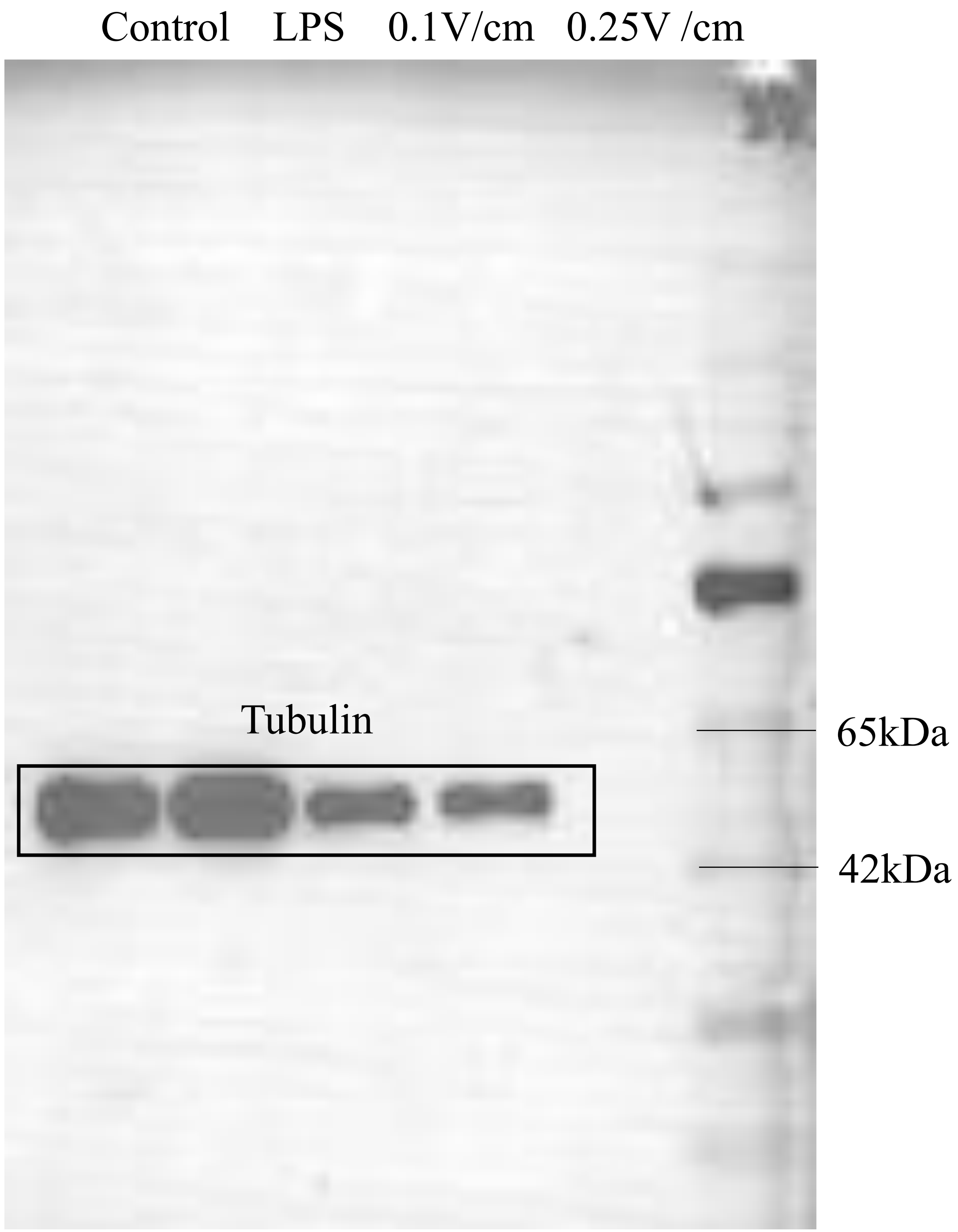
Control LPS 0.1V/cm 0.25V /cm
Tubulin
65kDa
42kDa

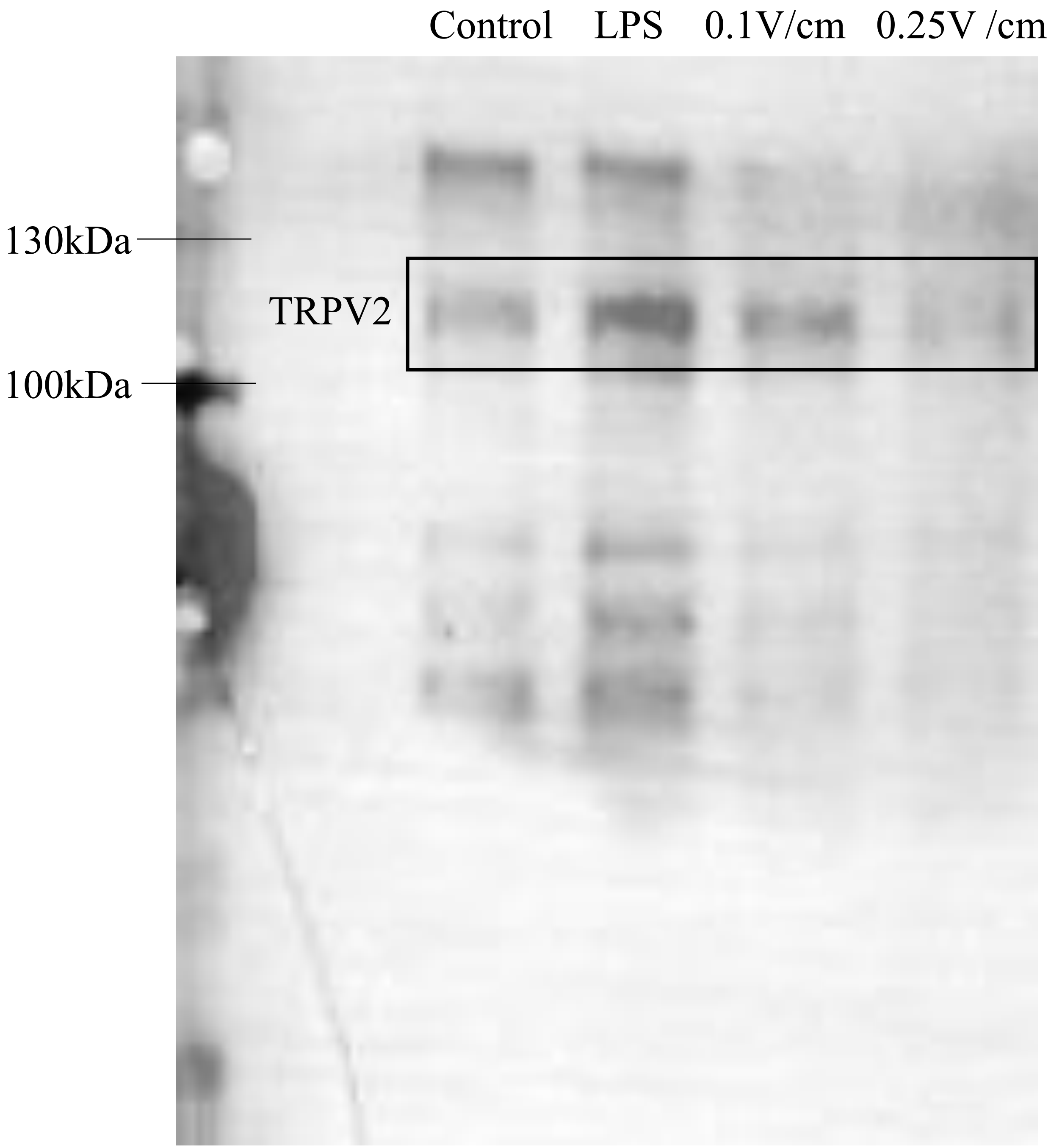
Control
LPS
0.1V/cm
0.25V /cm
130kDa
100kDa
TRPV2

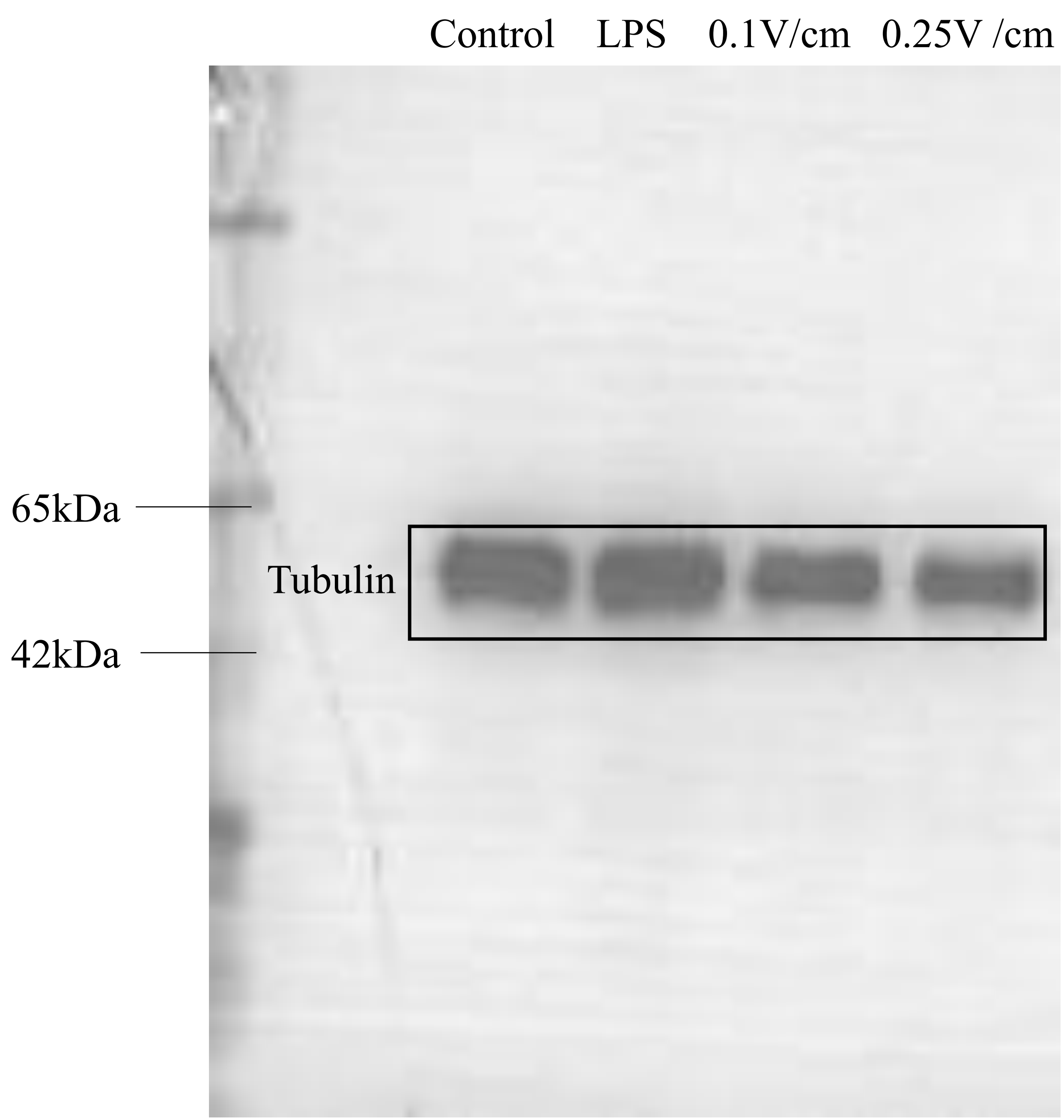
Control
LPS
0.1V/cm
0.25V /cm
65kDa
Tubulin
42kDa

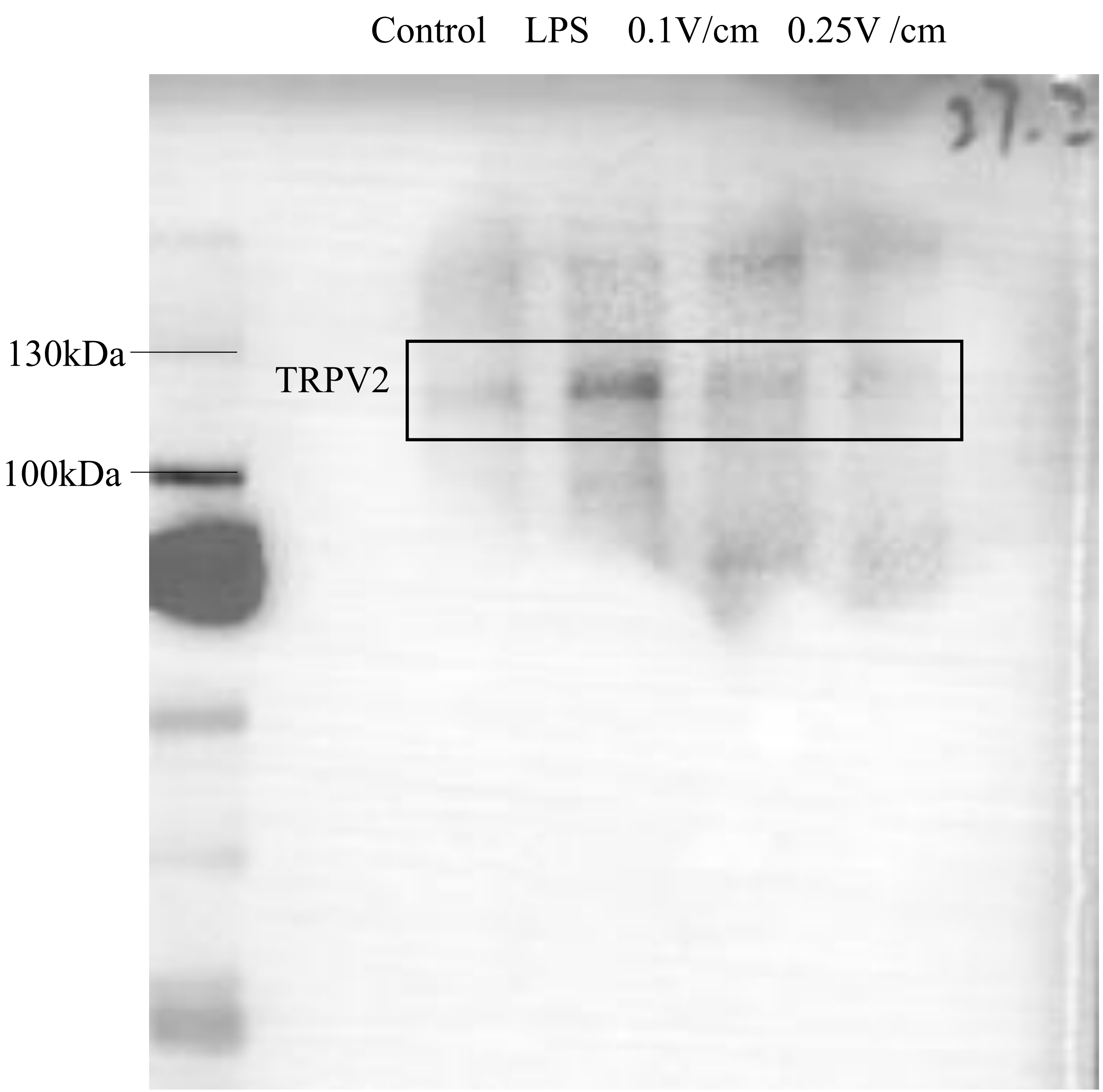
Control
LPS
0.1V/cm
0.25V /cm
130kDa
100kDa
TRPV2

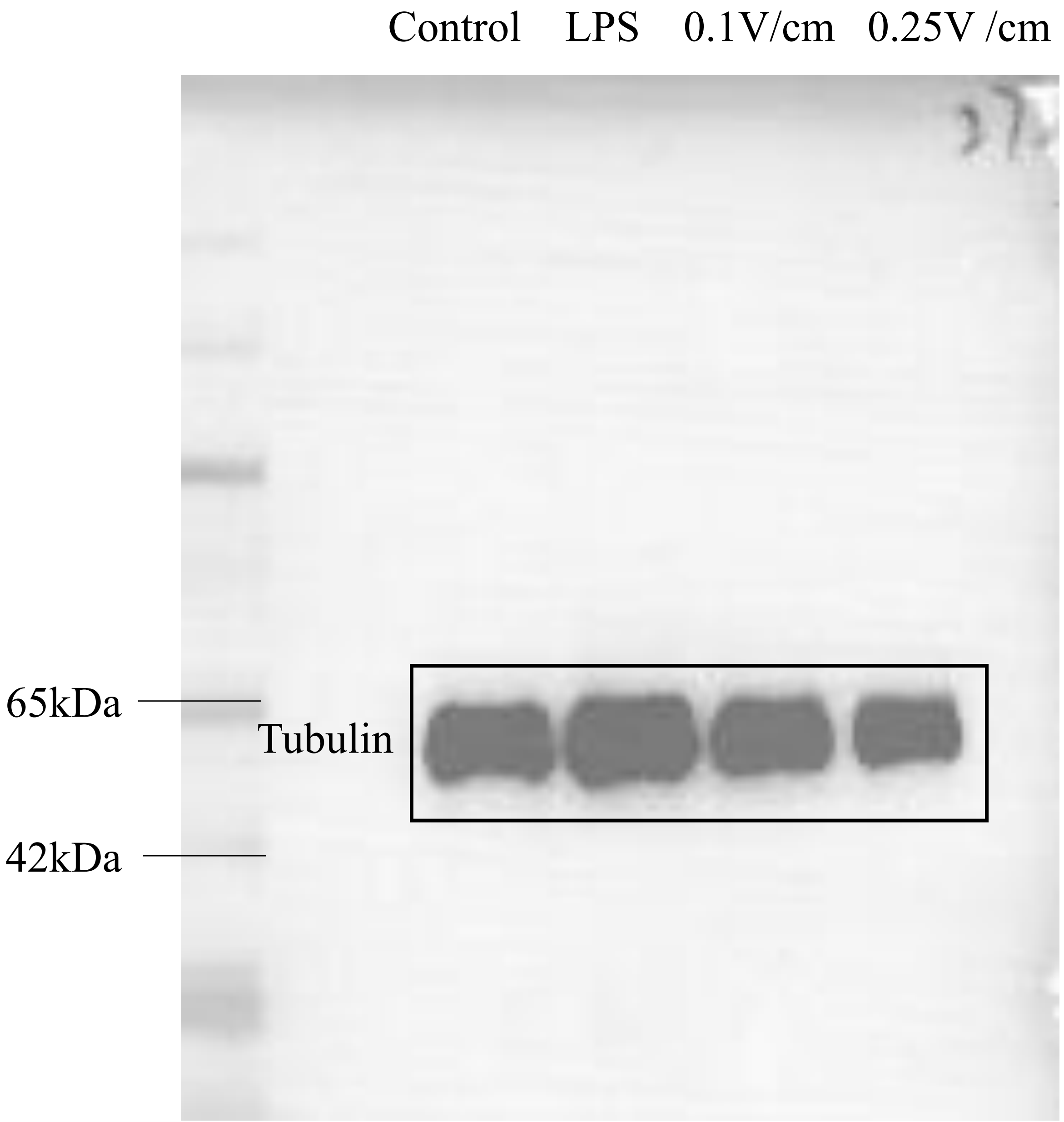
Control
LPS
0.1V/cm
0.25V /cm
65kDa
Tubulin
42kDa

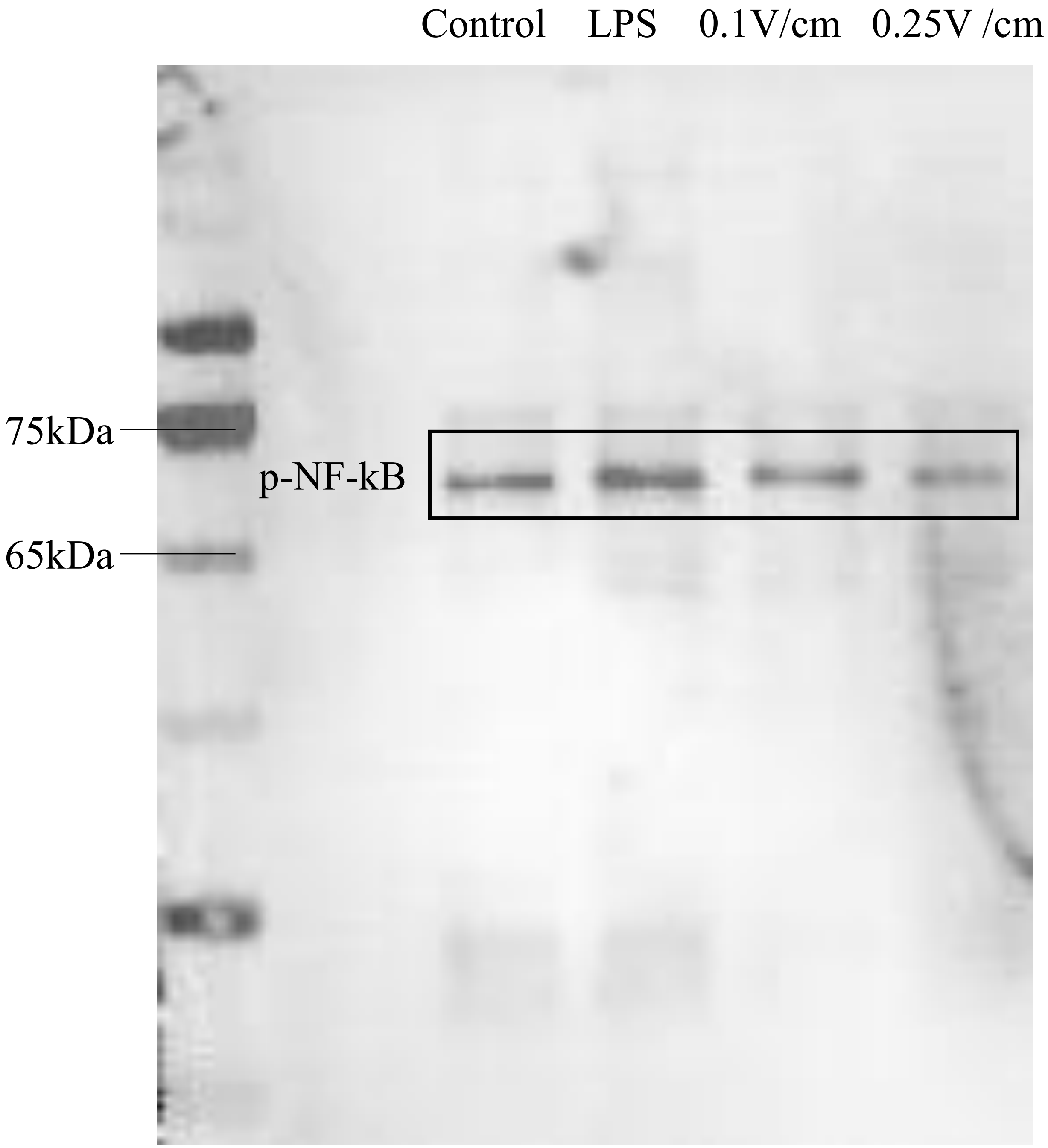

Control
LPS
0.1V/cm
0.25V /cm
75kDa
p-NF-kB
65kDa

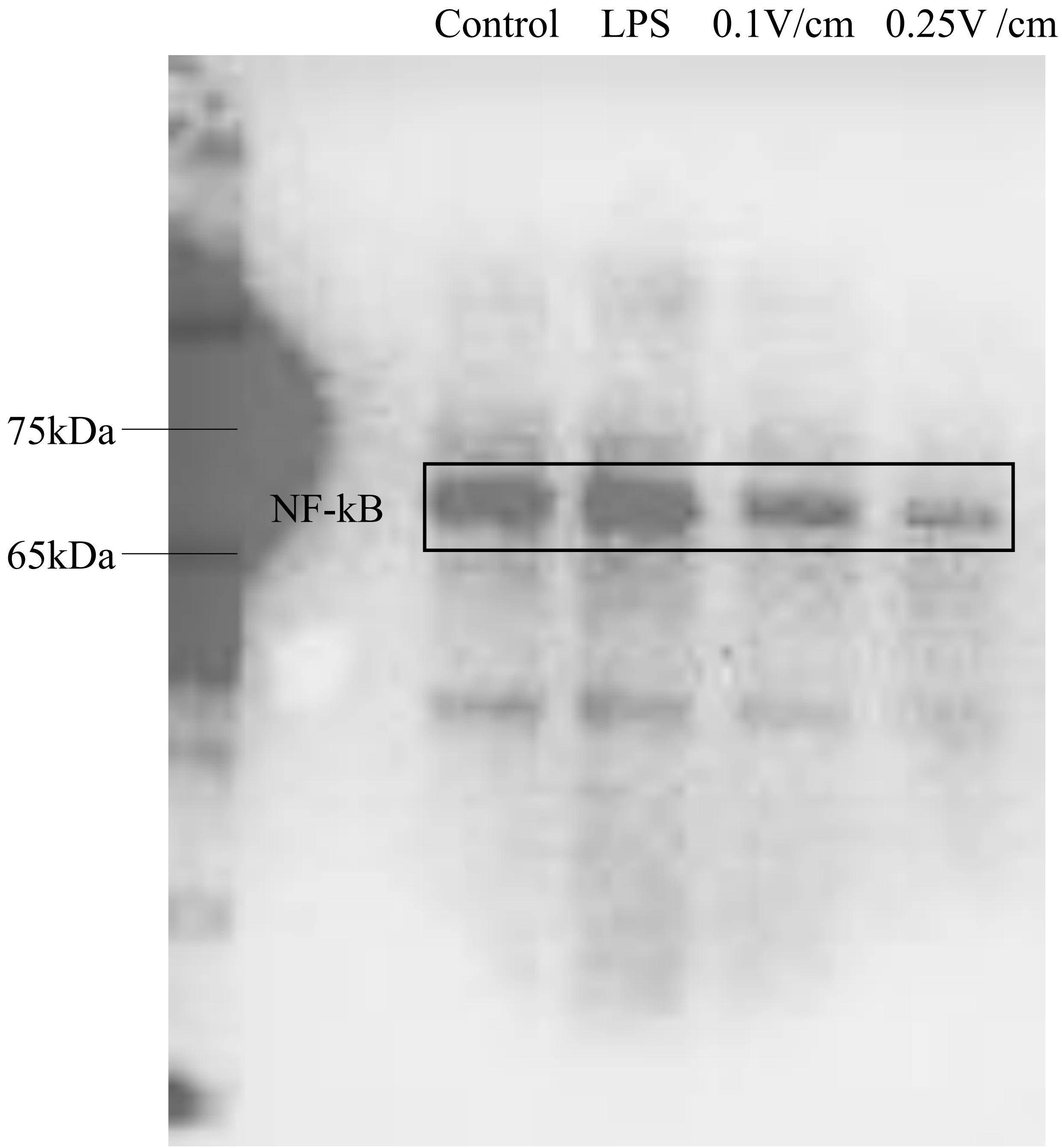
Control
LPS
0.1V/cm
0.25V /cm
75kDa
NF-kB
65kDa

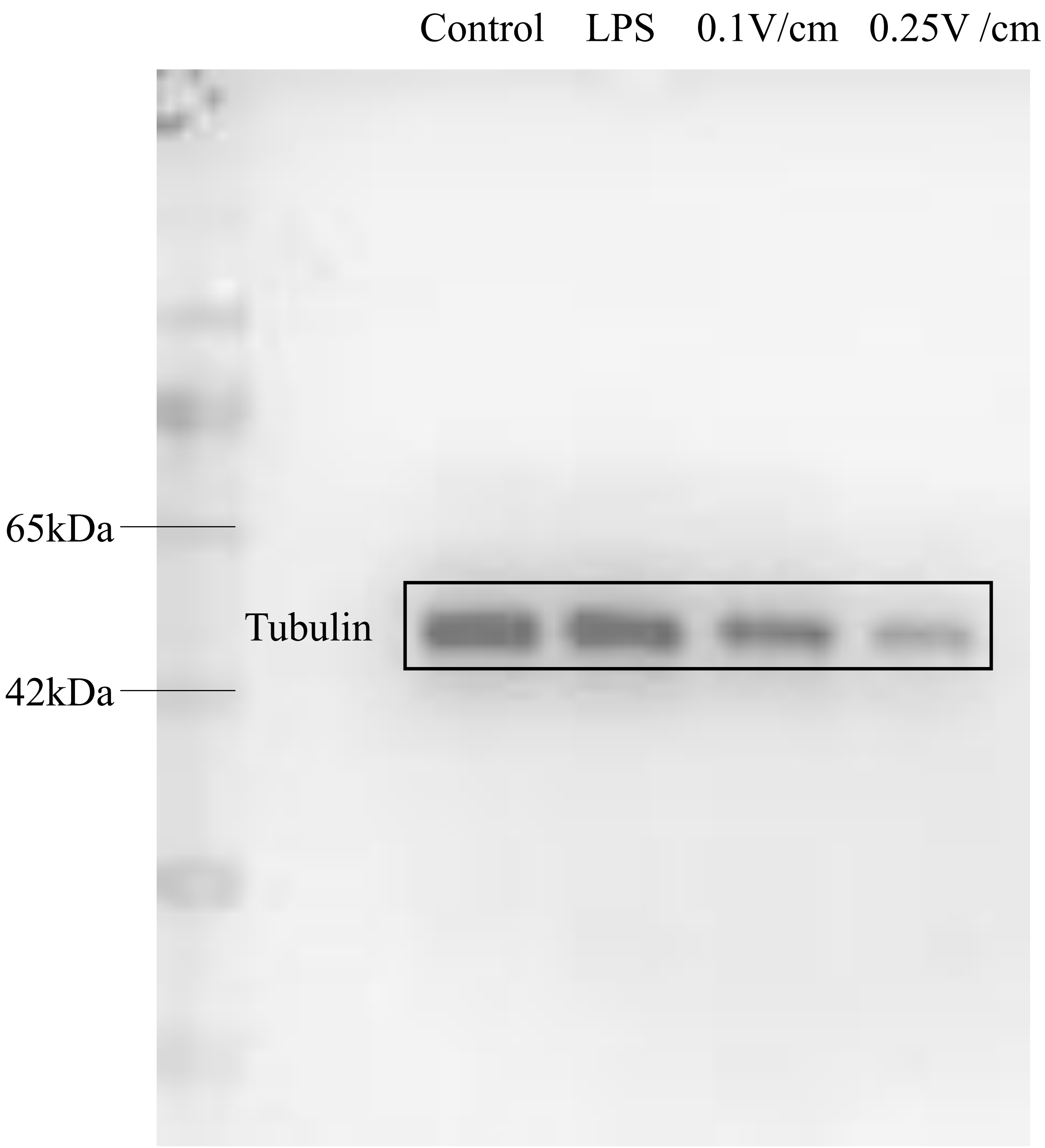
Control LPS 0.1V/cm 0.25V /cm
65kDa
Tubulin
42kDa

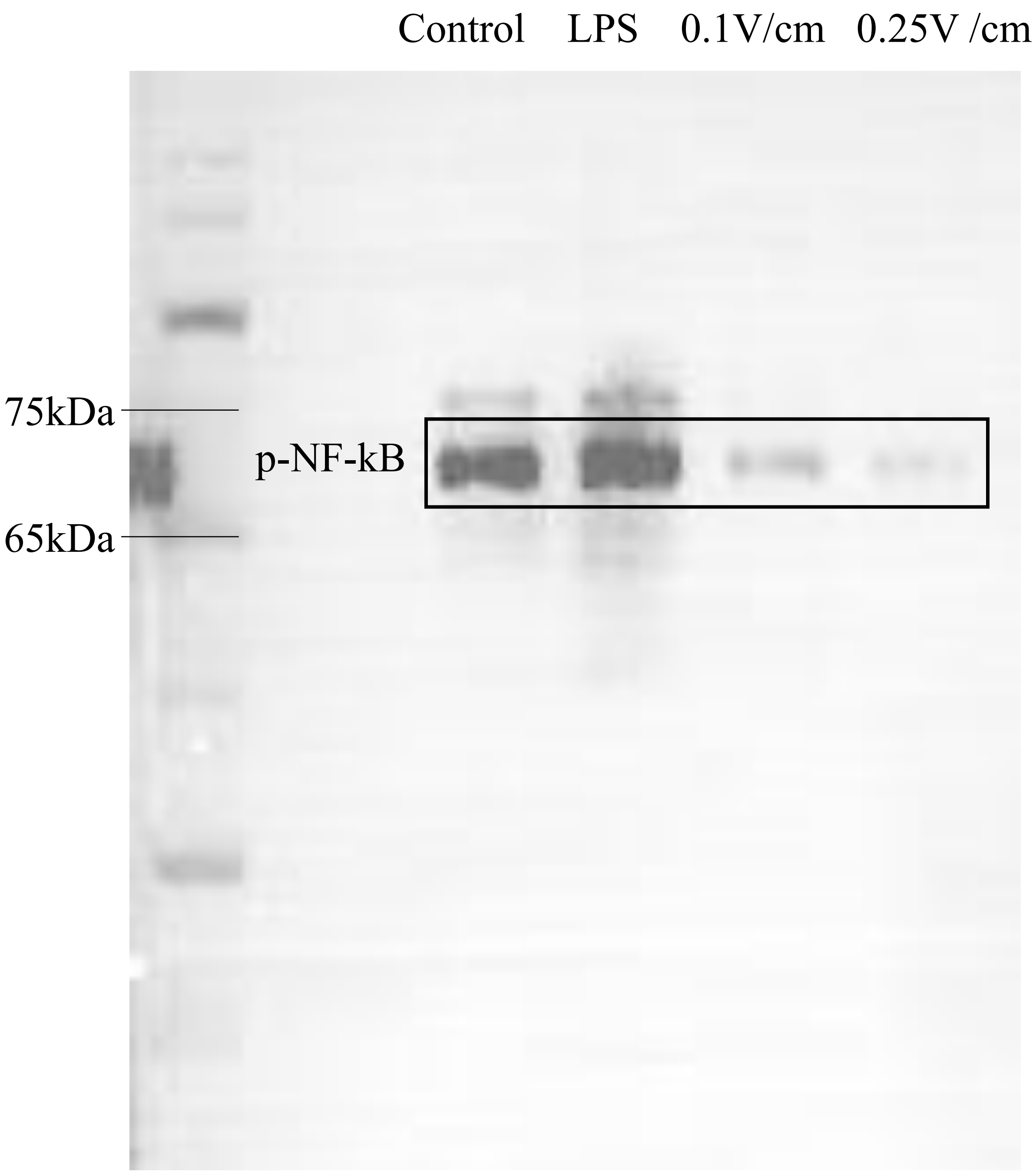
Control LPS 0.1V/cm 0.25V /cm
75kDa
p-NF-kB
65kDa

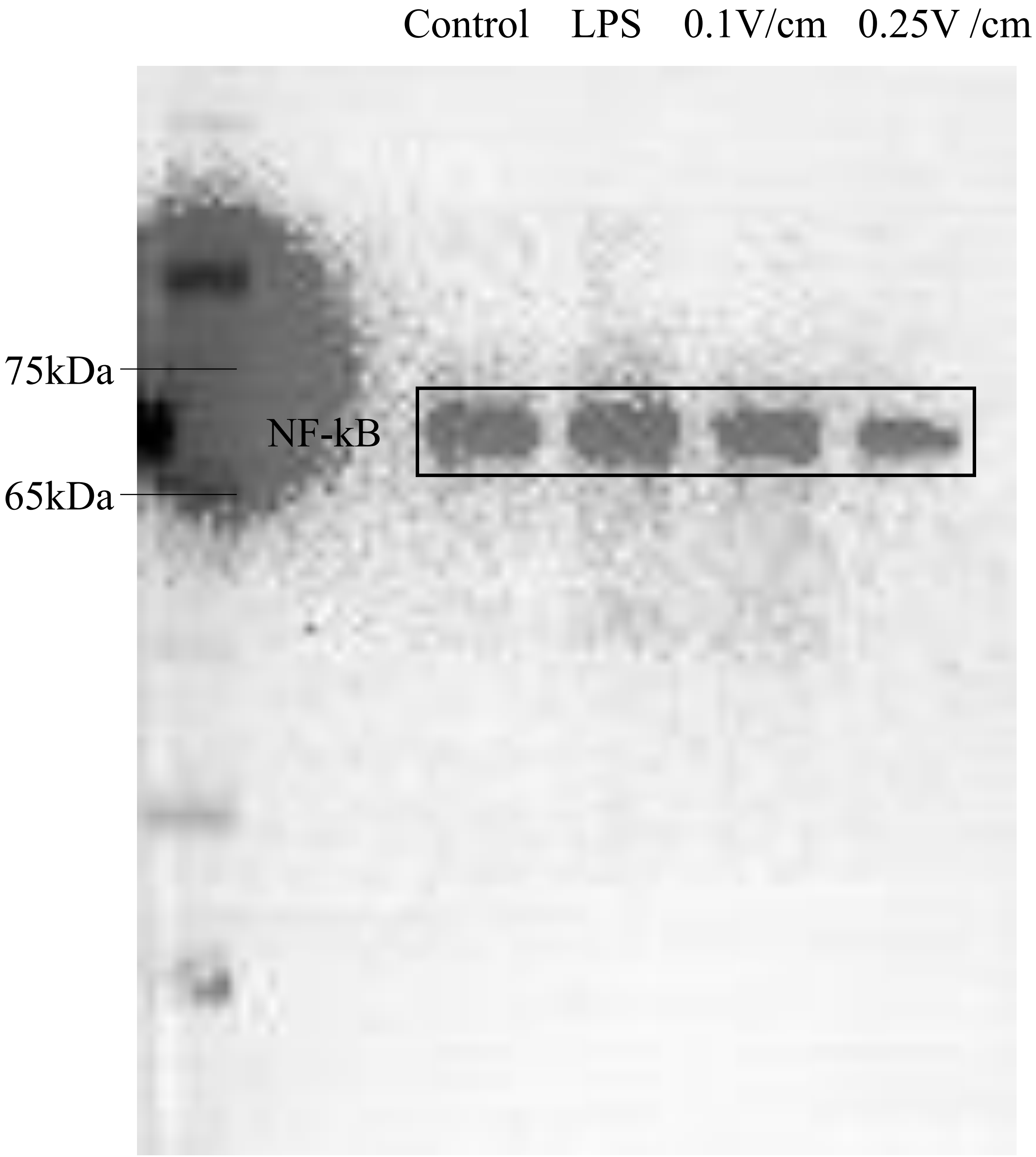
Control LPS 0.1V/cm 0.25V /cm
75kDa
NF-kB
65kDa

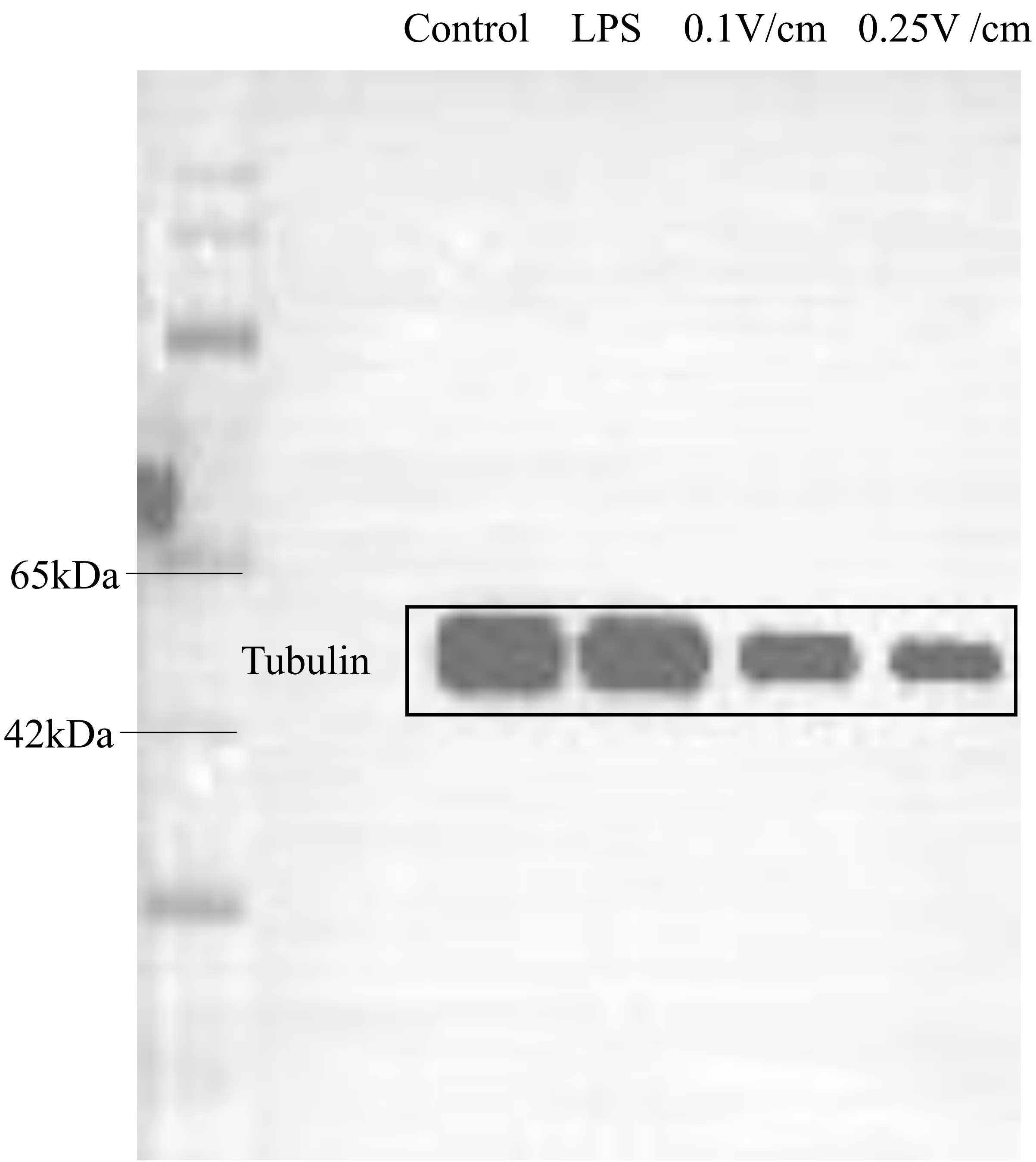
Control
LPS
0.1V/cm
0.25V /cm
65kDa
Tubulin
42kDa

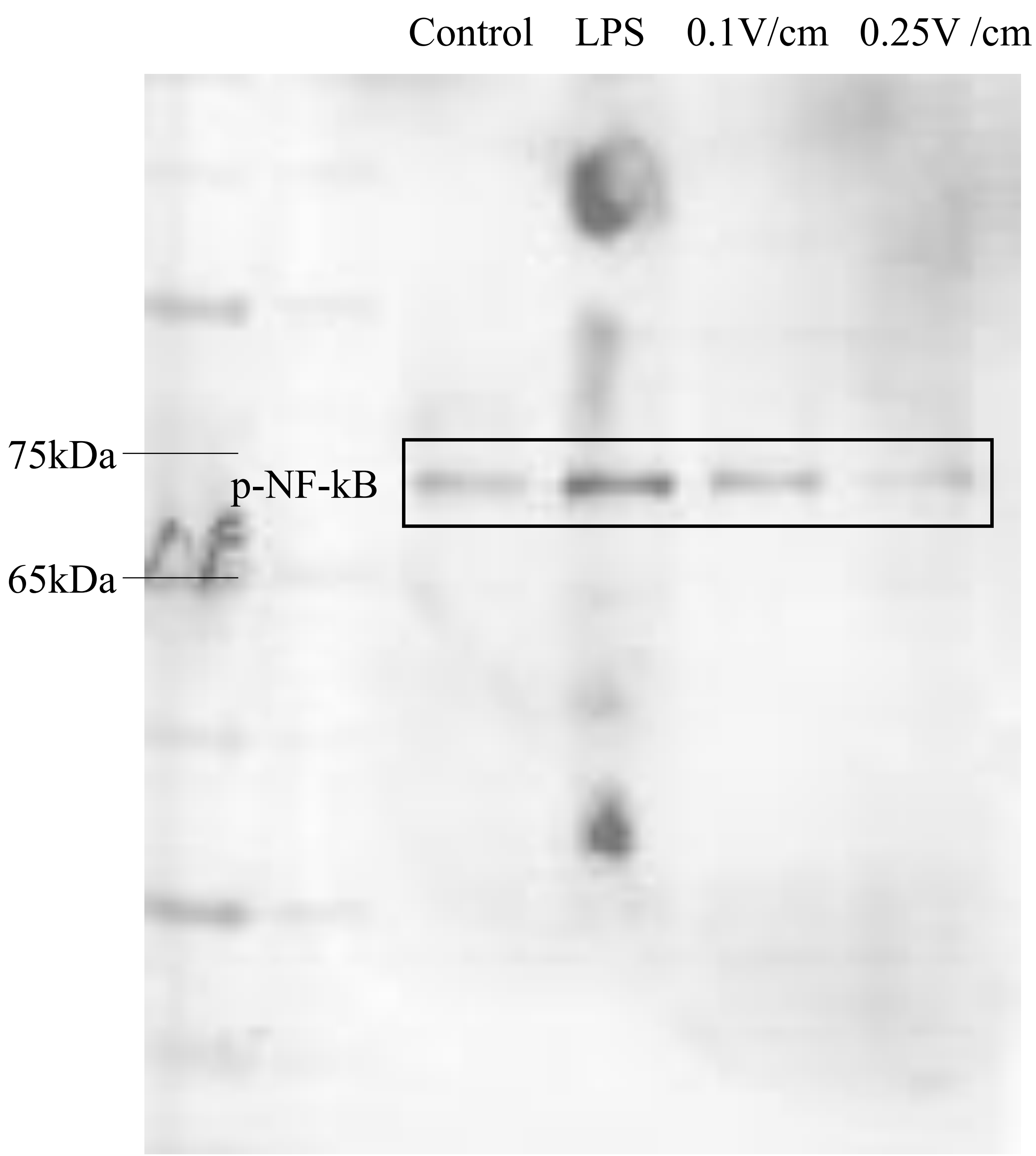
Control
LPS
0.1V/cm
0.25V /cm
75kDa
p-NF-kB
65kDa

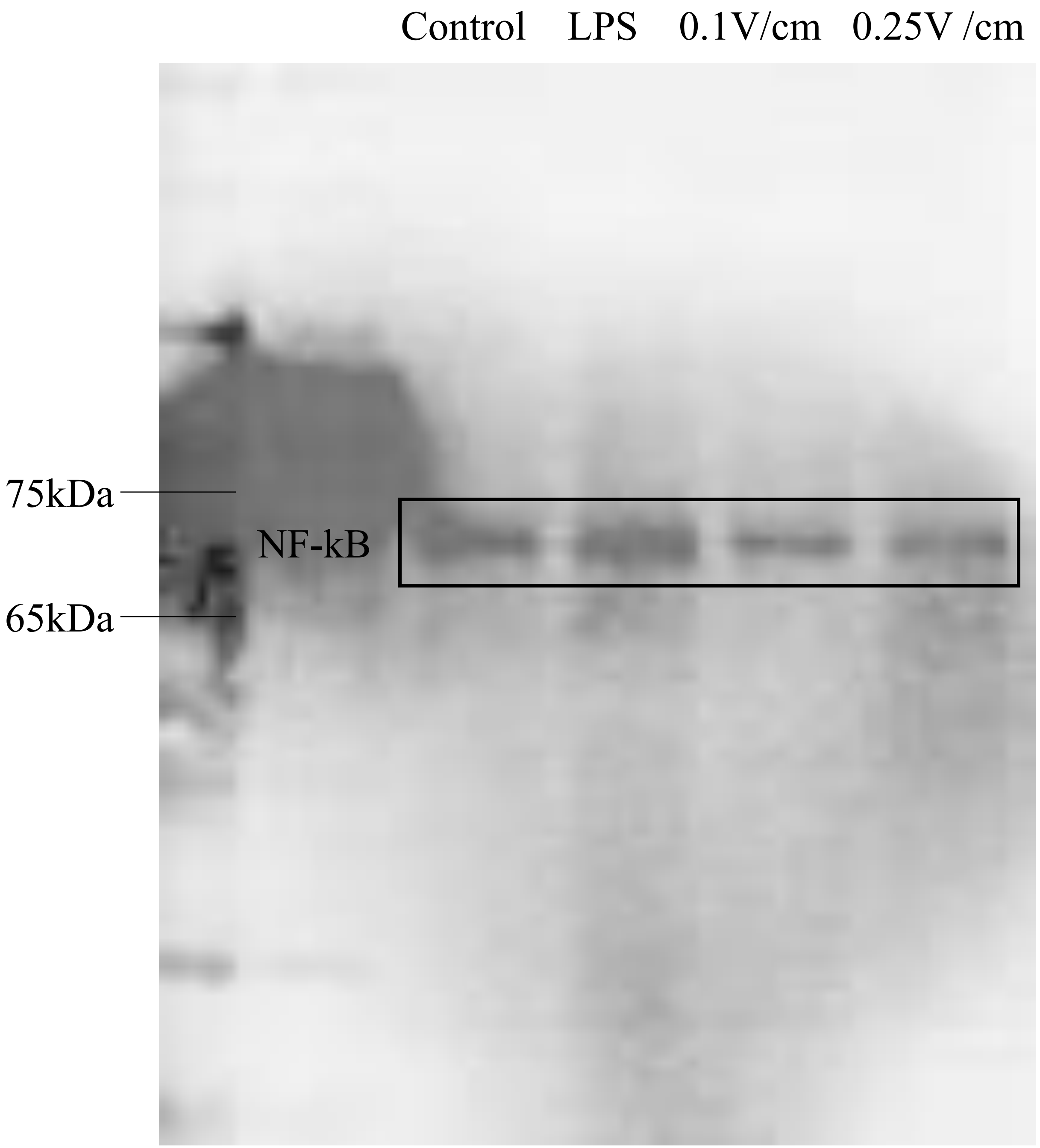
Control
LPS
0.1V/cm
0.25V /cm
75kDa
NF-kB
65kDa

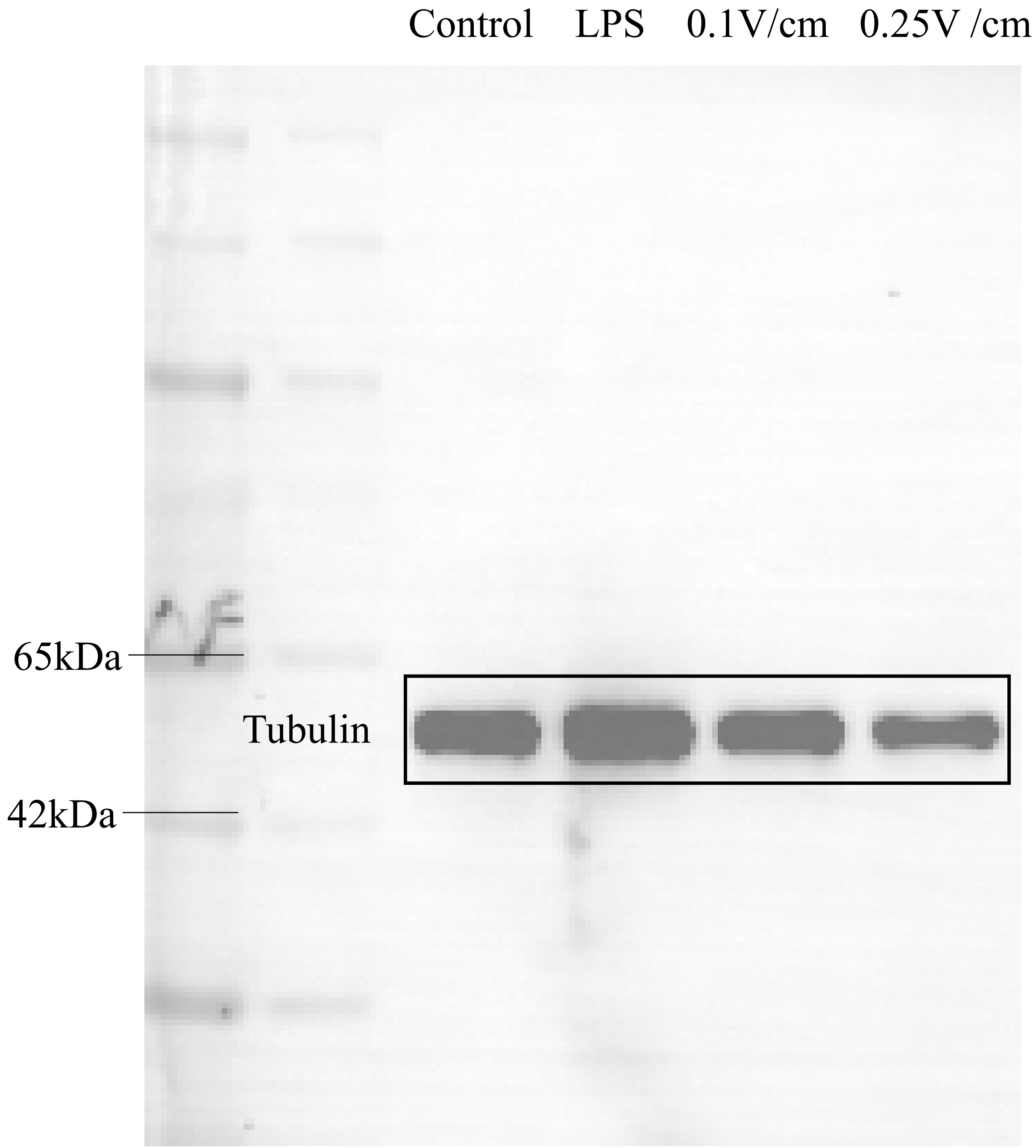
Control
LPS
0.1V/cm
0.25V /cm
65kDa
Tubulin
42kDa

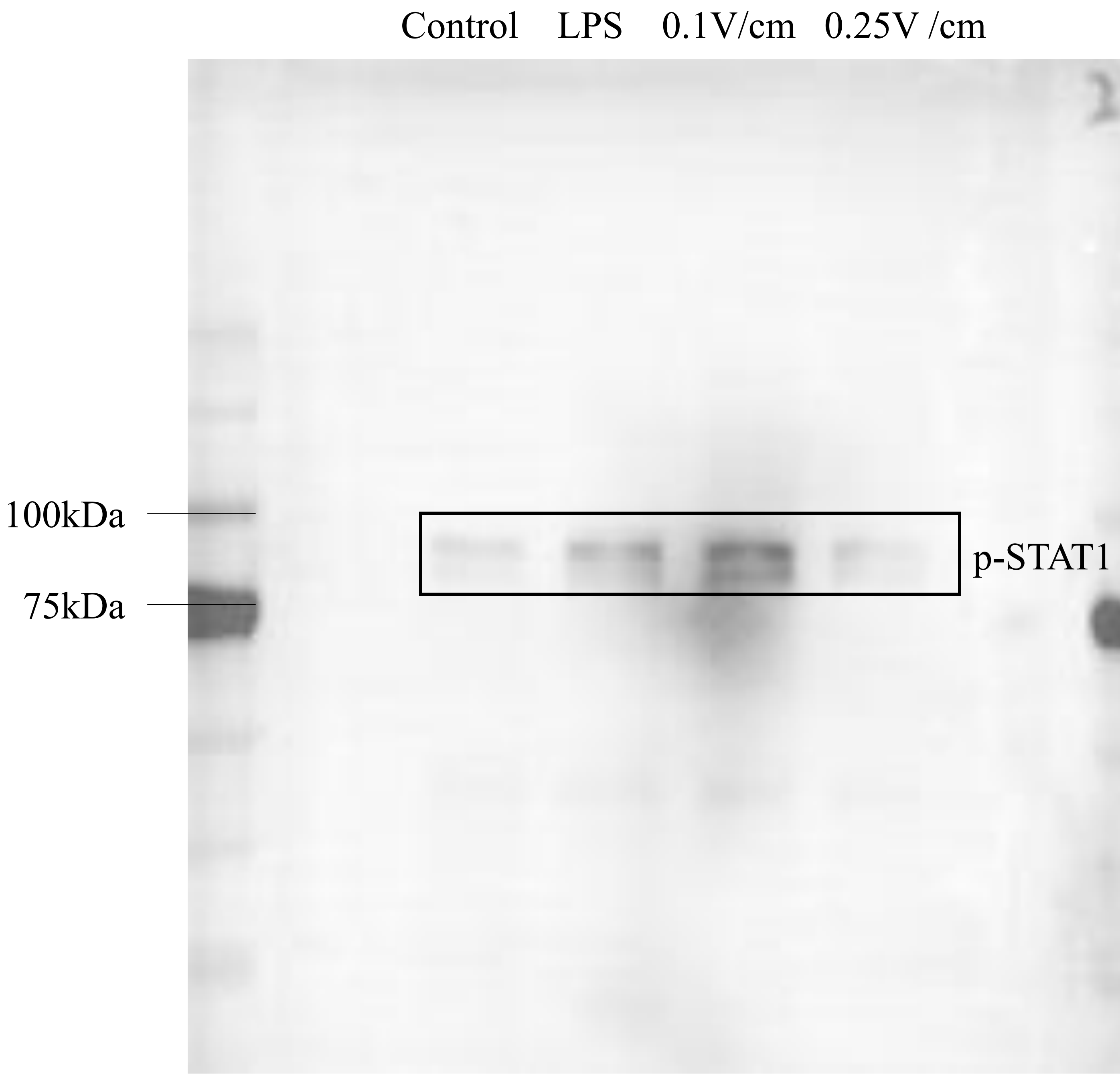
Control
LPS
0.1V/cm
0.25V /cm
100kDa
75kDa
p-STAT1

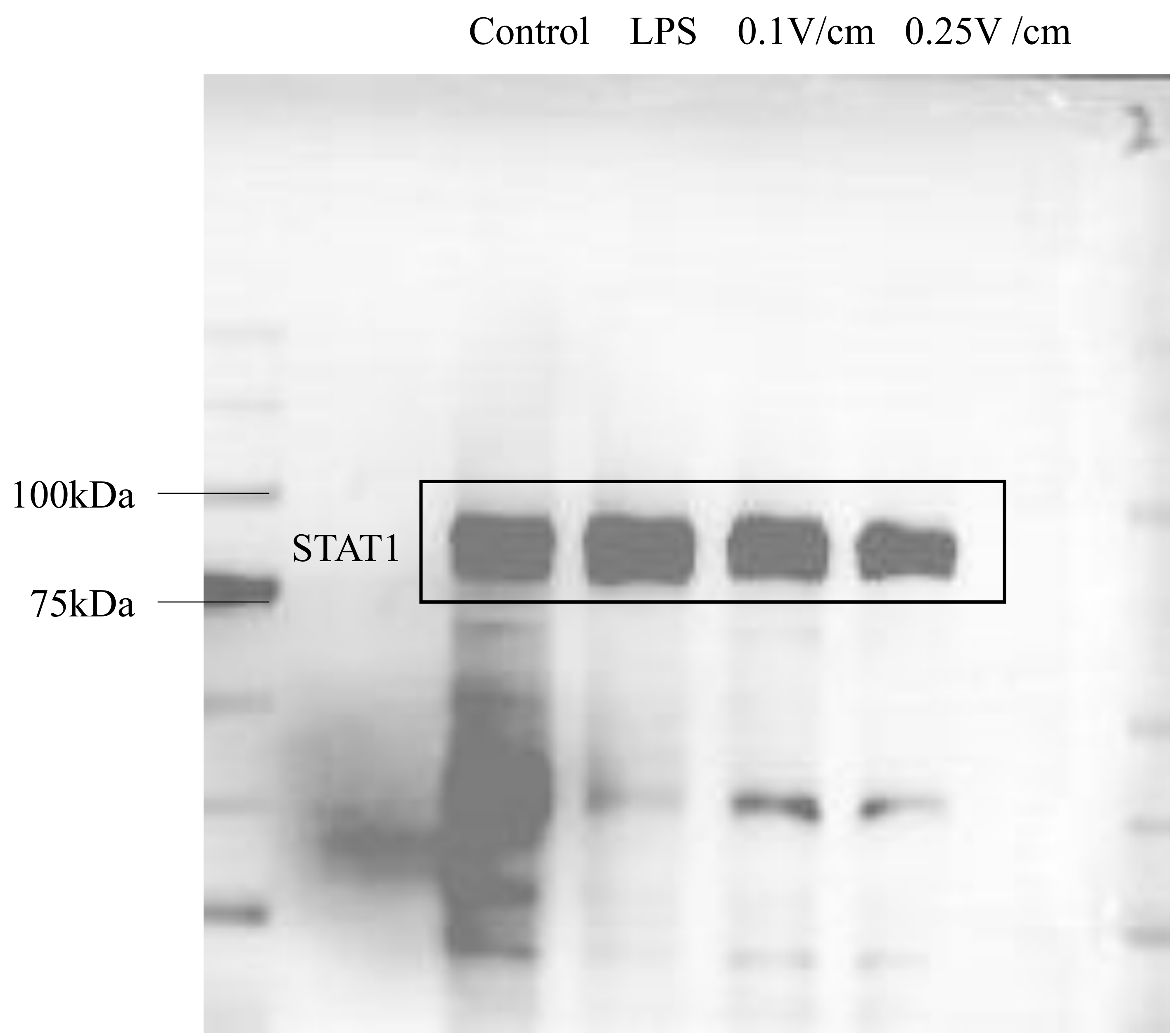
Control LPS 0.1V/cm 0.25V /cm
100kDa
STAT1
75kDa

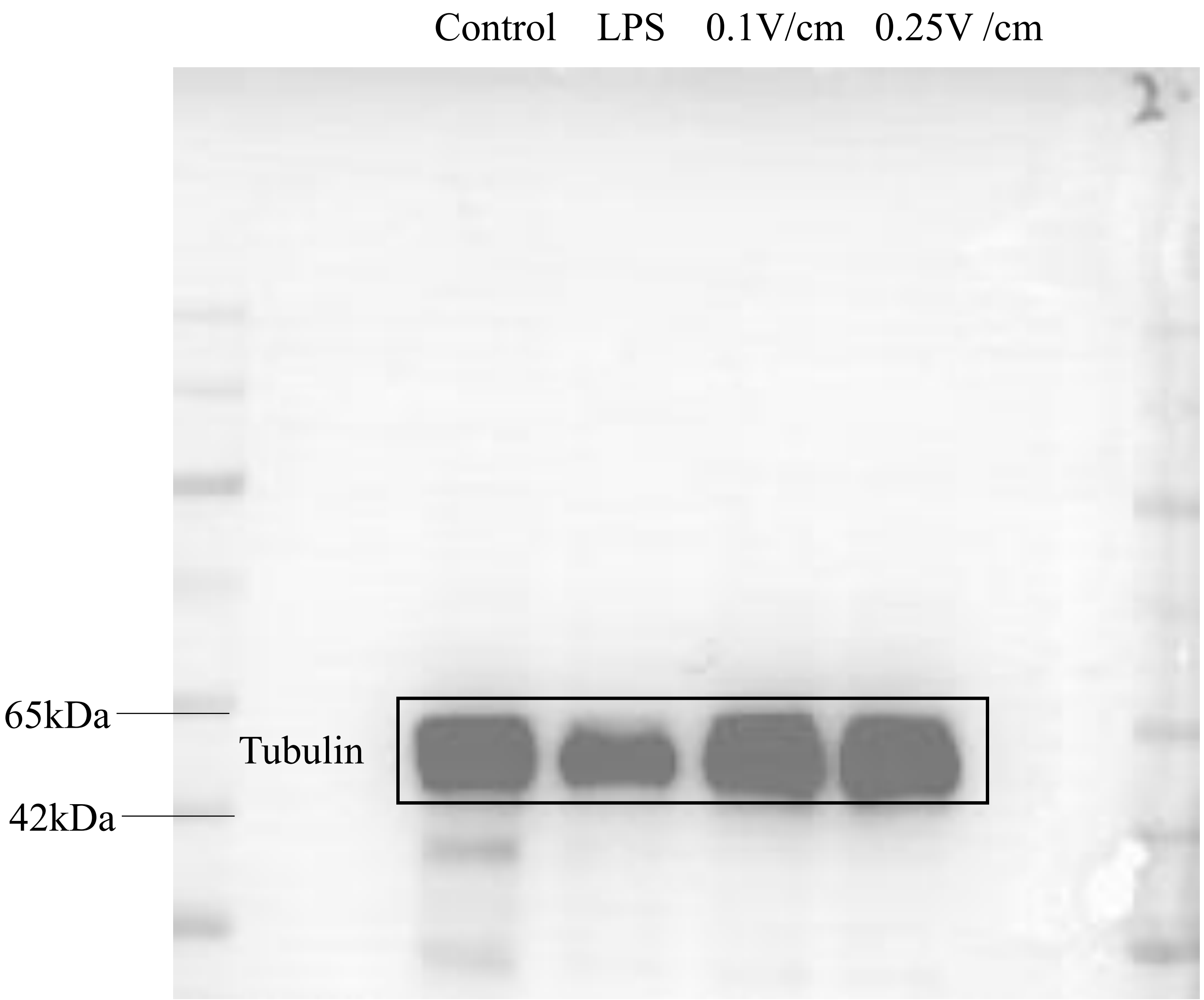
Control
LPS
0.1V/cm
0.25V /cm
65kDa
Tubulin
42kDa

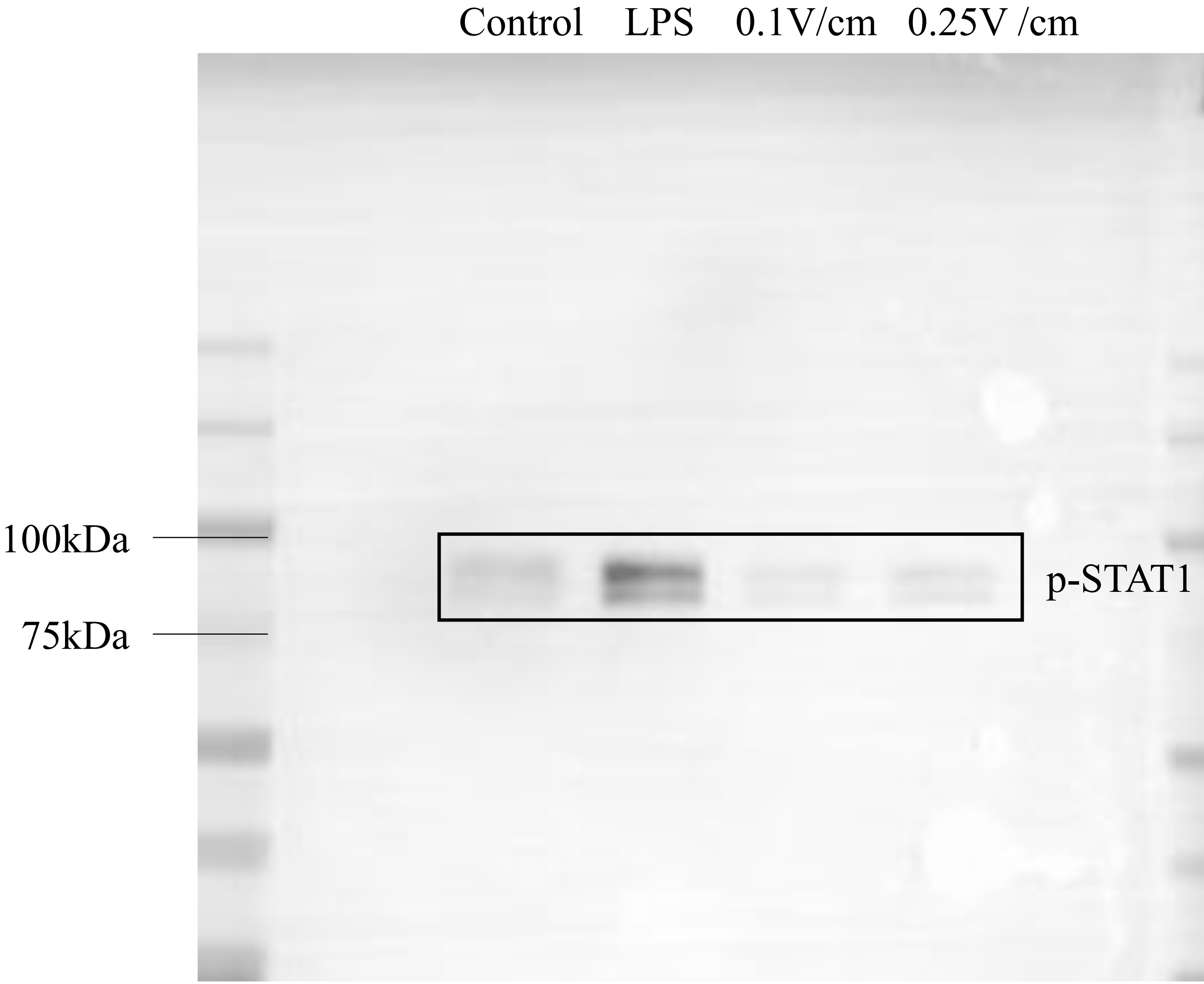
Control LPS 0.1V/cm 0.25V /cm
100kDa
75kDa
p-STAT1

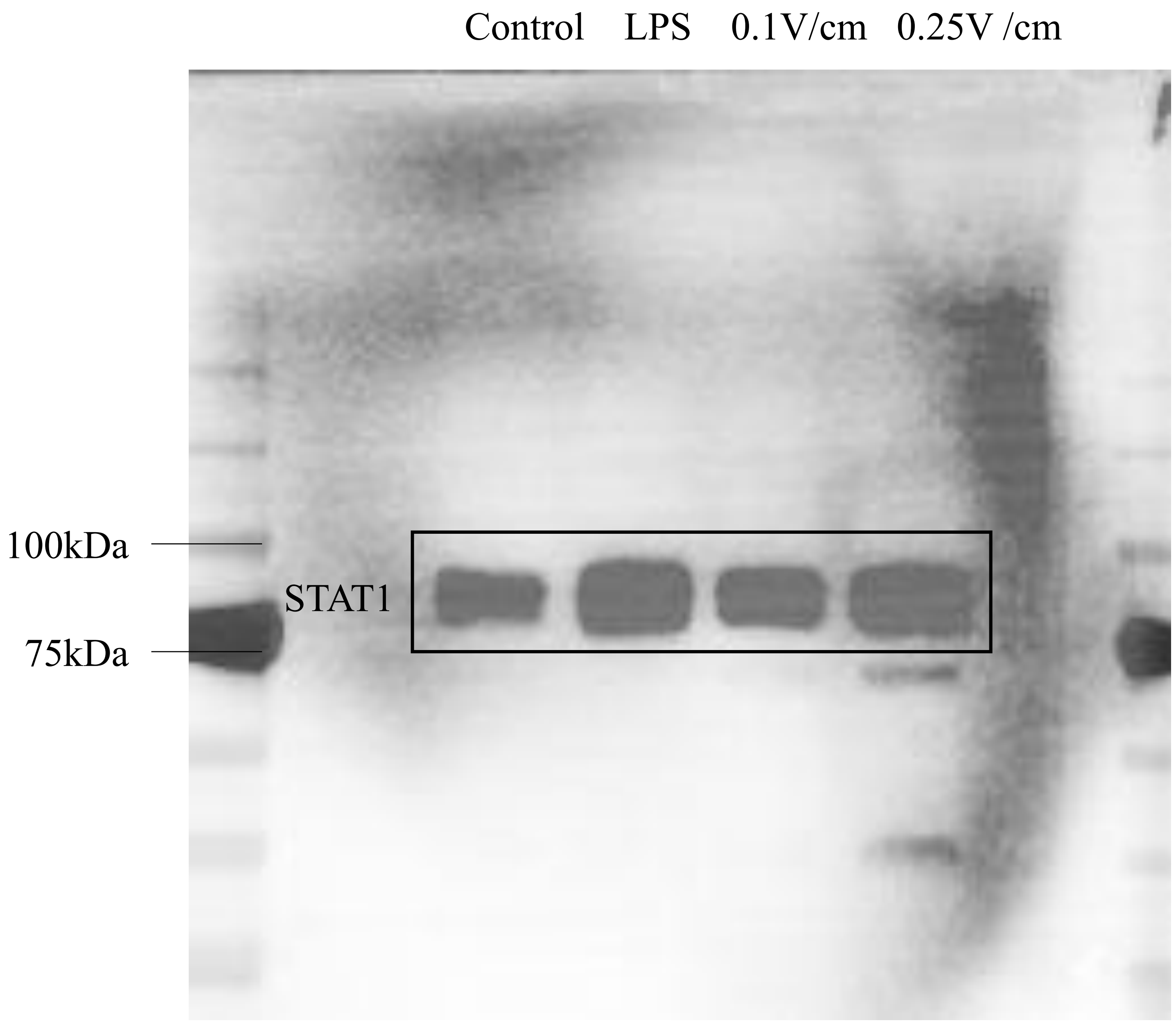

Control
LPS
0.1V/cm
0.25V /cm
100kDa
STAT1
75kDa

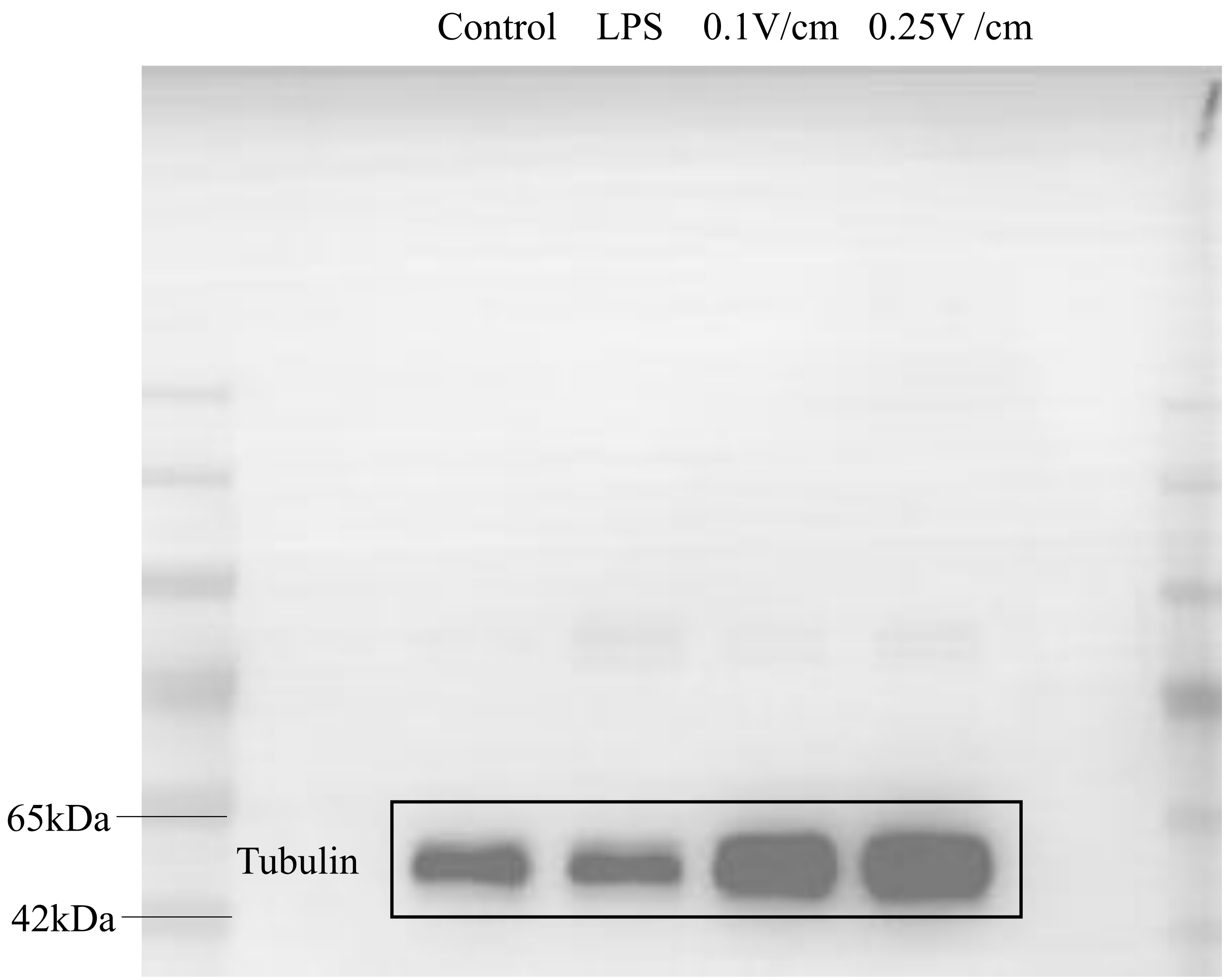
Control
LPS
0.1V/cm
0.25V /cm
65kDa
Tubulin
42kDa

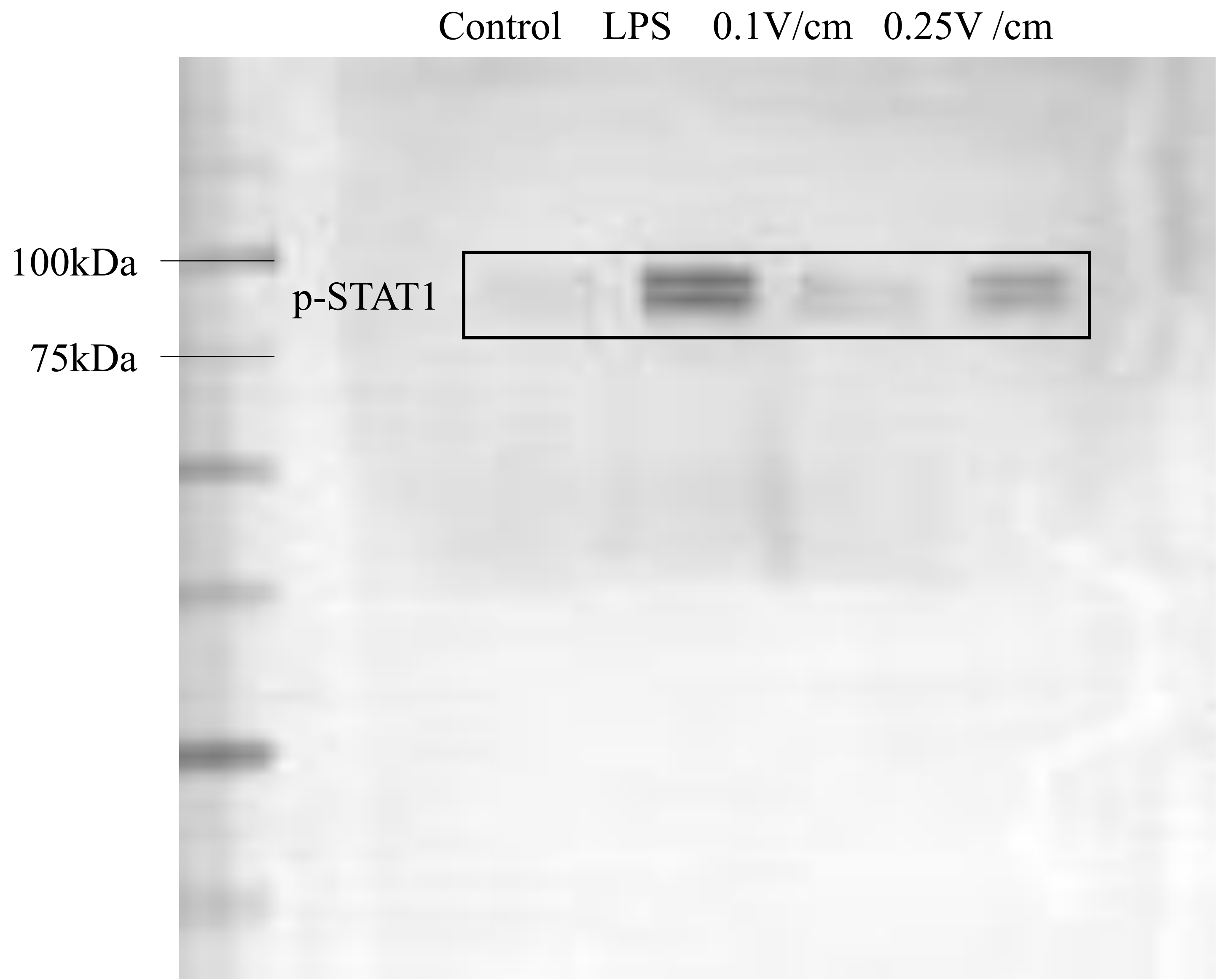
Control
LPS
0.1V/cm
0.25V /cm
100kDa
75kDa
p-STAT1

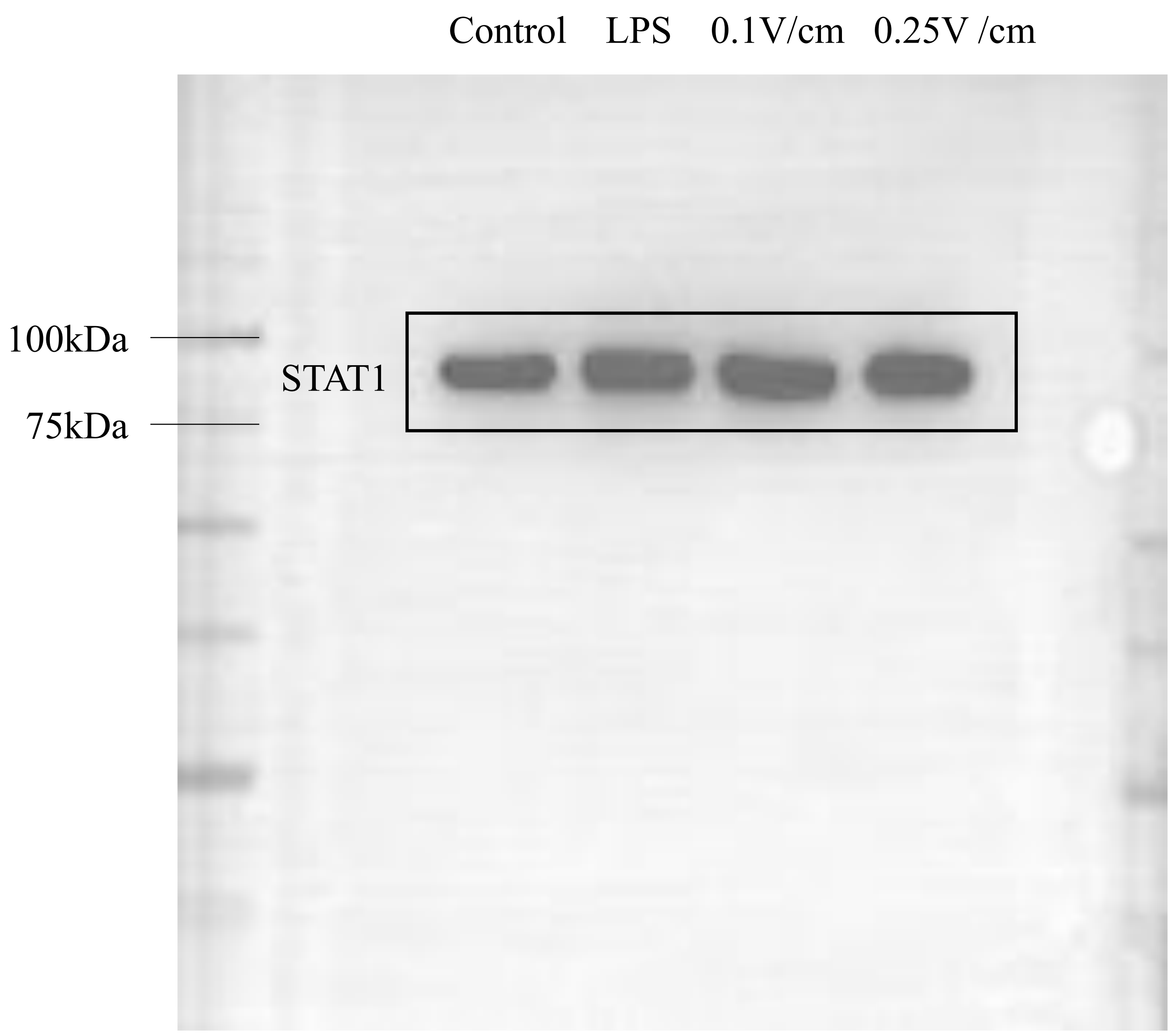
Control
LPS
0.1V/cm
0.25V /cm
100kDa
STAT1
75kDa

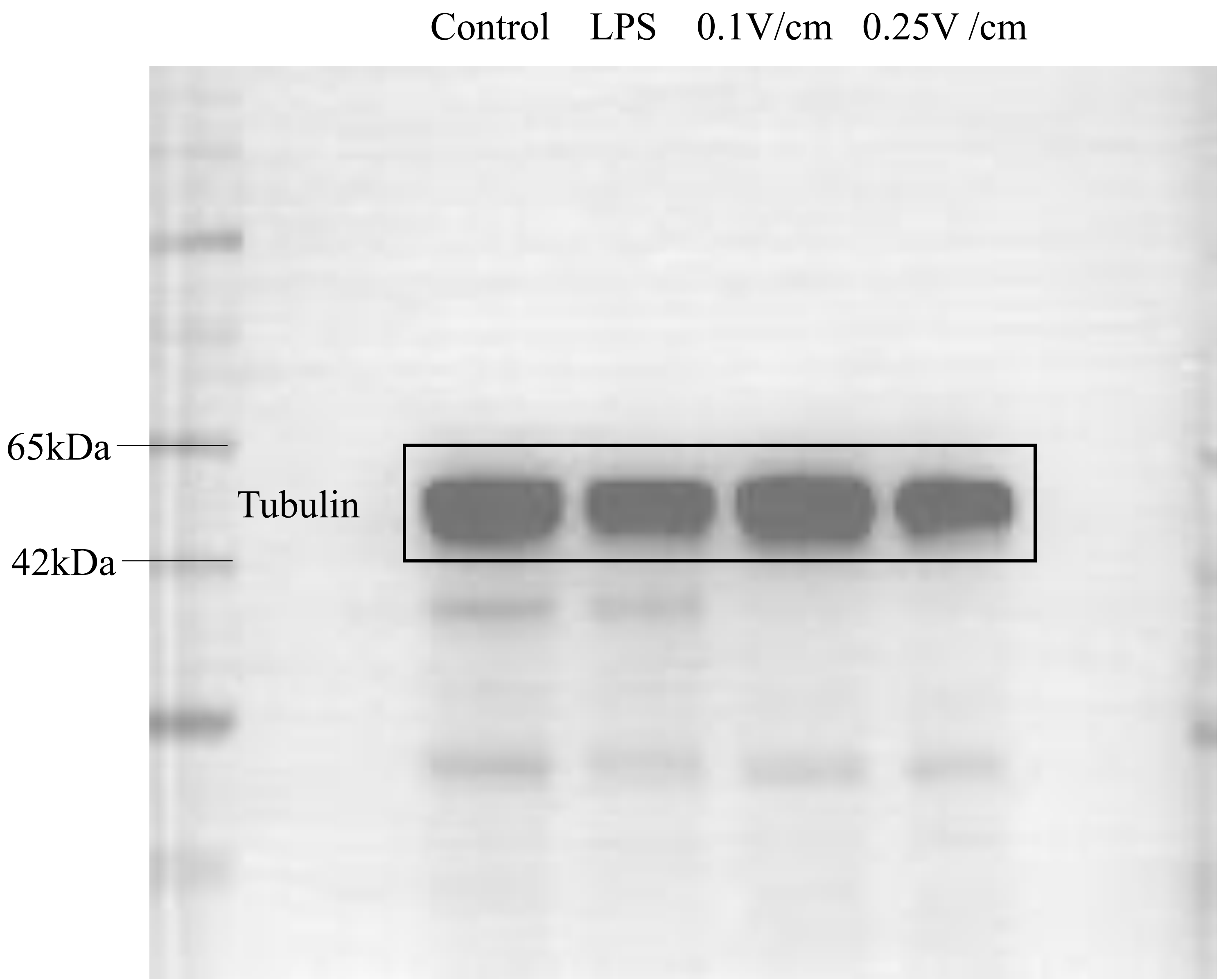
Control
LPS
0.1V/cm
0.25V /cm
65kDa
Tubulin
42kDa

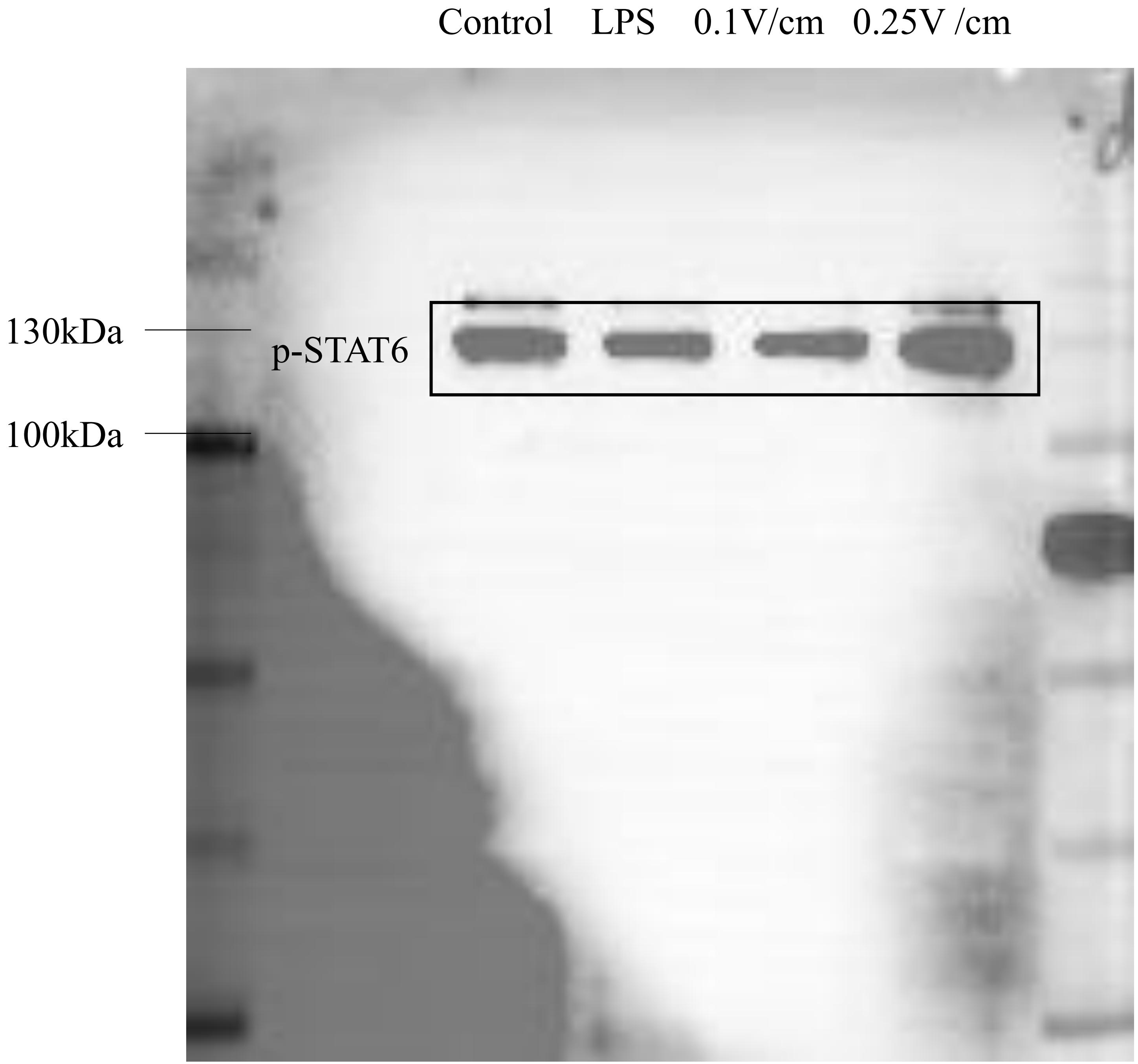
Control LPS 0.1V/cm 0.25V /cm
130kDa
100kDa
p-STAT6

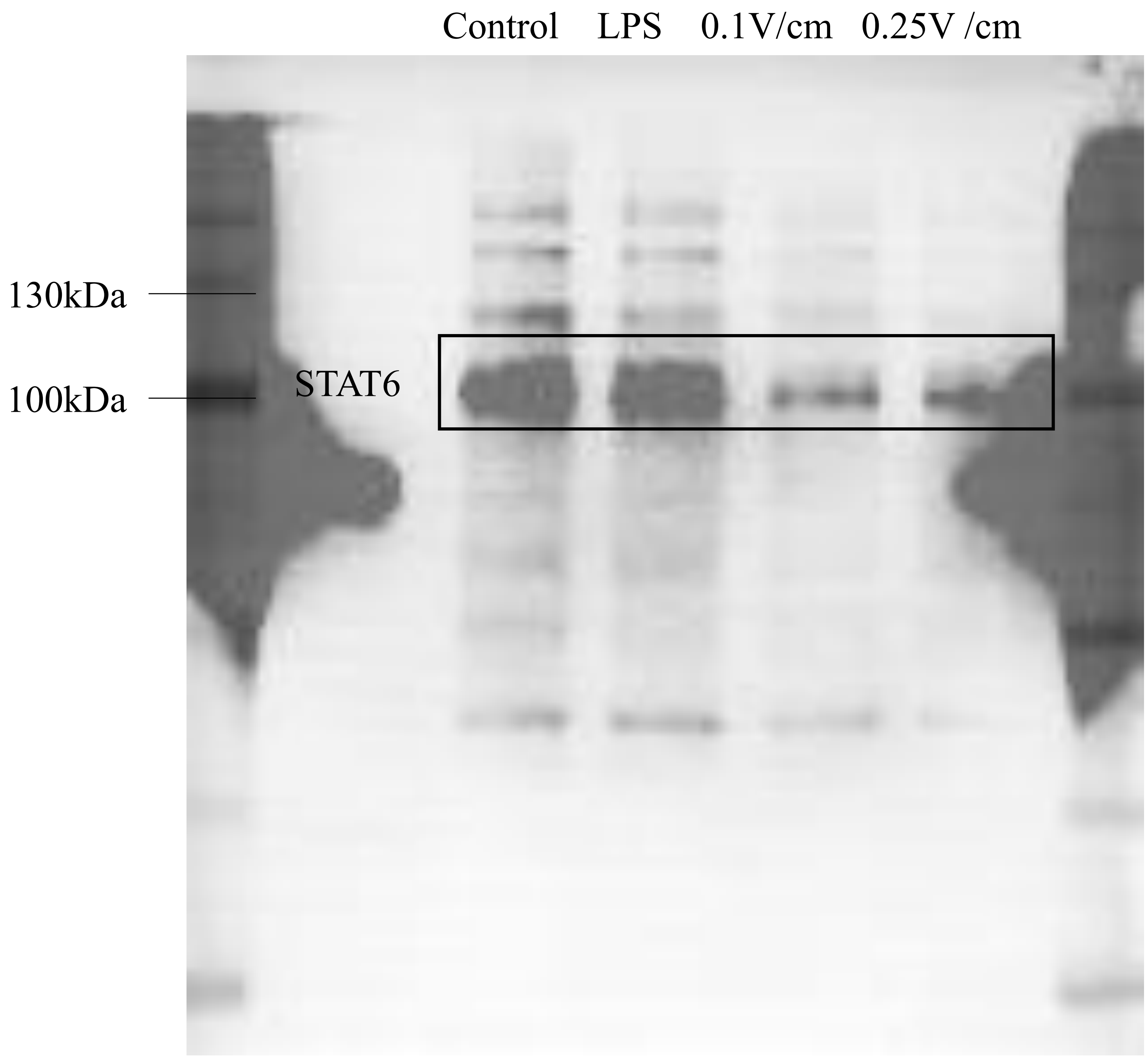
Control
LPS
0.1V/cm
0.25V /cm
130kDa
100kDa
STAT6

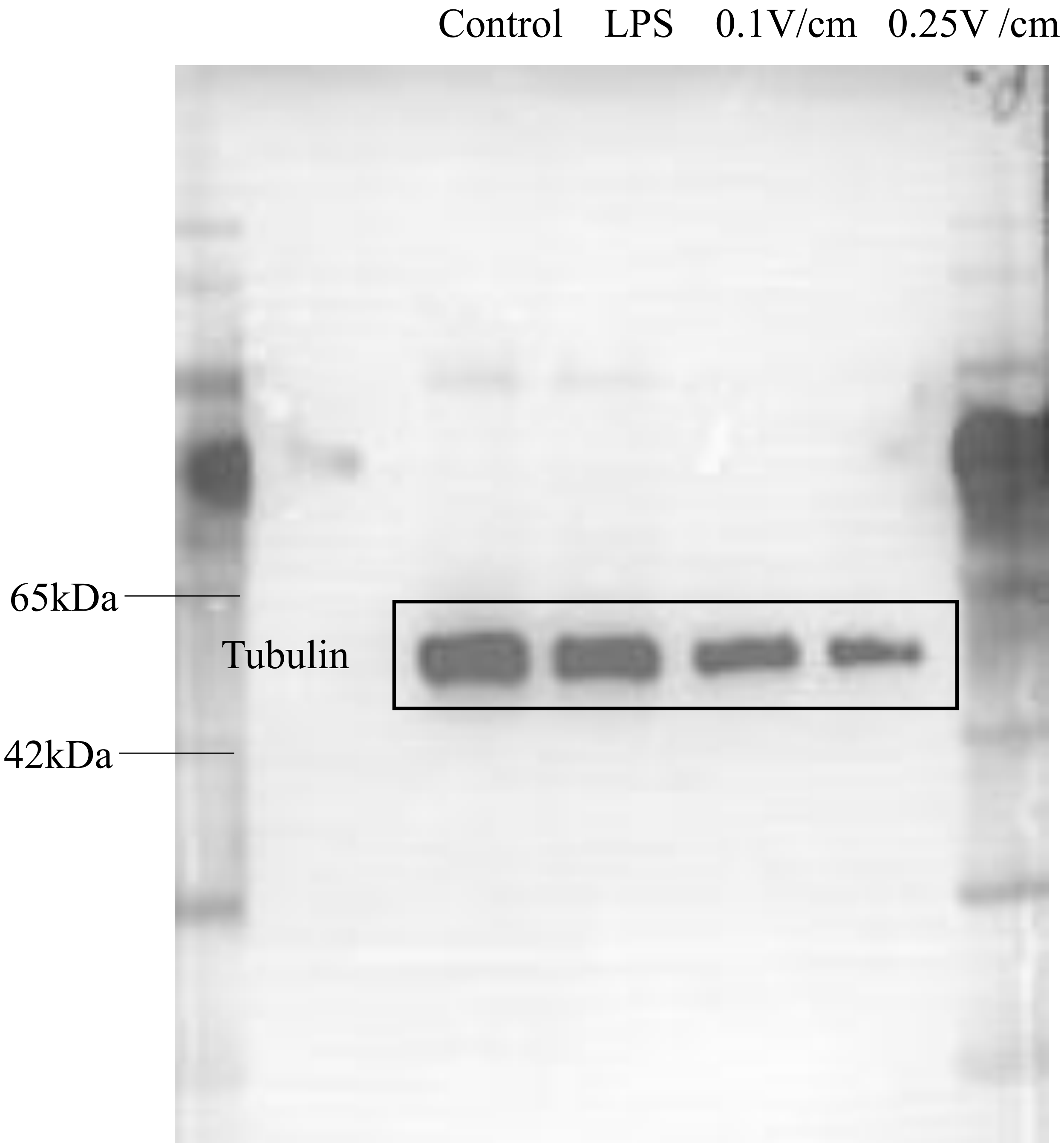
Control LPS 0.1V/cm 0.25V /cm
65kDa
Tubulin
42kDa

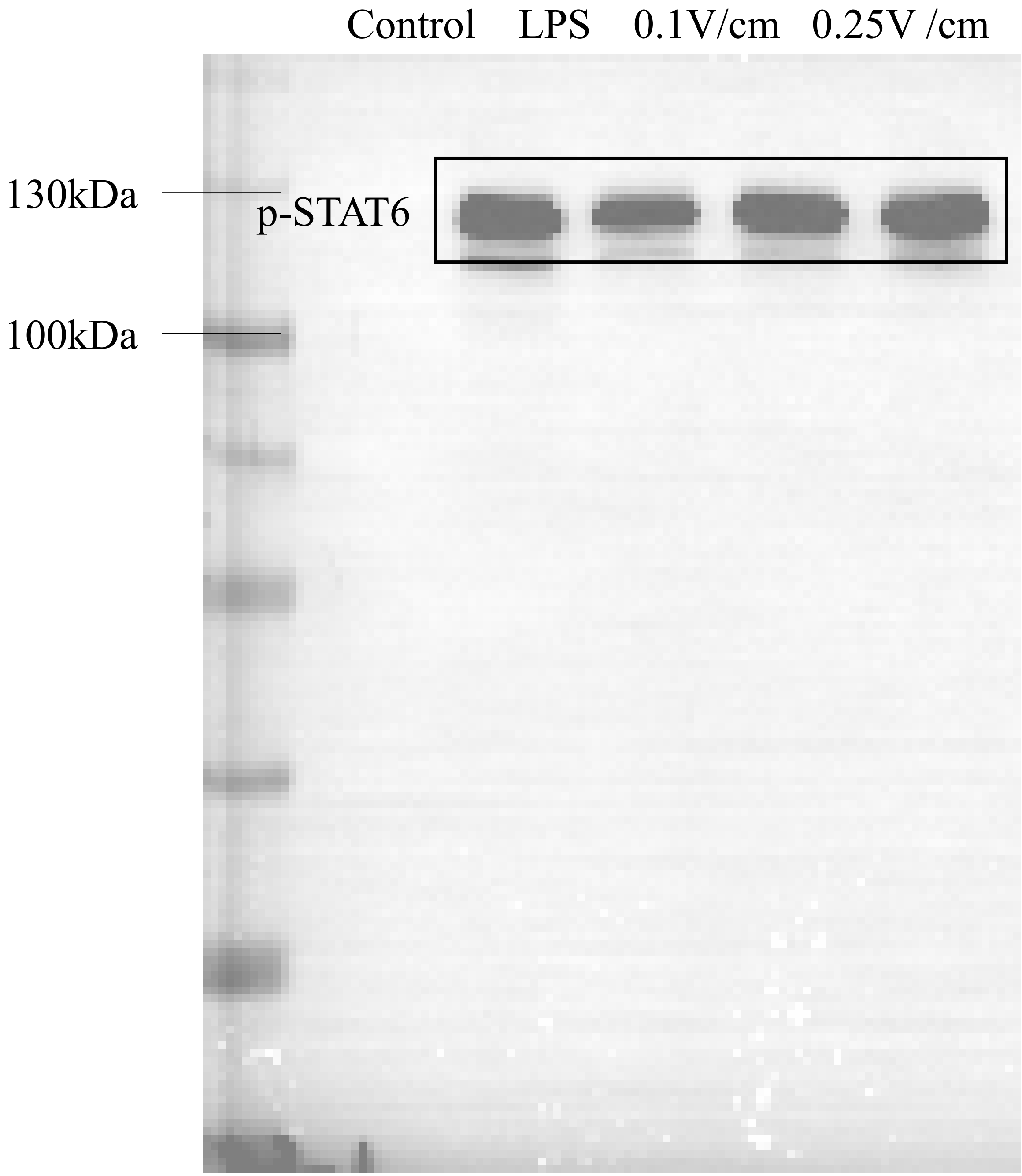
Control
LPS
0.1V/cm
0.25V /cm
130kDa
p-STAT6
100kDa

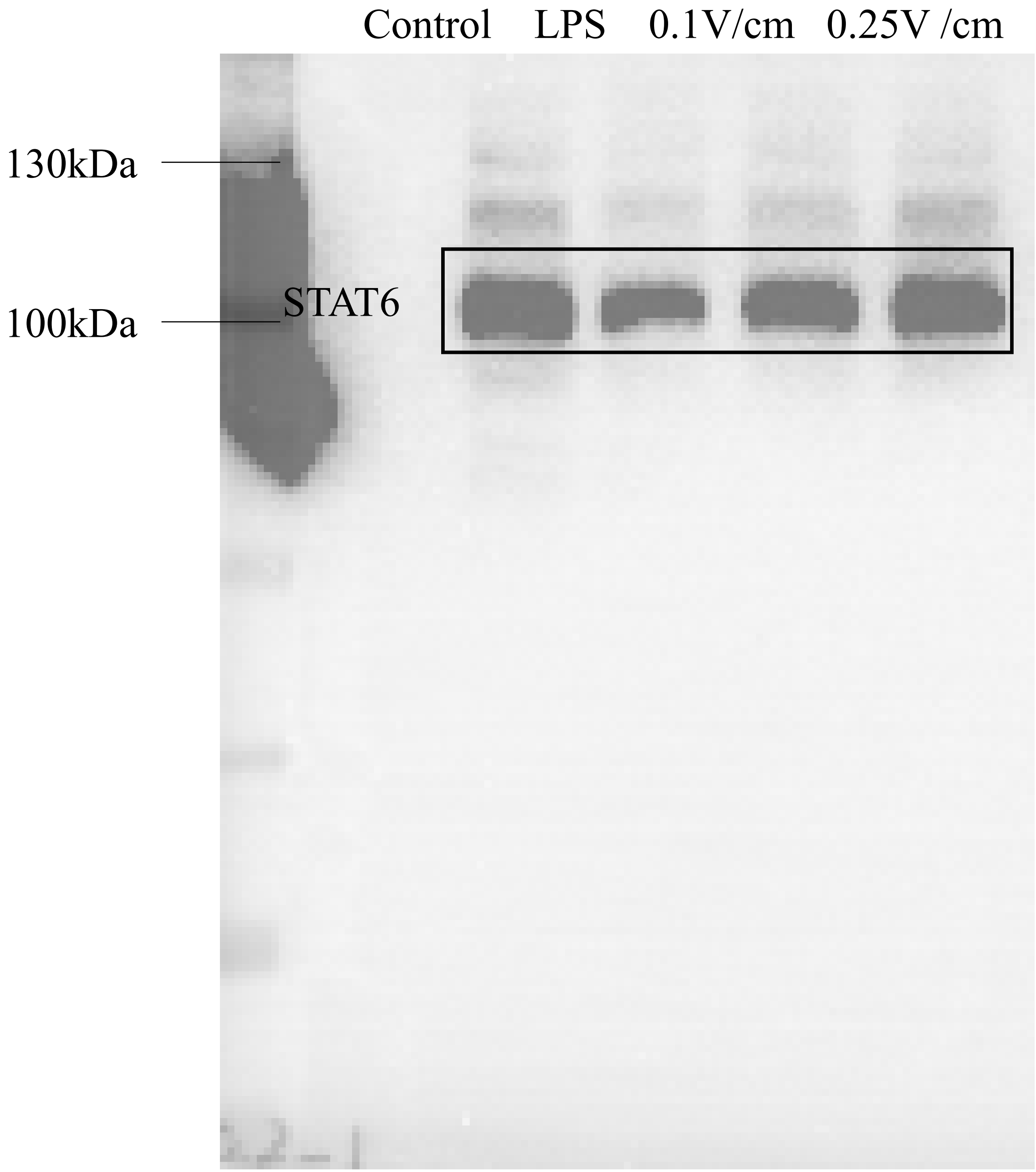
Control
LPS
0.1V/cm
0.25V /cm
130kDa
100kDa
STAT6

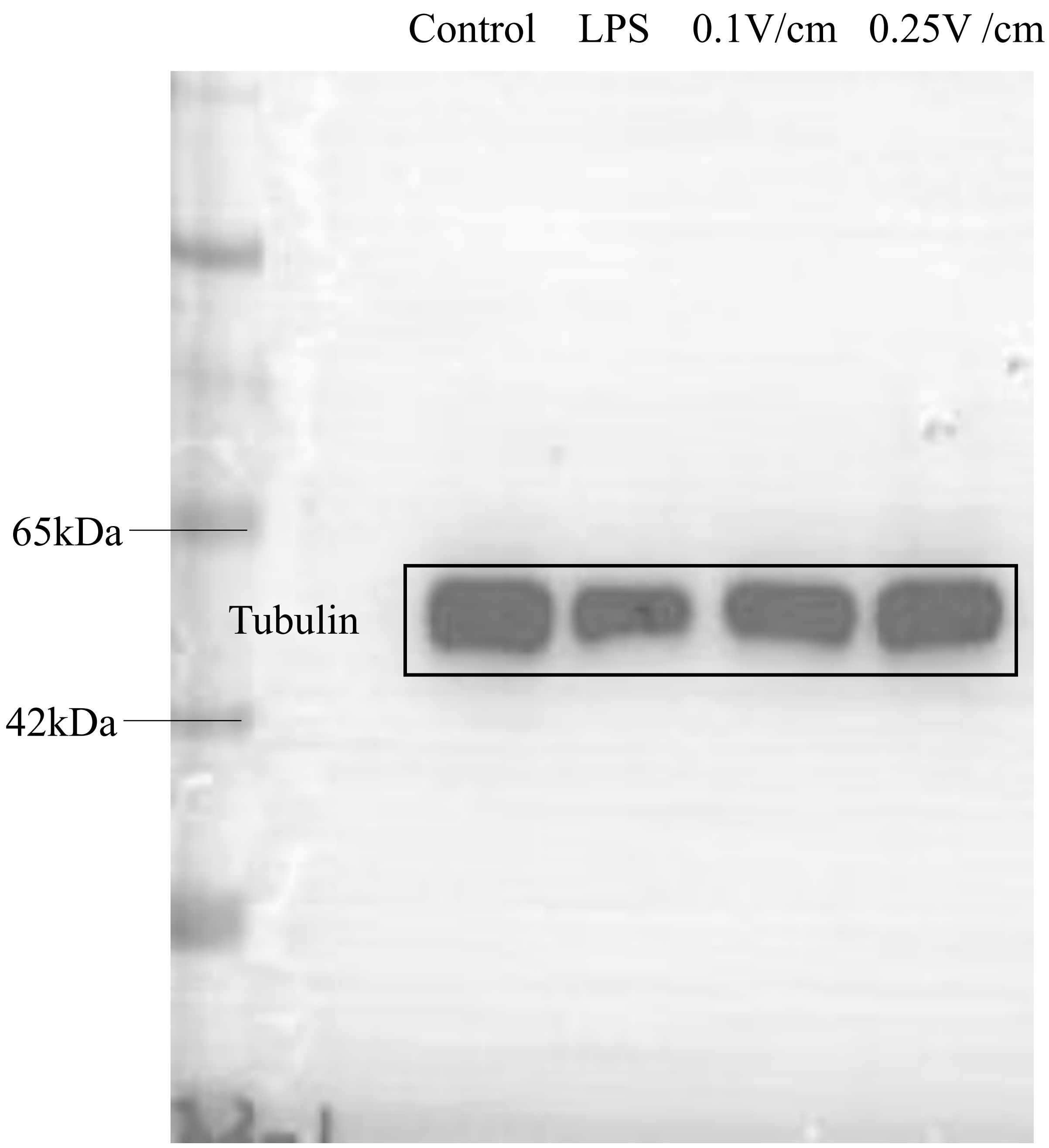
Control
LPS
0.1V/cm
0.25V /cm
65kDa
Tubulin
42kDa

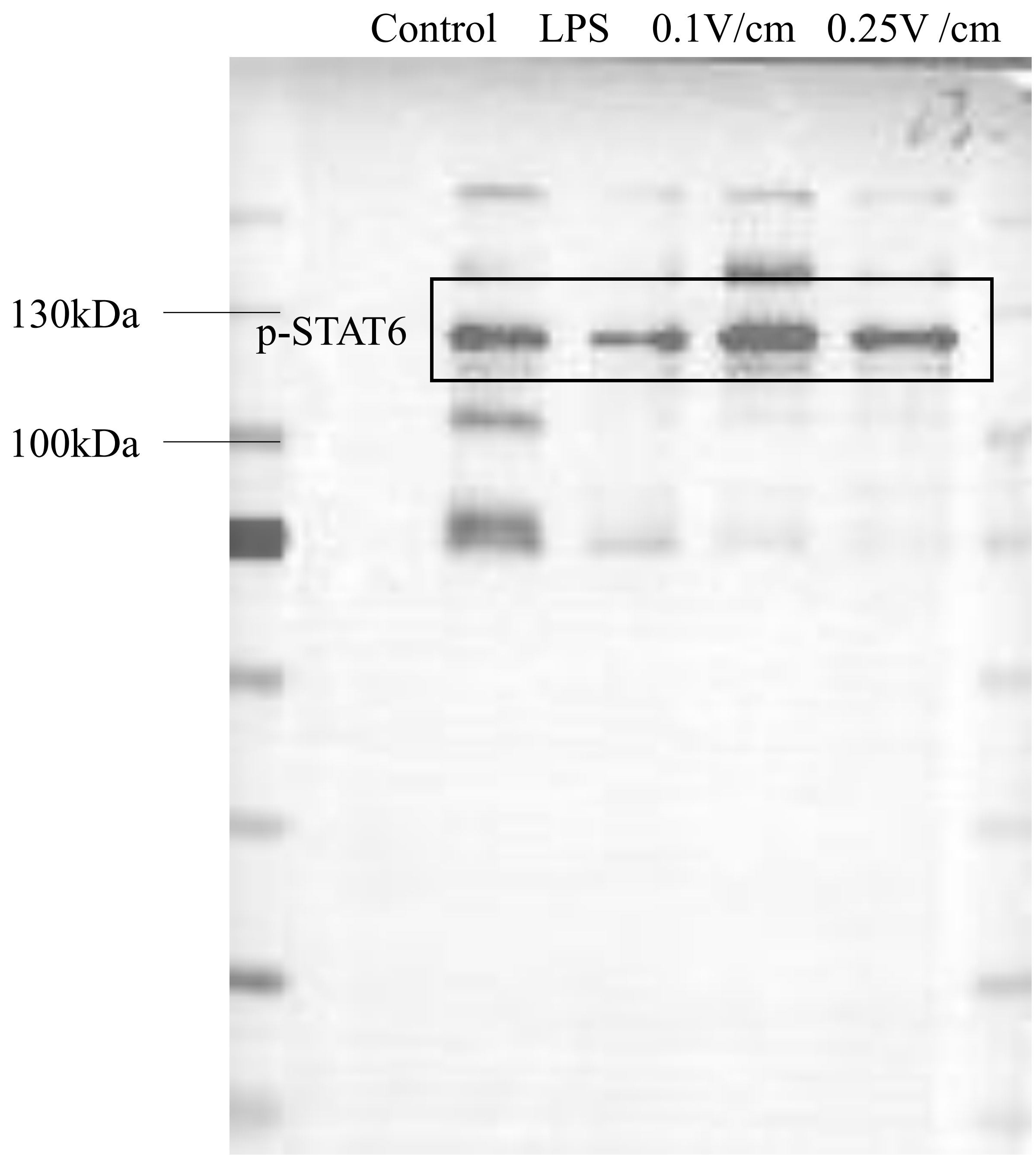

Control
LPS
0.1V/cm
0.25V /cm
130kDa
p-STAT6
100kDa

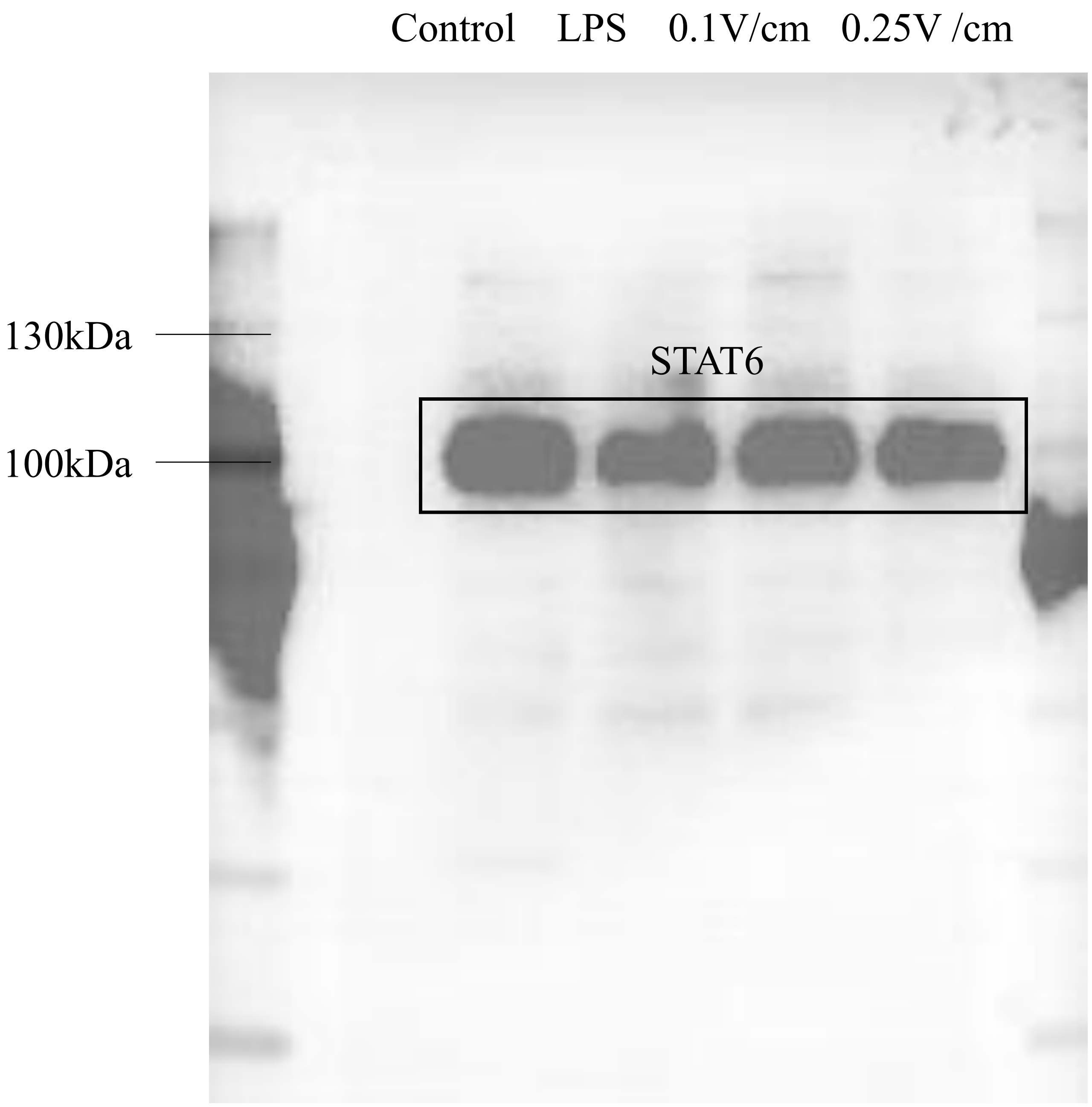
Control LPS 0.1V/cm 0.25V /cm
130kDa
100kDa
STAT6

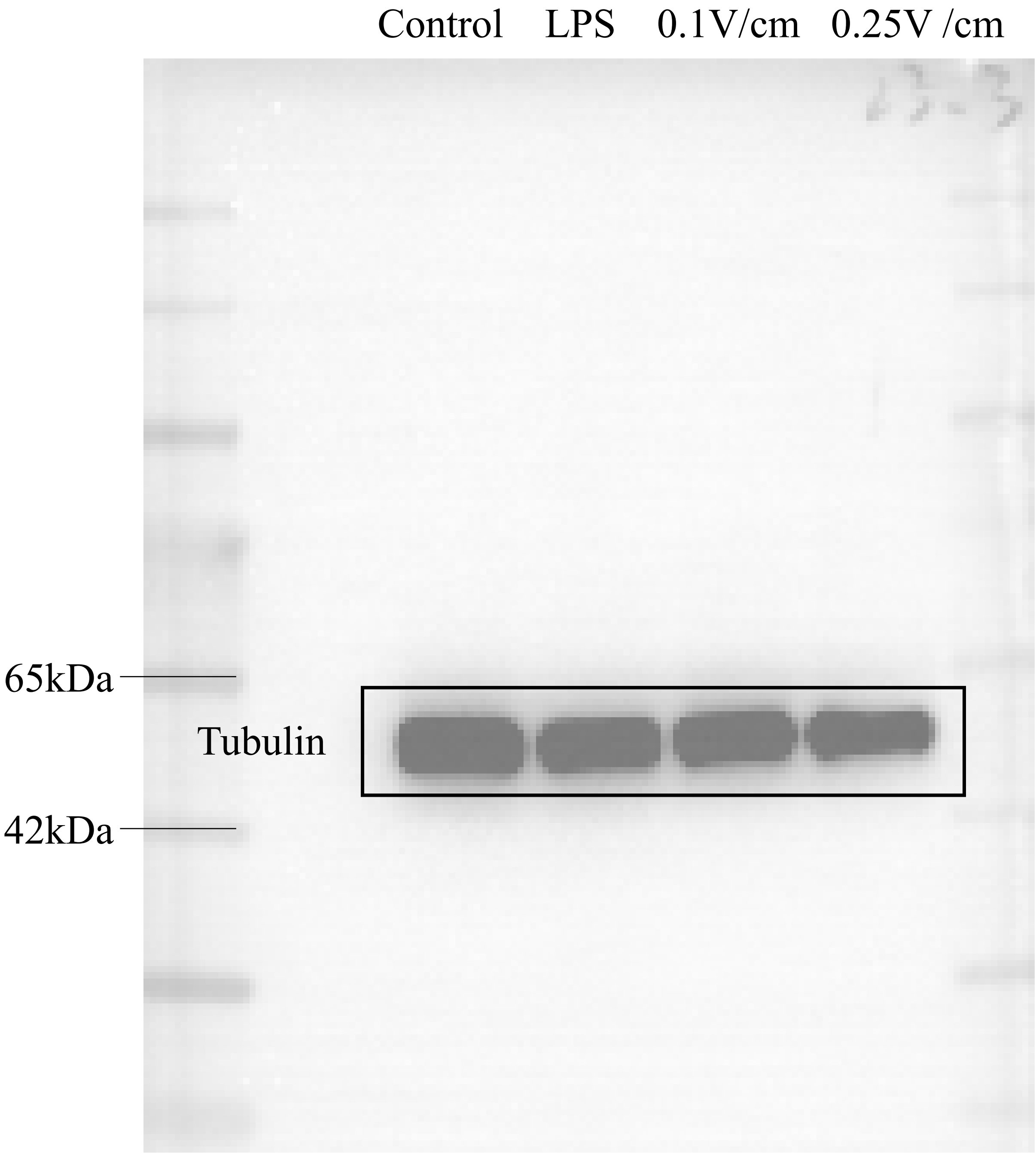
Control
LPS
0.1V/cm
0.25V /cm
65kDa
Tubulin
42kDa

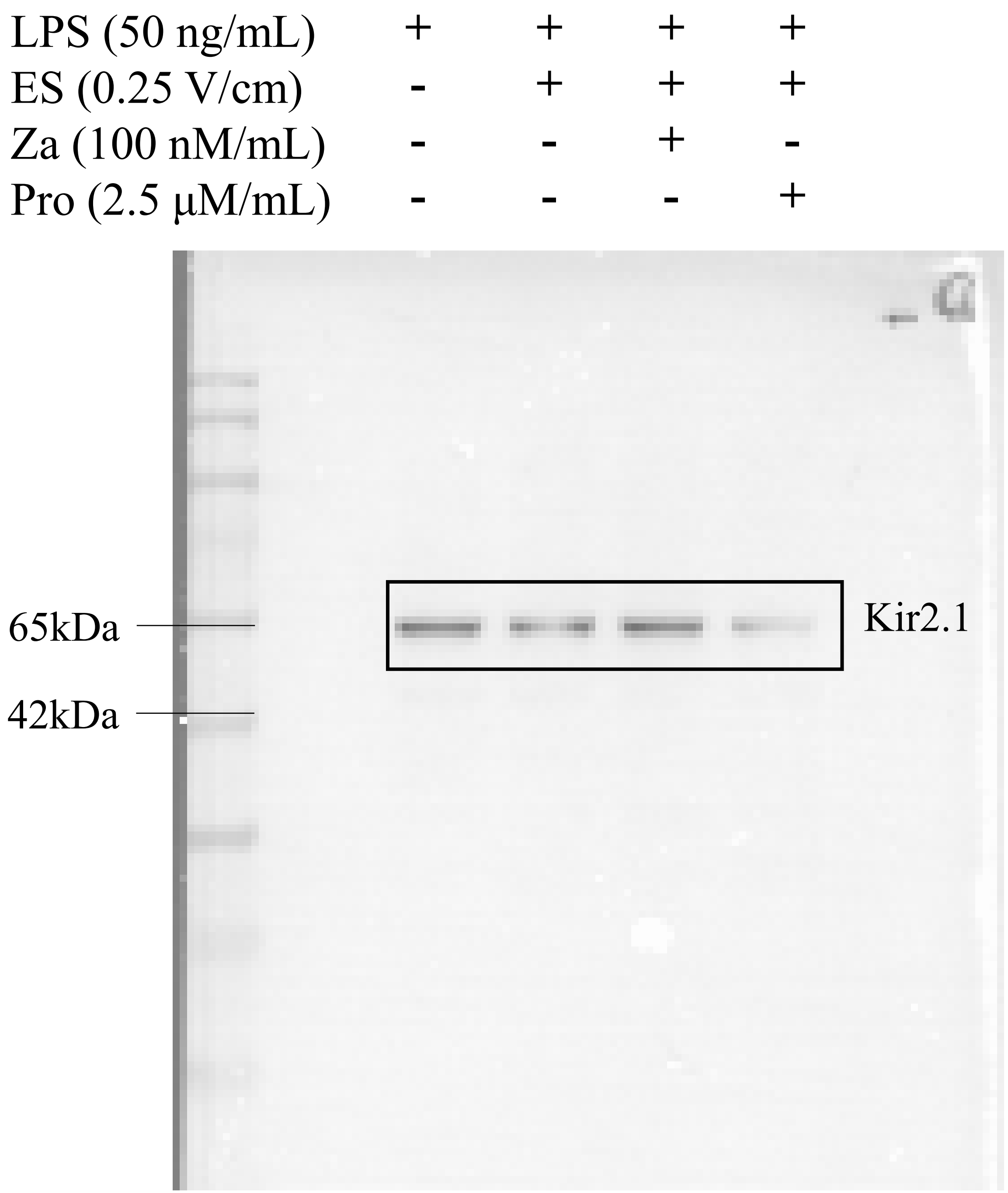
LPS (50 ng/mL) + + + +
ES (0.25 V/cm) - + + +
Za (100 nM/mL) - - + -
Pro (2.5 μM/mL) - - - +
65kDa
42kDa
Kir2.1

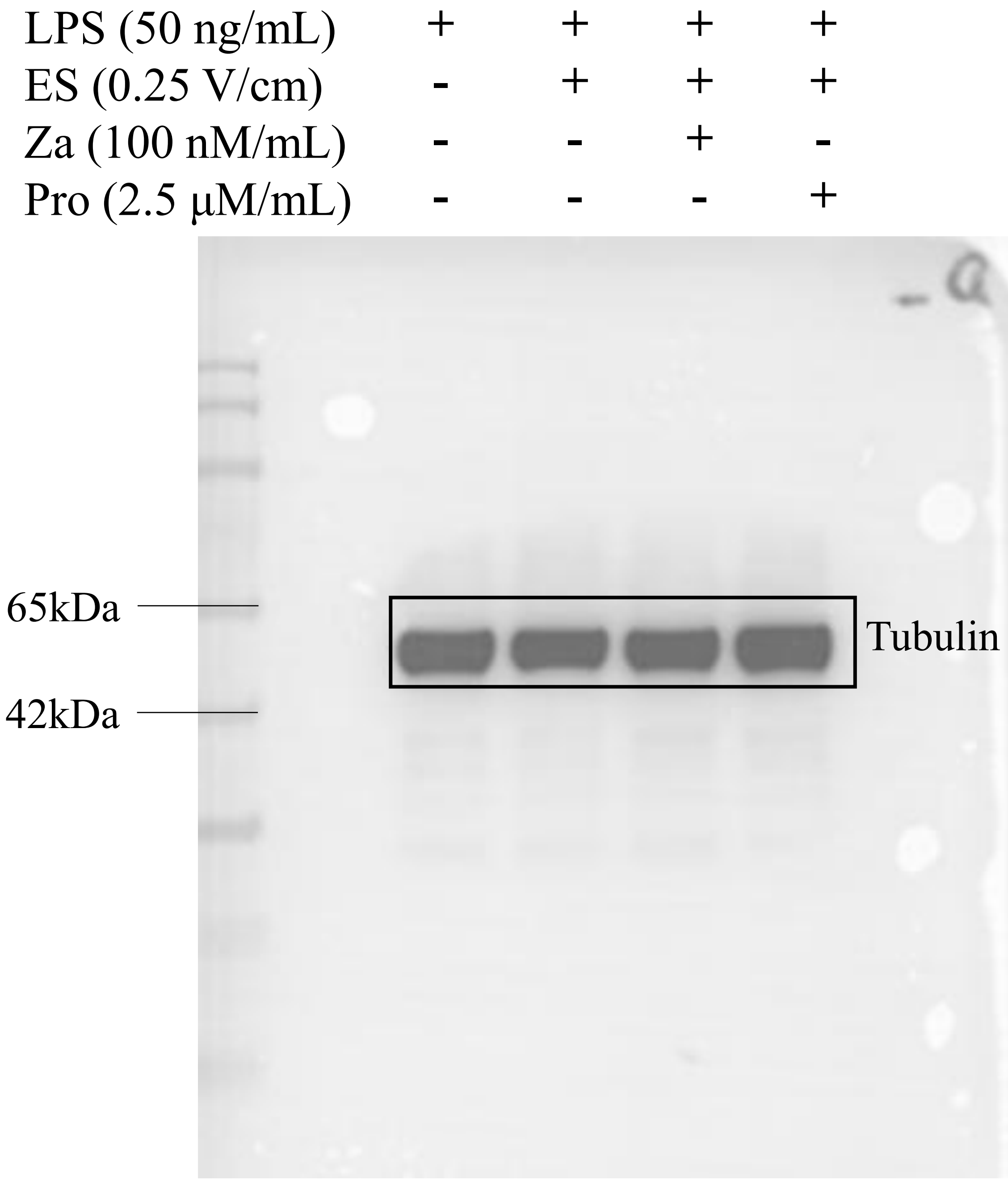
LPS (50 ng/mL) + + + +
ES (0.25 V/cm) - + + +
Za (100 nM/mL) - - + -
Pro (2.5 μM/mL) - - - +
65kDa
42kDa
Tubulin

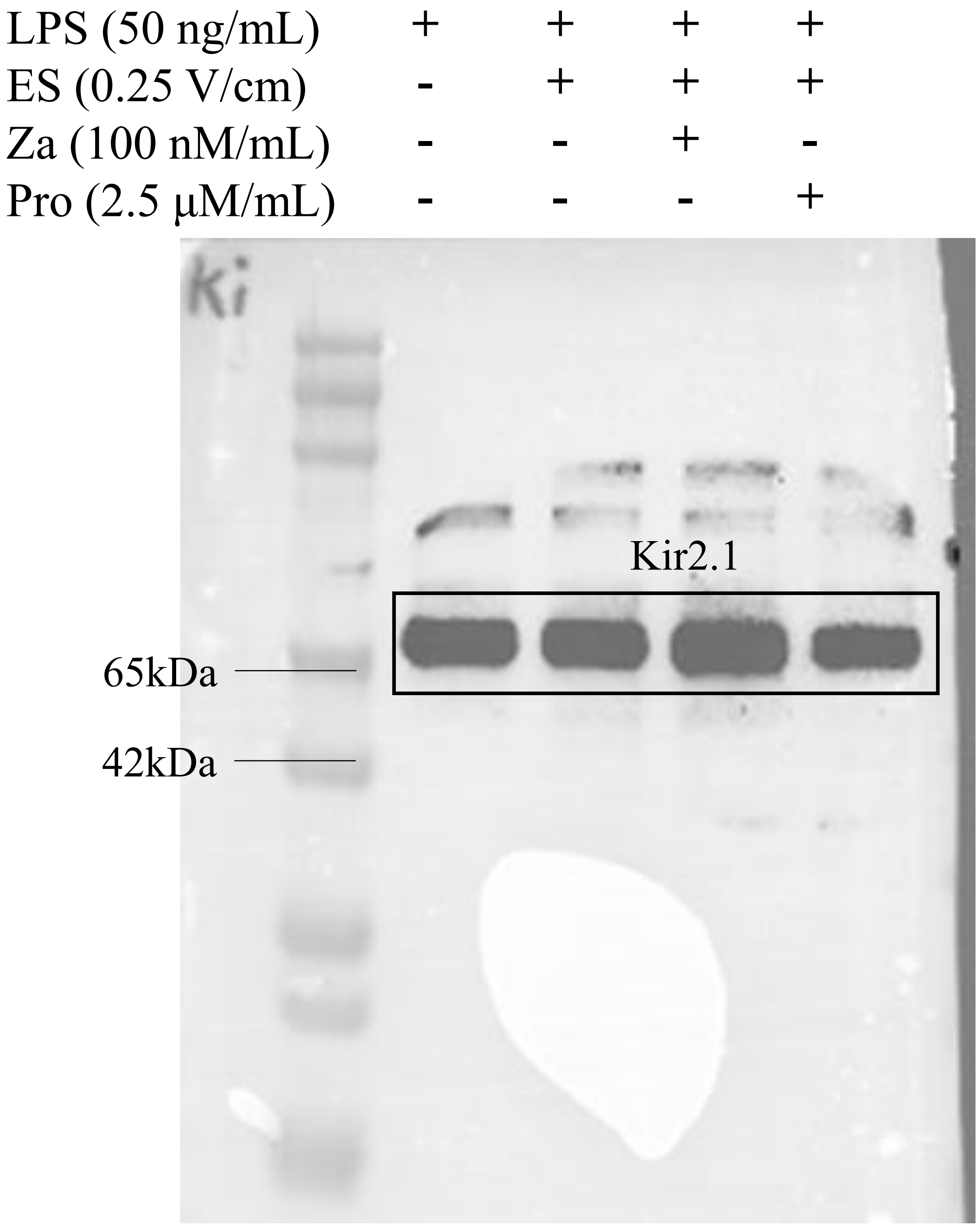

LPS (50 ng/mL) + + + +
ES (0.25 V/cm) - + + +
Za (100 nM/mL) - - + -
Pro (2.5 μM/mL) - - - +
Kir2.1
65kDa
42kDa

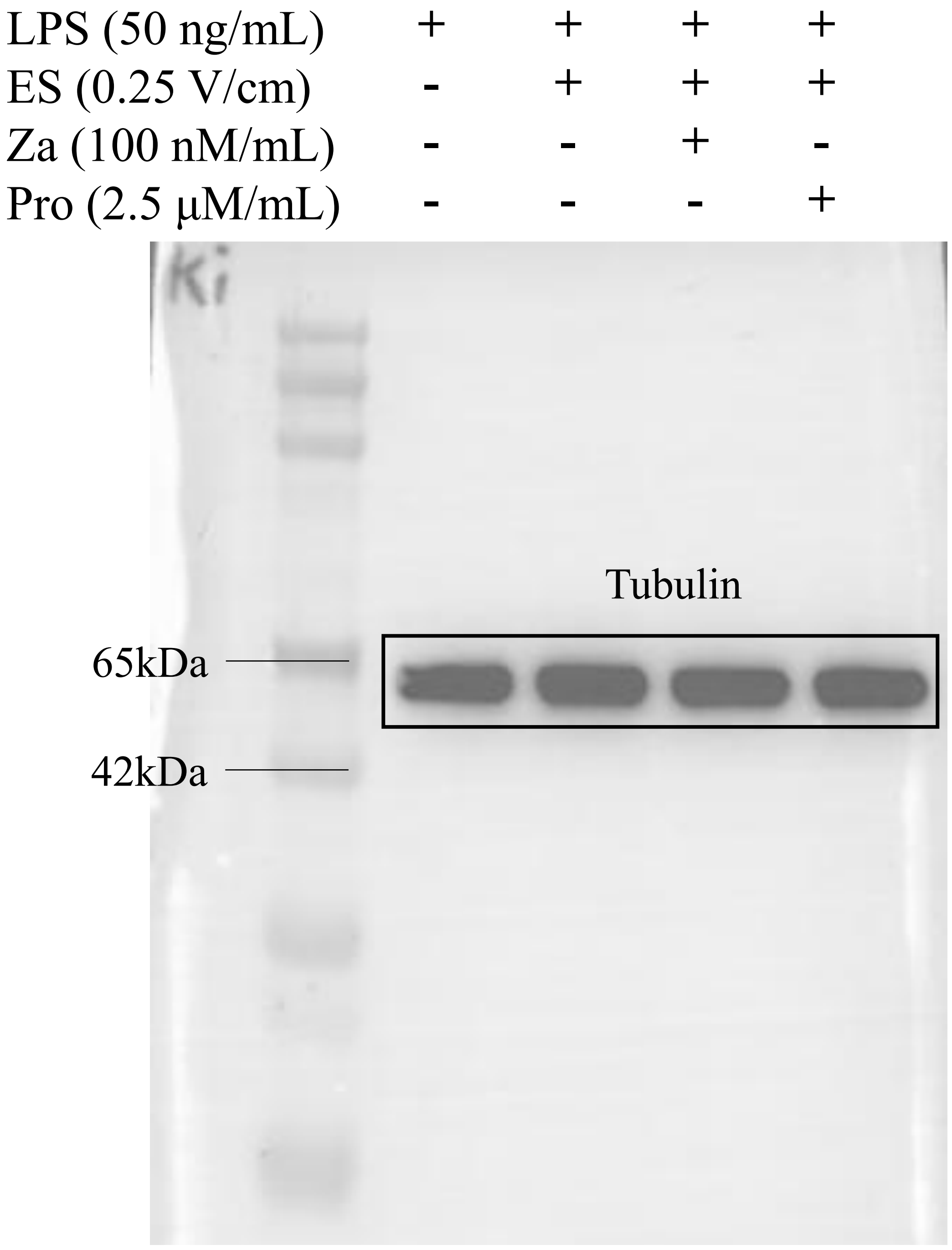
LPS (50 ng/mL) + + + +
ES (0.25 V/cm) - + + +
Za (100 nM/mL) - - + -
Pro (2.5 μM/mL) - - - +
Ki
Tubulin
65kDa
42kDa

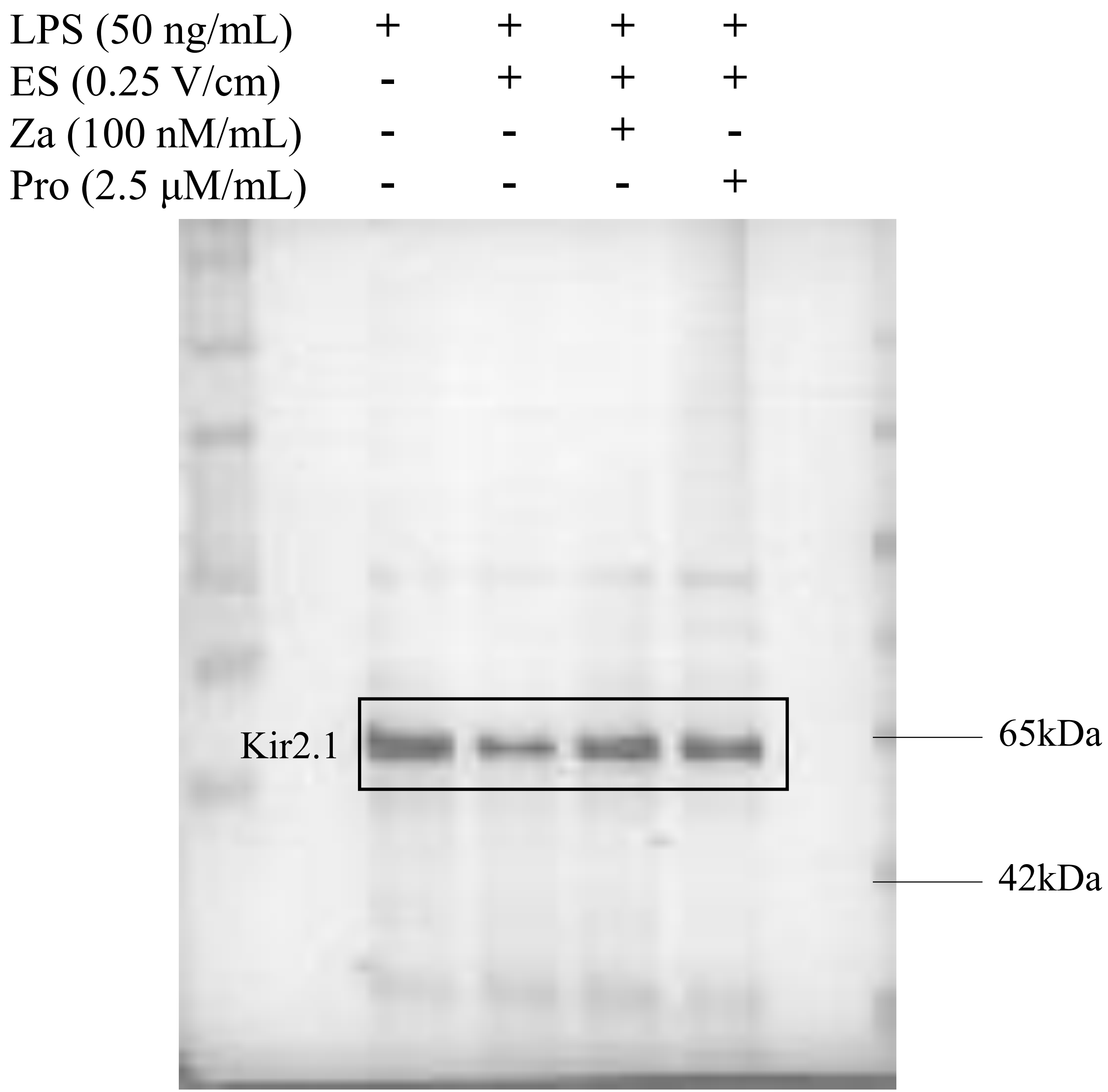
LPS (50 ng/mL) + + + +
ES (0.25 V/cm) - + + +
Za (100 nM/mL) - - + -
Pro (2.5 μM/mL) - - - +
Kir2.1
65kDa
42kDa

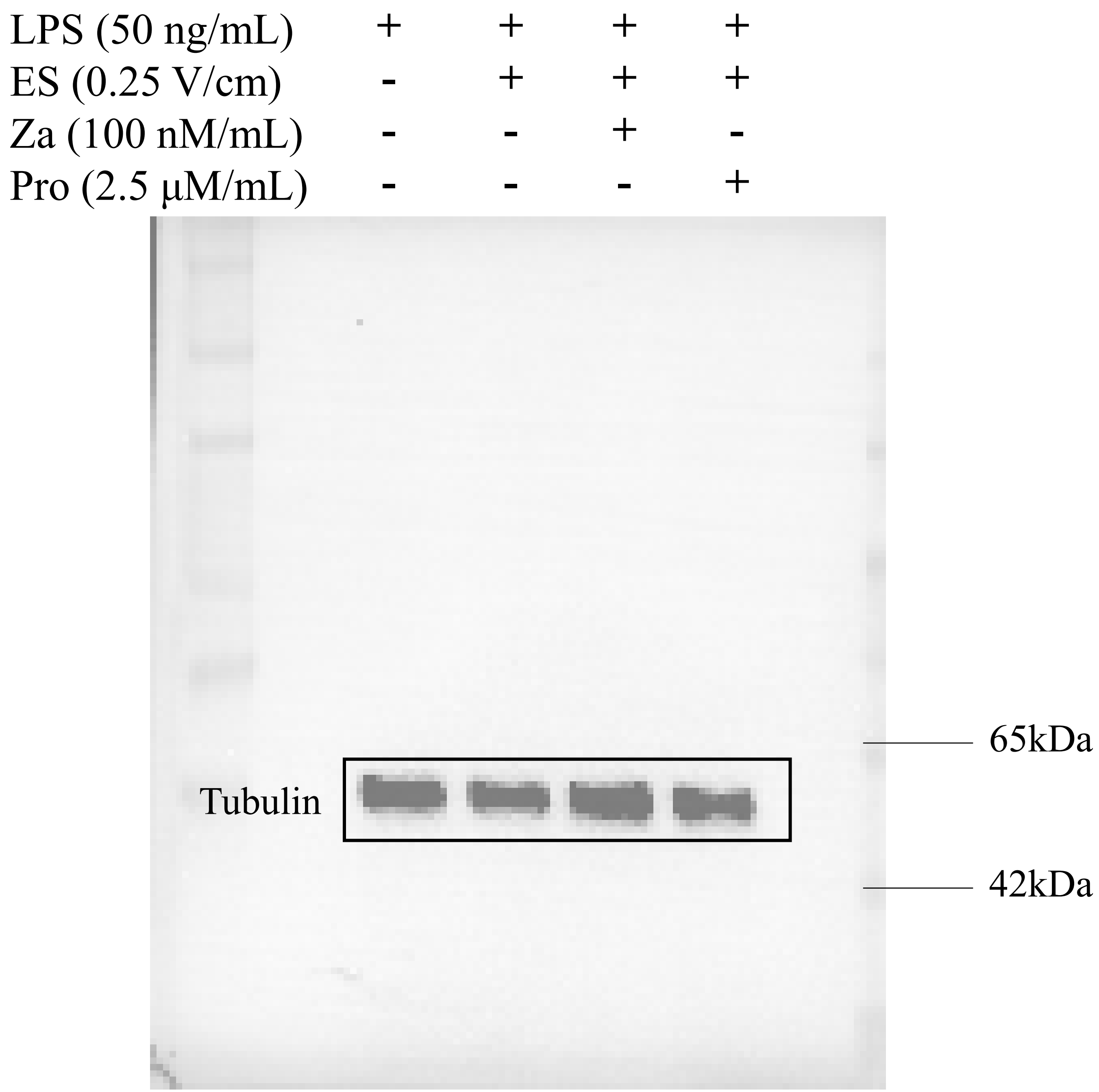
LPS (50 ng/mL) + + + +
ES (0.25 V/cm) - + + +
Za (100 nM/mL) - - + -
Pro (2.5 μM/mL) - - - +
Tubulin
65kDa
42kDa

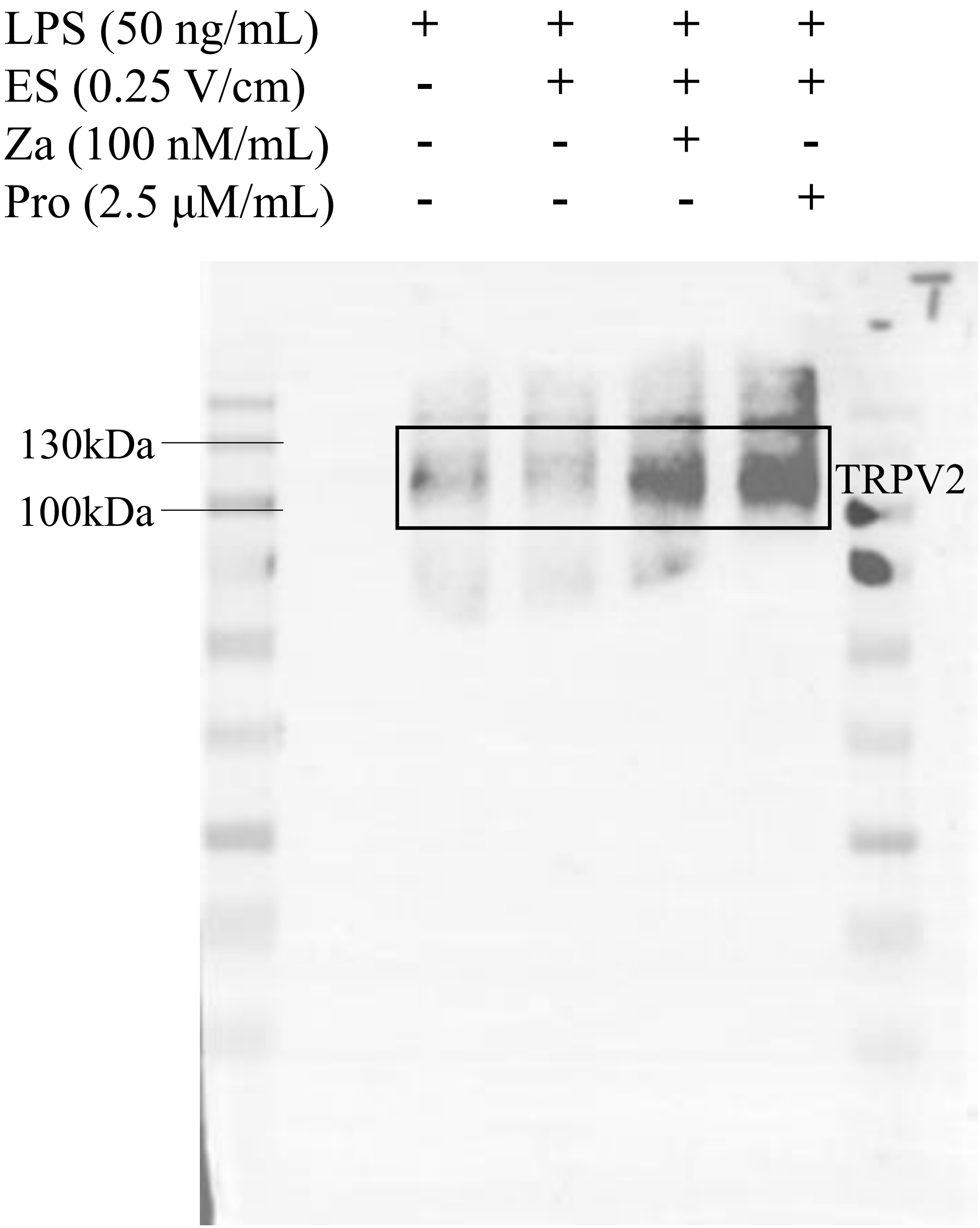

LPS (50 ng/mL) + + + +
ES (0.25 V/cm) - + + +
Za (100 nM/mL) - - + -
Pro (2.5 μM/mL) - - - +
130kDa
100kDa
TRPV2

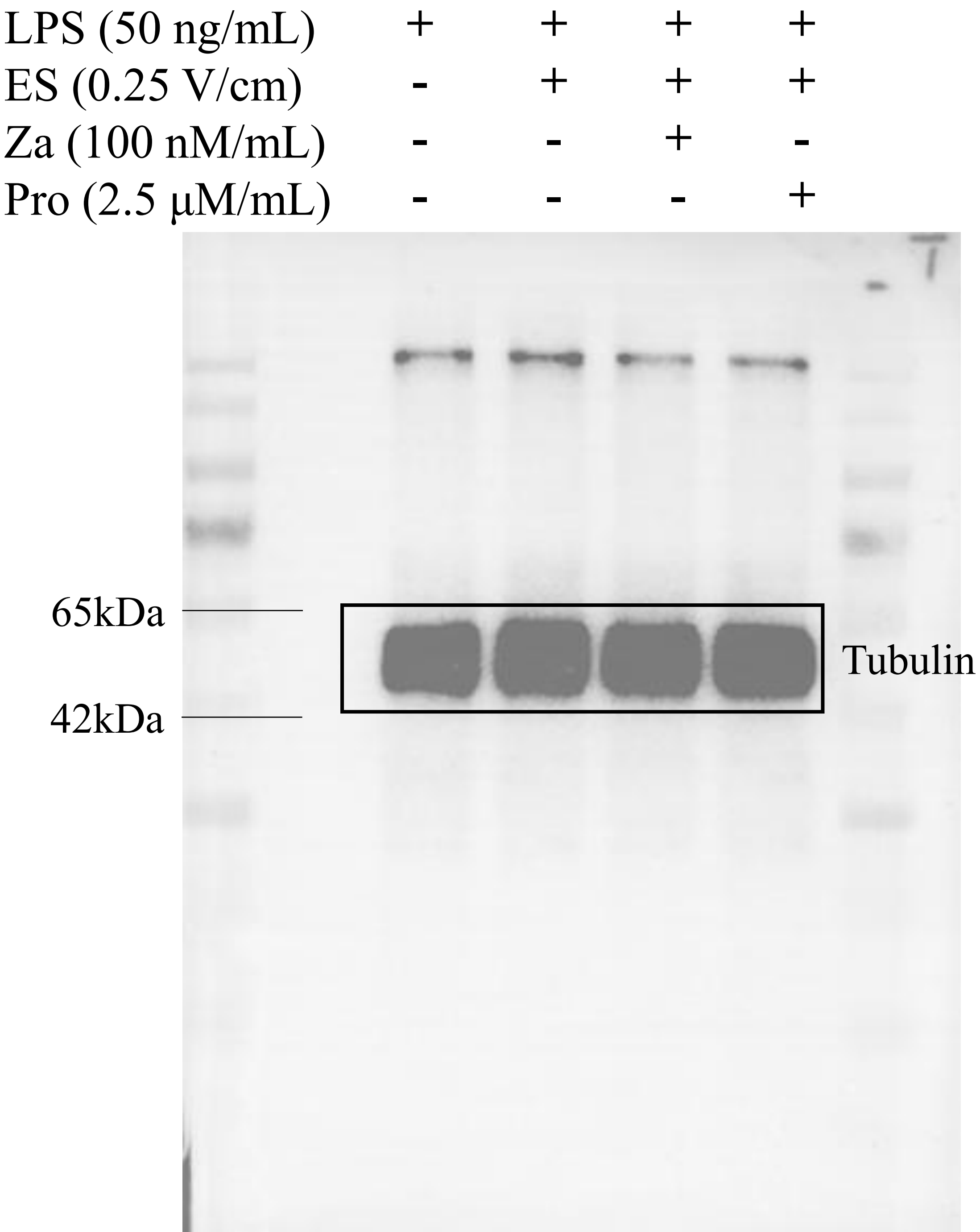
LPS (50 ng/mL) + + + +
ES (0.25 V/cm) - + + +
Za (100 nM/mL) - - + -
Pro (2.5 μM/mL) - - - +
65kDa
42kDa
Tubulin

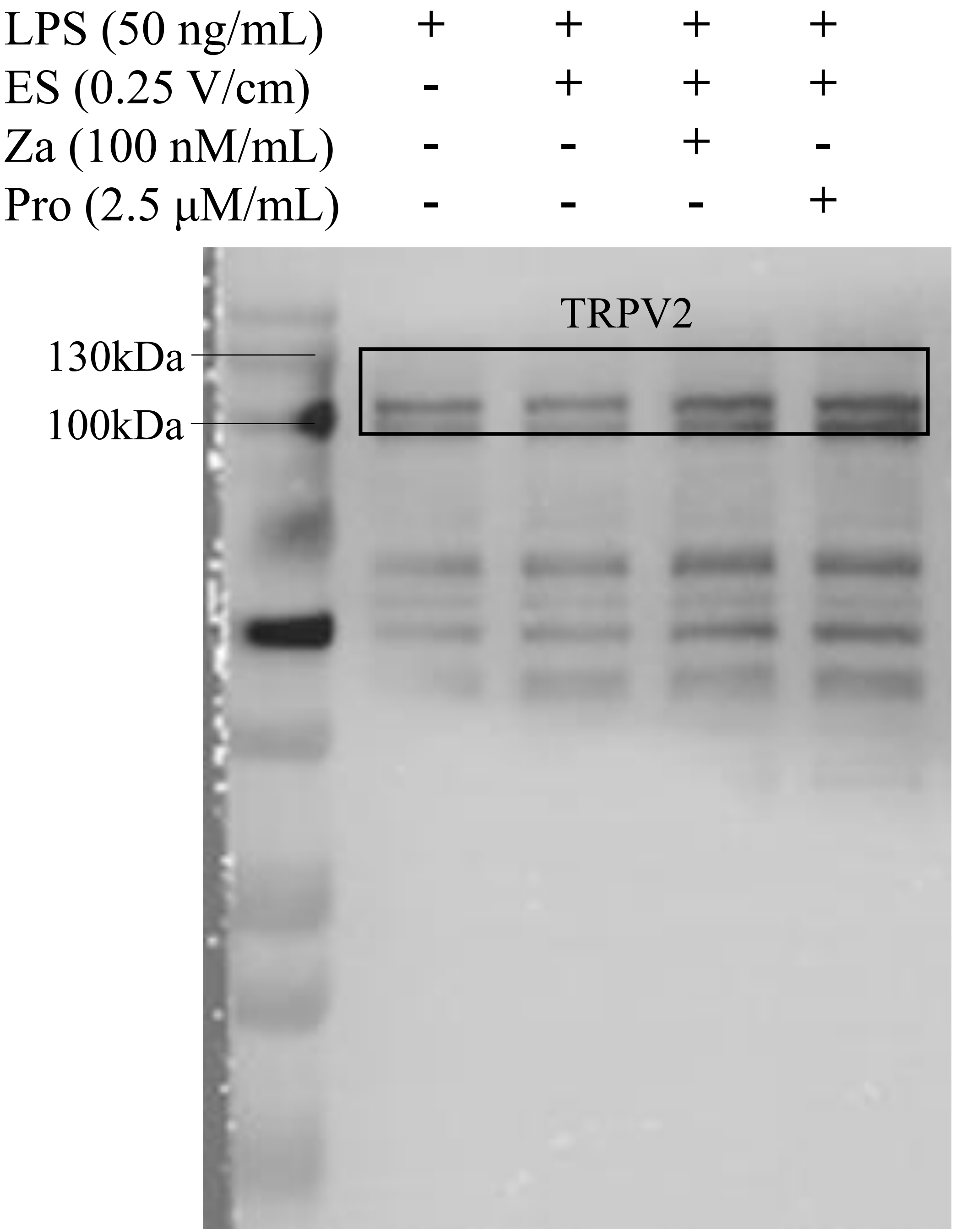
LPS (50 ng/mL) + + + +
ES (0.25 V/cm) - + + +
Za (100 nM/mL) - - + -
Pro (2.5 μM/mL) - - - +
TRPV2
130kDa
100kDa

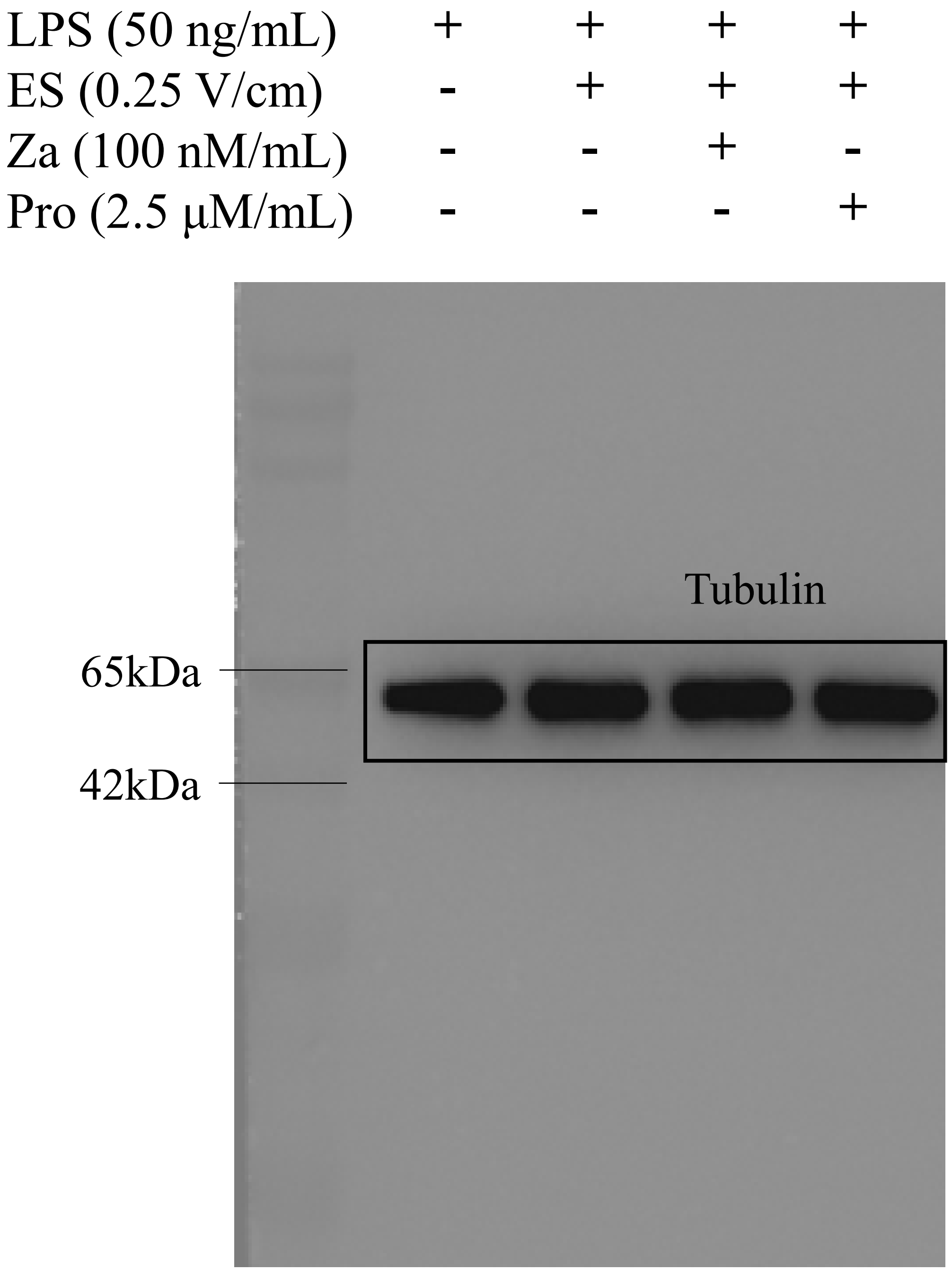
LPS (50 ng/mL) + + + +
ES (0.25 V/cm) - + + +
Za (100 nM/mL) - - + -
Pro (2.5 μM/mL) - - - +
Tubulin
65kDa
42kDa

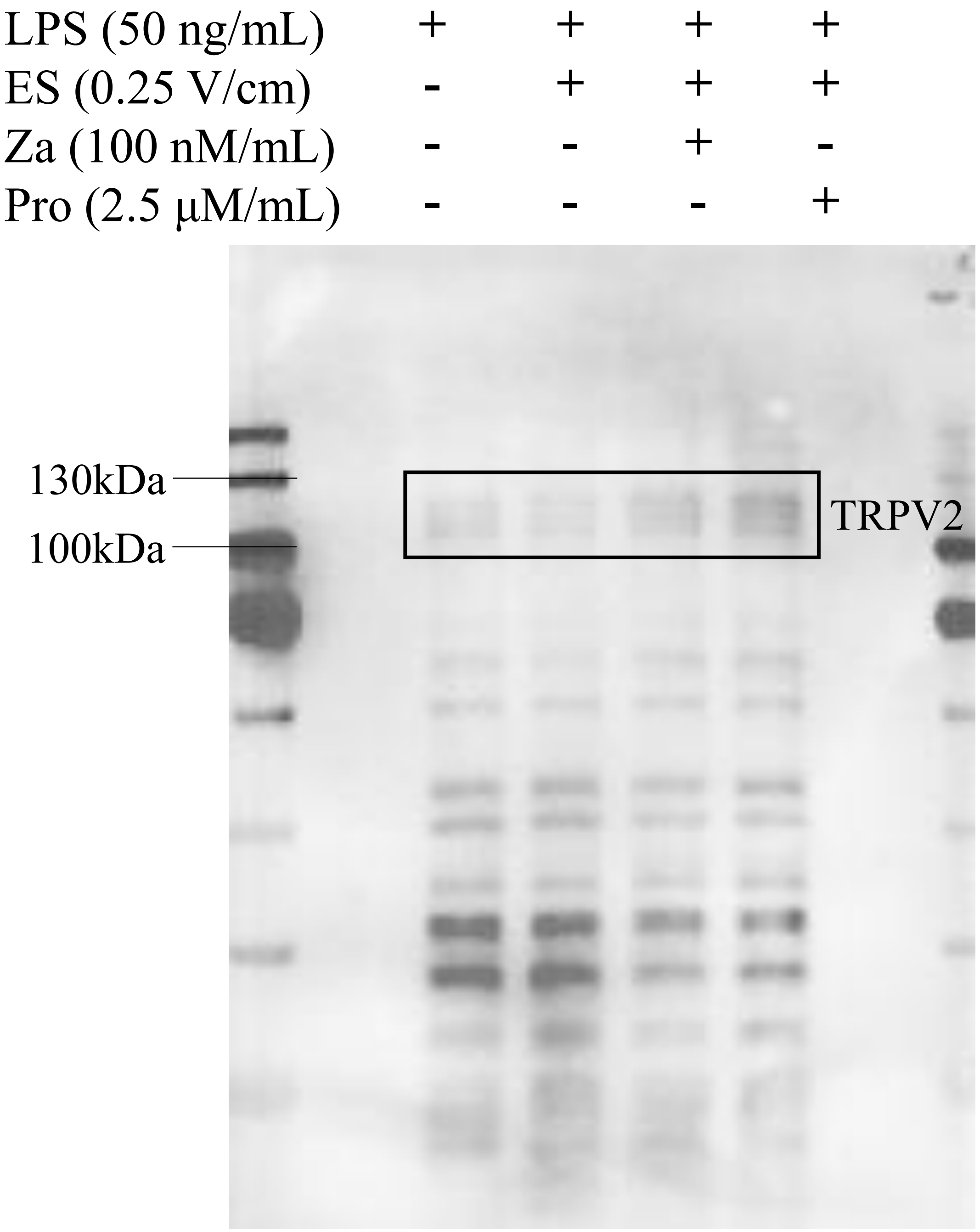
LPS (50 ng/mL) + + + +
ES (0.25 V/cm) - + + +
Za (100 nM/mL) - - + -
Pro (2.5 μM/mL) - - - +
130kDa
100kDa
TRPV2

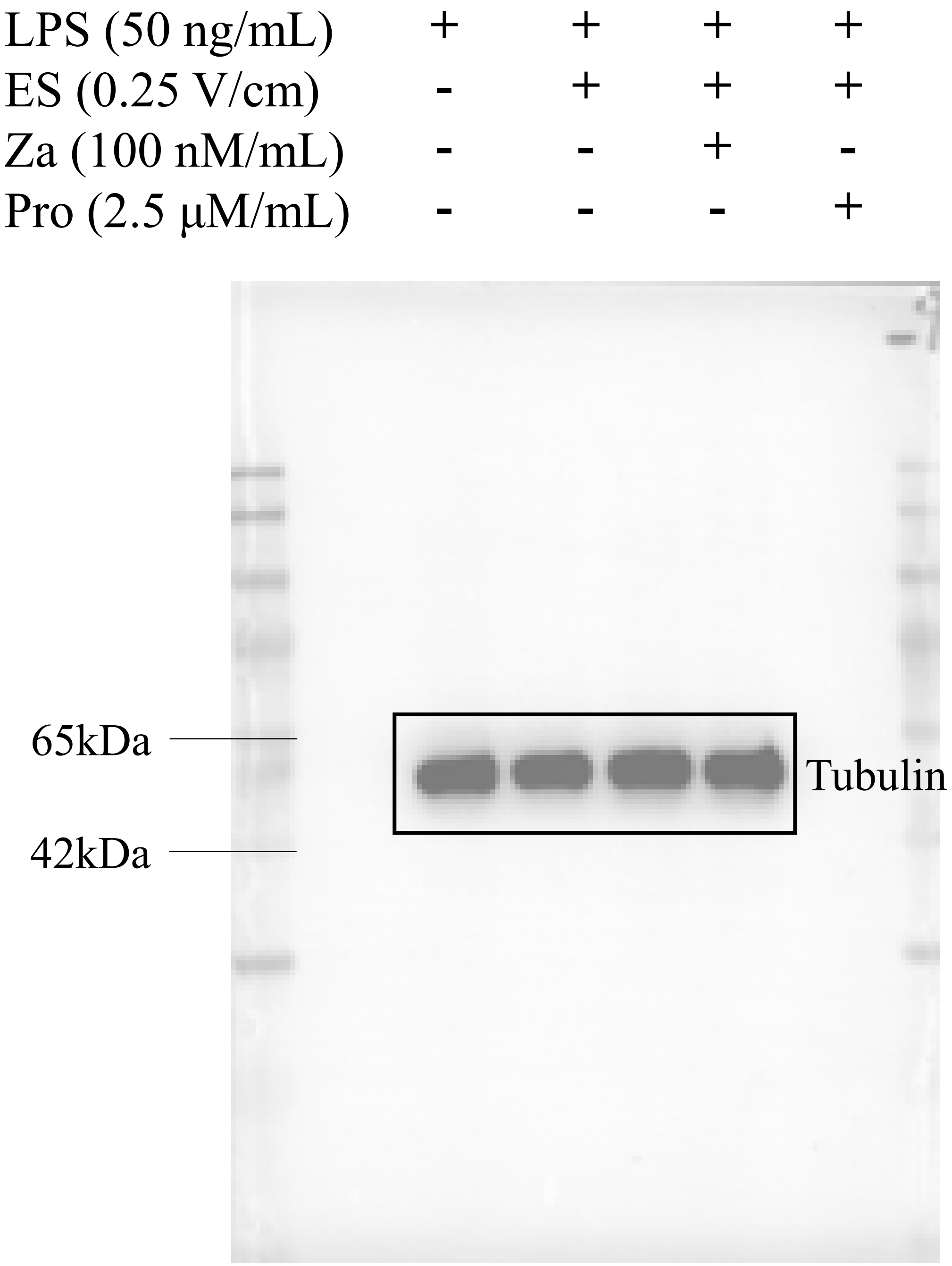
LPS (50 ng/mL) + + + +
ES (0.25 V/cm) - + + +
Za (100 nM/mL) - - + -
Pro (2.5 μM/mL) - - - +
65kDa
42kDa
Tubulin

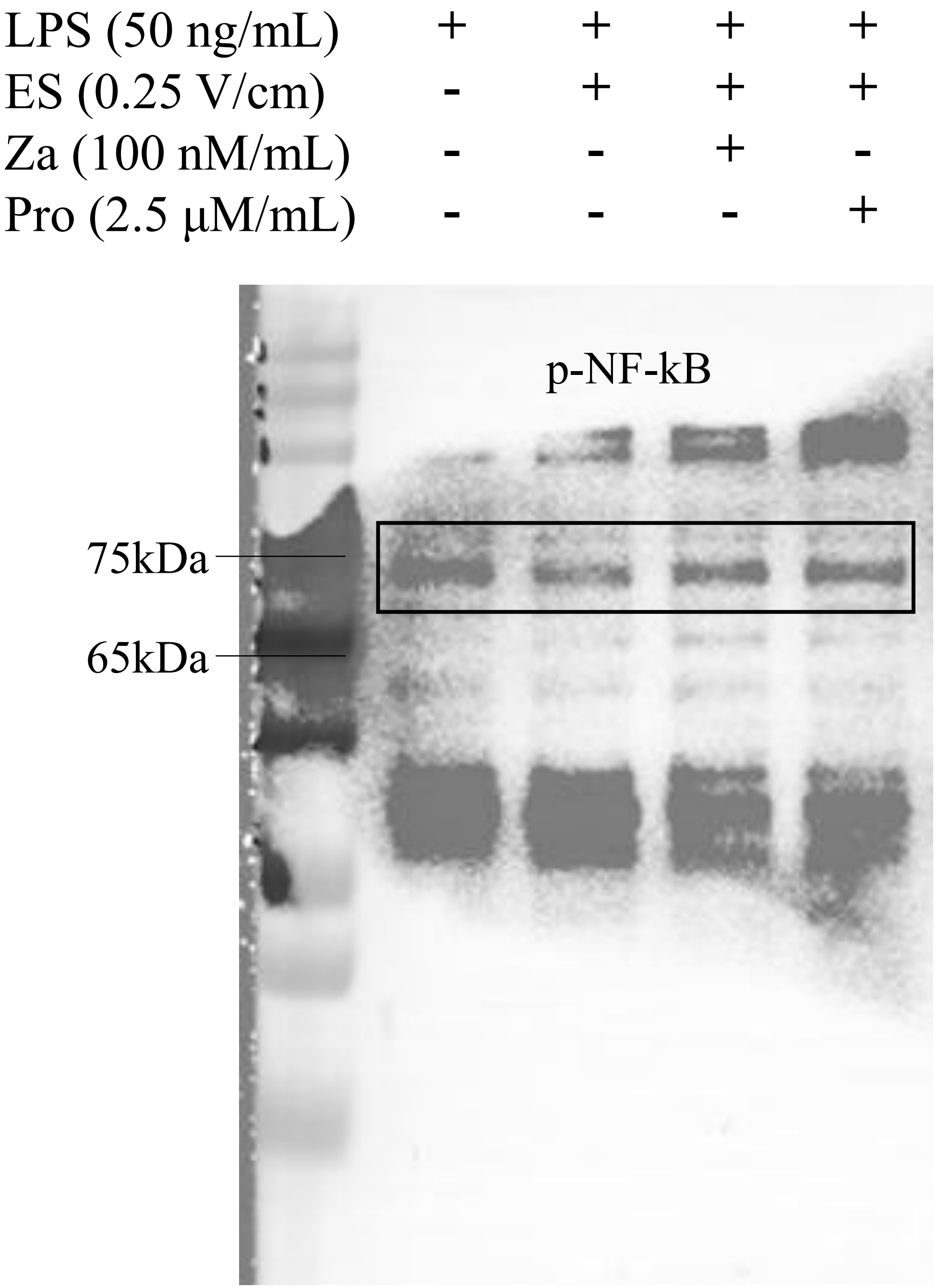

LPS (50 ng/mL) + + + +
ES (0.25 V/cm) - + + +
Za (100 nM/mL) - - + -
Pro (2.5 µM/mL) - - - +
p-NF-kB
75kDa
65kDa

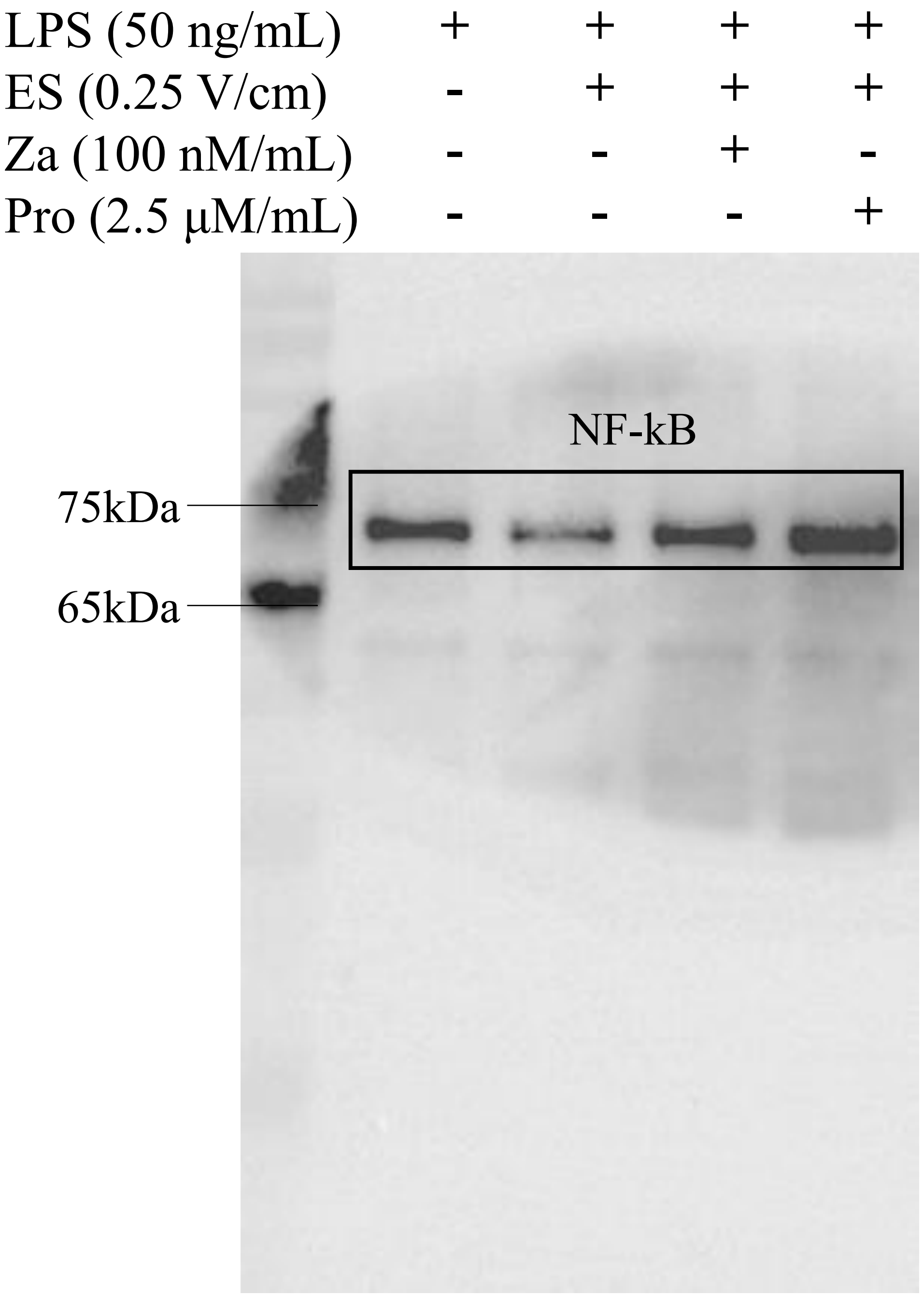
LPS (50 ng/mL) + + + +
ES (0.25 V/cm) - + + +
Za (100 nM/mL) - - + -
Pro (2.5 μM/mL) - - - +
NF-kB
75kDa
65kDa

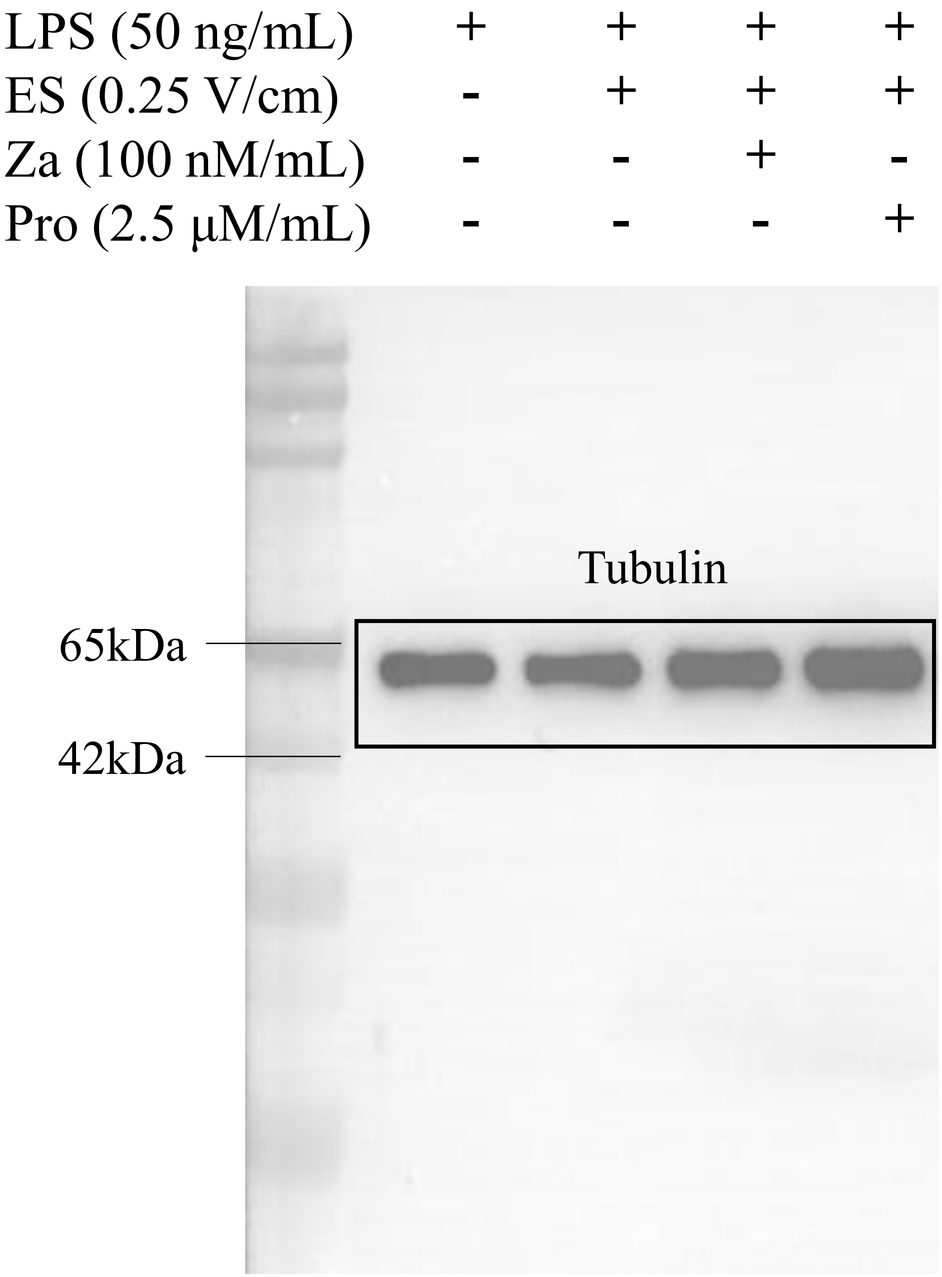
LPS (50 ng/mL) + + + +
ES (0.25 V/cm) - + + +
Za (100 nM/mL) - - + -
Pro (2.5 μM/mL) - - - +
Tubulin
65kDa
42kDa

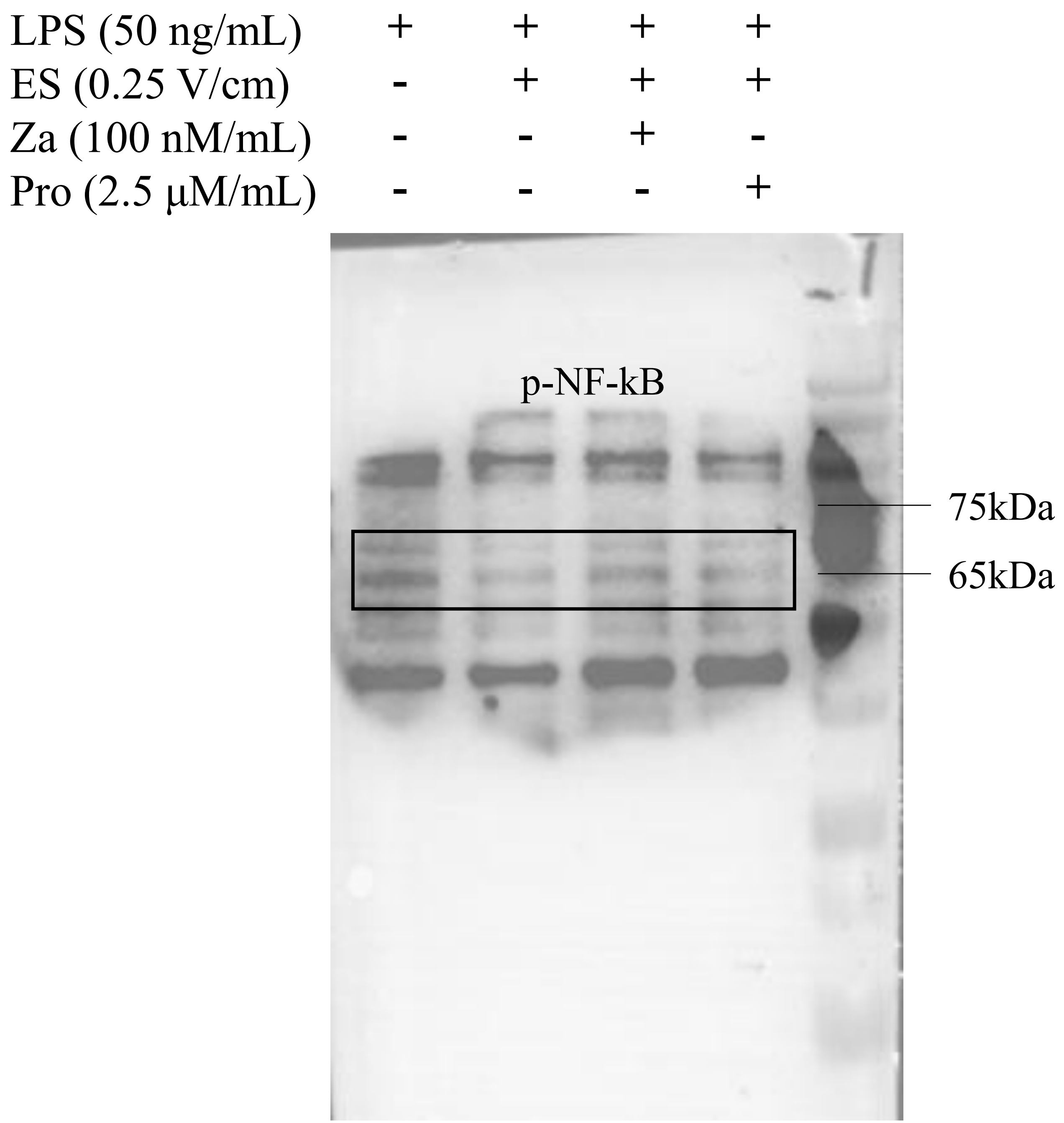
LPS (50 ng/mL) + + + +
ES (0.25 V/cm) - + + +
Za (100 nM/mL) - - + -
Pro (2.5 µM/mL) - - - +
p-NF-kB
75kDa
65kDa

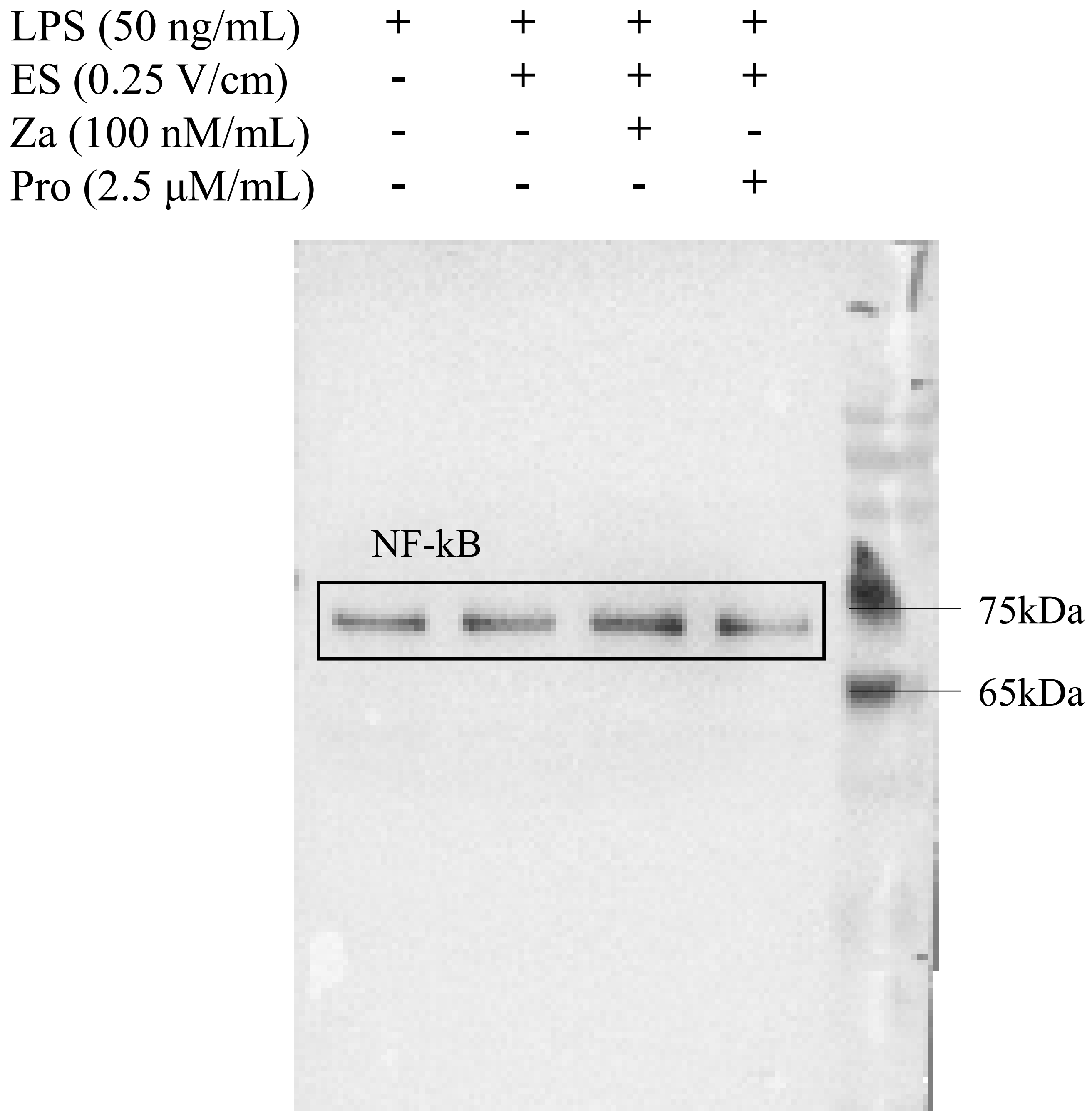

LPS (50 ng/mL) + + + +
ES (0.25 V/cm) - + + +
Za (100 nM/mL) - - + -
Pro (2.5 μM/mL) - - - +
NF-kB
75kDa
65kDa

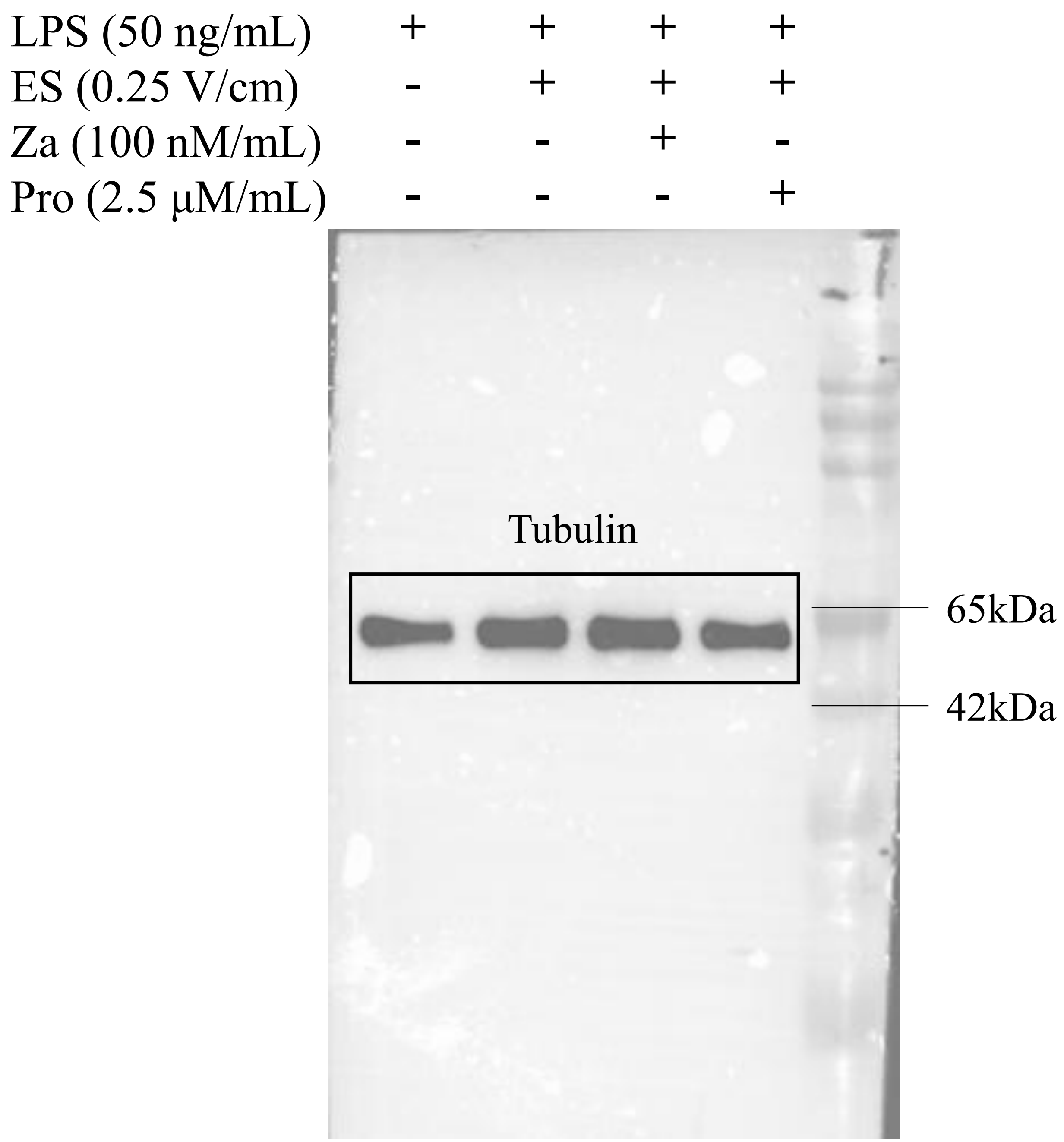
LPS (50 ng/mL) + + + +
ES (0.25 V/cm) - + + +
Za (100 nM/mL) - - + -
Pro (2.5 μM/mL) - - - +
Tubulin
65kDa
42kDa

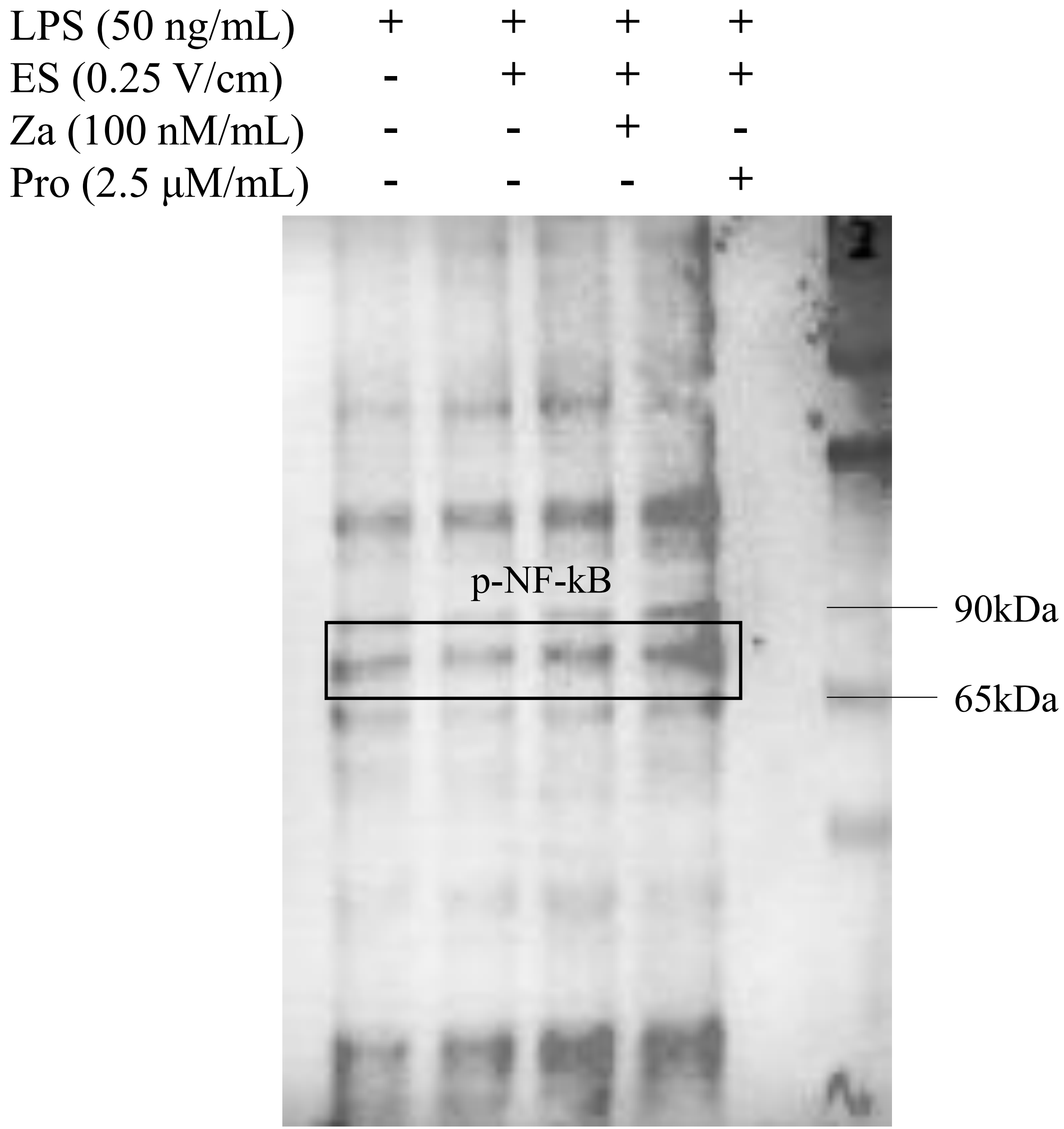
LPS (50 ng/mL) + + + +
ES (0.25 V/cm) - + + +
Za (100 nM/mL) - - + -
Pro (2.5 μM/mL) - - - +
p-NF-kB
90kDa
65kDa

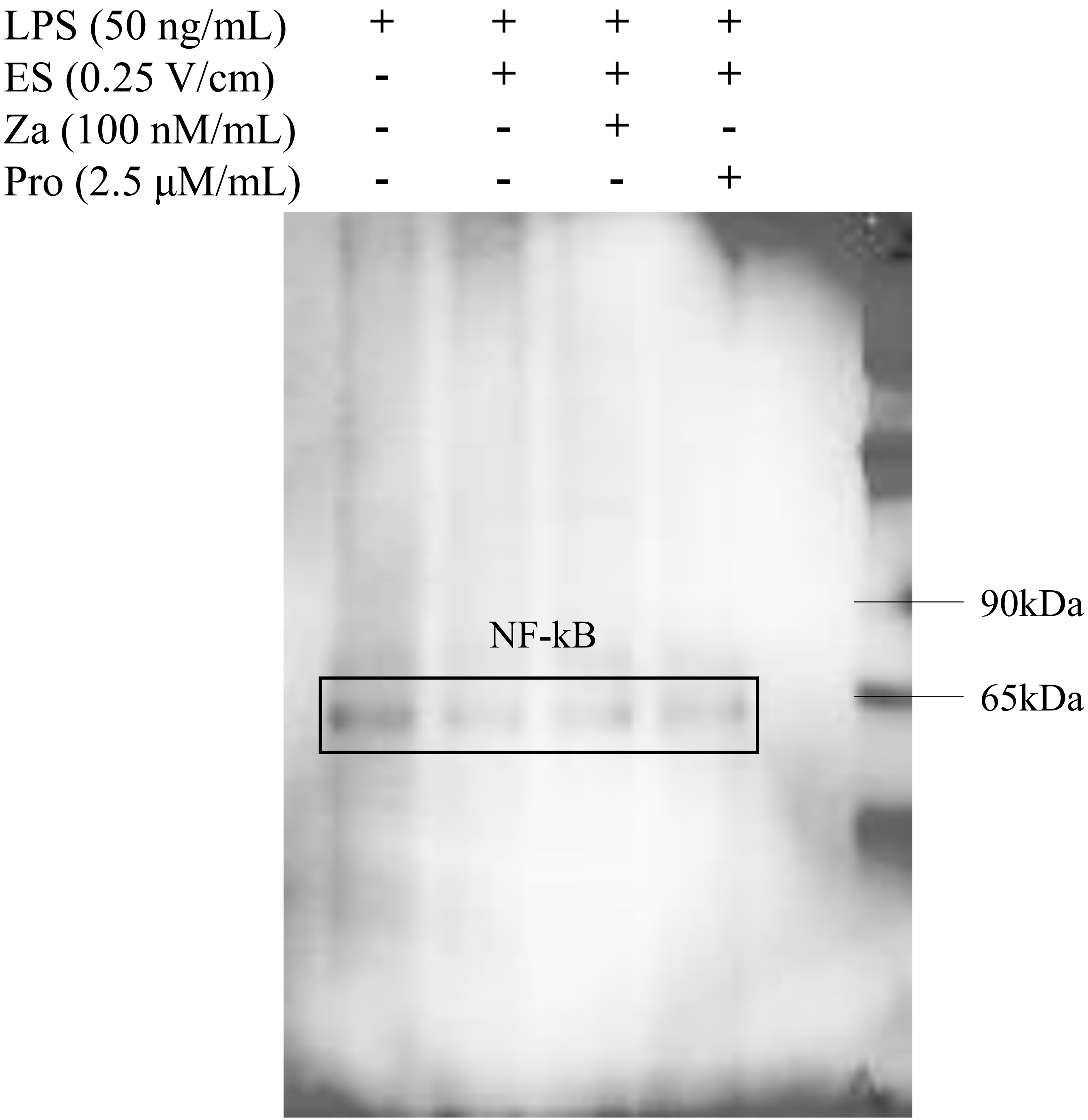
LPS (50 ng/mL) + + + +
ES (0.25 V/cm) - + + +
Za (100 nM/mL) - - + -
Pro (2.5 μM/mL) - - - +
NF-kB
90kDa
65kDa

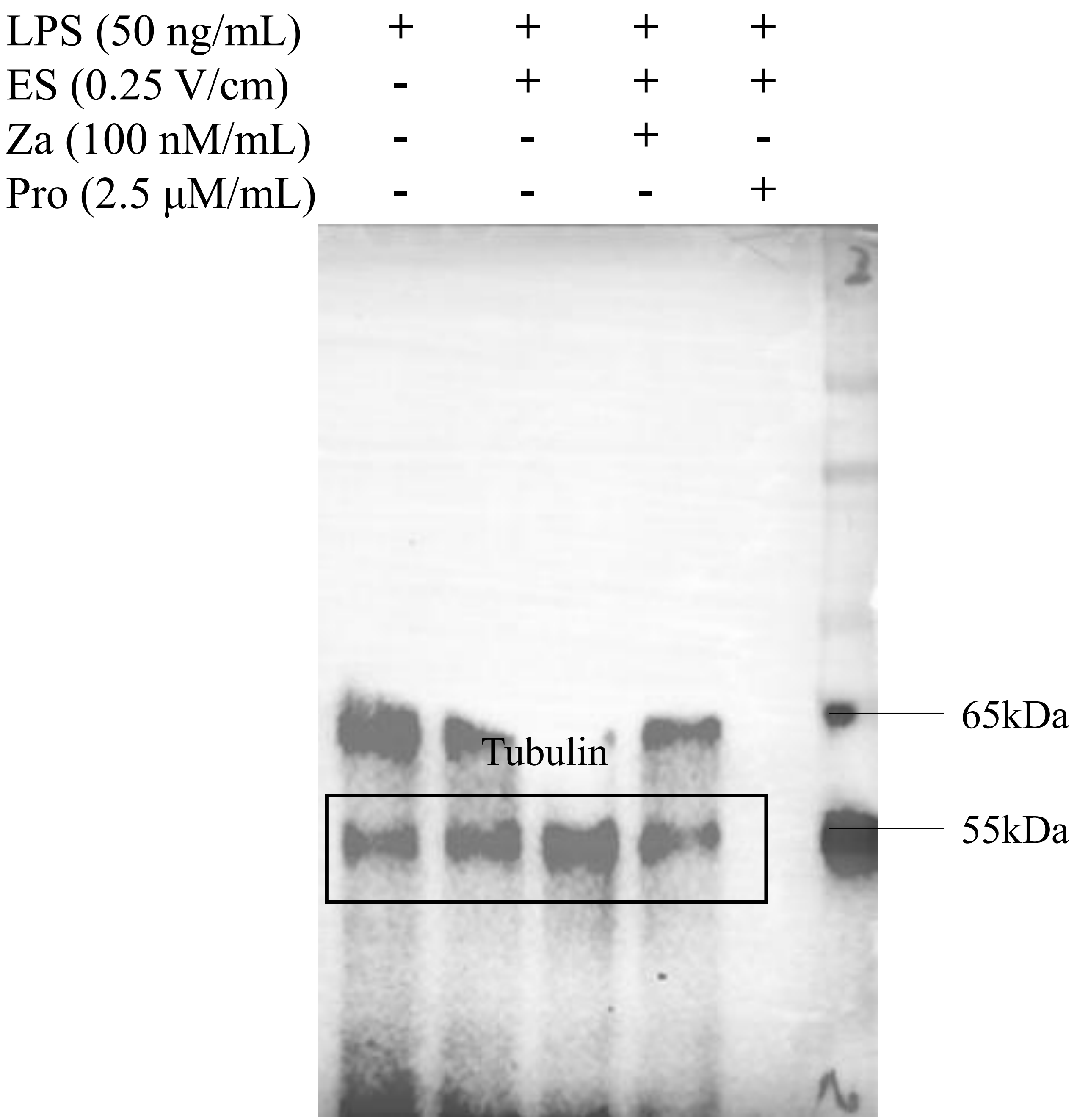
LPS (50 ng/mL) + + + +
ES (0.25 V/cm) - + + +
Za (100 nM/mL) - - + -
Pro (2.5 μM/mL) - - - +
Tubulin
65kDa
55kDa

LPS (50 ng/mL)	+	+	+	+
ES (0.25 V/cm)	-	+	+	+
Za (100 nM/mL)	-	-	+	-
Pro (2.5 μM/mL)	-	-	-	+

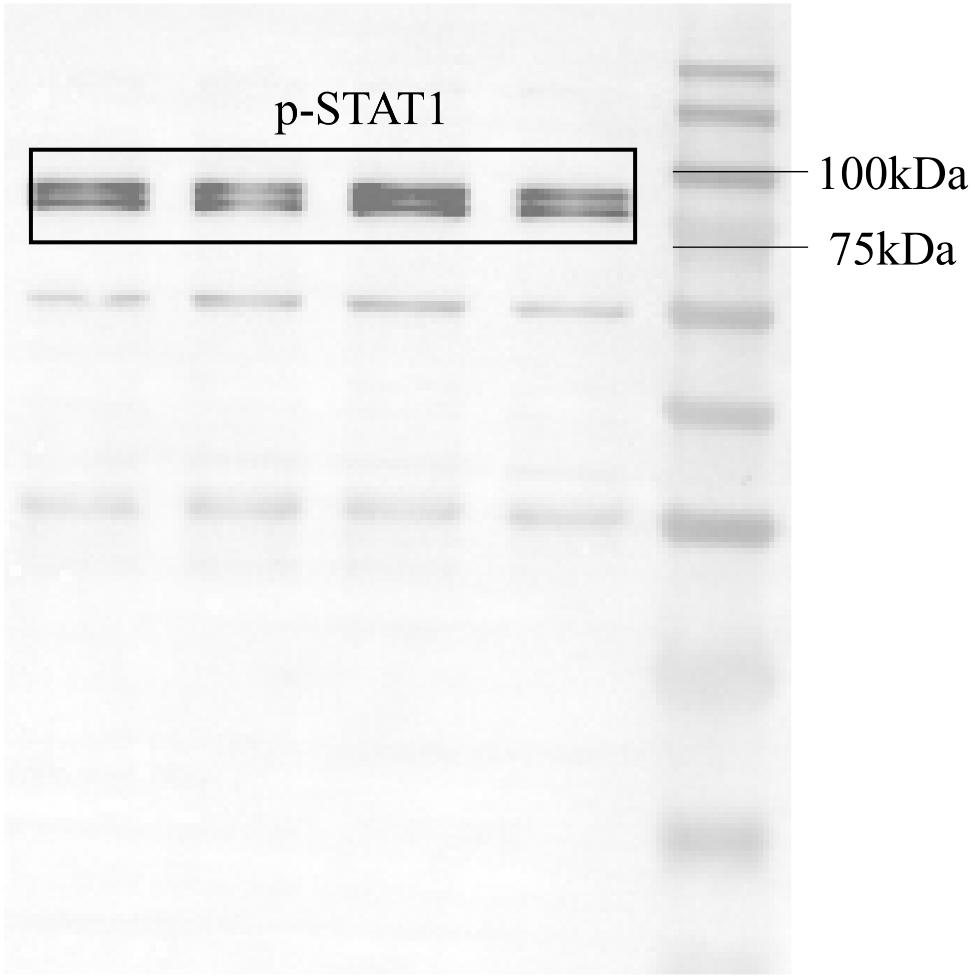

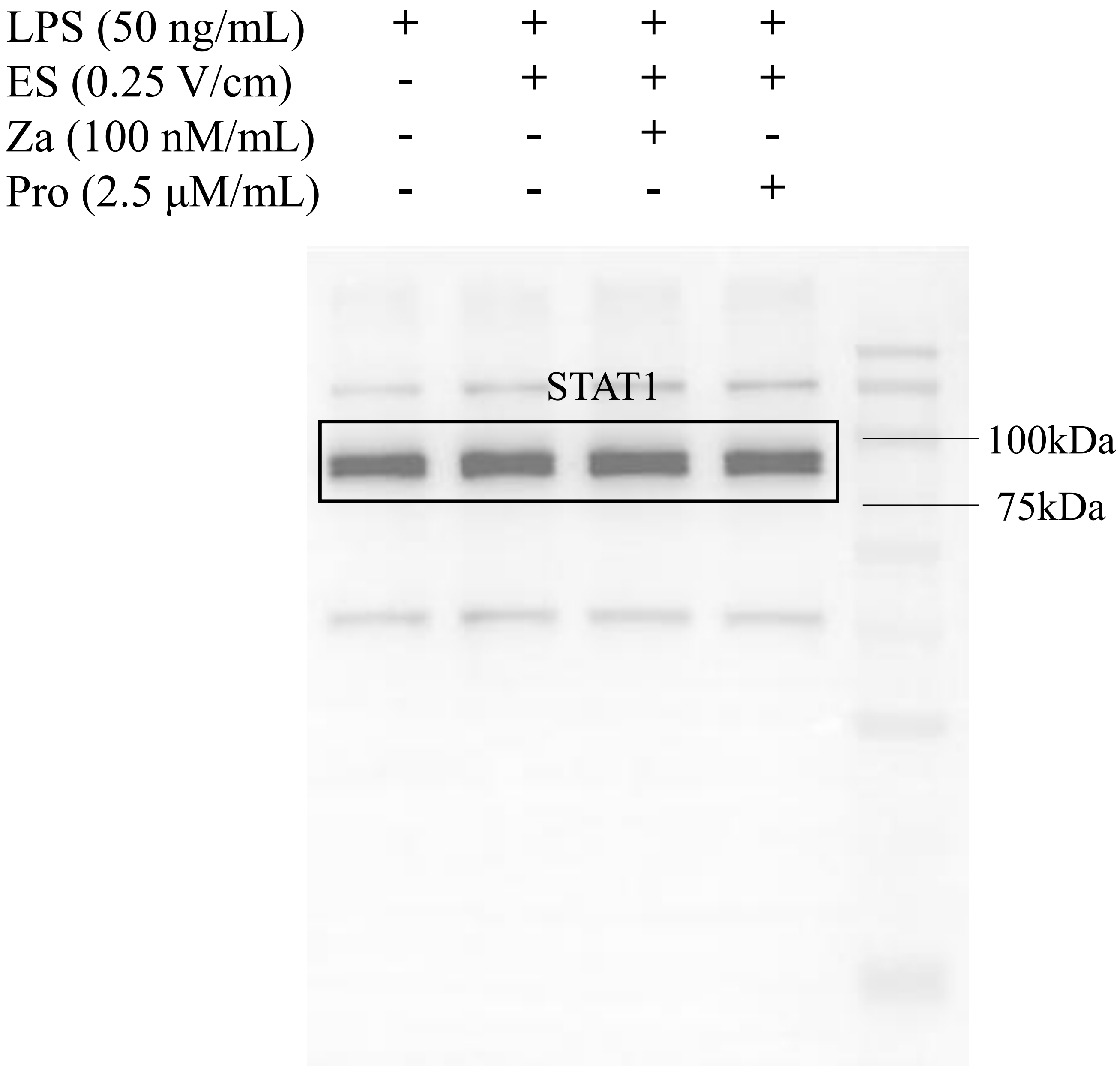
LPS (50 ng/mL) + + + +
ES (0.25 V/cm) - + + +
Za (100 nM/mL) - - + -
Pro (2.5 μM/mL) - - - +
STAT1
100kDa
75kDa

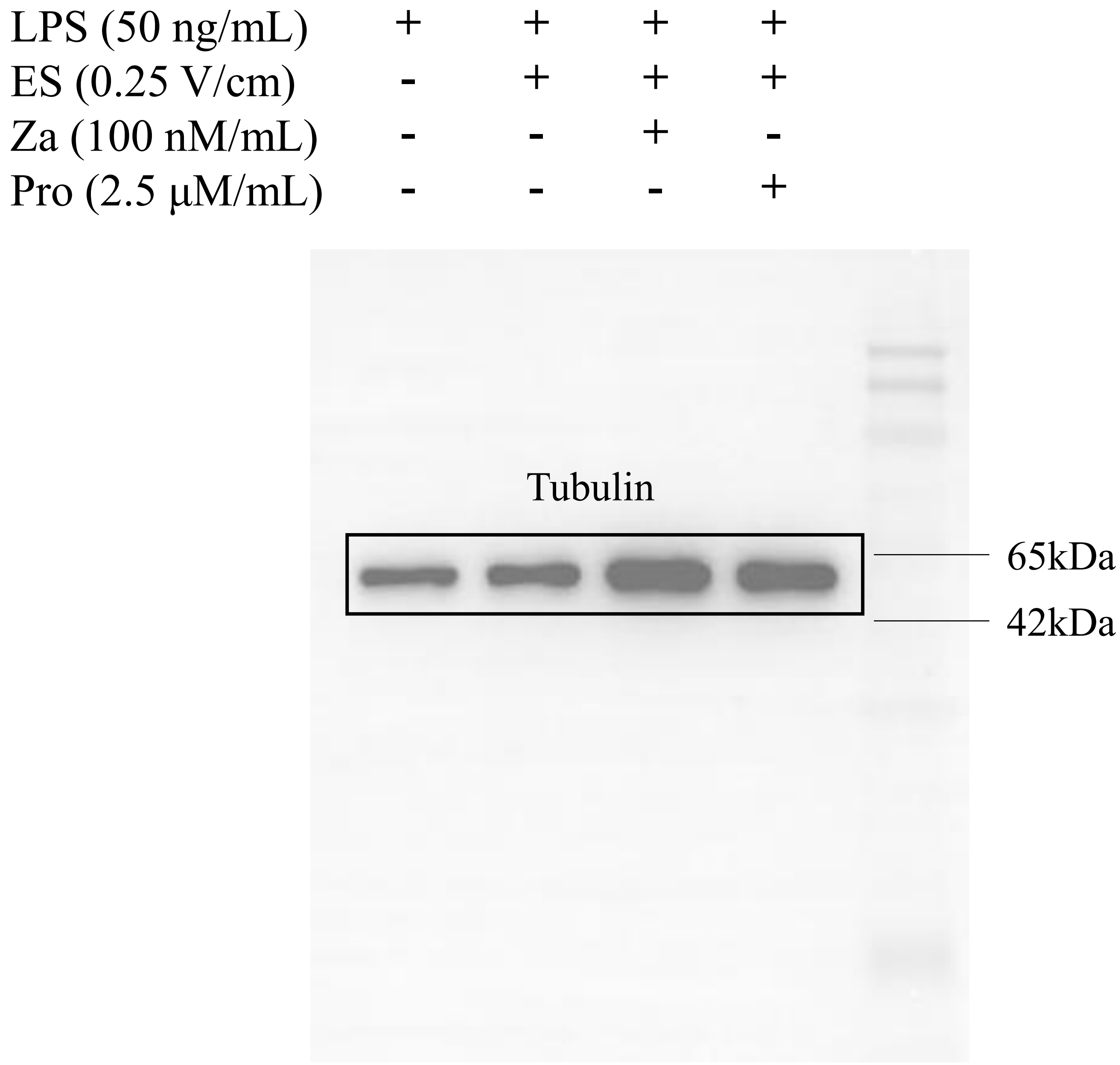
LPS (50 ng/mL) + + + +
ES (0.25 V/cm) - + + +
Za (100 nM/mL) - - + -
Pro (2.5 μM/mL) - - - +
Tubulin
65kDa
42kDa

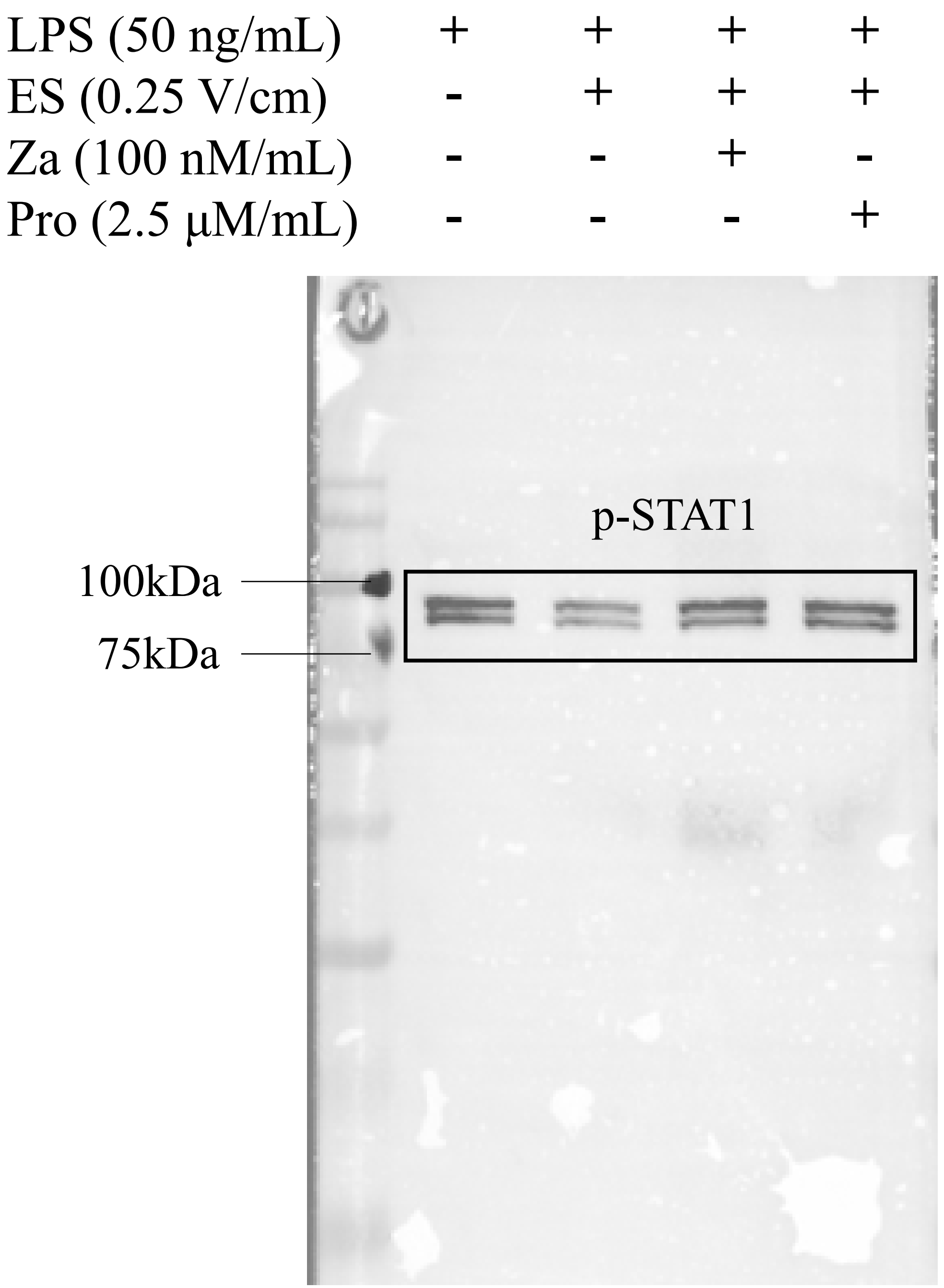
LPS (50 ng/mL) + + + +
ES (0.25 V/cm) - + + +
Za (100 nM/mL) - - + -
Pro (2.5 μM/mL) - - - +
p-STAT1
100kDa
75kDa

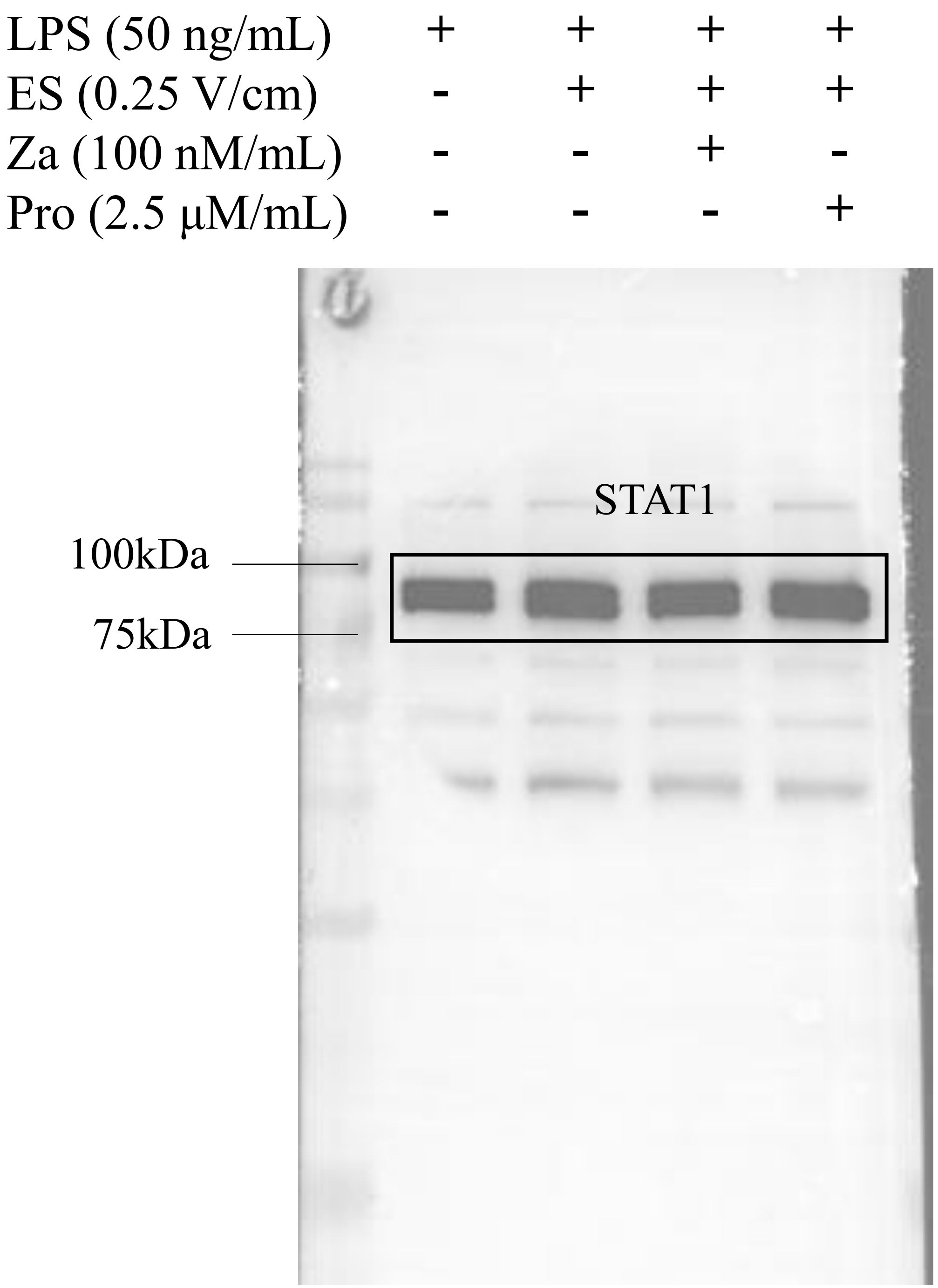

LPS (50 ng/mL) + + + +
ES (0.25 V/cm) - + + +
Za (100 nM/mL) - - + -
Pro (2.5 μM/mL) - - - +
STAT1
100kDa
75kDa

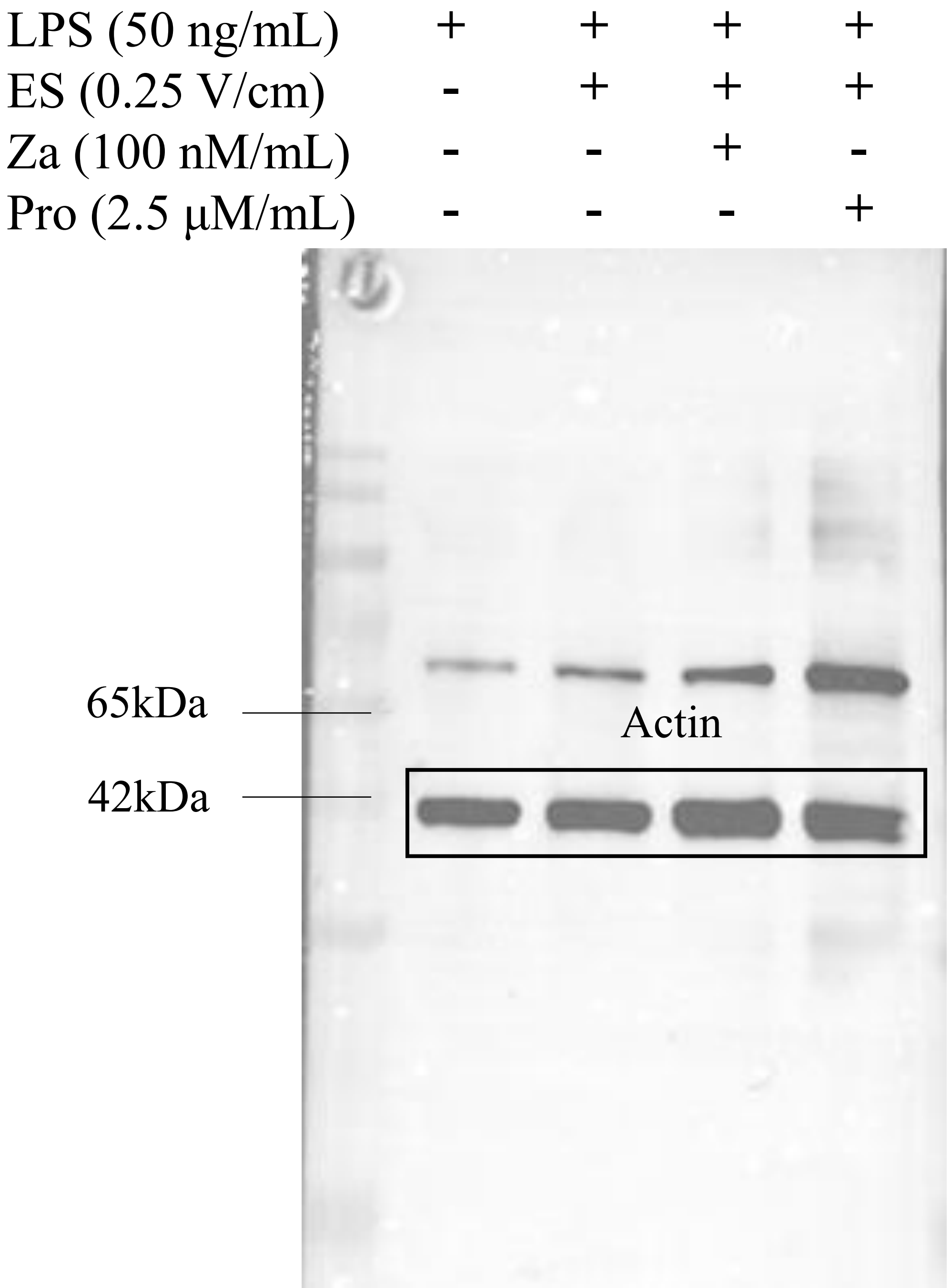
LPS (50 ng/mL) + + + +
ES (0.25 V/cm) - + + +
Za (100 nM/mL) - - + -
Pro (2.5 μM/mL) - - - +
65kDa
Actin
42kDa

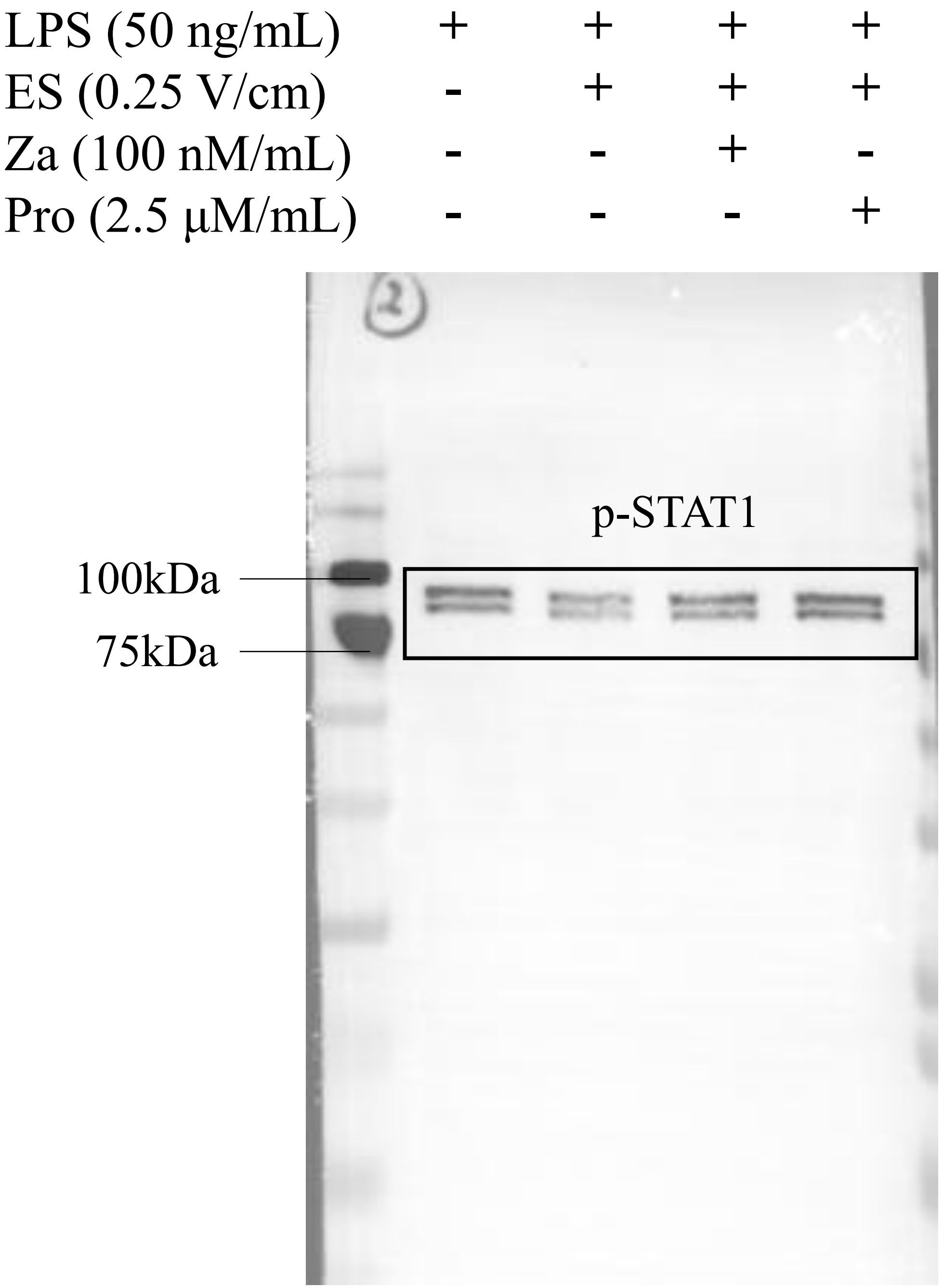
LPS (50 ng/mL) + + + +
ES (0.25 V/cm) - + + +
Za (100 nM/mL) - - + -
Pro (2.5 μM/mL) - - - +
p-STAT1
100kDa
75kDa

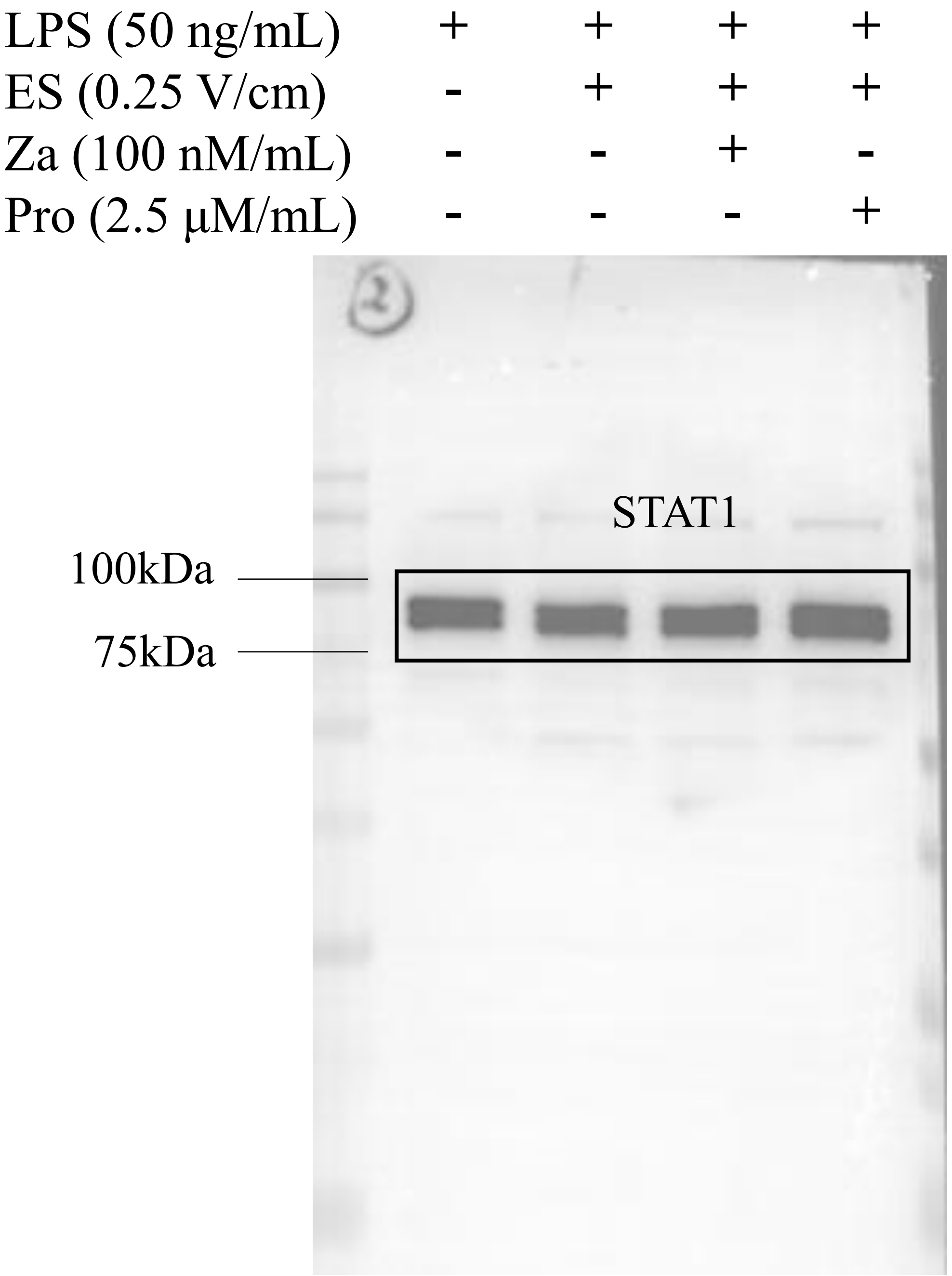
LPS (50 ng/mL) + + + +
ES (0.25 V/cm) - + + +
Za (100 nM/mL) - - + -
Pro (2.5 μM/mL) - - - +
STAT1
100kDa
75kDa

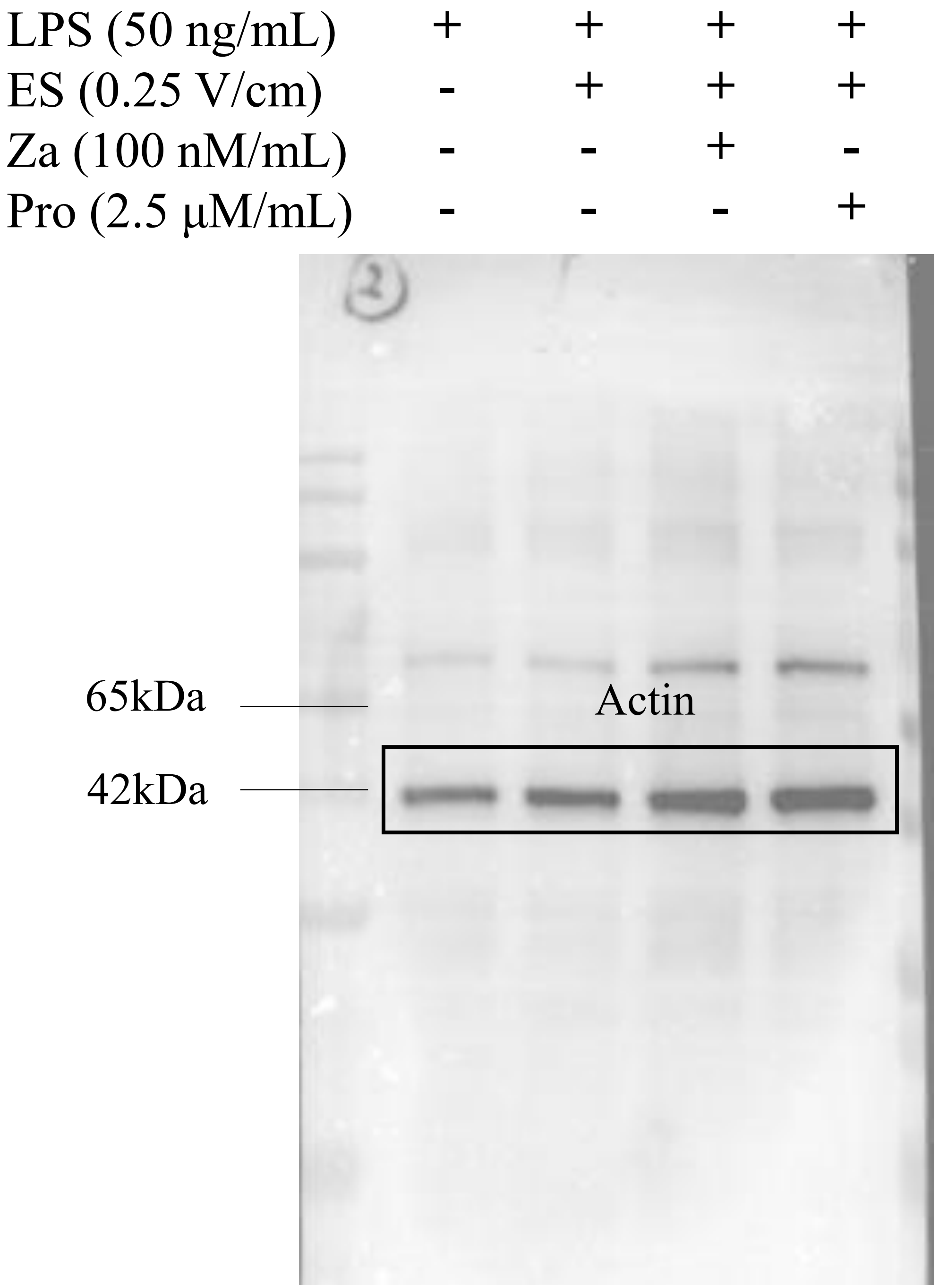
LPS (50 ng/mL) + + + +
ES (0.25 V/cm) - + + +
Za (100 nM/mL) - - + -
Pro (2.5 µM/mL) - - - +
65kDa
Actin
42kDa

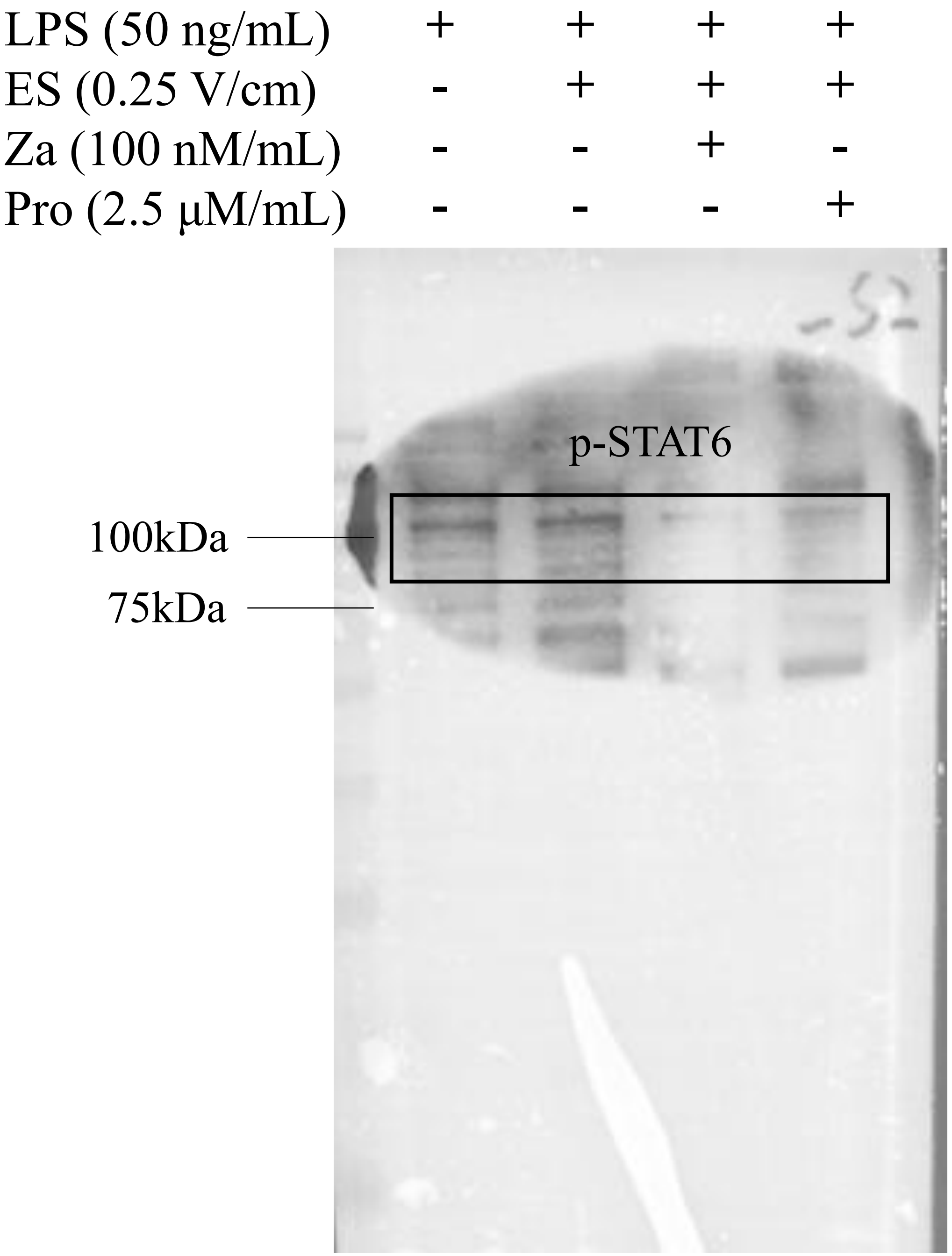
LPS (50 ng/mL) + + + +
ES (0.25 V/cm) - + + +
Za (100 nM/mL) - - + -
Pro (2.5 μM/mL) - - - +
p-STAT6
100kDa
75kDa

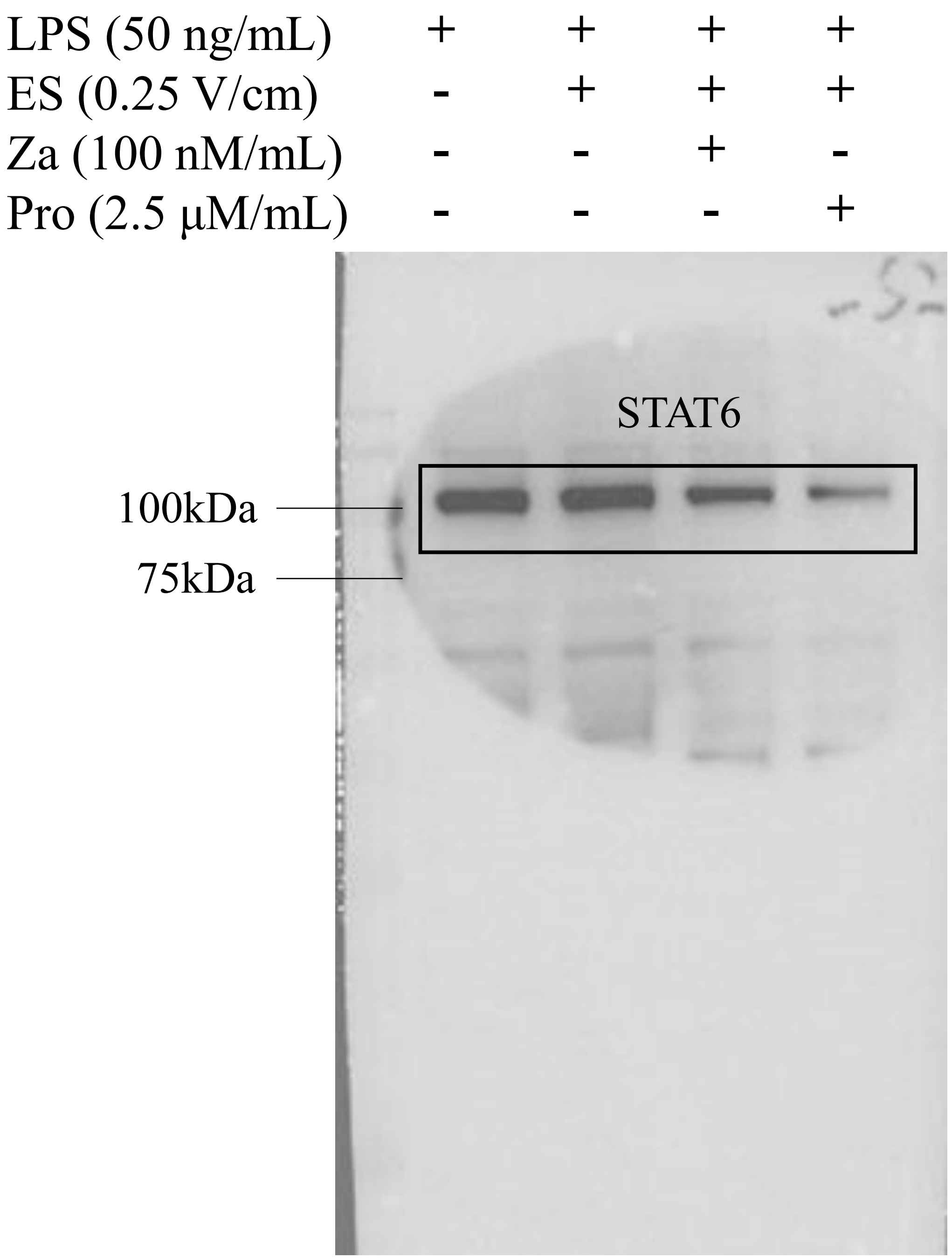
LPS (50 ng/mL) + + + +
ES (0.25 V/cm) - + + +
Za (100 nM/mL) - - + -
Pro (2.5 μM/mL) - - - +
STAT6
100kDa
75kDa

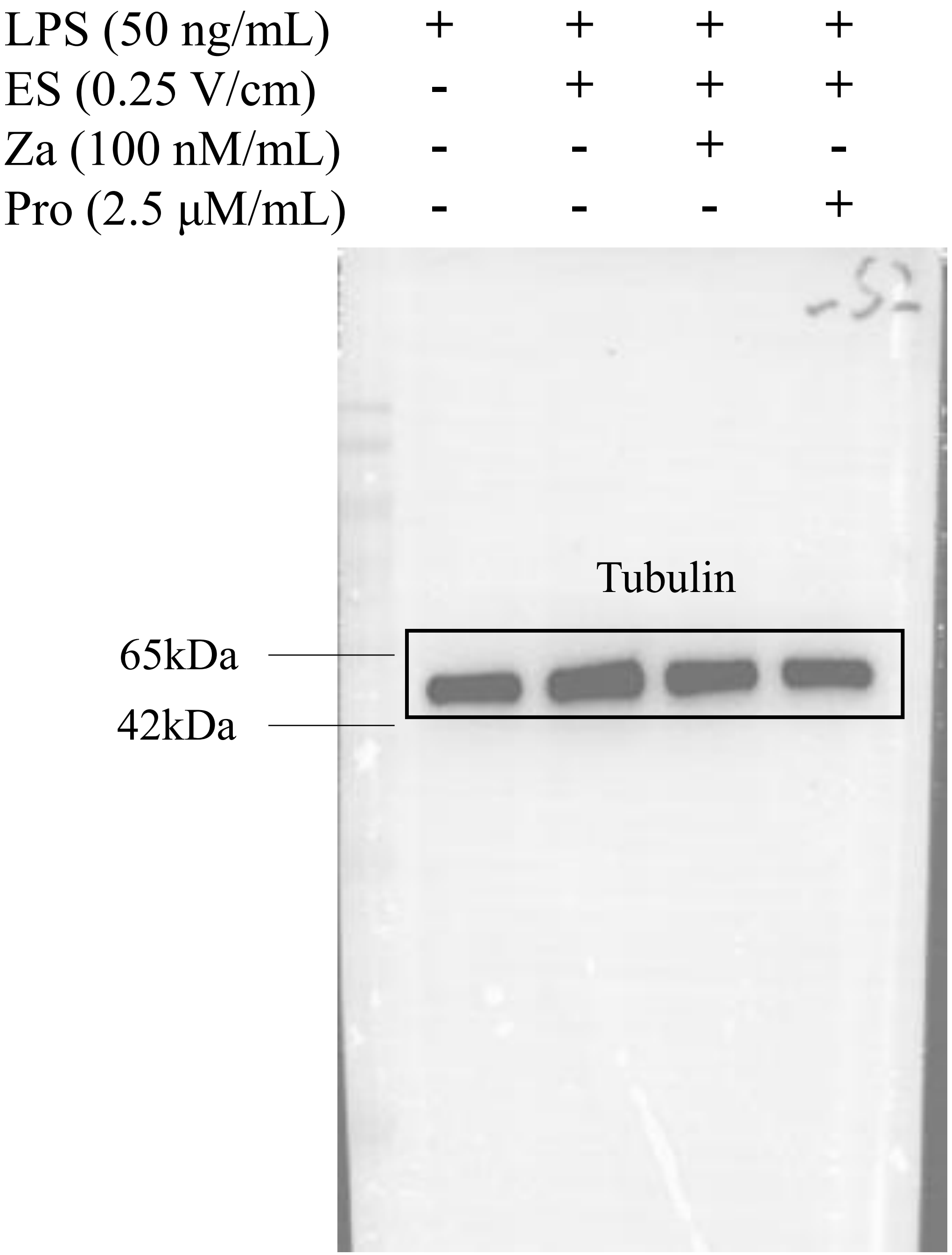
LPS (50 ng/mL) + + + +
ES (0.25 V/cm) - + + +
Za (100 nM/mL) - - + -
Pro (2.5 µM/mL) - - - +
Tubulin
65kDa
42kDa

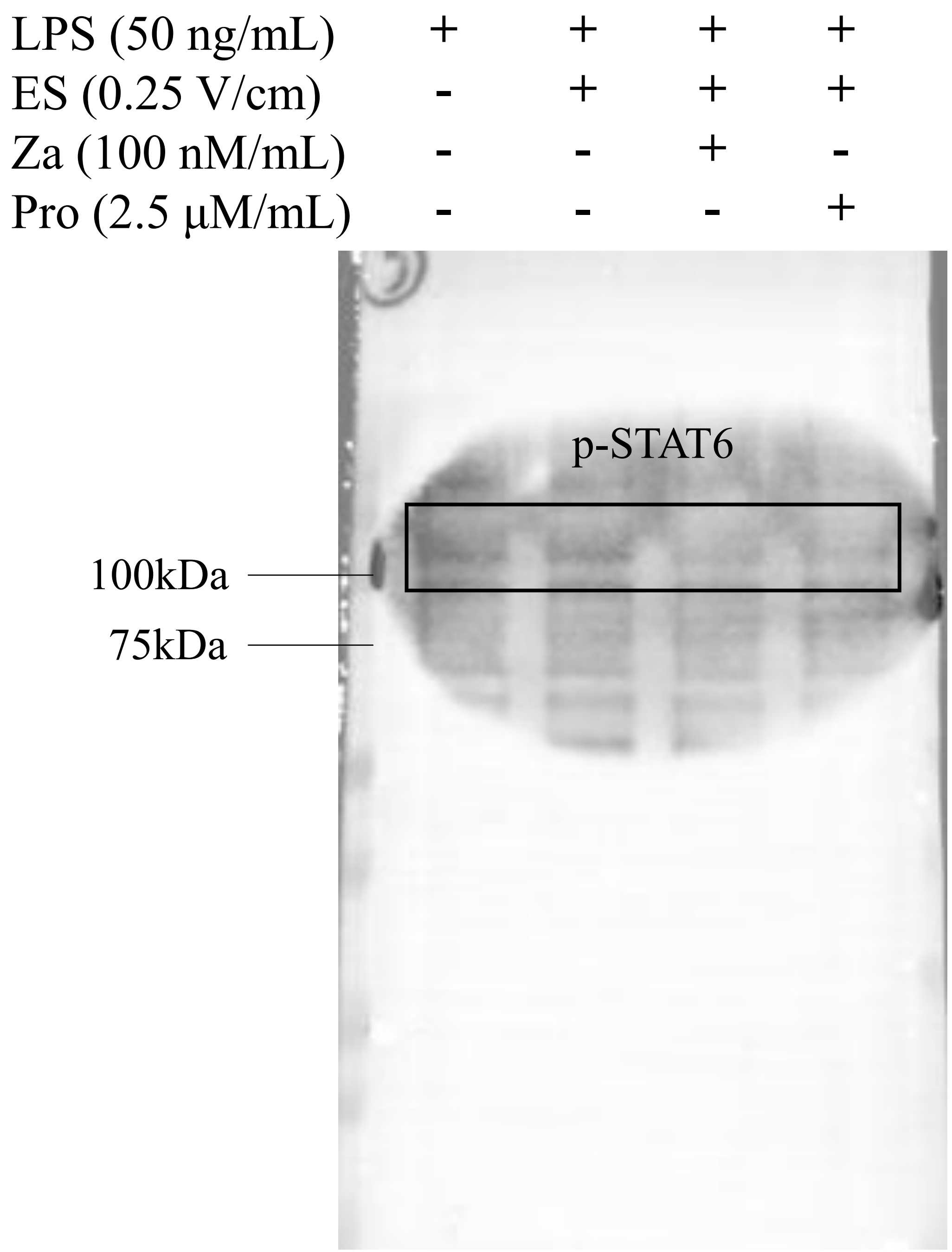

LPS (50 ng/mL) + + + +
ES (0.25 V/cm) - + + +
Za (100 nM/mL) - - + -
Pro (2.5 μM/mL) - - - +
p-STAT6
100kDa
75kDa

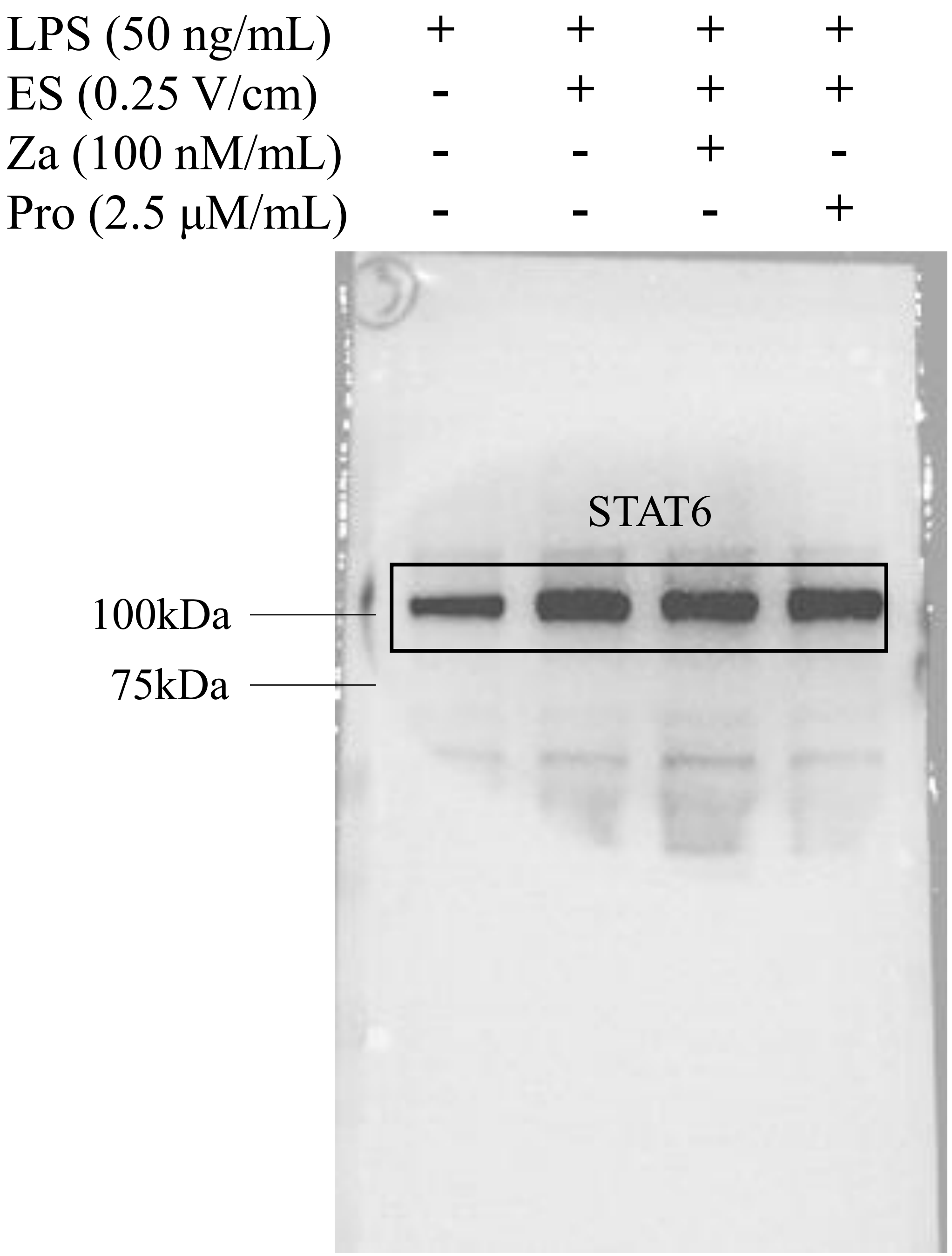
LPS (50 ng/mL) + + + +
ES (0.25 V/cm) - + + +
Za (100 nM/mL) - - + -
Pro (2.5 μM/mL) - - - +
STAT6
100kDa
75kDa

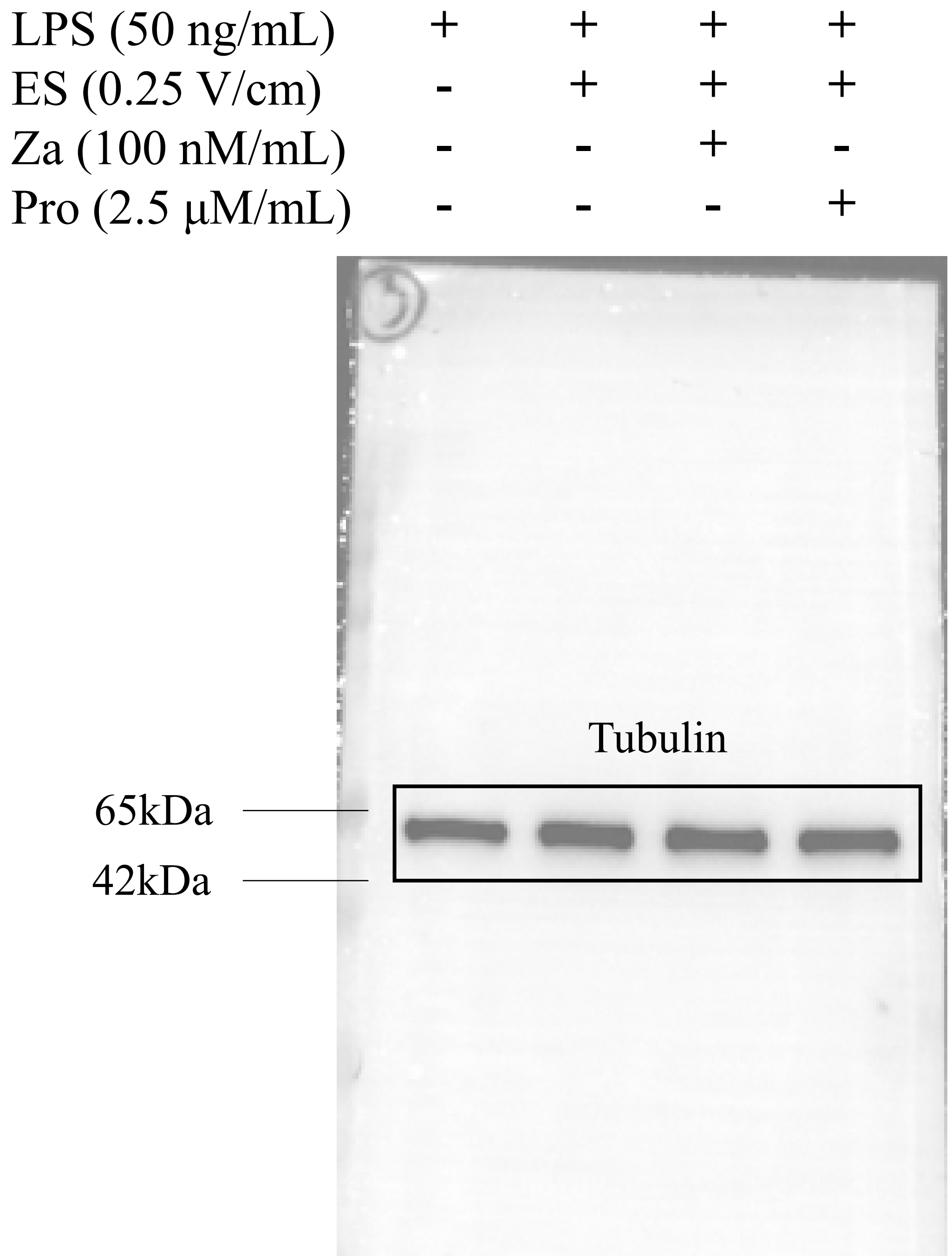
LPS (50 ng/mL) + + + +
ES (0.25 V/cm) - + + +
Za (100 nM/mL) - - + -
Pro (2.5 µM/mL) - - - +
Tubulin
65kDa
42kDa

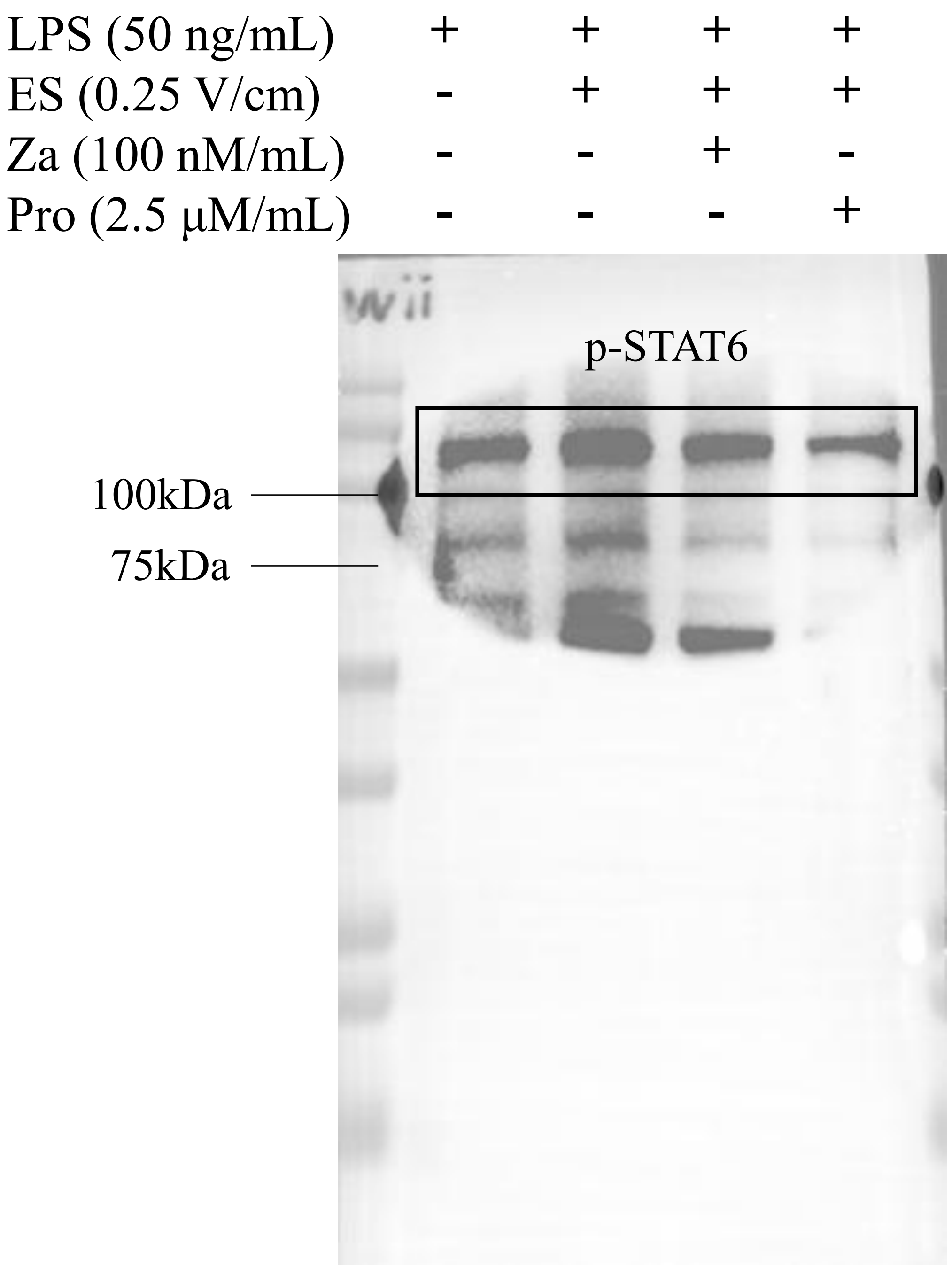
LPS (50 ng/mL) + + + +
ES (0.25 V/cm) - + + +
Za (100 nM/mL) - - + -
Pro (2.5 μM/mL) - - - +
p-STAT6
100kDa
75kDa

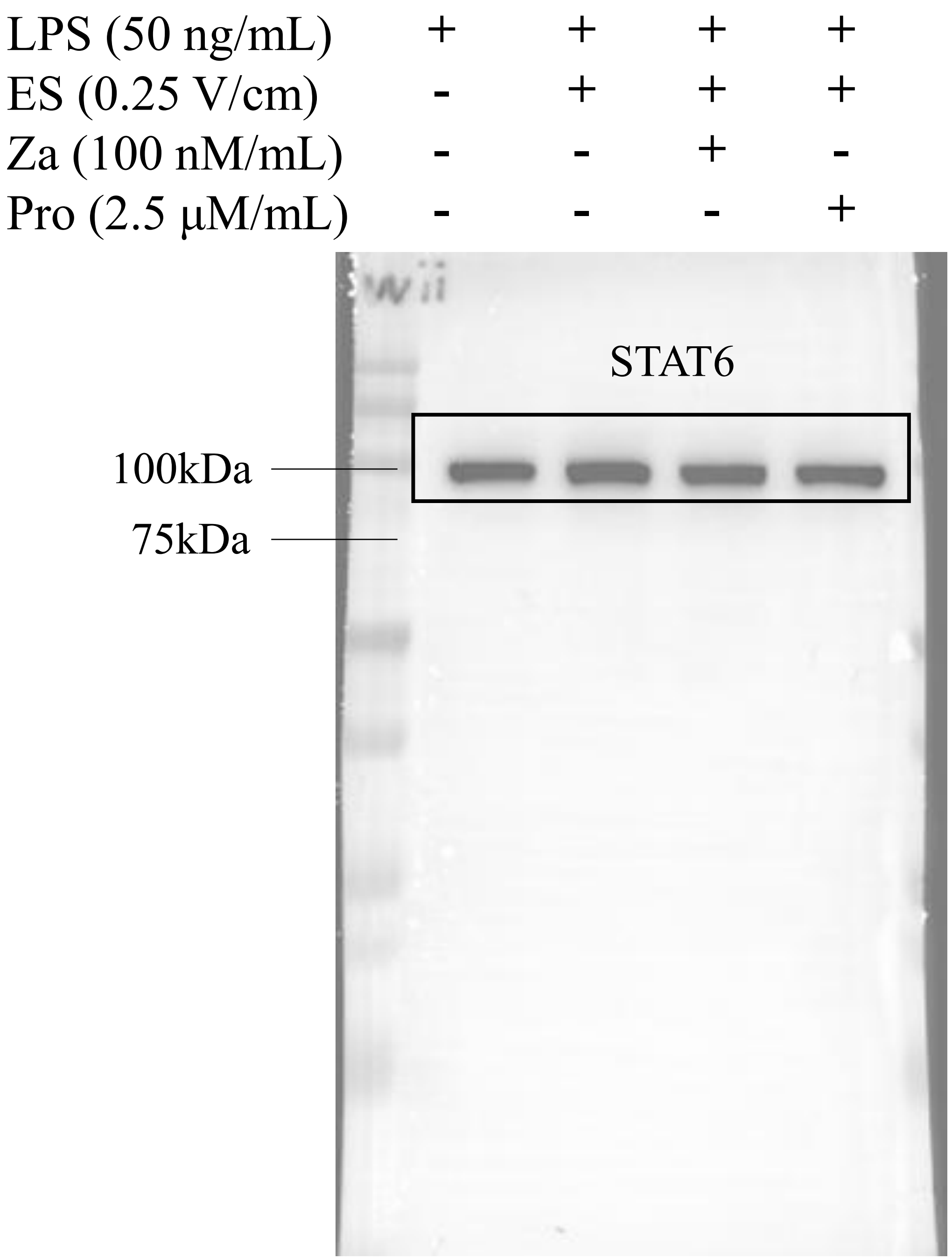
LPS (50 ng/mL) + + + +
ES (0.25 V/cm) - + + +
Za (100 nM/mL) - - + -
Pro (2.5 μM/mL) - - - +
STAT6
100kDa
75kDa

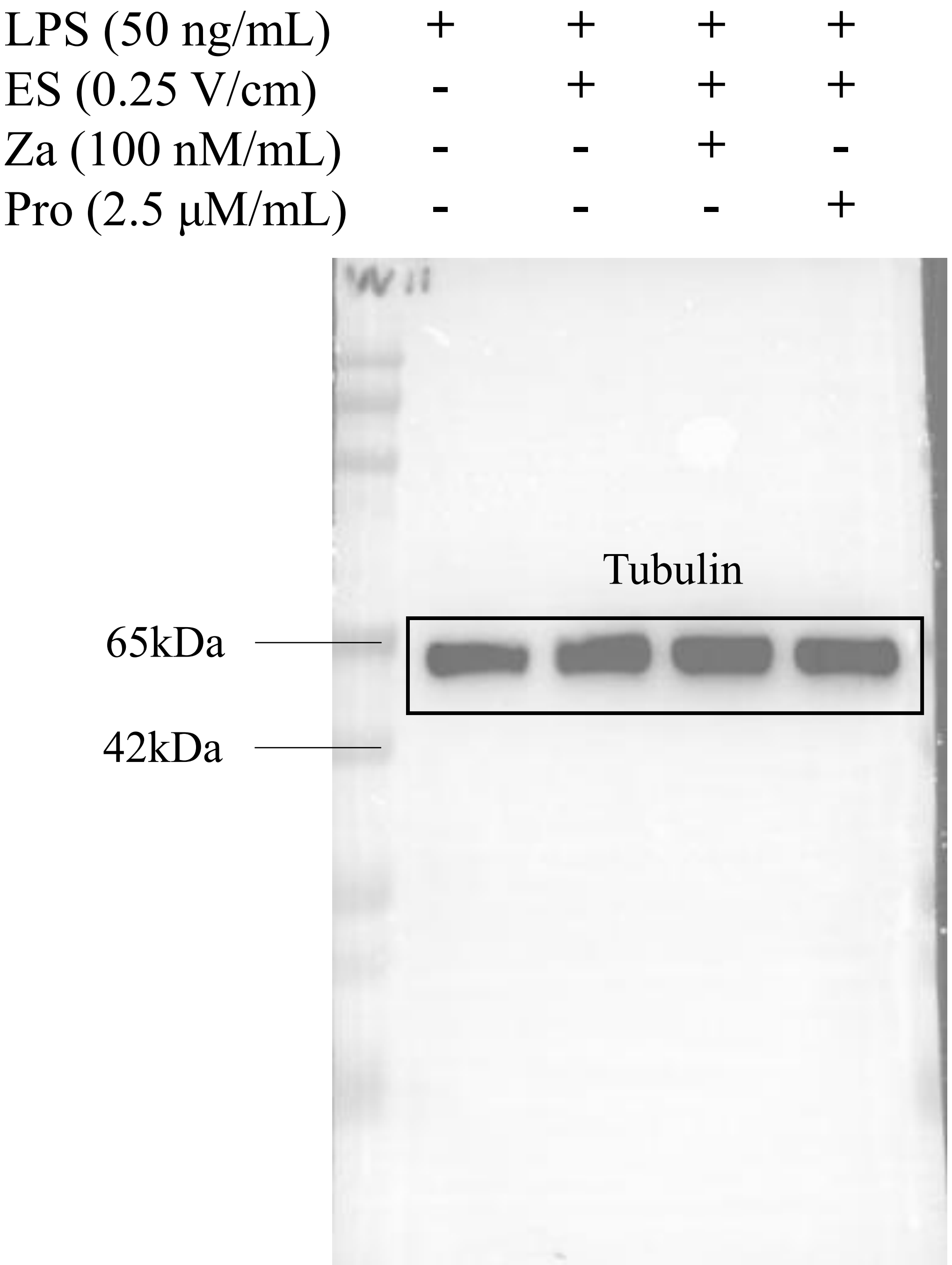

LPS (50 ng/mL) + + + +
ES (0.25 V/cm) - + + +
Za (100 nM/mL) - - + -
Pro (2.5 μM/mL) - - - +
Tubulin
65kDa
42kDa
