# Supplementary material for: TENS alleviates CP/CPPS-related inflammation and pain by modulating Kir2.1-dependent macrophage polarization
Source: Front Immunol. 2026 Jan 12;16:1683500. doi: 10.3389/fimmu.2025.1683500 (PMC12857300; doi:10.3389/fimmu.2025.1683500)
Supplement: Supplementary Figure 1 — EAP rats with TENS treatment showed efficacy, specificity and safety in vivo. (a) Schematic illustration of in vivo experiment. (b) Assessment of abnormal pain sense of prostate. Point 1: The time point of the first TENS treatment. Curves of behavioral pain assessments were performed using mixed-effects models. (c) Representative HE images of bladder, spermatophore and testicles tissue. (d) Representative HE images of heart, liver, spleen, lung and kidney tissue. Scale bars: 50 μm. Data are shown as the mean ± SEM. Behavioral pain assessments of individual timepoints were performed utilizing one-way ANOVA followed by Tukey’s post-hoc test. ###p < 0.001 vs the Control group. **p < 0.01 and ***p < 0.001 vs the EAP group. (n = 3 per group). [file DataSheet2.pdf]

Supplement

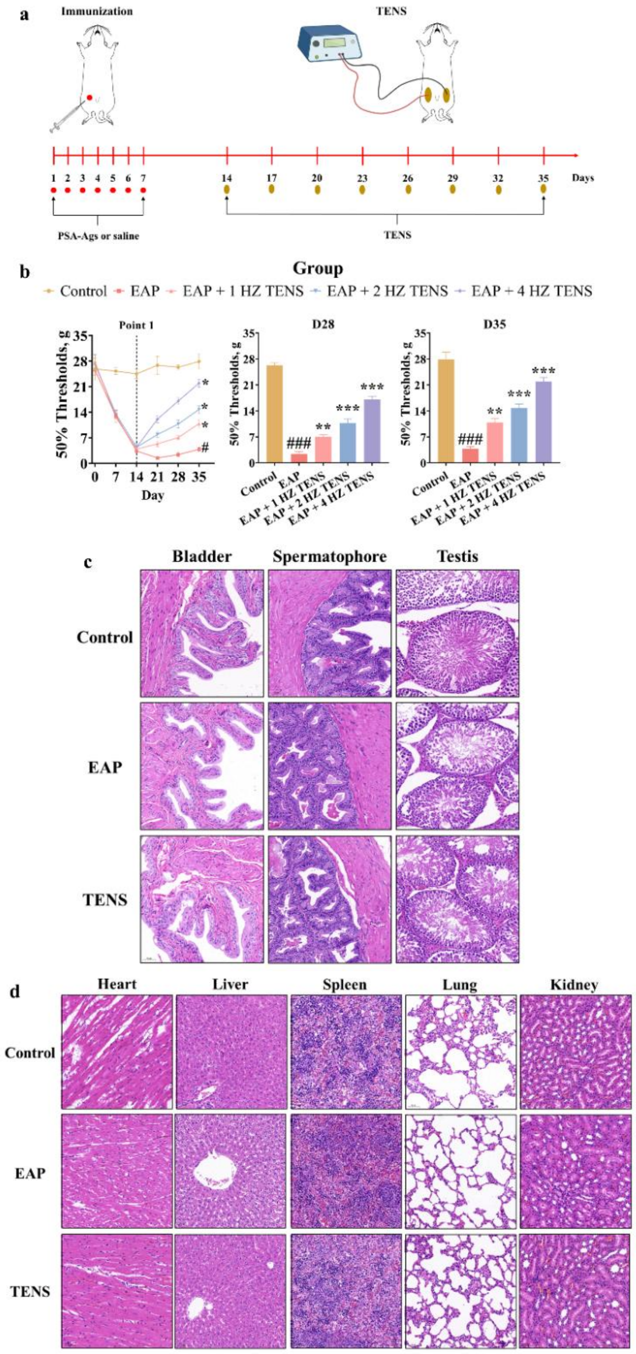

Fig. S1 EAP rats with TENS treatment showed efficacy, specificity and safety in vivo. (**a**) Schematic illustration of in vivo experiment. (**b**) Assessment of abnormal pain sense of prostate. Point 1: The time point of the first TENS treatment. Curves of behavioral pain assessments were performed using mixed-effects models. (**c**) Representative HE images of bladder, spermatophore and testicles tissue. (**d**) Representative HE images of heart, liver, spleen, lung and kidney tissue. Scale bars: 50 μm. Data are shown as the mean ± SEM. Behavioral pain assessments of individual timepoints were performed utilizing one-way ANOVA followed by Tukey's post-hoc test. ###p < 0.001 vs the Control group. **p < 0.01 and ***p < 0.001 vs the EAP group. (n = 3 per group).

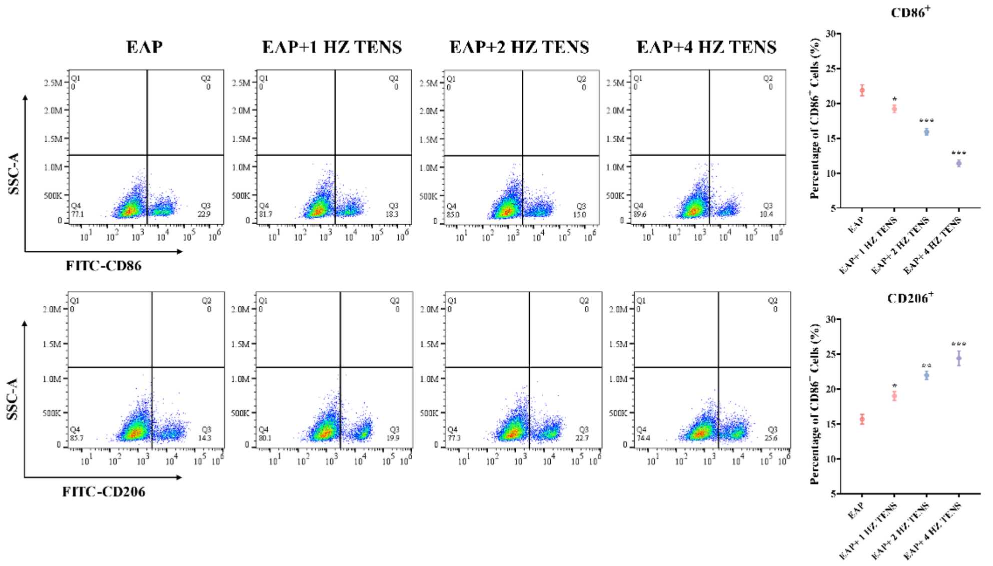

Fig. S2 Flow cytometry analysis revealed that TENS stimulation facilitated the polarization of prostate-infiltrating macrophages toward the M2 phenotype ($CD86^{-}CD206^{+}$) in rats with EAP. Measured by flow cytometry. MFI: Mean Fluorescence Intensity. Data are shown as the mean ± SEM. One-way ANOVA followed by Tukey's post-hoc test. *$p < 0.05$, **$p < 0.01$ and ***$p < 0.001$ vs the EAP group. (n = 3 per group).

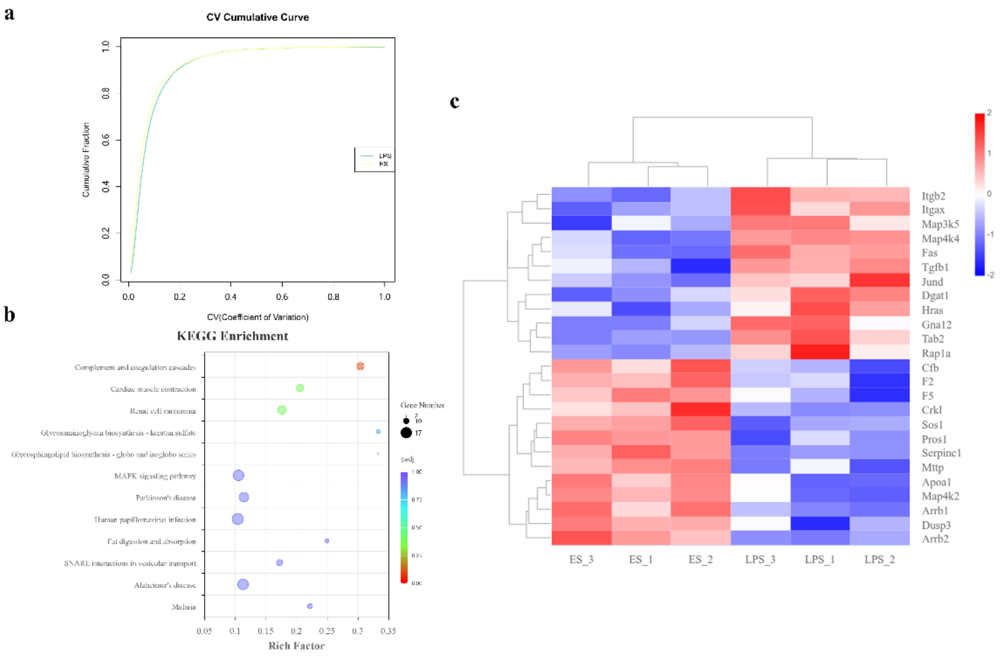

Fig. S3 MS results. (**a**) Results of repetitive CV analysis. (**b**) KEGG pathway enrichment analysis of differentially expressed proteins between the LPS and ES groups ($p < 0.05$). (**c**) Heat map of differentially clustered complement and coagulation cascades (Kumar et al., 2021), fat digestion and absorption, and MAPK signaling pathway. (n = 3 per group).

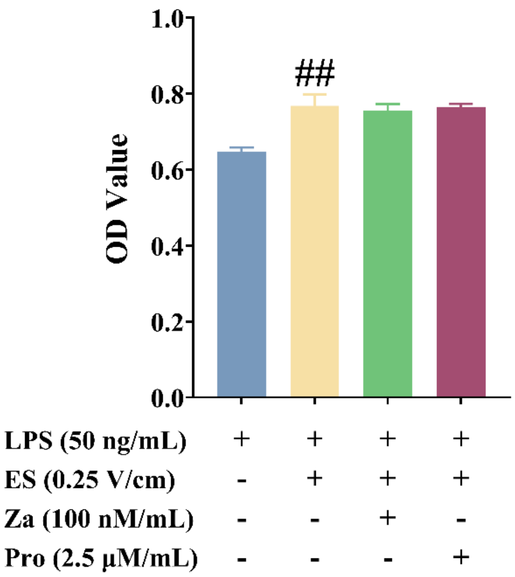

Fig. S4 Cell viability after ES and agonists tested by CCK8 assay. Data are shown as the mean ± SEM. One-way ANOVA followed by Tukey's post-hoc test. ##p < 0.01 vs the LPS group. (n = 3 per group).

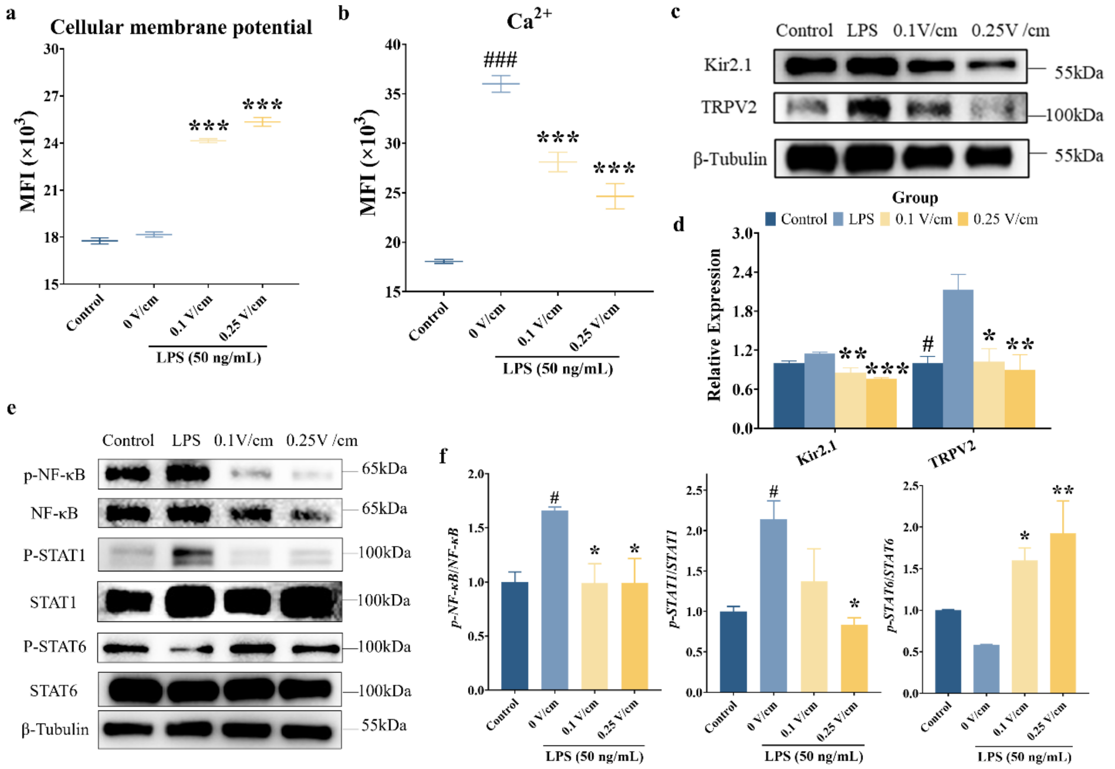

Fig. S5 Electrophysiological phenotype and polarization signaling pathway in macrophages with LPS and ES intervention. Quantitative analysis of (**a**) cellular membrane potential and intracellular Ca^{2+} concentration (**b**) in macrophages measured by flow cytometry. MFI: Mean Fluorescence Intensity. (**c**) Representative WB images of Kir2.1 and TRPV2 expression. (**d**) Quantitative analyses of Kir2.1 and TRPV2 markers expression. (**e**) Representative WB images of NF-κB/ STAT1/ STAT6 signaling pathway expression. (**f**) Quantitative analyses of NF-κB/ STAT1/ STAT6 signaling pathway expression. Data are shown as the mean ± SEM. One-way ANOVA followed by Tukey's post-hoc test. ##$p < 0.01$ and ###$p < 0.001$ vs the LPS group. *$p < 0.05$, **$p < 0.01$ and ***$p < 0.001$ vs the ES group. (n = 3 per group).

Table. S1 ELISA kits used in the experiments.

Product	Source	No. of Catalogue
**Blood samples**		
TNF-α	Jonln	JL13202
IL-1β	Jonln	JL20884
IL-6	Servicebio	GER0001
IL-10	Jonln	JL13427
COX-2	Jonln	JL21044
SP	Jonln	JL12596
PSA	Mlbio	ML003111
**culture media**		
TNF-α	Abclonal	RKO0027
IL-1β	Jonln	JL18442
IL-6	Jonln	JL20268
IL-10	Jonln	JL20242

Table. S2 Primary antibodies used in the experiments.

Product	Source	No. of Catalogue
CD86	Proteintech	13395-1-AP
TLR4	Abclonal	A5258
iNOS	Abclonal	A3774
CD206	Proteintech	18704-1-AP
CD209	Abclonal	A1466
CD163	Abclonal	A26411PM|
Kir2.1	Proteintech	19965-1-AP
TRPV2	Proteintech	68563-1-lg
p- NF-κB	Abclonal	AP1294
NF-κB	Abclonal	A19653
p-STAT1	Abclonal	AP0054
STAT1	Abclonal	A19563
p-STAT6	Absin	ABS147254
STAT6	Abclonal	A19120
β-Actin	Abclonal	AC028
β-Tubulin	Abclonal	AC030

Table. S3 Mixed-effects models for behavioral pain assessments.

	Chi-square	P value	F	Geisser-Greenhouse's epsilon	SD	Variance
	14.35	<0.001				
**Fixed effect**						
Treatment		0.003	13.48	0.4241		
**Random effects**						
Individual					6.157	37.91
Residual					4.565	20.84
